# Genome-scale metabolic model of the fission yeast *Schizosaccharomyces pombe* and the reconciliation of *in silico/in vivo* mutant growth

**DOI:** 10.1186/1752-0509-6-49

**Published:** 2012-07-05

**Authors:** Seung Bum Sohn, Tae Yong Kim, Jay H Lee, Sang Yup Lee

**Affiliations:** 1 Metabolic and Biomolecular Engineering National Research Laboratory, Department of Chemical and Biomolecular Engineering (BK21 program), Center for Systems and Synthetic Biotechnology, Institute for the BioCentury, KAIST, Daejeon, Republic of Korea; 2 Bioinformatics Research Center, KAIST, Daejeon, Republic of Korea; 3 Department of Bio and Brain Engineering and Bioinformatics Research Center, KAIST, Daejeon, Republic of Korea; 4 Department of Chemical and Biomolecular Engineering (WCU Program), KAIST, Daejeon, Republic of Korea

**Keywords:** *Schizosaccharomyces pombe*, Genome-scale metabolic model, Single-gene mutant growth, Essentiality

## Abstract

**Background:**

Over the last decade, the genome-scale metabolic models have been playing increasingly important roles in elucidating metabolic characteristics of biological systems for a wide range of applications including, but not limited to, system-wide identification of drug targets and production of high value biochemical compounds. However, these genome-scale metabolic models must be able to first predict known *in vivo* phenotypes before it is applied towards these applications with high confidence. One benchmark for measuring the *in silico* capability in predicting *in vivo* phenotypes is the use of single-gene mutant libraries to measure the accuracy of knockout simulations in predicting mutant growth phenotypes.

**Results:**

Here we employed a systematic and iterative process, designated as Reconciling *In silico/in vivo* mutaNt Growth (RING), to settle discrepancies between *in silico* prediction and *in vivo* observations to a newly reconstructed genome-scale metabolic model of the fission yeast, *Schizosaccharomyces pombe*, SpoMBEL1693. The predictive capabilities of the genome-scale metabolic model in predicting single-gene mutant growth phenotypes were measured against the single-gene mutant library of *S. pombe*. The use of RING resulted in improving the overall predictive capability of SpoMBEL1693 by 21.5%, from 61.2% to 82.7% (92.5% of the negative predictions matched the observed growth phenotype and 79.7% the positive predictions matched the observed growth phenotype).

**Conclusion:**

This study presents validation and refinement of a newly reconstructed metabolic model of the yeast *S. pombe*, through improving the metabolic model’s predictive capabilities by reconciling the *in silico* predicted growth phenotypes of single-gene knockout mutants, with experimental *in vivo* growth data.

## Background

Genome-scale metabolic models have proven themselves in a wide range of applications in the field of biotechnology, such as system-wide drug targeting, metabolic engineering of microbial systems for production of various chemicals and materials, and system-wide understanding of cellular metabolism [1-5]. Although a large majority of these genome-scale metabolic models are of prokaryotic organisms, genome-scale metabolic models of eukaryotic organisms exist and have contributed in the study of eukaryotic metabolism [2,6]. For instance, the human genome-scale metabolic model has been employed in the study of Alzheimer’s disease, giving insight into the disease and suggesting potential treatments [7]. Other eukaryotic genome-scale metabolic models, in addition to *Homo sapiens*[8,9]*,* include *Mus musculus*[10]*, Leishmania major*[11]*, Aspergillus nidulans*[12]*, Aspergillus niger*[13], *Saccharomyces cerevisiae*[6,14]*,* and *Pichia pastoris*[15].

However, eukaryotic genome-scale metabolic models are far from being complete due to the complexity of eukaryotic systems, such as the presence of intracellular organelles, requiring compartmentalization of the metabolism, and a more complex regulation and gene expression network than bacterial systems. To ensure that the metabolic model can accurately represent the biological system of interest, the predictive capabilities of the metabolic model is compared against experimental data as a means of validating the metabolic model. This standard in evaluating metabolic models is applied to different conditions for which data are available [16]. Discrepancies between the predictions made by the metabolic model, or *in silico* predictions, and experimental results are used to direct revisions to the genome-scale metabolic model to improve its predictive capabilities [17-19]. Here we present a strategy in improving the predictive capabilities of genome-scale metabolic model of single-gene knockout growth phenotypes through Reconciliation of *In silico*/*in vivo* mutaNt Growth (RING) with the single-gene knockout mutant library and apply RING on the newly reconstructed genome-scale metabolic model of the fission yeast *Schizosaccharomyces pombe* to validate and improve the metabolic model*.*

The fission yeast *S. pombe* is widely used as a model system for studying eukaryotic systems in life science research [20,21]. This yeast is also gaining acceptance in biotechnology as a cell factory platform in industrial applications [22]. It possesses a relatively small genome size for a eukaryote, 13.8 Mbp distributed over 3 chromosomes [20]. Genome studies of the yeast have identified fifty genes homologous to human genes, acquiring interest from biomedical research [20]. Furthermore, its unique cell cycle characteristics compared to other yeasts (*e.g.,* cell division through medial fission instead of budding) make it an ideal model in the studying mammalian cell cycle control. A high percentage of the research on *S. pombe* is dedicated to understanding cell cycle control in *S. pombe*, as well as other cellular functions, such as DNA repair and cellular maintenance. Little research on the metabolism of *S. pombe* is found beyond the catabolism of substrates other than glucose, ethanol production and even less on the metabolic engineering of *S. pombe*. With the introduction of a genome-scale metabolic model of *S. pombe* validated with RING, research into the metabolism of this yeast will gather momentum.

The genome-scale metabolic model of *S. pombe*, SpoMBEL1693, consists of 1693 metabolic reactions and 1744 metabolites, distributed among 8 different compartments representing the intracellular organelles. Employing the single-gene knockout mutant library of *S. pombe,* RING was applied to improve and refine SpoMBEL1693 to accurately represent the metabolic network of *S. pombe*[23]. Initial *in silico* predictions compared to the single-gene knockout mutant library resulted in a 61.2% of all predictions correctly reflected the observed phenotypes (41.4% of the predicted lethal phenotypes and 65.4% of the predicted viable phenotypes matched with their respective observed *in vivo* growth phenotypes). After analysis and reconciliation of the false predictions, SpoMBEL1693 was updated and the accuracy was improved to 82.6% of all the predictions of the single-gene knockout mutant growth phenotypes matched the observed *in vivo* phenotype (92.5% of the predicted lethal phenotypes and 79.6% of the predicted viable phenotypes matched with their respective observed *in vivo* phenotypes).

## Results

**Figure 1:**
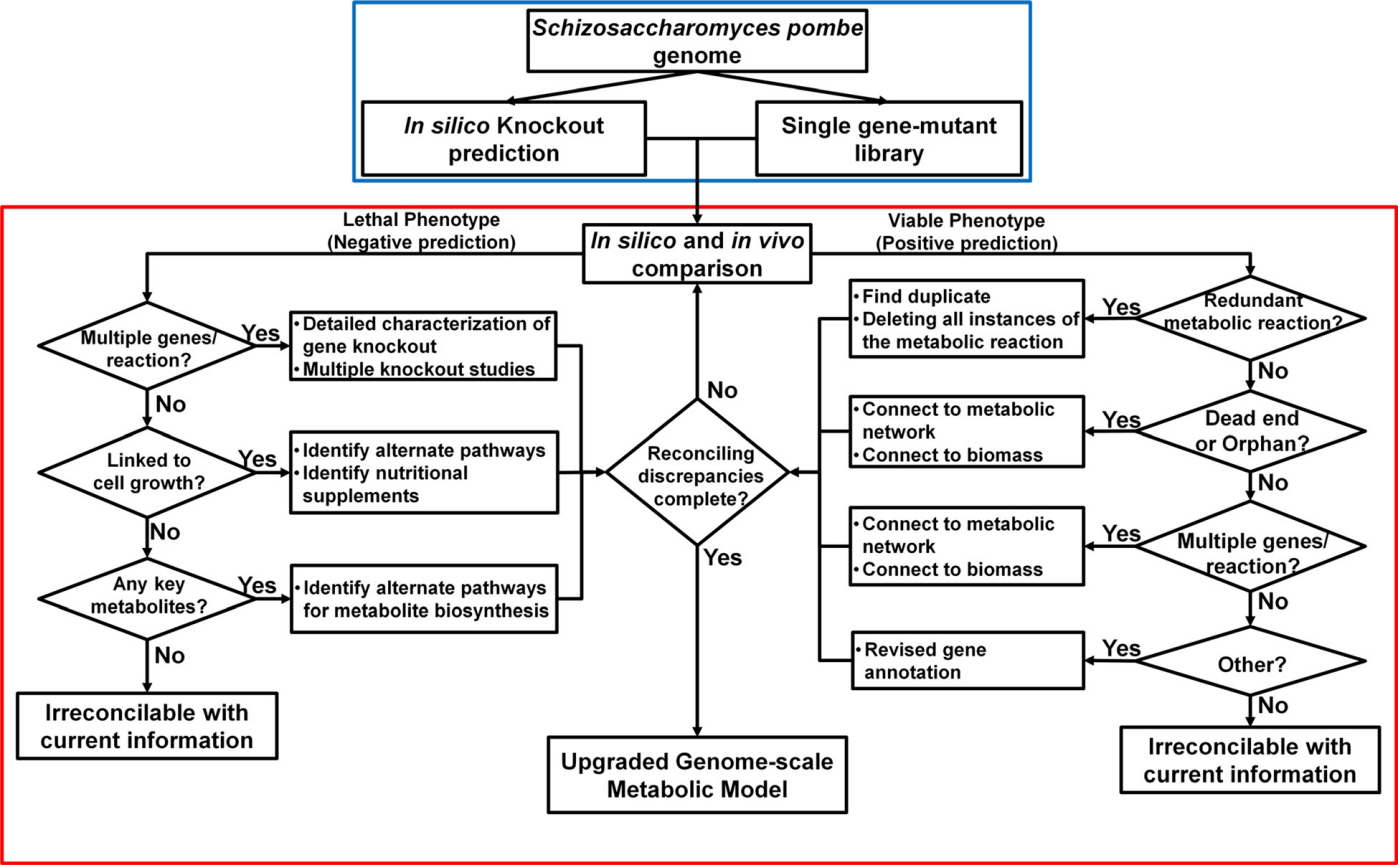
**Diagram of the overall scheme of RING (Reconciliation of*In silico/in vivo*mutaNt Growth) divided into two stages.** In the top stage (Blue Box), the reconstruction of the genome-scale metabolic model and the single-gene mutant growth simulations from the *S. pombe* genome is performed. Also in this stage is the generation of the single-gene mutant knockout library which was imported from Kim et al. [23]. In the second stage (Red Box), the analysis and reconciliation of the discrepancies between *in silico* prediction and *in vivo* observation is performed in an iterative manner. Once all possible strategies in reconciling the differences between the *in silico* predictions and *in vivo* observations are exhausted based on current information and knowledge available, the end result is an updated genome-scale metabolic model.

Here the strategy for reconciling differences between *in silico* predictions and *in vivo* observations (RING) is applied to validate and upgrade the first reconstruction of the genome-scale metabolic model of *S. pombe*, SpoMBEL1693. The ability of this newly reconstructed metabolic model to represent the metabolic physiology of this yeast was analyzed by comparing the growth phenotypes obtained by single gene knockout simulations with those experimentally observed for the single-gene knockout mutant library [23]. Using RING, the discrepancies between *in silico* predictions and *in vivo* observations were systematically and iteratively resolved. The overall scheme for the process can be seen in Figure 1.

*In silico* growth phenotypes for the deletion of every metabolic reaction were generated and the respective genes associated to each metabolic reaction were identified. These growth phenotypes were then categorized as either positive or negative (viable or lethal) with a viability threshold of 10% of the “wild-type” growth rate. Then the *in vivo* phenotypes for each gene was then retrieved from the single-gene knockout mutant library publically available [23]. Once the *in vivo* phenotypes were retrieved and compared against the *in silico* predictions, the growth phenotypes were then further categorized based on whether the predictions matched the *in vivo* observations (True or False). The false predictions were then sorted and analyzed in a step-wise manner outlined in Figure 1 until all predictions were examined.

The iterative manner, with which RING was employed, was to ensure that the changes made to SpoMBEL1693 to reconcile the discrepancies, do not alter other results and negatively affect the overall accuracy, defined as the number of correct *in silico* predictions over the total number of predictions made, of the metabolic model. By reconciling discrepancies between *in silico* prediction and *in vivo* data, the genome-scale metabolic model was able to accurately represent the metabolic characteristics of *S. pombe*. Simulations were performed under YES media conditions and the knockout results were categorized as either positive or negative, where the positive represents viable phenotype for a given knockout and the negative represents a lethal phenotype for that knockout. When compared against the published results obtained with mutant library, the results are categorized as either true/false positives or true/false negatives according to whether the prediction agrees with the *in vivo* results. True results indicate that the *in silico* predictions match with the *in vivo* results and false results indicate a discrepancy between the two. A false positive indicates that SpoMBEL1693 predicts a viable phenotype while the *in vivo* result shows a lethal phenotype (Table 1). A false negative result represents that the SpoMBEL1693 predicts a lethal phenotype while the *in vivo* result shows a viable phenotype (Table 1). Analysis of false predictions *via* RING highlights gaps in the knowledge of the metabolism of *S. pombe* and leads to improvements to the metabolic model by reconciling these differences between the *in silico* prediction and *in vivo* observations.

**Table 1:** Terms and definitions used in the analysis between *in silico* predictions and *in vivo* observations

**Term**	**Definition**	**Description**
True postive (TP)	*In silico*: viable phenotype	Results where the metabolic model predicts a viable phenotype and the actual phenotype is also viable
	*In vivo: *viable phenotype	
True negative (TN)	*In silico*: lethal phenotype *In vivo: *lethal phenotype	Results where the metabolic model predicts a lethal phenotype and the actual phenotype is also lethal
False positive (FP)	*In silico*: viable phenotype*In vivo: *lethal phenotype	Results where the metabolic model predicts a viable phenotype but the actual phenotype is lethal
False negative (FN)	*In silico*: lethal phenotype *In vivo: *viable phenotype	Results where the metabolic model predicts a lethal phenotype but the actual phenotype is viable
Overall accuracy	(TP + TN)**/**(TP + TN + FP + FN)	Percentage of correct predictions by the metabolic model
Negative prediction rate	(TN)/(TN + FN)	Percentage of negative predictions that are correctly predicted as lethal
Positive prediction rate	(TP)/(TP + FP)	Percentage of positive predictions that are correctly predicted as viable
Sensitivity	(TP)/(TP + FN)	Percentage of viable predictions correctly predicted as positive
Specificity	(TN)/(TN + FP)	Percentage of lethal predictions correctly predicted as negative

### Metabolic model characteristics

The metabolic model of *S. pombe*, SpoMBEL1693, consists of 1693 metabolic reactions, including 386 transport and exchange reactions, and 1744 metabolites. The metabolic model is divided into 8 different compartments to represent the different organelles in *S. pombe*: cytoplasm, mitochondria, nucleus, peroxisome, endoplasmic reticulum, golgi apparatus, vacuole and the extracellular environment (Additional file 1). The metabolic reactions were taken from the Kyoto Encyclopedia of Genes and Genomes [24], NCBI, and supplemented with information in the *S. pombe* gene database on GeneDB [25]. Compartmental assignment of the reactions was based on the reports in which protein localization experiments were performed [26,27]. The total gene coverage of the metabolic model is 605 genes out of 4940 protein-coding genes.

An important metabolic reaction in SpoMBEL1693 is the biomass formation reaction. This “pseudo” metabolic reaction is used to represent the synthesis of cellular biomass, or cell growth. Construction of the biomass reaction involves the accumulation of all important components necessary for biomass formation with the coefficients determined through both experimental measurements and data present in the literature. The biomass reaction is particularly important in our analysis as it is employed to indicate whether a metabolic reaction and their respective genes are essential for growth. Detailed information in the construction of the biomass reaction can be found in the methods and in Additional file 2. To validate the reconstruction of this metabolic model, the *in silico* single knockout simulations was measured against the single-gene knockout mutant library through the use of the RING strategy and will be discussed in detailed here. Furthermore, additional validation of the metabolic model was done by comparing the metabolic model’s capability in utilizing various carbon sources and production of ethanol at different dilution rates (See Additional file 3).

### Gene/reaction essentiality simulation

Gene knockout simulations were performed to evaluate the capability of the metabolic model to predict growth phenotypes of *S. pombe.* The impact of each metabolic reaction and its respective gene on the growth phenotype was investigated using the metabolic model. As a result, 198 essential metabolic reactions corresponding to 84 genes were identified (Additional file 4). Transport reactions and metabolic reactions for which no gene assignment or experimental data were available were not included in the analysis. However, duplicate metabolic reactions in different compartments were included and this accounts for the large difference in number of metabolic reactions and genes. It should be noted that the *in silico* simulation of the genome-scale metabolic model was based solely on the stoichiometry of the metabolic reactions, while the regulatory, signaling or other interactive information was not included.

Lethal genes were determined by observing the change in the *in silico* growth rate when the corresponding metabolic reaction was removed from the model, representing the deletion of its respective genes. If the cell growth rate dropped to zero or less than 10% of the original “wild-type” growth rate, the resulting phenotype was classified as lethal and the reaction and its respective genes were considered to be essential. When no change to the *in silico* growth rate was observed or remained greater than 10% of the “wild-type” growth rate, the metabolic reaction and its respective genes were determined to be non-essential, as the resulting phenotype is viable. The RING analysis was performed in an iterative manner where the metabolic model was revised based on the analysis of the comparison between the results of *in silico* knockout simulation and those experimentally observed with single-gene knockout library [23].

### Resolution and analysis of false positive predictions

**Figure 2:**
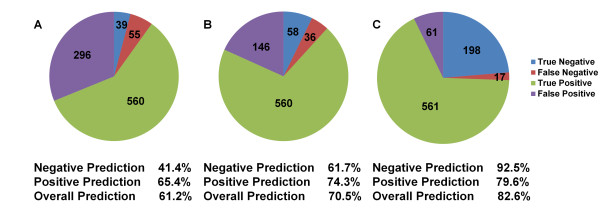
**Summary of the metabolic reaction in their categories from the results of the*in silico*single-gene mutant prediction for SpoMBEL1693.A)** Initial results and percentages of SpoMBEL1693 on predicting single-gene mutant growth phenotype. **B)** Improved rates in true predictions by SpoMBEL1693 after one iteration of RING. **C)** Final results and percentages of SpoMBEL1693 on predicting single-gene mutant growth phenotype after updating the metabolic model to resolve discrepancies between *in silico* prediction and *in vivo* data. Percentages were calculated by the number of true predictions over the total number of predictions for each group.

**Figure 3:**
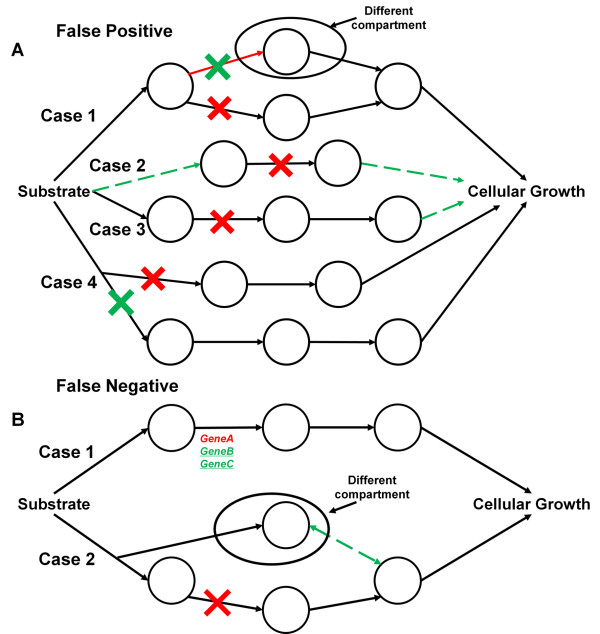
**Diagram of possible causes and solutions for false predictions.** Circles represent metabolites and arrows represent metabolic reactions **A)** false positive predictions **B)** false negative predictions. Red arrows indicate problems in the network and green arrows indicate possible solutions. Red Xs indicate knockout of the reaction and green Xs are knockouts that would reconcile the issue to achieve the correct prediction. For false negative predictions where the reaction associated with multiple genes, the gene in red indicates that the gene is knocked out or with uncertain metabolic essentiality (metabolically non-essential but essential in another capacity) and the genes in green indicate additional knockouts that can potentially resolve the false prediction.

False results indicate that information is absent or incorrect in the metabolic model resulting in a discrepancy with what is observed *in vivo*. Thus, these false results must be resolved through adding missing or correcting erroneous information such that the *in silico* predictions match the observed *in vivo* phenotypes. In this section we will examine the different cases for which false positive prediction arises and strategies to resolve these discrepancies. A false positive prediction indicates that a viable phenotype is incorrectly predicted by the metabolic model when a metabolic reaction (and by association, its corresponding gene) is deleted. Analysis of the initial positive, or viable, predictions of mutant phenotypes of SpoMBEL1693 resulted in 65.4% of the positive predictions matching the observed *in vivo* phenotypes (296 false positives and 560 true positives) (Figure 2A). Strategies in resolving these inconsistencies through RING analysis are summarized in Figure 3A and are outlined in this section. The different strategies were implemented in stages to systematically analyze the false positive predictions.

The first step in reconciling false positive predictions the identification of all duplicated or redundant metabolic reactions localized in a different compartment of the metabolic network. The presence of these redundant metabolic reactions are the result of localization data placing the respective proteins in these compartment and as a result provides an alternate route through another cellular compartment (Figure 3A Case 1). Localization data can also place an enzyme in another compartment but with no other enzymes that would balance the generation or consumption of the metabolites (orphan reaction). Knockout of this reaction would give a false positive if the gene were to be essential and the duplicate metabolic reaction in the functional compartment a true negative prediction. A total of 41 metabolic reactions fall under this category and when resolved were reclassified under the negative predictions. For instance, many of the metabolic reactions have had their respective proteins localized in the nucleus isolated from other metabolic reactions in clusters or as individuals but no complete pathways, such as the first two steps into lower glycolysis, nicotinate metabolism and pentose metabolism. To validate the essentiality of the genes, all instances of the encoding metabolic reactions were deleted simultaneously.

Metabolic reactions with false positive predictions were then checked for their connectivity to the metabolic network. Analysis of the connectivity of these metabolic reactions showed that false predictions were also correlated to dead end metabolic reactions in pathways which are not connected at the downstream end, but connected at the upstream end (dead end reactions) and non-redundant orphan metabolic reactions. The orphan metabolic reactions (Figure 3A Case 2) account for 31 metabolic reactions in SpoMBEL1693, and include metabolic reactions that charge tRNA with amino acids to be used for protein synthesis. However, tRNA compositions have already been incorporated into the biomass formation reaction, making these metabolic reactions redundant and therefore were removed from the analysis, but retained in the metabolic model.

Metabolic reactions in dead end pathways were reconciled by connecting the ends of the pathways to the metabolic network (Figure 3A Case 3). In the extreme instance where linking the metabolic pathway to the metabolic network failed to resolve the false positive prediction, the major downstream metabolite was incorporated into the biomass metabolic reaction representing cellular growth, directly linking the metabolic pathway to cellular growth. The heme biosynthetic pathway is one example of this case. Heme showed no metabolic role or function in the metabolic model, resulting in false positive results in the knockout simulation*.* However, the genes encoding for the metabolic reactions of the heme biosynthesis pathway were found to be essential for growth according to the single-gene mutant library as evidenced by the lethal phenotype displayed in knockouts of genes in heme biosynthesis. Thus, heme was incorporated into the biomass metabolic reaction with a coefficient calculated with a negligible cellular concentration to prevent any drain of cellular resources by heme biosynthesis. By incorporating heme into the biomass metabolic reaction, the biosynthesis of heme becomes linked to cellular growth. A consequence of linking heme to biomass is the inclusion of iron ions into the YES media. Sterol biosynthesis is one instance where linking the metabolic pathways to the rest of the network was sufficient for resolving false positive predictions. Gaps in the metabolic pathway of sterol biosynthesis were filled (SPBC1709.07 and SPBC16E9.05) and confirmed through GeneDB to resolve the false positive predictions. A total of 37 metabolic reactions with false positive predictions were resolved and re-categorized as true negatives.

The gene associations to metabolic reactions were then examined to reconcile false positive prediction from the knockout simulation. One instance of this case is the association of multiple metabolic reactions to a single gene (Figure 3A Case 4). Enzymes encoded by a gene have been known to participate in multiple functionalities in the metabolic network, and as a result, multiple metabolic reactions in the metabolic model are associated with the same gene. Hence, deletion of just one of the metabolic reactions does not accurately reflect the single gene knockout of the respective gene. To resolve this, all metabolic reactions associated to the target gene were deleted simultaneously. With the metabolic reactions simultaneously deleted, such false positive prediction was resolved and a lethal phenotype was predicted. Sixty-four metabolic reactions were reconciled in this manner (Figure 2B).

The remaining false positive predictions were those that could not be reconciled in RING, due to lack of the information available regarding the metabolic network*.* Sixty-two metabolic reactions with false positive predictions showed no flux in the *in silico* wild-type flux distribution, indicating that these metabolic reactions are not used for growth, despite the fact that the deletion of their corresponding genes gives a lethal phenotype *in vivo*. The absence of any flux through these 62 metabolic reactions could be attributed to the lack of regulatory information that would direct the flux through that metabolic reaction. Thirty seven metabolic reactions that showed false predictions were not reconciled with high confidence due to the simultaneous assignment of both viable and lethal genes to the metabolic reactions. Eight of the 37 metabolic reactions overlap with the previous category where the metabolic reactions exhibit no flux in SpoMBEL1693. The remaining 29 metabolic reactions are utilized and exhibit fluxes when the growth rate is maximized. However, there is no indication whether the deletion of the reaction results in a lethal phenotype or the lethal gene(s) functions in another capacity that is essential for growth, but not reflected in the metabolic network. Therefore, to resolve these cases with high confidence, detailed characterization of all the genes associated to the metabolic reaction is needed. Overall, the correct prediction rate of viable phenotype was improved to 79.6% (61 false positive and 561 true positive predictions) (Figure 2C) after RING was applied (Additional file 4).

### Resolution and analysis of false negative predictions

False negative predictions are results where the growth phenotype is predicted to be lethal, but instead is viable experimentally. Initial negative prediction rate was 41.4% (55 false negative and 39 true negative predictions) (Figure 2A). These false negative predictions were also analyzed in stages and reconciled through RING (Figure 3B).

Analysis of false negative predictions started with the examination of the genes associated to the metabolic reactions with false negative predictions. The large majority of false negative metabolic reactions were found to have multiple genes associated with the metabolic reactions (Figure 3B Case 1). Eleven of the metabolic reactions were associated with both viable and lethal genes and 25 metabolic reactions were associated with only viable genes. Reconciling the false prediction of these metabolic reactions could not be resolved due to insufficient information regarding the functional roles these genes play in the metabolic reactions. For example, in metabolic reactions associated with both lethal and viable genes, it is possible that the viable gene is a minor or non-essential contributor to the functional performance of the metabolic reaction. Also, for metabolic reactions with multiple viable genes associated, it is possible that they perform an auxiliary role to each other and can functionally replace the other when that gene is deleted. In this instance, all genes associated to the metabolic reaction would have to be deleted to confirm essentiality of the reaction.

Another instance of Case 1 is where all the genes associated with the metabolic reaction are viable; it is also uncertain if the metabolic reaction is essential to the metabolic network (true negative) or if the negative prediction is indeed a false prediction. If the metabolic reaction is truly essential to the metabolic network, then the knockout of all the genes that are associated with the metabolic reaction would give the lethal phenotype when predicted using SpoMBEL1693. Single-gene knockout mutants for these genes would not be sufficient in suppressing the metabolic reaction as it would be compensated by the presence of alternate genes that can function in place of the deleted gene. Due to the lack of information that would allow for the reconciling of these false predictions, the metabolic reactions were removed from the analysis and noted for future research.

The remaining false negative predictions were examined to determine if the metabolic reactions affected the biosynthesis of biomass components for cellular growth. In this case, an alternate metabolic reaction is needed to resolve this false prediction (Figure 3B Case 2). If a metabolic reaction is the only source of an essential metabolite (*i.e.* an essential intermediate necessary for the biosynthesis of biomass components), strategies were investigated to supply the essential metabolite from other sources within the metabolic network (*e.g.* another compartment). For example, in the cytoplasm, acetyl-CoA was produced only through the metabolic reaction represented by the enzyme Acetyl-CoA synthetase, which is a non-essential enzyme for growth based on the single-gene knockout mutant library. However, knockout simulations show that acetyl-CoA in the cytoplasm is essential for growth, a precursor to the synthesis of biomass components. Thus, an alternate pathway that can produce acetyl-CoA is needed in the cytoplasm. Alternate metabolic reactions capable of producing acetyl-CoA were found in the mitochondria. However, localization data of the metabolic enzymes in *S. pombe* does not support the presence of the corresponding metabolic reactions in the cytoplasm [27]. Thus, to allow the cytoplasm compartment access to the acetyl-CoA produced in the mitochondria, the exchange reaction for acetyl-CoA between the mitochondria and the cytoplasm was added to confirm that a viable phenotype can be attained (Figure 3B Case 2). The addition of this exchange reaction resulted in a viable phenotype and suggests the presence of an acetyl-CoA transport from the mitochondria to the cytosol. Direct transport of acetyl-CoA between the intracellular compartments is not possible due to the compound’s bulkiness and amphiphilic nature [28], therefore, the *S. pombe* genome was searched for a carnitine-acetyl-CoA shuttle that has been reported in *S. cerevisiae* (CAT2, YAT1 and YAT2). However, a search through the genome annotation and a BLAST search for the carnitine-acetyl-CoA shuttle in *S. pombe* resulted in no candidates. Due to the lack of any possible candidates as a transport protein for acetyl-CoA across the mitochondrial membrane and the improbability of a direct transport of acetyl-CoA, the inconsistency of acetyl-CoA synthetase remained unresolved. The remaining 16 metabolic reactions were unable to be reconciled due to insufficient information. After RING analysis of false negative predictions, the reconciliation between *in silico* and *in vivo* phenotypes resulted in the improvement of the correct prediction rate from 41.4% to 92.5% of the negative predictions matching the observed *in vivo* phenotypes (17 false negative predictions and 198 true negative predictions) (Figure 2C).

### Comparative analysis of the yeast metabolic models

The predictive capability of the *S. pombe* genome-scale metabolic model was compared to the predictive capability of another yeast metabolic model that has been reconstructed, *S. cerevisiae i*MM904 [17,18]. *i*MM904 was employed for similar studies in predicting the *in silico* growth phenotypes and was used as a basis for eukaryotic metabolic model’s prediction capability of mutant growth phenotypes [18]. First, the overall metabolisms of the two yeasts were examined with compartmental assignment of duplicate metabolic reactions ignored in both yeasts, with the exception of metabolic reactions where the localization of these reactions was distinctly different. One distinct difference between *S. pombe* and *S. cerevisiae* is the lack of metabolic reactions localized in the peroxisome, due to the scarcity of knowledge on peroxisome in the fission yeast, highlighting the need for additional studies into peroxisomal metabolism in *S. pombe*[18,29]. The central metabolic network between the two yeasts displayed little variability in the structure of the metabolic network, with the exception of the absence of the glyoxylate shunt in *S. pombe.*

**Figure 4:**
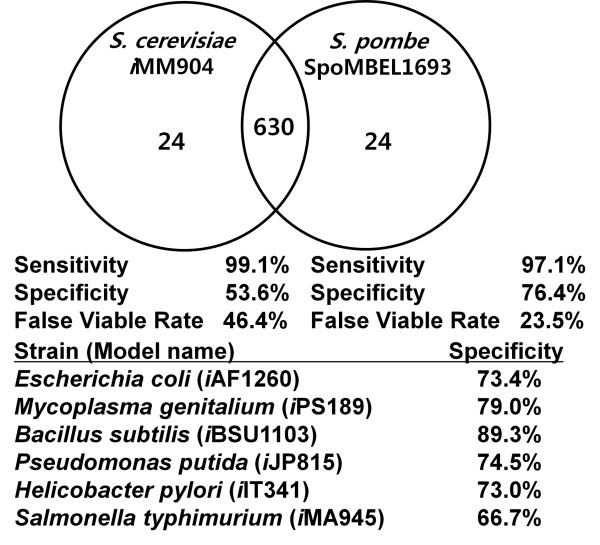
**Comparison of the performance and composition of the*S.cerevisiae* metabolic model *iMM904 and the S. pombe* metabolic model SpoMBEL1693.** The numbers in the Venn Diagram indicate the number of reactions in the metabolic models. Specificity = TN/(TN + FP), Sensitivity = TP/(TP + FN), False Viable Rate = FP/(FP + FN), TN = True Negative, TP = True Positive, FN = False Negative, FP = False Positive.

The results of the analysis of SpoMBEL1693 to predict mutant growth phenotypes were compared to those obtained with the *S. cerevisiae* metabolic model *i*MM904 [18]. In the analysis of *i*MM904, the statistical classification function, specificity and sensitivity, were employed in the analysis of the essentiality simulation to represent the proportion of negative and positive (lethal and viable) phenotypes correctly predicted as negative and positive, respectively (Table 1). In other words, specificity represents the proportion of negative phenotypes that were correctly predicted to be negative by the metabolic model (TN:TN + FP). Sensitivity is defined the same except that it looks at the proportion of positive phenotypes correctly predicted to be positive by the metabolic model (TP:TP + FN). The specificity of 53.6% and sensitivity of 99.1% were achieved using *i*MM904 [18]. For comparison, the specificity and sensitivity in predicting the phenotypes of single-gene knockout mutants using SpoMBEL1693 were calculated. A higher specificity of 76.4% and a comparable sensitivity of 97.1% were obtained with SpoMBEL1693. A false viable rate, FP/(FP + FN), or the ratio of false predictions that have been experimentally observed to be lethal, was also calculated for *i*MM904 and compared with that obtained with SpoMBEL1693. The false viable rate obtained with SpoMBEL1693 (23.5%) was lower than that (46.4%) obtained with *i*MM904 (Figure 4). The specificities of other metabolic models, for which essentiality analysis was performed, were also calculated. It was found that the specificity of SpoMBEL1693 was similar to four of the seven metabolic models (70-80%), and of the remaining three, only one had a higher specificity than SpoMBEL1693 (Figure 4). The metabolic model of the extensively studied bacterium *Escherichia coli, i*AF1260, was listed to have a specificity of 73.4%, placing the *S. pombe* metabolic model on the same level of performance with this bacterium in predicting mutant growth phenotypes.

With the *S. pombe* genome-scale metabolic model improved through RING, its metabolic capabilities were examined and compared to the metabolic capabilities of the *S. cerevisiae* genome-scale metabolic model. The maximum *in silico* mol yield of 4 different metabolites, which have been targeted in the past metabolic engineering (acetate, ethanol, lactate and succinate), was determined for each yeast using their respective genome-scale metabolic models (SpoMBEL1693 and *i*MM904). Results show a difference in maximum *in silico* yield for the metabolites acetate and lactate and no difference in the yields for ethanol and succinate (Table 2). Simulations show that *S. pombe* has a higher yield in producing lactate than *S. cerevisiae* (approximately 15% less than *S. pombe*) suggesting that *S. pombe* would be a more ideal host for producing lactate from glucose. With acetate, *S. pombe* shows a slightly lower yield than in *S. cerevisiae*, which is an advantage for *S. pombe* as acetate is commonly found as a metabolic by-product. Furthermore, the lower acetate yield may also be a reflection of the absence of acetate during the aerobic ethanol fermentation in *S. pombe*, whereas acetate was observed in *S. cerevisiae*[30]*.*

**Table 2:** **Maximum*in silico*molar yields of various metabolites**^**a**^

**Metabolites**	***Schizosaccharomyces pombe***	***Saccharomyces cerevisiae***
Acetate	2.55	2.62
Ethanol	2	2
Lactate	2	1.73
Succinate	1.5	1.5

a) Molar yields are in (mol metabolites/mol glucose).

## Discussion

Here we reported the validation and improvement of the newly reconstructed genome-scale metabolic model of a fission yeast *S. pombe*, SpoMBEL1693, presented here for the first time. The experimental results imported from the publically available single-gene knockout mutant library were utilized to improve the accuracy of SpoMBEL1693 in predicting mutant growth phenotypes. The strategy designated as RING, was employed to identify and reconcile discrepancies between the *in silico* prediction results and the experimental results. Iterative application of RING resulted in a step-wise improvement in the accuracy of the genome-scale metabolic model of this less studied yeast. The first iteration of RING resulted in improving the overall accuracy by 9% (61.2% to 70.5%). The second iteration was then performed and further increased the accuracy by another 12.2% (70.5% to 82.7%).

Previous studies have been done in reconciling differences between *in silico* prediction and *in vivo* observations of mutant phenotypes [17,18,31]. In the recent study with *i*MM904, GrowMatch was employed to resolve discrepancies between the *in silico* predictions and *in vivo* observation [18]. Here the gene-protein-reaction (GPR) relationship was employed to simulate the gene knockout. However, GrowMatch also suggested several suppression strategies that went beyond the knockout of single genes to resolve inconsistencies for a single-gene mutant phenotype [32]. With the full GPR relationship in *S. pombe* not fully characterized for most of the metabolic reactions, it was decided that a direct metabolic reaction knockout would be more suitable in simulating the mutant metabolic phenotype as opposed to the knock out the metabolic reaction through uncertain GPR relationships for metabolic reactions. Furthermore, without the constraint of a preconceived GPR relationship, information into the actual GPR relation of the genes, proteins and reactions can be illuminated.

Analysis of the false predictions has identified a number of areas for which insufficient information is available to improve the accuracy of the metabolic model in predicting growth phenotypes of single-gene knockout mutants. For instance, the case where multiple genes are associated with a single metabolic reaction was discussed. In some instances, both viable and lethal genes are associated whereas in other instances multiple viable genes are associated to a reaction that is predicted to be essential in the metabolic network. Further experimental data on these metabolic reactions and their corresponding genes will provide hints on how to resolve these issues and further improve the representation of the yeast *S. pombe* by SpoMBEL1693 or its upgraded future version.

Reconciliation of discrepancies between the *in silico* prediction results and experimental results showed that a majority of the reconciled metabolic reactions are those that were predicted to be false positives. The reconciliation of these false positive predictions was achieved through the linking of the metabolic pathways to cellular growth. This contributed to the improved accuracy in negative predictions by increasing the number of true negatives. Literature evidence supporting these modifications is lacking, and therefore are potential points of interest for further studies and characterization into the metabolism of *S. pombe.* Of the remaining false positives, many of the metabolic reactions displayed no flux in the metabolic model. This indicates the lack of specific characterization on the role of the metabolic reaction in the metabolism of the yeast. As many of these metabolic reactions are found in nucleotide metabolism or secondary metabolic pathways, it is likely that the annotation of the genes for these enzymes is incomplete. Included in the false positive predictions were the results for which no experimental data or literature evidence, were available, and so whether they are truly false positive prediction or true positive predictions was undeterminable. Thus, they were included as the false predictions to highlight the need for experimental data for these genes.

Reconciliation of false negative predictions required a different approach from reconciling false positive predictions. Because the pathways were important for the synthesis of components used in the generation of biomass for cellular growth, alternate pathways would be required to bypass the deleted metabolic reaction and allow cellular growth. However, localization data for the enzymes in *S. pombe* prevents the addition of metabolic reactions into compartments which the respective enzymes have not been localized in. Thus, these alternate pathways that would allow cellular growth were made accessible through exchange reactions between the different compartments. This is shown in the case with Acetyl-CoA synthetase, where alternate pathways that synthesize acetyl-CoA in the mitochondria were made available to the cytoplasm through the exchange reaction for acetyl-CoA between the two compartments. However, due to the absence of any known acetyl-CoA shuttle system, the discrepancy could not be resolve with a high level of confidence. While this discrepancy remained unresolved, it did manage to deduce the presence of an uncharacterized transporter for acetyl-CoA across the mitochondrial membrane. Furthermore, in comparison with the *S. cerevisiae* metabolic network, it was found that Acetyl-CoA synthetase is also found to be essential, but *S. cerevisiae* has two genes associate to the reaction: one essential and one non-essential gene. As *S. cerevisiae* possesses the carnitine-acetyl-CoA shuttle (absent in the metabolic model of *S. cerevisiae*), this suggests a more in-depth study into the essential gene associated to Acetyl-CoA synthetase in *S. cerevisiae*. Other false negative predictions require additional information beyond what the single-gene knockout mutant library can provide. For instance, an essential metabolic reaction can be associated to two genes where one gene may compensate for the knockout of the other and would require a double-gene knockout to determine the validity of the predicted *in silico* phenotype.

Comparison of the RING analysis results on reconciling single-gene mutant phenotypes in *S. pombe* with the study of reconciling single-gene mutants of *S. cerevisiae* using GrowMatch, demonstrates the advantages of the flexibility RING brings to the process. It should be noted that while both approaches examined the problem of reconciling *in silico* predictions with *in vivo* observations at both the gene and reaction levels, the simulations done at the reaction level (RING) and simulations done at the gene level (GrowMatch), may not be directly comparable. Yet, using the RING strategy, we were able to resolve a higher proportion of false positive predictions as demonstrated by the higher specificity (*i.e.* false positives are lethal phenotypes predicted to be viable). Also the proportion of viable phenotypes accurately predicted to be viable (sensitivity) in *S. pombe* is comparable, though slightly lower than the study with *S. cerevisiae.* Considering that the volume of knowledge on *S. cerevisiae* is far greater than that on *S. pombe*, the results attain with RING is notable.

## Conclusions

In this paper, we reported the reconstruction of the genome-scale metabolic model of the fission yeast *S. pombe* SpoMBEL1693 and the strategy for refining the model’s ability to predict the growth phenotypes of the single-gene knockout mutants. An iterative process called RING was employed in reconciling false *in silico* predictions with experimentally observed phenotypes to improve the accuracy of the metabolic model by 21.5%. Despite the huge increase in accuracy of the metabolic model in predicting single-gene mutant phenotypes, unresolved inconsistencies between *in silico* predictions and *in vivo* observations highlight the gaps in our knowledge regarding the metabolism of *S. pombe*. The lack of literature evidence supporting the reconciled changes to the metabolic model based on our analysis highlights the gaps in our knowledge. Detailed characterization of GPR relationships specific to *S. pombe* would increase the confidence level in resolving the inconsistencies. Furthermore double-gene mutant phenotypes would also aid in revolving many of the inconsistencies where gene duplicates exist. The SpoMBEL1693 metabolic model reconstructed and validated here is a first step towards enhancing our understanding of eukaryotic metabolism.

## Methods

### Model reconstruction

The initial reconstruction of the metabolic model was performed using the set of biochemical reactions annotated from the genome and presented in the Kyoto Encyclopedia of Genes and Genomes [24], NCBI, and the *S. pombe* gene database on GeneDB [25]. Compartment assignment was also taken from previous reports where protein localization was determined experimentally [26,27]. Transport reactions were brought in from the TransportDB [33] (See Additional file 5).

From the KEGG databases, the genomic information of *S. pombe* was downloaded and the gene information and the E.C. numbers assigned to the enzymes encoded by the respective genes were extracted. All metabolic reactions were collected and transferred into the metabolic model reconstruction (See Additional file 1). Water and hydroxyl ions were not balanced by assuming that there are other non-enzymatic functions in the cell that uses these molecules and therefore do not need to be balanced in the set composed of enzymatic reactions. Once the set of biochemical reactions has been collected, the list is curated for any inconsistencies or gaps in the network.

### Strains and culture conditions

Cultures of the fission yeast were performed to obtain data utilized in the reconstruction of the genome-scale metabolic model. *S. pombe* was obtained from the DSMZ (DSM-70576) and was cultured in yeast nitrogen base media without amino acids to create a stock of the yeast stored at −80°C until thawed for fermentation and wet experiments performed for the validation of the model. *S. pombe* was cultured at 30°C.

Batch cultures were carried out as follows. Seed cultures were prepared by transferring 500 μL of 10 mL overnight cultures prepared in yeast nitrogen based media without amino acids plus 10 g/L of glucose into 250 mL Erlenmeyer flask containing 100 mL of the same medium and incubated in a shaker at 30°C. Cultured cells used to inoculate the fermenter containing 2 L of yeast nitrogen based media without amino acids medium containing 20 g/L glucose at 30°C. Batch culture was carried out in a 6.6 L Bioflo 3000 fermenter (New Brunswick Scientific Co., Edison, NJ). The agitation speed was initially set at 200 rpm and was increased accordingly using automatic controlling to maintain a dissolved oxygen concentration (DOC) at 40% of air saturation or greater. The pH was adjusted at 6.00 ± 0.1 using 28% (v/v) ammonia solution. Foaming was controlled by the addition of Antifoam 289 (Sigma, St. Louis, MO). Aeration was done at a flow rate of 0.25 vvm during the whole period of fermentation.

Samples for the measurement of amino acid composition, to be used for cellular growth, was taken from the batch cultures during the exponential phase. Nine milliliters of the culture was centrifuged and the supernatant was removed, leaving the cell pellet, which was used to analyze the amino acid composition (See analytical procedures).

### Analytical procedures

Cell growth was monitored by measuring the absorbance at 600 nm (OD_600_) with an Ultrospec3000 spectrophotometer (Amersham Biosciences, Uppsala, Sweden). Cell concentration defined as gram dry cell weight (gDCW) per liter was determined by using the correlation found in literature relating the OD_600_ to dry weight (1 OD_600_ = 0.62 gDCW/L). The concentrations of glucose and by-products in the media were determined by high-performance liquid chromatography (Varian ProStar 210, Palo Alto, CA) equipped with UV/VIS (Varian ProStar 320, Palo Alto, CA) and RI (Shodex RI-71, Tokyo, Japan) detectors. A MetaCarb 87 H column (300 × 7.8 mm, Varian) was isocratically eluted with 0.01 N H_2_SO_4_ at 60°C and a flow rate of 0.6 mL/min.

>Composition of the amino acids and fatty acids in *S. pombe* was determined from samples obtained from batch fermentations during the exponential growth phase in the yeast nitrogen base media without amino acids, containing 20 g/L glucose as a carbon source. Amino acid compositions were quantified using a Waters HPLC system (Waters Corporation, Milford, MA) which consists of two 510 HPLC pumps, a gradient controller, 717 automatic sampler, 996 photodiode array detector, and a Millennium 32 chromatography manager together with Waters pico-tag column (3.9 x 300 mm). Absorbance at 254 nm was measured. Other components were adopted from the literature or assumed (See Additional file 2).

### *In silico* flux analysis

For the analysis of the genome-scale metabolic model, *in silico* flux analysis was used where the internal metabolites were first balanced under the assumption of pseudo-steady state [34]. This resulted in a stoichiometric model S_ij_·v_j_ = 0, in which S_ij_ is a stoichiometric coefficient of a metabolite i in the jth reaction and v_j_ is the flux of the jth reaction given in mmol/gDCW/h. Linear programming (LP), subject to the constraints pertaining to mass conservation, reaction thermodynamics, and capacity, was carried out to determine the fluxes [35]. These constraints were presented in the forms of upper and lower bounds for the fluxes (v_j,min_ ≤ v_j_ ≤ v_j,max_) for each reaction j, and used together with an objective function Z, usually the growth rate [14,36].

Gene/reaction essentiality and mutant growth phenotype simulations were performed in GAMS: Integrate Development Environment using the CPLEX solver. Reaction knockout was simulated by constraining each flux to zero, while the objective function was set to maximize cellular growth. If the resulting cellular growth, or biomass formation, was less than 10% of the “wild-type” value while the flux of the metabolic reaction was constrained to zero, then the deletion of the corresponding gene was considered to be lethal and the metabolic reaction to be essential. If no change to the cellular growth was observed or biomass formation was greater than 10% of the “wild-type” when the metabolic reaction was constrained to zero, then the resulting growth phenotype was considered to be viable and the metabolic reaction to be non-essential. The media YES was used in the *in silico* simulations to mimic the growth conditions which the single-gene mutant library was conducted in and where nutrients found in yeast extract and adenine, histidine, leucine, uracil, and lysine [23] were unconstrained and glucose uptake rate was set to the experimentally determined value of 4.19 mmol glucose/gDCW/h. Additional compounds, such as iron, were also included to ensure cell growth rate.

## Misc

Seung Bum Sohn and Tae Yong Kim contributed equally to this work

## Competing interests

The authors declare that they have no competing interests.

## Author contributions

SBS, TYK, JHL, and SYL participated in the design of this study and editing of the manuscript. SBS, TYK, JHL, and SYL performed the simulations, analysis and drafting of the manuscript. All authors read and approved the final manuscript

## Supplementary Material

Additional file 1: List of metabolic reactions and metabolite abbreviations used in SpoMBEL1693. Click here for file

Additional file 2: SpoMBEL1693 Characteristics and biomass composition. Click here for file

Additional file 3: Additional validation studies for SpoMBEL1693 - Carbon source utilization and Flux Variability Analysis of ethanol production capacity. Click here for file

Additional file 4: List of False positive and False negative predictions from single knockout simulation using SpoMBEL1693. Click here for file

Additional file 5: SpoMBEL1693 in SBML format. Click here for file

## References

[B1] Park JH Lee KH Kim TY Lee SY Metabolic engineering of Escherichia coli for the production of L-valine based on transcriptome analysis and in silico gene knockout simulation Proc Natl Acad Sci U S A 2007 104 7797 7802 10.1073/pnas.0702609104 17463081 PMC1857225

[B2] Mo ML Palsson BO Understanding human metabolic physiology: a genome-to-systems approach Trends Biotechnol 2009 27 37 44 10.1016/j.tibtech.2008.09.007 19010556

[B3] Lewis NE Cho BK Knight EM Palsson BØ Gene expression profiling and the use of genome-scale in silico models of Escherichia coli for analysis: providing context for content J Bacteriol 2009 191 3437 3444 10.1128/JB.00034-09 19363119 PMC2681886

[B4] Kim TY Kim HU Lee SY Metabolite-centric approaches for the discovery of antibacterials using genome-scale metabolic networks Metab Eng 2010 12 105 111 10.1016/j.ymben.2009.05.004 19481614

[B5] Jeong KJ Jang SH Velmurugan N Recombinant antibodies: engineering and production in yeast and bacterial hosts Biotechnol J 2011 6 16 27 10.1002/biot.201000381 21170983

[B6] Mo ML Palsson BO Herrgard MJ Connecting extracellular metabolomic measurements to intracellular flux states in yeast BMC Syst Biol 2009 3 37 10.1186/1752-0509-3-37 19321003 PMC2679711

[B7] Lewis NE Schramm G Bordbar A Schellenberger J Andersen MP Cheng JK Patel N Yee A Lewis RA Eils R Large-scale in silico modeling of metabolic interactions between cell types in the human brain Nat Biotechnol 2010 28 1279 1285 10.1038/nbt.1711 21102456 PMC3140076

[B8] Duarte NC Becker SA Jamshidi N Thiele I Mo ML Vo TD Srivas R Palsson BØ Global reconstruction of the human metabolic network based on genomic and bibliomic data Proc Natl Acad Sci U S A 2007 104 1777 1782 10.1073/pnas.0610772104 17267599 PMC1794290

[B9] Ma H Sorokin A Mazein A Selkov A Selkov E Demin O Goryanin I The Edinburgh human metabolic network reconstruction and its functional analysis Mol Syst Biol 2007 3 135 17882155 10.1038/msb4100177 PMC2013923

[B10] Selvarasu S Karimi IA Ghim GH Lee DY Genome-scale modeling and in silico analysis of mouse cell metabolic network Mol Biosyst 2010 6 142 151 10.1039/b908412f 20024077

[B11] Doyle M MacRae J De Souza D Saunders E McConville M Liki V LeishCyc: a biochemical pathways database for Leishmania major BMC Syst Biol 2009 3 57 10.1186/1752-0509-3-57 19497128 PMC2700086

[B12] David H Ozcelik IS Hofmann G Nielsen J Analysis of Aspergillus nidulans metabolism at the genome-scale BMC Genomics 2008 9 163 10.1186/1471-2164-9-163 18405346 PMC2386489

[B13] Andersen MR Nielsen ML Nielsen J Metabolic model integration of the bibliome, genome, metabolome and reactome of Aspergillus niger Mol Syst Biol 2008 4 178 18364712 10.1038/msb.2008.12 PMC2290933

[B14] Duarte NC Herrgard MJ Palsson BO Reconstruction and validation of Saccharomyces cerevisiae iND750, a fully compartmentalized genome-scale metabolic model Genome Res 2004 14 1298 1309 10.1101/gr.2250904 15197165 PMC442145

[B15] Sohn SB Graf AB Kim TY Gasser B Maurer M Ferrer P Mattanovich D Lee SY Genome-scale metabolic model of methylotrophic yeast Pichia pastoris and its use for in silico analysis of heterologous protein production Biotechnol J 2010 5 705 715 10.1002/biot.201000078 20503221

[B16] Thiele I Palsson BØ A protocol for generating a high-quality genome-scale metabolic reconstruction Nat Protoc 2010 5 93 121 20057383 10.1038/nprot.2009.203 PMC3125167

[B17] Forster J Famili I Palsson BO Nielsen J Large-scale evaluation of in silico gene deletions in Saccharomyces cerevisiae OMICS 2003 7 193 202 10.1089/153623103322246584 14506848

[B18] Zomorrodi A Maranas C Improving the iMM904 S. cerevisiae metabolic model using essentiality and synthetic lethality data BMC Syst Biol 2010 4 178 10.1186/1752-0509-4-178 21190580 PMC3023687

[B19] Park JM Kim TY Lee SY Prediction of metabolic fluxes by incorporating genomic context and flux-converging pattern analyses Proc Natl Acad Sci U S A 2010 107 14931 14936 10.1073/pnas.1003740107 20679215 PMC2930451

[B20] Wood V Gwilliam R Rajandream MA Lyne M Lyne R Stewart A Sgouros J Peat N Hayles J Baker S The genome sequence of Schizosaccharomyces pombe Nature 2002 415 871 880 10.1038/nature724 11859360

[B21] Dragan CA Peters FT Bour P Schwaninger AE Schaan SM Neunzig I Widjaja M Zapp J Kraemer T Maurer HH Bureik M Convenient gram-scale metabolite synthesis by engineered fission yeast strains expressing functional human P450 systems Appl Biochem Biotechnol 2011 163 965 980 10.1007/s12010-010-9100-3 20927605

[B22] Takegawa K Tohda H Sasaki M Idiris A Ohashi T Mukaiyama H Giga-Hama Y Kumagai H Production of heterologous proteins using the fission-yeast (Schizosaccharomyces pombe) expression system Biotechnol Appl Biochem 2009 53 227 235 10.1042/BA20090048 19531030

[B23] Kim DU Hayles J Kim D Wood V Park HO Won M Yoo HS Duhig T Nam M Palmer G Analysis of a genome-wide set of gene deletions in the fission yeast Schizosaccharomyces pombe Nat Biotechnol 2010 28 617 623 10.1038/nbt.1628 20473289 PMC3962850

[B24] Kanehisa M Goto S Hattori M Aoki-Kinoshita KF Itoh M Kawashima S Katayama T Araki M Hirakawa M From genomics to chemical genomics: new developments in KEGG Nucleic Acids Res 2006 34 D354 D357 10.1093/nar/gkj102 16381885 PMC1347464

[B25] Hertz-Fowler C Peacock CS Wood V Aslett M Kerhornou A Mooney P Tivey A Berriman M Hall N Rutherford K GeneDB: a resource for prokaryotic and eukaryotic organisms Nucleic Acids Res 2004 32 D339 D343 10.1093/nar/gkh007 14681429 PMC308742

[B26] Aslett M Wood V Gene Ontology annotation status of the fission yeast genome: preliminary coverage approaches 100 % Yeast 2006 23 913 919 10.1002/yea.1420 17072883

[B27] Matsuyama A Arai R Yashiroda Y Shirai A Kamata A Sekido S Kobayashi Y Hashimoto A Hamamoto M Hiraoka Y ORFeome cloning and global analysis of protein localization in the fission yeast Schizosaccharomyces pombe Nat Biotechnol 2006 24 841 847 10.1038/nbt1222 16823372

[B28] Strijbis K Distel B Intracellular acetyl unit transport in fungal carbon metabolism Eukaryot Cell 2010 9 1809 1815 10.1128/EC.00172-10 20889721 PMC3008284

[B29] Jourdain I Sontam D Johnson C Dillies C Hyams JS Dynamin-dependent biogenesis, cell cycle regulation and mitochondrial association of peroxisomes in fission yeast Traffic 2008 9 353 365 10.1111/j.1600-0854.2007.00685.x 18088324

[B30] de Jong-Gubbels P van Dijken JP Pronk JT Metabolic fluxes in chemostat cultures of Schizosaccharomyces pombe grown on mixtures of glucose and ethanol Microbiology 1996 142 Pt 6 1399 1407 8704980 10.1099/13500872-142-6-1399

[B31] Feist AM Henry CS Reed JL Krummenacker M Joyce AR Karp PD Broadbelt LJ Hatzimanikatis V Palsson BO A genome-scale metabolic reconstruction for Escherichia coli K-12 MG1655 that accounts for 1260 ORFs and thermodynamic information Mol Syst Biol 2007 3 121 17593909 10.1038/msb4100155 PMC1911197

[B32] Kumar VS Maranas CD GrowMatch: an automated method for reconciling in silico/in vivo growth predictions PLoS Comput Biol 2009 5 e1000308 10.1371/journal.pcbi.1000308 19282964 PMC2645679

[B33] Ren Q Chen K Paulsen IT TransportDB: a comprehensive database resource for cytoplasmic membrane transport systems and outer membrane channels Nucleic Acids Res 2007 35 D274 D279 10.1093/nar/gkl925 17135193 PMC1747178

[B34] Gombert AK Nielsen J Mathematical modelling of metabolism Curr Opin Biotechnol 2000 11 180 186 10.1016/S0958-1669(00)00079-3 10753761

[B35] Varma A Palsson BO Stoichiometric flux balance models quantitatively predict growth and metabolic by-product secretion in wild-type Escherichia coli W3110 Appl Environ Microbiol 1994 60 3724 3731 7986045 10.1128/aem.60.10.3724-3731.1994 PMC201879

[B36] Kim TY Kim HU Park JM Song H Kim JS Lee SY Genome-scale analysis of Mannheimia succiniciproducens metabolism Biotechnol Bioeng 2007 97 657 671 10.1002/bit.21433 17405177

