# Supplementary material for: Genome-scale metabolic model of the fission yeast Schizosaccharomyces pombe and the reconciliation of in silico/in vivo mutant growth
Source: BMC Syst Biol. 2012 Jul 5;6:49. doi: 10.1186/1752-0509-6-49 (PMC3390277; doi:10.1186/1752-0509-6-49)
Supplement: Additional file 1: — List of metabolic reactions and metabolite abbreviations used in SpoMBEL1693. [file 1752-0509-6-49-S1.pdf]

Supporting Information 1A: List of metabolic reactions used in SpoMBEL1693

REACTION NAME	COMPARTMENT	METABOLIC REACTION	SUBSYSTEM	Gene(s) S. pombe	EC
alanine racemase	Cytosol	ALA <-> dALA	Alanine and Aspartate Metabolism	SPBC359.02	5.1.1.1
alanine aminotransferase (predicted)	Cytosol	AKG + ALA <-> GLU + PYR	Alanine and Aspartate Metabolism	SPBC582.08	2.6.1.2
L-alanine transaminase	Mitochondria	AKG + ALA <-> GLU + PYR	Alanine and Aspartate Metabolism	SPBC582.08	2.6.1.2
aspartate-semialdehyde dehydrogenase	Cytosol	4pasp + H + NADPH -> aspSA + NADP + Pi	Alanine and Aspartate Metabolism	SPCC1827.06c	1.2.1.11
asparagine synthase (glutamine-hydrolysing)	Cytosol	ASP + ATP + GLN + H2O -> AMP + ASN + GLU + H + PPi	Alanine and Aspartate Metabolism	SPBC119.10	6.3.5.4
aspartate kinase (predicted)	Cytosol	ASP + ATP -> 4pasp + ADP	Alanine and Aspartate Metabolism	SPBC19F5.04	2.7.2.4
aspartate transaminase (predicted)	Cytosol	AKG + ASP <-> GLU + OAA	Alanine and Aspartate Metabolism	SPAC10F6.13c	2.6.1.1
aspartate transaminase (predicted)	Mitochondria	AKG + ASP <-> GLU + OAA	Alanine and Aspartate Metabolism	SPBC725.01	2.6.1.1
aspartate transaminase (predicted)	Nucleus	AKG + ASP <-> GLU + OAA	Alanine and Aspartate Metabolism	SPAC10F6.13c	2.6.1.1
homocysteine S-methyltransferase	Cytosol	SAM + HCYS -> SAH + H + MET	Alanine and Aspartate Metabolism	SPAC57A7.07c	2.1.1.10
1,4-alpha-D-Glucan glucanohydrolase	Cytosol	GLYCogen + H2O -> dext	Alternate Carbon Metabolism	SPAC23D3.14c	3.2.1.1
Dextrin 6-alpha-D-glucanohydrolase	Cytosol	dext + H2O -> GLC	Alternate Carbon Metabolism	SPBC14C8.05c	3.2.1.3
L-lactate dehydrogenase	Cytosol	LAC + NAD <-> PYR + NADH + H	Alternate Carbon Metabolism	SPAC186.08c	1.1.1.27
L-lactate dehydrogenase (mitochondria??)	Mitochondria	LAC + NAD <-> PYR + NADH + H	Alternate Carbon Metabolism	SPAC186.08c	1.1.1.27
beta-fructofuranosidase-like protein	Cytosol	H2O + sucr -> fru + GLC	Alternate Carbon Metabolism	SPAPB24D3.10c	3.2.1.20
beta-fructofuranosidase-like protein	Extracellular	H2O + sucr -> fru + GLC	Alternate Carbon Metabolism	SPAPB24D3.10c	3.2.1.20
glucan 1,4-alpha-glucosidase	Golgi apparatus	GLYCogen + H2O -> GLC	Alternate Carbon Metabolism	SPBC14C8.05c	3.2.1.3
glucan 1,4-alpha-glucosidase, vacuole	Endoplasmic Reticulum	GLYCogen + H2O -> GLC	Alternate Carbon Metabolism	SPBC14C8.05c	3.2.1.3
lactoylglutathione lyase	Nucleus	GTHrd + mtHgxl -> lgt-S	Alternate Carbon Metabolism	SPBC12C2.12c	
hydroxyacylglutathione hydrolase (predicted)	Nucleus	H2O + lgt-S -> GTHrd + H + dLAC	Alternate Carbon Metabolism	SPCC13B11.03c	3.1.2.6
1,3-beta-glucan synthase	Cytosol	UDPg -> 13BDGLCn + H + UDP	Alternate Carbon Metabolism	SPAC24C9.07c<br>SPBC19G7.05c<br>SPCC1840.02c<br>SPAC19B12.03	2.4.1.34
1,6-beta-glucan synthase	Cytosol	UDPg -> 16BDGLCn + H + UDP	Alternate Carbon Metabolism	SPAC23H3.11c<br>SPAC17G6.11c	
13-alpha-glucan synthase	Cytosol	UDPg -> 13ADGLCn + H + UDP	Alternate Carbon Metabolism		
Aminoacetone:oxygen oxidoreductase(deaminating)(flavin-containing)	Cytosol	aact + H2O + O2 -> H2O2 + mtHgxl + NH4	Alternate Carbon Metabolism		
(R,R)-butanediol dehydrogenase	Cytosol	btd-RR + NAD <-> actn-R + H + NADH	Alternate Carbon Metabolism		1.1.1.4
hydroxyacylglutathione hydrolase (predicted)	Cytosol	H2O + lgt-S -> GTHrd + H + dLAC	Alternate Carbon Metabolism	SPAC824.07	3.1.2.6
lactaldehyde dehydrogenase	Cytosol	H2O + lald-L + NAD -> 2 H + LAC + NADH	Alternate Carbon Metabolism		
lactoylglutathione lyase	Cytosol	GTHrd + mtHgxl -> lgt-S	Alternate Carbon Metabolism	SPBC12C2.12c	
methylglyoxal synthase	Cytosol	DHAP -> mtHgxl + Pi	Alternate Carbon Metabolism		
D-sorbitol dehydrogenase (D-fructose producing)	Cytosol	NAD + sbt-D -> fru + H + NADH	Alternate Carbon Metabolism	SPBC1773.05c	1.1.1.14
L-sorbitol dehydrogenase (L-sorbose-producing)	Cytosol	NAD + sbt-L -> H + NADH + srb-L	Alternate Carbon Metabolism	SPBC1773.05c	1.1.1.14
trehalose-phosphatase	Cytosol	H2O + TRE6p -> Pi + TRE	Alternate Carbon Metabolism	SPAC328.03+SPAC19G12.15c+SPAC22F8.05<br>SPAC328.03+SPAC19G12.15c+SPACUNK4.16c	2.4.1.15
alpha,alpha-trehalose-phosphate synthase (UDP-forming)	Cytosol	G6P + UDPg -> H + TRE6p + UDP	Alternate Carbon Metabolism	SPAC328.03+SPAC19G12.15c+SPAC22F8.05<br>SPAC328.03+SPAC19G12.15c+SPACUNK4.16c	2.4.1.15
alpha,alpha-trehalase	Cytosol	H2O + TRE -> 2 GLC	Alternate Carbon Metabolism	SPBC660.07	3.2.1.28
alpha,alpha-trehalase (vacuolar)	Vacuole	H2O + TRE -> 2 GLC	Alternate Carbon Metabolism		3.2.1.28
2-Hydroxybutyrate:NAD+ oxidoreductase	Cytosol	2Hb + NAD -> 2obut + NADH + H	Anaplerotic reactions	SPAC186.08c	1.1.1.27
1-aminocyclopropane-1-carboxylate aminohydrolase (isomerizing)	Cytosol	ACP1C + H2O <-> 2obut + NH4	Anaplerotic reactions	SPAC922.03	3.5.99.7
malic enzyme (NAD),	Cytosol	MAL + NAD -> CO2 + NADH + PYR	Anaplerotic reactions	SPCC794.12c	1.1.1.38
malic enzyme (NADP),	Cytosol	MAL + NADP -> CO2 + NADPH + PYR	Anaplerotic reactions	SPCC794.12c	1.1.1.38
fructose-bisphosphatase	Cytosol	FDP + H2O -> F6P + Pi	Anaplerotic reactions	SPBC1198.14c	3.1.3.11
Isocitrate lyase	Cytosol	ICIT -> GLX + SUCC	Anaplerotic reactions	SPBC1683.11c	4.1.3.1
methylisocitrate lyase	Mitochondria	MICIT -> PYR + SUCC	Anaplerotic reactions	SPBC1683.11c	4.1.3.1
2-methylcitrate synthase	Mitochondria	H2O + OAA + ppCoA -> 2MCIT + CoA + H	Anaplerotic reactions	SPAC6C3.04	2.3.3.1
malic enzyme (NAD)	Mitochondria	MAL + NAD -> CO2 + NADH + PYR	Anaplerotic reactions	SPCC794.12c	1.1.1.38
malic enzyme (NADP)	Mitochondria	MAL + NADP -> CO2 + NADPH + PYR	Anaplerotic reactions	SPCC794.12c	1.1.1.38
pyruvate carboxylase	Cytosol	ATP + HCO3 + PYR -> ADP + H + OAA + Pi	Anaplerotic reactions	SPBC17G9.11c	6.4.1.1
Estrone 3-sulfate sulfohydrolase	Cytosol	e3s + H2O -> est + SO4	Androgen and Estrogen Metabolism	SPBPB10D8.02c	3.1.6.1
Estrone 3-sulfate sulfohydrolase	Nucleus	e3s + H2O -> est + SO4	Androgen and Estrogen Metabolism	SPBPB10D8.02c	3.1.6.1
D-arabinono-1,4-lactone oxidase	Mitochondria	Dara14lac + O2 <-> ertascb-D + H2O2	Arabinose Metabolism	SPAPB1A10.12c	
D-arabinose 1-dehydrogenase (NADP)	Cytosol	arab-D + NADP -> Dara14lac + H + NADPH	Arabinose Metabolism		
ornithine cyclodeaminase	Cytosol	ORN <-> PRO + NH4	Arginine and Proline Metabolism	SPAP11E10.01	4.3.1.12
ornithine carbamoyltransferase	Mitochondria	cbp +ORN -> CITR + H + Pi	Arginine and Proline Metabolism	SPAC4G9.10	2.1.3.3
deoxyhypusine synthaseic/nucleus		H2O[n] + Q6[m] + spmd[n] -> 13dAMPp[n] + 4abutn[n] + Q6H2[m]	Arginine and Proline Metabolism	SPBC1271.04c	2.5.1.46
acteylornithine transaminase	Nucleus	acg5SA + GLU -> acorn + AKG	Arginine and Proline Metabolism	SPCC777.09c	2.6.1.11
ornithine transaminase	Nucleus	AKG +ORN -> GLU + GLU5SA	Arginine and Proline Metabolism	SPBC21C3.08c	2.6.1.13
spermidine synthase	Nucleus	ametam + ptrc -> 5mta + H + spmd	Arginine and Proline Metabolism	SPBC12C2.07c	2.5.1.16
arginase	Nucleus	ARG + H2O ->ORN + urea	Arginine and Proline Metabolism	SPBP26C9.02c<br>SPAC3H1.07	3.5.3.1
glutamate-5-semialdehyde dehydrogenase	Nucleus	GLU5p + H + NADPH -> GLU5SA + NADP + Pi	Arginine and Proline Metabolism	SPAC821.11	1.2.1.41
Ornithine Decarboxylase	Nucleus	H +ORN -> CO2 + ptrc	Arginine and Proline Metabolism	SPAC144.04c	4.1.1.17
L-4-hydroxyglutamate semialdehyde dehydrogenase	Mitochondria	4HGLUSA + H2O + NAD <-> e4HGLU + 2 H + NADH	Arginine and Proline Metabolism	SPBC24C6.04	
acetylglutamate kinase	Mitochondria	acGLU + ATP -> acg5p + ADP	Arginine and Proline Metabolism	SPAC4G9.09c	1.2.1.38, 2.7.2.8
N-acteylglutamate synthase (predicted)	Mitochondria	ACCoA + GLU -> acGLU + CoA + H	Arginine and Proline Metabolism	SPBC1271.14	2.3.1.35, 2.3.1.1
acteylornithine transaminase	Mitochondria	acg5SA + GLU -> acorn + AKG	Arginine and Proline Metabolism	SPCC777.09c	2.6.1.11
N-acetyl-g-glutamyl-phosphate reductase	Mitochondria	acg5p + H + NADPH -> acg5SA + NADP + Pi	Arginine and Proline Metabolism	SPAC4G9.09c	1.2.1.38, 2.7.2.8
amidase	Cytosol	4gudbd + H2O -> 4gudbutn + NH4	Arginine and Proline Metabolism	SPBPB8B6.03	3.5.1.4
arginase	Cytosol	ARG + H2O -> ORN + urea	Arginine and Proline Metabolism	SPBP26C9.02c<br>SPAC3H1.07	3.5.3.1
argininosuccinate lyase	Cytosol	argsuc <-> ARG + FUM	Arginine and Proline Metabolism	SPBC1539.03c<br>SPBC1773.14	4.3.2.1
argininosuccinate synthase	Cytosol	ASP + ATP + CITR <-> AMP + argsuc + H + PPi	Arginine and Proline Metabolism	SPBC428.05c	6.3.4.5

carbamoyl-phosphate synthase (glutamine-hydrolysing)	Cytosol	2 ATP + GLN + H2O + HCO3 -> 2 ADP + cbp + GLU + 2 H + Pi	Arginine and Proline Metabolism	SPAC22G7.06c SPAC16.03c SPBC56F2.09c	6.3.5.5, 2.1.3.2
deoxyhypusine synthase		H2O[c] + Q6[m] + spmd[c] -> 13dAMPp[c] + 4abutn[c] + Q6H2[m]	Arginine and Proline Metabolism	SPBC1271.04c	2.5.1.46
L-erythro-4-Hydroxyglutamate:2-oxoglutarate aminotransferase (predicted)	Cytosol	AKG + e4HGLU -> 4H2Oglt + GLU	Arginine and Proline Metabolism	SPAC10F6.13c	2.6.1.1
L-erythro-4-Hydroxyglutamate:2-oxoglutarate aminotransferase (predicted)	Mitochondria	AKG + e4HGLU -> 4H2Oglt + GLU	Arginine and Proline Metabolism	SPBC725.01	2.6.1.1
L-glutamate 5-semialdehyde dehydratase	Cytosol	GLU5SA <-> 1PYR5c + H + H2O	Arginine and Proline Metabolism		
L-glutamate 5-semialdehyde dehydratase	Mitochondria	GLU5SA <-> 1PYR5c + H + H2O	Arginine and Proline Metabolism		
glutamate-5-semialdehyde dehydrogenase	Cytosol	GLU5p + H + NADPH -> GLU5SA + NADP + Pi	Arginine and Proline Metabolism	SPAC821.11	1.2.1.41
glutamate 5-kinase	Cytosol	ATP + GLU -> ADP + GLU5p	Arginine and Proline Metabolism	SPAC17H9.13c	2.7.2.11
L-hydroxyproline reductase (NAD)	Cytosol	1p3H5c + 2 H + NADH -> 4HPROT + NAD	Arginine and Proline Metabolism	SPAPYUG7.05	1.5.1.2
L-hydroxyproline reductase (NADP)	Cytosol	1p3H5c + 2 H + NADPH -> 4HPROT + NADP	Arginine and Proline Metabolism	SPAPYUG7.05	1.5.1.2
L-hydroxyproline dehydrogenase (NAD)	Mitochondria	4HPROT + NAD -> 1p3H5c + 2 H + NADH	Arginine and Proline Metabolism	SPBC24C6.04	
L-hydroxyproline dehydrogenase (NADP)	Mitochondria	4HPROT + NADP -> 1p3H5c + 2 H + NADPH	Arginine and Proline Metabolism	SPBC24C6.04	
ornithine carbamoyltransferase	Cytosol	cbp +ORN -> CITR + H + Pi	Arginine and Proline Metabolism	SPAC4G9.10	2.1.3.3
Ornithine Decarboxylase	Cytosol	H +ORN -> CO2 + ptrc	Arginine and Proline Metabolism	SPAC144.04c	4.1.1.17
ornithine transaminase	Cytosol	AKG +ORN -> GLU + GLU5SA	Arginine and Proline Metabolism	SPBC21C3.08c	2.6.1.13
glutamate N-acetyltransferase (predicted)	Mitochondria	acorn + GLU -> acGLU +ORN	Arginine and Proline Metabolism	SPBC1271.14	2.3.1.35, 2.3.1.1
pyrroline-5-carboxylate reductase	Cytosol	1PYR5c + 2 H + NADPH -> NADP + PRO	Arginine and Proline Metabolism	SPAPYUG7.05	1.5.1.2
L-1-pyrroline-3-hydroxy-5-carboxylate dehydrogenase	Mitochondria	1p3H5c + 2 H2O + NAD -> e4HGLU + H + NADH	Arginine and Proline Metabolism	SPBC24C6.04	
L-1-Pyrroline-3-hydroxy-5-carboxylate spontaneous conversion to L-4-Hydroxyglutamate semialdehyde	Mitochondria	1p3H5c + H + H2O <-> 4HGLUSA	Arginine and Proline Metabolism		
spermidine synthase	Cytosol	ametam + ptrc -> 5mta + H + spmd	Arginine and Proline Metabolism	SPBC12C2.07c	2.5.1.16
L-asparaginase	Golgi apparatus	ASN + H2O -> ASP + NH4	Asparagine metabolism	SPAC186.03 SPAC977.12 SPBPB8B6.05c SPBPB21E7.09	3.5.1.1
L-asparaginase	Cytoplasm	ASN + H2O -> ASP + NH4	Asparagine metabolism	SPAC186.03 SPAC977.12 SPBPB8B6.05c SPBPB21E7.09	3.5.1.1
L-asparaginase	Extracellular	ASN + H2O -> ASP + NH4	Asparagine metabolism	SPAC186.03 SPAC977.12 SPBPB8B6.05c SPBPB21E7.09	3.5.1.1
Isocitrate dehydrogenase (NAD+)	Mitochondria	ICIT + NAD <-> AKG + CO2 + NADH	Citric Acid Cycle	SPAC11G7.03 SPBC902.05c	1.1.1.41
isocitrate dehydrogenase (NADP)	Cytosol	ICIT + NADP <-> AKG + CO2 + NADPH	Citric Acid Cycle	SPAC11G7.03 SPBC902.05c	1.1.1.41
Isocitrate dehydrogenase (NADP+)	Mitochondria	ICIT + NADP <-> AKG + CO2 + NADPH	Citric Acid Cycle	SPAC11G7.03 SPBC902.05c	1.1.1.41
Isocitrate dehydrogenase (NADP+)	Peroxisome	ICIT + NADP <-> AKG + CO2 + NADPH	Citric Acid Cycle	SPAC11G7.03 SPBC902.05c	1.1.1.41
ATP citrate synthase	Mitochondria	ADP + Pi + ACCoA + OAA -> CIT + ATP + CoA	Citric Acid Cycle	SPAC22A12.16	2.3.3.8
aconitase	Cytosol	CIT <-> ICIT	Citric Acid Cycle	SPAC24C9.06c SPBP4H10.15	4.2.1.3
Aconitate hydratase	Mitochondria	CIT <-> ICIT	Citric Acid Cycle	SPAC24C9.06c SPBP4H10.15	4.2.1.3
oxoglutarate dehydrogenase (lipoamide)	Mitochondria	AKG + H + lpam <-> CO2 + sdHlam	Citric Acid Cycle	SPBC3H7.03c	1.2.4.2
oxoglutarate dehydrogenase (dihydrolipoamide S-succinyltransferase)	Mitochondria	CoA + sdHlam -> dHlam + SUCCoA	Citric Acid Cycle	SPBC3H7.03c	1.2.4.2
citrate synthase	Mitochondria	ACCoA + H2O + OAA -> CIT + CoA + H	Citric Acid Cycle	SPAC6C3.04	2.3.3.1
citrate synthase	Peroxisome	ACCoA + H2O + OAA -> CIT + CoA + H	Citric Acid Cycle	SPAC6C3.04	2.3.3.1
Itaconate--CoA ligase (ADP-forming)	Mitochondria	ATP + CoA + itacon <-> ADP + itACCoA + Pi	Citric Acid Cycle	SPCC1620.08 SPAC16E8.17c	6.2.1.4, 6.2.1.5
succinate dehydrogenase (ubiquinone-6)	Mitochondria	Q6 + SUCC <-> FUM + Q6H2	Citric Acid Cycle	SPBP23A10.16+SPAC140.01+SPCC330.12c+SPAC1556.02c SPBP23A10.16+SPAC140.01+SPCC330.12c+SPAC1556.02c SPAC140.01+SPAC1556.02c+SPCC330.12c+SPBP23A10.16 SPAC140.01+SPAC1556.02c+SPBP23A10.16+SPCC330.12c	1.3.5.1
Succinate--CoA ligase (ADP-forming)	Mitochondria	ATP + CoA + SUCC <-> ADP + Pi + SUCCoA	Citric Acid Cycle	SPCC1620.08 SPAC16E8.17c	6.2.1.4, 6.2.1.5
3-Isopropylmalate 3-methyltransferase	Cytosol	3c2Hmp + SAM -> 3ipmmest + SAH	Complex Alcohol Metabolism	SPAC25B8.09 SPAC25B8.10	
4-Methyl-2-oxopentanoate decarboxylase	Cytosol	4MOP + H -> 3mbald + CO2	Complex Alcohol Metabolism	SPAC13A11.06 SPAC3G9.11c SPAC186.09 SPAC1F8.07c	4.1.1.1
Isoamyl acetate-hydrolyzing esterase	Cytosol	H2O + iamac -> AC + H + IAMOH	Complex Alcohol Metabolism	SPCC126.10	
Isobutyl acetate-hydrolyzing esterase	Cytosol	H2O + ibutac -> AC + H + IBUTOH	Complex Alcohol Metabolism	SPCC126.10	
Ethyl acetate-hydrolyzing esterase	Cytosol	aces + H2O -> AC + ETOH + H	Complex Alcohol Metabolism	SPCC126.10	
aldehyde dehydrogenase (2-methylbutanol, NAD)	Cytosol	2mbald + H + NADH -> 2mbtoH + NAD	Complex Alcohol Metabolism	SPBC1773.06c SPAC5H10.06c	1.1.1.1
aldehyde dehydrogenase (2-methylbutanol, NAD)	Mitochondria	2mbald + H + NADH -> 2mbtoH + NAD	Complex Alcohol Metabolism	SPCC13B11.01	1.1.1.1
aldehyde dehydrogenase (2-methylbutanol, NADP)	Cytosol	2mbald + H + NADPH -> 2mbtoH + NADP	Complex Alcohol Metabolism		
aldehyde dehydrogenase (isobutyl alcohol, NAD)	Cytosol	2mppal + H + NADH -> IBUTOH + NAD	Complex Alcohol Metabolism	SPBC1773.06c SPAC5H10.06c	1.1.1.1
aldehyde dehydrogenase (isobutyl alcohol, NAD)	Mitochondria	2mppal + H + NADH -> IBUTOH + NAD	Complex Alcohol Metabolism	SPCC13B11.01	1.1.1.1
aldehyde dehydrogenase (isoamyl alcohol, NAD)	Cytosol	3mbald + H + NADH -> IAMOH + NAD	Complex Alcohol Metabolism	SPBC1773.06c SPAC5H10.06c	1.1.1.1
aldehyde dehydrogenase (isoamyl alcohol, NAD)	Mitochondria	3mbald + H + NADH -> IAMOH + NAD	Complex Alcohol Metabolism	SPCC13B11.01	1.1.1.1
aldehyde dehydrogenase (2-phenylethanol, NAD)	Cytosol	H + NADH + PACALD -> 2PHETOH + NAD	Complex Alcohol Metabolism	SPBC1773.06c SPAC5H10.06c	1.1.1.1
aldehyde dehydrogenase (2-phenylethanol, NAD)	Mitochondria	H + NADH + PACALD -> 2PHETOH + NAD	Complex Alcohol Metabolism	SPCC13B11.01	1.1.1.1
aldehyde dehydrogenase (tryptophol, NAD)	Cytosol	H + id3acald + NADH -> ind3etH + NAD	Complex Alcohol Metabolism	SPBC1773.06c SPAC5H10.06c	1.1.1.1
aldehyde dehydrogenase (tryptophol, NAD)	Mitochondria	H + id3acald + NADH -> ind3etH + NAD	Complex Alcohol Metabolism	SPCC13B11.01	1.1.1.1
Alcohol Acetyltransferase (ethanol)	Cytosol	ACCoA + ETOH -> aces + CoA	Complex Alcohol Metabolism		
Alcohol Acetyltransferase (isoamyl alcohol)	Cytosol	ACCoA + IAMOH -> CoA + iamac	Complex Alcohol Metabolism		
Alcohol Acetyltransferase (2-methylbutanol)	Cytosol	2mbtoH + ACCoA -> 2mbac + CoA	Complex Alcohol Metabolism		
Alcohol Acetyltransferase (isobutyl alcohol)	Cytosol	ACCoA + IBUTOH -> CoA + ibutac	Complex Alcohol Metabolism		
Alcohol Acetyltransferase (Phenylethanol alcohol)	Cytosol	2PHETOH + ACCoA -> CoA + PHEAC	Complex Alcohol Metabolism		
cysteine synthase	Mitochondria	ACSER + H2S -> AC + CYS + H	Cysteine Metabolism	SPBC36.04 SPAC3A12.17c	2.5.1.47
3',5'-bisphosphate nucleotidase	Nucleus	H2O + PAP -> AMP + Pi	Cysteine Metabolism	SPCC1753.04	3.1.3.7

phosphoadenylyl-sulfate reductase (thioredoxin)	Nucleus	PAPS + TRDrd -> 2 H + PAP + SO3 + TRDox	Cysteine Metabolism	SPAC13G7.06	1.8.4.8
2-hydroxybutyrate cotransport with proton		2Hb[e] + H[e] <-> 2Hb[c] + H[c]	Cysteine Metabolism		
adenylyl-sulfate kinase	Cytosol	APS + ATP -> ADP + H + PAPS	Cysteine Metabolism	SPAC1782.11	2.7.1.25
adenylyl-sulfate kinase	Nucleus	APS + ATP -> ADP + H + PAPS	Cysteine Metabolism	SPAC1782.11	2.7.1.25
3',5'-bisphosphate nucleotidase	Cytosol	H2O + PAP -> AMP + Pi	Cysteine Metabolism	SPCC1753.04	3.1.3.7
phosphoadenylyl-sulfate reductase (thioredoxin)	Cytosol	PAPS + TRDrd -> 2 H + PAP + SO3 + TRDox	Cysteine Metabolism	SPAC13G7.06	1.8.4.8
sulfate adenylyltransferase	Cytosol	ATP + H + SO4 -> APS + PPi	Cysteine Metabolism	SPBC27.08c	2.7.7.4
sulfate adenylyltransferase	Mitochondria	ATP + H + SO4 -> APS + PPi	Cysteine Metabolism	SPBC27.08c	2.7.7.4
serine O-acetyltransferase	Cytosol	ACCoA + SER -> ACSER + CoA	Cysteine Metabolism	SPAC1039.08	2.3.1.30
sulfite reductase (NADPH2)	Cytosol	3 H2O + H2S + 3 NADP <-> 5 H + 3 NADPH + SO3	Cysteine Metabolism	SPCC584.01c SPAC10F6.01c	1.8.1.2
fatty-acid--CoA ligase (tetradecanoate)	Golgi apparatus	ATP + CoA + C140 <-> AMP + PPi + C140CoA	Fatty Acid Biosynthesis	SPBP4H10.11c	
fatty acid synthase (n-C14:1)	Endoplasmic Reticulum	C120 + 4 H + MALCoA + 3 NADPH + O2 -> CO2 + CoA + 3 H2O + 3 NADP + C141	Fatty Acid Biosynthesis	SPAC1B2.03c	
fatty acid synthase (n-C16:1)	Endoplasmic Reticulum	C140 + 4 H + MALCoA + 3 NADPH + O2 -> CO2 + CoA + 3 H2O + 3 NADP + C161	Fatty Acid Biosynthesis	SPAC1B2.03c	
fatty acid synthase (n-C18:1)	Endoplasmic Reticulum	C160 + 4 H + MALCoA + 3 NADPH + O2 -> CO2 + CoA + 3 H2O + 3 NADP + C181	Fatty Acid Biosynthesis	SPAC1B2.03c	
fatty-acid--CoA ligase (tetradecanoate)	Endoplasmic Reticulum	ATP + CoA + C140 <-> AMP + PPi + C140CoA	Fatty Acid Biosynthesis	SPBC18H10.02	
Palmitoyl-CoA desaturase (n-C16:0CoA -> n-C16:1CoA)	Endoplasmic Reticulum	H + NADPH + O2 + C160CoA -> 2 H2O + C161CoA + NADP	Fatty Acid Biosynthesis	SPCC1281.06c	1.14.19.1
stearoyl-CoA desaturase (n-C18:0CoA -> n-C18:1CoA)	Endoplasmic Reticulum	H + NADPH + O2 + C180CoA -> 2 H2O + NADP + C181CoA	Fatty Acid Biosynthesis	SPCC1281.06c	1.14.19.1
acetyl-CoA C-acetyltransferase	Cytosol	2 ACCoA -> AACoA + CoA	Fatty Acid Biosynthesis	SPBC215.09c	2.3.1.9
acetyl-CoA C-acetyltransferase	Mitochondria	2 ACCoA -> AACoA + CoA	Fatty Acid Biosynthesis	SPBC215.09c	2.3.1.9
acetyl-CoA carboxylase reaction	Cytosol	ACCoA + ATP + HCO3 <-> ADP + H + MALCoA + Pi	Fatty Acid Biosynthesis	SPAC56E4.04c	6.4.1.2
Acetyl-Coa carboxylase	Mitochondria	ACCoA + ATP + HCO3 <-> ADP + H + MALCoA + Pi	Fatty Acid Biosynthesis	SPAC56E4.04c	6.4.1.2
Acetyl-CoA ACP transacylase	Cytosol	ACP + ACCoA <-> ACACP + CoA	Fatty Acid Biosynthesis	SPAC926.09c + SPAC4A8.11c	3.1.2.14
Acetyl-CoA ACP transacylase	Mitochondria	ACP + ACCoA <-> ACACP + CoA	Fatty Acid Biosynthesis	SPBC1105.15c SPAC4H3.09 SPAC3G9.02 SPAC11G7.05c	
Myristicoyl-CoA desaturase (n-C14:0CoA -> n-C14:1CoA)	Cytosol	H + NADPH + O2 + C140CoA -> 2 H2O + NADP + C141CoA	Fatty Acid Biosynthesis		
Oleoyl-CoA desaturase (n-C18:1CoA -> n-C18:2CoA)	Cytosol	H + NADPH + O2 + C181CoA -> 2 H2O + NADP + C182CoA	Fatty Acid Biosynthesis		
fatty-acyl-ACP hydrolase	Cytosol	H2O + C120ACP <-> ACP + H + C120	Fatty Acid Biosynthesis	SPAC926.09c+ SPAC4A8.11c	3.1.2.14
fatty-acyl-ACP hydrolase	Cytosol	H2O + C140ACP <-> ACP + H + C140	Fatty Acid Biosynthesis	SPAC926.09c+ SPAC4A8.11c	3.1.2.14
fatty-acyl-ACP hydrolase	Cytosol	H2O + C141ACP <-> ACP + H + C141	Fatty Acid Biosynthesis	SPAC926.09c+ SPAC4A8.11c	3.1.2.14
fatty-acyl-ACP hydrolase	Cytosol	H2O + C160ACP <-> ACP + H + C160	Fatty Acid Biosynthesis	SPAC926.09c+ SPAC4A8.11c	3.1.2.14
fatty-acyl-ACP hydrolase	Cytosol	H2O + C161ACP <-> ACP + H + C161	Fatty Acid Biosynthesis	SPAC926.09c+ SPAC4A8.11c	3.1.2.14
fatty-acyl-ACP hydrolase	Cytosol	H2O + C180ACP <-> ACP + H + C180	Fatty Acid Biosynthesis	SPAC926.09c+ SPAC4A8.11c	3.1.2.14
fatty-acyl-ACP hydrolase	Cytosol	H2O + C181ACP <-> ACP + H + C181	Fatty Acid Biosynthesis	SPAC926.09c+ SPAC4A8.11c	3.1.2.14
fatty-acyl-ACP hydrolase	Cytosol	H2O + C182ACP <-> ACP + H + C182	Fatty Acid Biosynthesis	SPAC926.09c+ SPAC4A8.11c	3.1.2.14
fatty-acid--CoA ligase (tetradecanoate)	Cytosol	ATP + CoA + C140 <-> AMP + PPi + C140CoA	Fatty Acid Biosynthesis	SPBP4H10.11c SPBC18H10.02	6.2.1.3
fatty-acid--CoA ligase (tetradecenoate)	Cytosol	ATP + CoA + C141 <-> AMP + PPi + C141CoA	Fatty Acid Biosynthesis	SPBP4H10.11c SPBC18H10.02	
fatty-acid--CoA ligase (hexadecanoate)	Cytosol	ATP + CoA + C160 <-> AMP + PPi + C160CoA	Fatty Acid Biosynthesis	SPBP4H10.11c SPBC18H10.02	
fatty-acid--CoA ligase (hexadecenoate)	Cytosol	ATP + CoA + C161 <-> AMP + PPi + C161CoA	Fatty Acid Biosynthesis	SPBP4H10.11c SPBC18H10.02	
fatty-acid--CoA ligase (Octodecanoate)	Cytosol	ATP + CoA + C180 <-> AMP + PPi + C180CoA	Fatty Acid Biosynthesis	SPBP4H10.11c SPBC18H10.02	
fatty-acid--CoA ligase (Octodecanoate)	Cytosol	ATP + CoA + C181 <-> AMP + PPi + C181CoA	Fatty Acid Biosynthesis	SPBP4H10.11c SPBC18H10.02	
fatty-acid--CoA ligase (Octodecanoate)	Cytosol	ATP + CoA + C182 <-> AMP + PPi + C182CoA	Fatty Acid Biosynthesis	SPBP4H10.11c SPBC18H10.02	
fatty acid synthase (n-C10:0)	Cytosol	3 H + MALCoA + 2 NADPH + C080 -> CO2 + CoA + C100 + H2O + 2 NADP	Fatty Acid Biosynthesis	SPAC56E4.04c SPAC926.09c+SPAC4A8.11c	6.3.4.14, 3.1.2.14
fatty-acyl-ACP synthase (n-C10:0ACP)	Mitochondria	3 H + MALACP + 2 NADPH + C080ACP -> ACP + CO2 + C100ACP + H2O + 2 NADP	Fatty Acid Biosynthesis	SPBC1105.15c SPAC4H3.09 SPAC3G9.02 SPAC11G7.05c SPAC26F1.04c	
fatty acyl-CoA synthase (n-C10:0CoA)	Cytosol	3 H + MALCoA + 2 NADPH + C080CoA -> CO2 + CoA + C100CoA + H2O + 2 NADP	Fatty Acid Biosynthesis	SPAC56E4.04c SPAC926.09c+SPAC4A8.11c	6.3.4.14, 3.1.2.14
fatty acid synthase (n-C12:0)	Cytosol	C100 + 3 H + MALCoA + 2 NADPH -> CO2 + CoA + C120 + H2O + 2 NADP	Fatty Acid Biosynthesis	SPAC56E4.04c SPAC926.09c+SPAC4A8.11c	6.3.4.14, 3.1.2.14
fatty-acyl-ACP synthase (n-C12:0ACP)	Mitochondria	C100ACP + 3 H + MALACP + 2 NADPH -> ACP + CO2 + C120ACP + H2O + 2 NADP	Fatty Acid Biosynthesis	SPBC1105.15c SPAC4H3.09 SPAC3G9.02 SPAC11G7.05c SPAC26F1.04c	
fatty-acyl-CoA synthase (n-C12:0CoA)	Cytosol	C100CoA + 3 H + MALCoA + 2 NADPH -> CO2 + CoA + C120CoA + H2O + 2 NADP	Fatty Acid Biosynthesis	SPAC56E4.04c SPAC926.09c+SPAC4A8.11c	6.3.4.14, 3.1.2.14
fatty acid synthase (n-C14:0)	Cytosol	C120 + 3 H + MALCoA + 2 NADPH -> CO2 + CoA + H2O + 2 NADP + C140	Fatty Acid Biosynthesis	SPAC56E4.04c SPAC926.09c+SPAC4A8.11c	6.3.4.14, 3.1.2.14
fatty-acyl-ACP synthase (n-C14:0ACP)	Mitochondria	C120ACP + 3 H + MALACP + 2 NADPH -> ACP + CO2 + H2O + C140ACP + 2 NADP	Fatty Acid Biosynthesis	SPBC1105.15c SPAC4H3.09 SPAC3G9.02 SPAC11G7.05c SPAC26F1.04c	
fatty-acyl-CoA synthase (n-C14:0CoA)	Cytosol	C120CoA + 3 H + MALCoA + 2 NADPH -> CO2 + CoA + H2O + 2 NADP + C140CoA	Fatty Acid Biosynthesis	SPAC56E4.04c SPAC926.09c+SPAC4A8.11c	6.3.4.14, 3.1.2.14
fatty-acyl-ACP synthase (n-C14:1ACP)	Mitochondria	C120ACP + 4 H + MALACP + 3 NADPH + O2 -> ACP + CO2 + 3 H2O + 3 NADP + C141ACP	Fatty Acid Biosynthesis	SPBC1105.15c SPAC4H3.09 SPAC3G9.02 SPAC11G7.05c SPAC26F1.04c	
fatty acid synthase (n-C16:0)	Cytosol	3 H + MALCoA + 2 NADPH + C140 -> CO2 + CoA + H2O + C160 + 2 NADP	Fatty Acid Biosynthesis	SPAC56E4.04c SPAC926.09c+SPAC4A8.11c	6.3.4.14, 3.1.2.14

fatty-acyl-ACP synthase (n-C16:0ACP)	Mitochondria	3 H + MALACP + C140ACP + 2 NADPH -> ACP + CO2 + H2O + 2 NADP + C160ACP	Fatty Acid Biosynthesis	SPBC1105.15c<br>SPAC4H3.09<br>SPAC3G9.02<br>SPAC11G7.05c<br>SPAC26F1.04c	
fatty-acyl-CoA synthase (n-C16:0CoA)	Cytosol	3 H + MALCoA + 2 NADPH + C140CoA -> CO2 + CoA + H2O + 2 NADP + C160CoA	Fatty Acid Biosynthesis	SPAC56E4.04c<br>SPAC926.09c+SPAC4A8.11c	6.3.4.14, 3.1.2.14
fatty-acyl-ACP synthase (n-C16:1ACP)	Mitochondria	4 H + MALACP + C140ACP + 3 NADPH + O2 -> ACP + CO2 + 3 H2O + C161ACP + 3 NADP	Fatty Acid Biosynthesis	SPBC1105.15c<br>SPAC4H3.09<br>SPAC3G9.02<br>SPAC11G7.05c<br>SPAC26F1.04c	
fatty acid synthase (n-C18:0)	Cytosol	3 H + C160 + MALCoA + 2 NADPH -> CO2 + CoA + H2O + 2 NADP + C180	Fatty Acid Biosynthesis	SPAC56E4.04c<br>SPAC926.09c+SPAC4A8.11c	6.3.4.14, 3.1.2.14
fatty-acyl-ACP synthase (n-C18:0ACP)	Mitochondria	3 H + MALACP + 2 NADPH + C160ACP -> ACP + CO2 + H2O + 2 NADP + C180ACP	Fatty Acid Biosynthesis	SPBC1105.15c<br>SPAC4H3.09<br>SPAC3G9.02<br>SPAC11G7.05c<br>SPAC26F1.04c	
fatty-acyl-CoA synthase (n-C18:0CoA)	Cytosol	3 H + MALCoA + 2 NADPH + C160CoA -> CO2 + CoA + H2O + 2 NADP + C180CoA	Fatty Acid Biosynthesis	SPAC56E4.04c<br>SPAC926.09c+SPAC4A8.11c	6.3.4.14, 3.1.2.14
fatty-acyl-ACP synthase (n-C18:1ACP)	Mitochondria	4 H + MALACP + 3 NADPH + O2 + C160ACP -> ACP + CO2 + 3 H2O + 3 NADP + C181ACP	Fatty Acid Biosynthesis	SPBC1105.15c<br>SPAC4H3.09<br>SPAC3G9.02<br>SPAC11G7.05c<br>SPAC26F1.04c	
fatty-acyl-ACP synthase (n-C18:2ACP)	Mitochondria	5 H + MALACP + 4 NADPH + 2 O2 + C160ACP -> ACP + CO2 + 5 H2O + 4 NADP + C182ACP	Fatty Acid Biosynthesis	SPBC1105.15c<br>SPAC4H3.09<br>SPAC3G9.02<br>SPAC11G7.05c<br>SPAC26F1.04c	
fatty acid synthase (n-C8:0), lumped reaction	Cytosol	ACCoA + 8 H + 3 MALCoA + 6 NADPH -> 3 CO2 + 4 CoA + 2 H2O + 6 NADP + C080	Fatty Acid Biosynthesis	SPAC56E4.04c<br>SPAC926.09c+SPAC4A8.11c	6.3.4.14, 3.1.2.14
fatty acyl-ACP synthase (n-C8:0ACP), lumped reaction	Mitochondria	ACACP + 9 H + 3 MALACP + 6 NADPH -> 3 ACP + 3 CO2 + 3 H2O + 6 NADP + C080ACP	Fatty Acid Biosynthesis	SPBC1105.15c<br>SPAC4H3.09<br>SPAC3G9.02<br>SPAC11G7.05c<br>SPAC26F1.04c	
fatty acyl-CoA synthase (n-C8:0CoA), lumped reaction	Cytosol	ACCoA + 9 H + 3 MALCoA + 6 NADPH -> 3 CO2 + 3 CoA + 3 H2O + 6 NADP + C080CoA	Fatty Acid Biosynthesis	SPAC56E4.04c<br>SPAC926.09c+SPAC4A8.11c	6.3.4.14, 3.1.2.14
Malonyl-CoA-ACP transacylase	Cytosol	ACP + MALCoA <-> CoA + MALACP	Fatty Acid Biosynthesis	SPAC926.09c+<br>SPAC4A8.11c	3.1.2.14
Malonyl-CoA-ACP transacylase	Mitochondria	ACP + MALCoA <-> CoA + MALACP	Fatty Acid Biosynthesis	SPBC1105.15c<br>SPAC4H3.09<br>SPAC3G9.02<br>SPAC11G7.05c<br>SPAC26F1.04c	
dihydropteroate synthase	Cytosol	2AHHMD + PABA -> DHPT + PPi	Folate Metabolism	SPBC1734.03	4.1.2.25
dihydropteroate synthase	Cytosol	2AHHMP + PABA -> DHPT + H2O	Folate Metabolism	SPBC1734.03	4.1.2.25
2-amino-4-hydroxy-6-hydroxymethyldihydropteridine diphosphokinase	Cytosol	2AHHMP + ATP -> 2AHHMD + AMP + H	Folate Metabolism	SPBC1734.03	4.1.2.25
dihydroneopterin aldolase	Cytosol	DHNPT -> 2AHHMP + gcald + H	Folate Metabolism	SPBC1734.03	4.1.2.25
5,10-methylenetetrahydrofolatereductase (NADPH)	Mitochondria	2 H + MLTHF + NADPH -> 5mTHF + NADP	Folate Metabolism	SPAC56F8.10	1.5.1.20
Tetrahydrofolate:L-glutamate gamma-ligase (ADP-forming)	Mitochondria	ATP + GLU + THF <-> ADP + H + Pi + THFGLU	Folate Metabolism	SPAC227.09<br>SPBC1709.17	6.3.2.17
Tetrahydrofolate:L-glutamate gamma-ligase (ADP-forming)	Nucleus	ATP + GLU + THF <-> ADP + H + Pi + THFGLU	Folate Metabolism	SPBC1709.17	6.3.2.17
alkaline phosphatase (Dihydroneopterin), vacuole	Vacuole	AHDT + 3 H2O -> DHNPT + 2 H + 3 Pi	Folate Metabolism	SPBC14F5.13c	3.1.3.1
5,10-methylenetetrahydrofolatereductase (NADPH)	Nucleus	2 H + MLTHF + NADPH -> 5mTHF + NADP	Folate Metabolism	SPAC56F8.10	1.5.1.20
GTP cyclohydrolase I	Nucleus	GTP + H2O -> AHDT + FORM + H	Folate Metabolism	SPAC17A5.13	3.5.4.16
methylenetetrahydrofolate dehydrogenase (NAD)	Nucleus	MLTHF + NAD -> METHF + NADH	Folate Metabolism	SPBC1711.04	1.5.1.15
4-aminobenzoate synthase	Cytosol	ADCHO -> PABA + H + PYR	Folate Metabolism	SPBC19G7.02	
4-aminobenzoate synthase	Nucleus	ADCHO -> PABA + H + PYR	Folate Metabolism	SPBC19G7.02	
para-aminobenzoate synthase	Cytosol	CHOR + GLN -> ADCHO + GLU	Folate Metabolism	SPBP8B7.29	
aspartate oxidase		ASP[c] + fad[m] -> FADH2[m] + H[c] + IMASP[c]	Folate Metabolism		
dihydrofolate reductase	Cytosol	DHF + H + NADPH -> NADP + THF	Folate Metabolism	SPCC1223.08c	1.5.1.3
dihydrofolate reductase	Mitochondria	DHF + H + NADPH -> NADP + THF	Folate Metabolism	SPCC1223.08c	1.5.1.3
dihydrofolate synthase	Cytosol	ATP + DHPT + GLU -> ADP + DHF + H + Pi	Folate Metabolism		
dihydroneopterin aldolase	Mitochondria	DHNPT -> 2AHHMP + gcald + H	Folate Metabolism	SPBC1734.03	4.1.2.25
		gcald -> pydxn			
dihydropteroate synthase	Mitochondria	2AHHMP + PABA -> DHPT + H2O	Folate Metabolism	SPBC1734.03	4.1.2.25
Dihydroneopterin monophosphate dephosphorylase	Nucleus	dHpmp + H2O -> DHNPT + Pi	Folate Metabolism	SPBC14F5.13c	3.6.3.16
Dihydroneopterin monophosphate dephosphorylase	Endoplasmic Reticulum	dHpmp + H2O -> DHNPT + Pi	Folate Metabolism	SPBC14F5.13c	3.6.3.16
Dihydroneopterin monophosphate dephosphorylase	Cytosol	dHpmp + H2O -> DHNPT + Pi	Folate Metabolism	SPBC14F5.13c	3.6.3.16
Dihydroneopterin triphosphate pyrophosphatase	Cytosol	AHDT + H2O -> dHpmp + H + PPi	Folate Metabolism		
dihydropteroate synthase	Mitochondria	2AHHMD + PABA -> DHPT + PPi	Folate Metabolism	SPBC1734.03	4.1.2.25
5-formethyltetrahydrofolate cyclo-ligase	Cytosol	5FTHF + ATP -> ADP + METHF + Pi	Folate Metabolism	SPBC1703.08c	
5-formethyltetrahydrofolate cyclo-ligase	Mitochondria	5FTHF + ATP -> ADP + METHF + Pi	Folate Metabolism	SPBC1703.08c	6.3.3.2
5-Formyltetrahydrofolate:10-Formyltetrahydrofolate isomerase	Cytosol	5FTHF + ATP + H2O -> 10FTHF + ADP + H + Pi	Folate Metabolism		
formate-tetrahydrofolate ligase	Cytosol	ATP + FORM + THF -> 10FTHF + ADP + Pi	Folate Metabolism	SPBC839.16	6.3.4.3
formate-tetrahydrofolate ligase	Mitochondria	ATP + FORM + THF -> 10FTHF + ADP + Pi	Folate Metabolism	SPBC839.16	6.3.4.3
GTP cyclohydrolase I	Cytosol	GTP + H2O -> AHDT + FORM + H	Folate Metabolism	SPAC17A5.13	3.5.4.16
2-amino-4-hydroxy-6-hydroxymethyldihydropteridine diphosphokinase	Mitochondria	2AHHMP + ATP -> 2AHHMD + AMP + H	Folate Metabolism	SPBC1734.03	4.1.2.25
methenyltetrahydrofolate cyclohydrolase	Cytosol	H2O + METHF <-> 10FTHF + H	Folate Metabolism	SPBC839.16	6.3.4.3
methenyltetrahydrifikate cyclohydrolase	Mitochondria	H2O + METHF <-> 10FTHF + H	Folate Metabolism	SPBC839.16	6.3.4.3
methylenetetrahydrofolate dehydrogenase (NADP)	Cytosol	MLTHF + NADP <-> METHF + NADPH	Folate Metabolism	SPBC839.16	6.3.4.3
methylenetetrahydrofolate dehydrogenase (NADP)	Mitochondria	MLTHF + NADP <-> METHF + NADPH	Folate Metabolism	SPBC839.16	6.3.4.3
methylenetetrahydrofolate dehydrogenase (NAD)	Cytosol	MLTHF + NAD -> METHF + NADH	Folate Metabolism	SPBC1711.04	1.5.1.15
5,10-methylenetetrahydrofolatereductase (NADPH)	Cytosol	2 H + MLTHF + NADPH -> 5mTHF + NADP	Folate Metabolism	SPAC56F8.10<br>SPAC343.10	1.5.1.20
quinolinate synthase	Cytosol	DHAP + IMASP -> 2 H2O + Pi + Quln	Folate Metabolism		
tetrahydrofolate aminomethyltransferase	Mitochondria	H2O + METHF -> 5FTHF + H	Folate Metabolism	SPAC13G6.06c<br>SPAC31G5.14<br>SPBP19A11.01<br>SPAC26F1.03+SPBC30D10.13c	1.4.4.2, 2.1.2.10

Tetrahydrofolate:L-glutamate gamma-ligase (ADP-forming)	Cytosol	ATP + GLU + THF <-> ADP + H + Pi + THFGLU	Folate Metabolism	SPAC227.09 SPBC1709.17	6.3.2.17
phosphomannomutase	Nucleus	man1p <-> man6p	Fructose and Mannose Metabolism	SPAC1556.07	5.4.2.8
mannose-6-phosphate isomerase	Nucleus	man6p <-> F6P	Fructose and Mannose Metabolism	SPBC2G2.16	5.3.1.8
Fructose-2,6-bisphosphate 2-phosphatase	Cytosol	f26bp + H2O -> F6P + Pi	Fructose and Mannose Metabolism	SPAC732.02c (main) SPAPB17E12.14c SPAC144.17c	3.1.3.46
fructose-1-phosphate kinase	Cytosol	ATP + f1p -> ADP + FDP + H	Fructose and Mannose Metabolism		
mannose-1-phosphate guanylyltransferase	Cytosol	GTP + H + man1p -> GDPmann + PPi	Fructose and Mannose Metabolism	SPCC1906.01	2.7.7.13
mannose-6-phosphate isomerase	Cytosol	man6p <-> F6P	Fructose and Mannose Metabolism	SPBC2G2.16	5.3.1.8
6-phosphofructo-2-kinase	Cytosol	ATP + F6P -> ADP + f26bp + H	Fructose and Mannose Metabolism	SPAPB17E12.14c SPAC222.13c	2.7.1.105
phosphomannomutase	Cytosol	man1p <-> man6p	Fructose and Mannose Metabolism	SPAC1556.07	5.4.2.8
	Extracellular	raffin + H2O -> melib + fru	Galactose metabolism	SPCC191.11	3.2.1.26
	Cytosol	raffin + H2O -> melib + fru	Galactose metabolism	SPAC8E11.01c	3.2.1.26
	Cytosol	sta + H2O -> mant + fru	Galactose metabolism	SPAC8E11.01c	3.2.1.26
	Endoplasmic Reticulum	sta + H2O -> raffin + gal	Galactose metabolism	SPAC869.07c	3.2.1.22
	Extracellular	sta + H2O -> raffin + gal	Galactose metabolism	SPAC869.07c	3.2.1.22
	Endoplasmic Reticulum	mant + H2O -> gal + melib	Galactose metabolism	SPAC869.07c	3.2.1.22
	Extracellular	mant + H2O -> gal + melib	Galactose metabolism	SPAC869.07c	3.2.1.22
1-alpha-D-Galactosyl-myo-inositol galactohydrolase	Endoplasmic Reticulum	GALI + H2O <-> inost + gal	Galactose metabolism	SPAC869.07c	3.2.1.22
1-alpha-D-Galactosyl-myo-inositol galactohydrolase	Extracellular	GALI + H2O <-> inost + gal	Galactose metabolism	SPAC869.07c	3.2.1.22
Melibiitol galactohydrolase	Endoplasmic Reticulum	melt + H2O <-> sbt-D + gal	Galactose metabolism	SPAC869.07c	3.2.1.22
Melibiitol galactohydrolase	Extracellular	melt + H2O <-> sbt-D + gal	Galactose metabolism	SPAC869.07c	3.2.1.22
EPimelibiose galactohydrolase	Endoplasmic Reticulum	emp + H2O <-> man + gal	Galactose metabolism	SPAC869.07c	3.2.1.22
EPimelibiose galactohydrolase	Extracellular	emp + H2O <-> man + gal	Galactose metabolism	SPAC869.07c	3.2.1.22
Galactosylglycerol galactohydrolase	Endoplasmic Reticulum	ggl + H2O <-> gal + GLYC	Galactose metabolism	SPAC869.07c	3.2.1.22
Galactosylglycerol galactohydrolase	Extracellular	ggl + H2O <-> gal + GLYC	Galactose metabolism	SPAC869.07c	3.2.1.22
alpha-D-glucoside glucohydrase	Nucleus	6dg + H2O -> gal + GLC	Galactose metabolism	SPBC1683.07 SPAC1039.11c SPAPB24D3.10c	3.2.1.20
UTP-glucose-1-phosphate URIdylyltransferase	Nucleus	g1p + H + UTP <-> PPi + UDPg	Galactose metabolism	SPCC794.10 SPCC1322.04	2.7.7.9
galactose-1-phosphate URIdylyltransferase	Nucleus	gal1p + H + UTP <-> PPi + UDPgal	Galactose metabolism	SPBPB2B2.10c	2.7.7.12
UDPglucose--hexose-1-phosphate URIdylyltransferase	Nucleus	gal1p + UDPg <-> g1p + UDPgal	Galactose metabolism	SPBPB2B2.10c	2.7.7.12
alpha-glucosidase	Nucleus	H2O + MALT -> 2 GLC	Galactose metabolism	SPBC1683.07 SPAC1039.11c SPAPB24D3.10c	3.2.1.20
UDPglucose 4-ePimerase	Nucleus	UDPg <-> UDPgal	Galactose metabolism	SPBPB2B2.12c SPBC365.14c	5.1.3.3
alpha-D-glucoside glucohydrase	Cytosol	6dg + H2O -> gal + GLC	Galactose metabolism	SPBC1683.07 SPAC1039.11c SPAPB24D3.10c	3.2.1.20
EPimelibiose galactohydrolase	Cytosol	epm + H2O <-> gal + man	Galactose metabolism	SPAC869.07c	
1-alpha-D-Galactosyl-myo-inositol galactohydrolase	Cytosol	GALI + H2O <-> gal + inost	Galactose metabolism	SPAC869.07c	
galactokinase	Cytosol	ATP + gal -> ADP + gal1p + H	Galactose metabolism	SPBPB2B2.13	2.7.1.6
a-galactosidase (melibiose)	Cytosol	H2O + melib -> gal + GLC	Galactose metabolism	SPAC869.07c	
galactose-1-phosphate URIdylyltransferase	Cytosol	gal1p + H + UTP <-> PPi + UDPgal	Galactose metabolism	SPBPB2B2.10c	2.7.7.12
UTP-glucose-1-phosphate URIdylyltransferase	Cytosol	g1p + H + UTP <-> PPi + UDPg	Galactose metabolism	SPCC794.10 SPCC1322.04	2.7.7.9
Galactosylglycerol galactohydrolase	Cytosol	ggl + H2O <-> gal + GLYC	Galactose metabolism	SPAC869.07c	
alpha-glucosidase	Cytosol	H2O + MALT -> 2 GLC	Galactose metabolism	SPBC1683.07 SPAC1039.11c SPAPB24D3.10c	3.2.1.20
Melibiitol galactohydrolase	Cytosol	melt + H2O <-> sbt-D + gal	Galactose metabolism	SPAC869.07c	
Raffinose galactohydrolase	Extracellular	raffin + H2O -> gal + sucr	Galactose metabolism	SPAC869.07c	3.2.1.22
Raffinose galactohydrolase	Endoplasmic Reticulum	raffin + H2O -> gal + sucr	Galactose metabolism	SPAC869.07c	3.2.1.22
UDPglucose 4-ePimerase	Cytosol	UDPg <-> UDPgal	Galactose metabolism	SPBPB2B2.12c SPBC365.14c	5.1.3.3
UDPglucose--hexose-1-phosphate URIdylyltransferase	Cytosol	gal1p + UDPg <-> g1p + UDPgal	Galactose metabolism	SPBPB2B2.10c	2.7.7.12
4-aminobutyrate transaminase	Mitochondria	4abut + AKG -> GLU + SUCSAL	Glutamate metabolism	SPAC19D5.07	2.6.1.19
glutamate synthase (NADH2)	Mitochondria	AKG + GLN + H + NADH -> 2 GLU + NAD	Glutamate metabolism	SPAPB1E7.07	1.4.1.13, 1.4.1.14
glutamate dehydrogenase (NADP)	Mitochondria	AKG + H + NADPH + NH4 -> GLU + H2O + NADP	Glutamate metabolism	SPCC622.12c	1.4.1.4
chitin synthase	Cytoplasm	UDPacgal -> cHitin + H + UDP	Glutamate metabolism	SPAC19G12.03	2.4.1.16
chitin synthase	Endoplasmic Reticulum	UDPacgal -> cHitin + H + UDP	Glutamate metabolism	SPAC13G6.12c SPBC1709.01	2.4.1.16
UDP-N-acetylglucosamine diphosphorylase	Nucleus	acgam1p + H + UTP <-> PPi + UDPacgal	Glutamate metabolism	SPBC1289.08	2.7.7.23
phosphoacetylglucosamine mutase	Nucleus	acgam6p <-> acgam1p	Glutamate metabolism	SPAC1296.01c SPAC13C5.05c	5.4.2.3
glutamate dehydrogenase (NADP)	Nucleus	AKG + H + NADPH + NH4 -> GLU + H2O + NADP	Glutamate metabolism	SPCC622.12c	1.4.1.4
phosphoglucosamine mutase	Nucleus	gam1p <-> gam6p	Glutamate metabolism	SPAC1296.01c SPAC13C5.05c	5.4.2.3
glutathione oxidoreductase	Nucleus	GTHox + H + NADPH -> 2 GTHrd + NADP	Glutamate metabolism	SPBC17A3.07 SPAC4F10.20 SPBC26H8.06	1.8.1.7
4-aminobutyrate transaminase	Cytosol	4abut + AKG -> GLU + SUCSAL	Glutamate metabolism	SPAC19D5.07	2.6.1.19
phosphoacetylglucosamine mutase	Cytosol	acgam6p <-> acgam1p	Glutamate metabolism	SPAC1296.01c SPAC13C5.05c	5.4.2.3
glucosamine-6-phosphate deaminase	Cytosol	gam6p + H2O -> F6P + NH4	Glutamate metabolism		
glutamate dehydrogenase (NAD)	Cytosol	GLU + H2O + NAD -> AKG + H + NADH + NH4	Glutamate metabolism	SPCC132.04c	1.4.1.2
glutamate dehydrogenase (NADP)	Cytosol	AKG + H + NADPH + NH4 -> GLU + H2O + NADP	Glutamate metabolism	SPCC622.12c	1.4.1.4
glutamate synthase (NADH2)	Cytosol	AKG + GLN + H + NADH -> 2 GLU + NAD	Glutamate metabolism	SPAPB1E7.07	1.4.1.13, 1.4.1.14
glutathione oxidoreductase	Mitochondria	GTHox + H + NADPH -> 2 GTHrd + NADP	Glutamate metabolism	SPBC17A3.07	1.8.1.7
glutathione peroxidase	Mitochondria	2 GTHrd + H2O2 <-> GTHox + 2 H2O	Glutamate metabolism	SPAPB2B4.02	
1-pyrroline-5-carboxylate dehydrogenase	Mitochondria	1PYR5c + 2 H2O + NAD -> GLU + H + NADH	Glutamate metabolism		
phosphoglucosamine mutase	Cytosol	gam1p <-> gam6p	Glutamate metabolism	SPAC1296.01c SPAC13C5.05c	5.4.2.3
D1-pyrroline-5-carboxylate dehydrogenase	Mitochondria	GLU5SA + H2O + NADP -> GLU + 2 H + NADPH	Glutamate metabolism	SPBC24C6.04	
succinate-semialdehyde dehydrogenase (NADP) (predicted)	Cytosol	H2O + NADP + SUCSAL -> 2 H + NADPH + SUCC	Glutamate metabolism	SPAC1002.12c SPAC139.05	
UDP-N-acetylglucosamine diphosphorylase	Cytosol	acgam1p + H + UTP <-> PPi + UDPacgal	Glutamate metabolism	SPBC1289.08	2.7.7.23
N-acetylglucosamine-6-phosphate synthase	Cytosol	acCoA + gam6p <-> acgam6p + CoA + H	Glutamate metabolism	SPAC16E8.03	

glutamine synthetase	Nucleus	ATP + GLU + NH4 -> ADP + GLN + H + Pi	Glutamine Metabolism	SPAC23H4.06	6.3.1.2
anthranilate synthase	Cytosol	CHOR + GLN -> antH + GLU + H + PYR	Glutamine Metabolism	SPBC1539.09c<br>SPCC1442.09	
glutamine-fructose-6-phosphate transaminase	Cytosol	F6P + GLN -> gam6p + GLU	Glutamine Metabolism	SPBC12C2.11	2.6.1.16
glutamine synthetase	Cytosol	ATP + GLU + NH4 -> ADP + GLN + H + Pi	Glutamine Metabolism	SPAC23H4.06	6.3.1.2
glutaminase	Cytosol	GLN + H2O -> GLU + NH4	Glutamine Metabolism		3.5.1.2
Glycerol dehydrogenase (NADP-dependent)	Mitochondria	GLYC + NADP -> DHA + H + NADPH	Glycerolipid Metabolism	SPAC13F5.03c	1.1.1.6
triglycerol synthesis	Endoplasmic Reticulum	0.01 12dgr + 0.02 C100CoA + 0.06 C120CoA + 0.17 C161CoA + 0.09 C182CoA + 0.24 C181CoA + 0.27 C160CoA + 0.05 C180CoA + 0.1 C140CoA -> CoA + 0.01 TAG	Glycerolipid Metabolism	SPAC13G7.05<br>SPCP1E11.05	2.3.1.26
phosphatidylcholine-diacylglycerol acyltransferase	Endoplasmic Reticulum	12dgr + pc -> 1agpc + TAG	Glycerolipid Metabolism	SPBC776.14<br>SPCC1235.15<br>SPCC1840.04	2.3.1.158
glycerol-3-phosphate dehydrogenase (NAD)	Nucleus	DHAP + H + NADH -> GLYC3p + NAD	Glycerolipid Metabolism	SPBC215.05	1.1.1.8
dihydroxyacetone kinase	Cytosol	ATP + DHA -> ADP + DHAP + H	Glycerolipid Metabolism	SPAC22A12.11<br>SPAC977.16c	2.7.1.29
glycerol-3-phosphate dehydrogenase (NAD)	Cytosol	DHAP + H + NADH -> GLYC3p + NAD	Glycerolipid Metabolism	SPAC23D3.04c<br>SPBC215.05	1.1.1.8
glycerol-3-phosphate dehydrogenase (FAD)	Mitochondria	fad + GLYC3p -> DHAP + FADH2	Glycerolipid Metabolism	SPCC1223.03c	1.1.5.3
glycerol-3-phosphatase	Cytosol	GLYC3p + H2O -> GLYC + Pi	Glycerolipid Metabolism		
glycerol kinase	Cytosol	ATP + GLYC -> ADP + GLYC3p + H	Glycerolipid Metabolism		
Glycerophosphodiester phosphodiesterase (Glycerophosphocholine)	Endoplasmic Reticulum	G3Pc + H2O -> CHOL + GLYC3p + H	Glycerophospholipid Metabolism	SPAC4D7.02c	3.1.4.46
Glycerophosphodiester phosphodiesterase (Glycerophosphocholine)	Mitochondria	G3Pc + H2O -> CHOL + GLYC3p + H	Glycerophospholipid Metabolism	SPAC4D7.02c	3.1.4.46
Triacylglycerol lipase	Cytosol	H2O + 0.01 TAG -> 0.01 12dgr + 0.02 C100 + 0.06 C120 + H + 0.27 C160 + 0.17 C161 + 0.05 C180 + 0.24 C181 + 0.09 C182 + 0.1 C140	Glycerolipid Metabolism	SPCC1450.16c	3.1.1.3
Alanine glyoxylate aminotransferase	Cytosol	ALA + GLX -> GLY + PYR	Glycine and Serine Metabolism		
glycine-cleavage complex (lipoamide)	Mitochondria	GLY + H + lpam <-> alpam + CO2	Glycine and Serine Metabolism	SPAC13G6.06c<br>SPAC1002.09c<br>SPAC31G5.14<br>SPBP19A11.01	1.4.4.2,<br>1.8.1.4,<br>2.1.2.10
glycine-cleavage system (lipoamide)	Mitochondria	alpam + THF -> dHlam + MLTHF + NH4	Glycine and Serine Metabolism	SPAC13G6.06c<br>SPAC1002.09c<br>SPAC31G5.14<br>SPBP19A11.01	1.4.4.2,<br>1.8.1.4,<br>2.1.2.10
glycine-cleavage complex (lipoamide)	Mitochondria	dHlam + NAD <-> H + lpam + NADH	Glycine and Serine Metabolism	SPAC13G6.06c<br>SPAC1002.09c<br>SPAC31G5.14<br>SPBP19A11.01	1.4.4.2,<br>1.8.1.4,<br>2.1.2.10
glycine-cleavage complex (lipoylprotein)	Mitochondria	GLY + H + lpro <-> alpro + CO2	Glycine and Serine Metabolism	SPAC13G6.06c<br>SPAC1002.09c<br>SPAC31G5.14<br>SPBP19A11.01	1.4.4.2,<br>1.8.1.4,<br>2.1.2.10
glycine-cleavage complex (lipoylprotein)	Mitochondria	alpro + THF -> dHlpro + MLTHF + NH4	Glycine and Serine Metabolism	SPAC13G6.06c<br>SPAC1002.09c<br>SPAC31G5.14<br>SPBP19A11.01	1.4.4.2,<br>1.8.1.4,<br>2.1.2.10
glycine-cleavage complex (lipoylprotein)	Mitochondria	dHlpro + NAD <-> H + lpro + NADH	Glycine and Serine Metabolism	SPAC13G6.06c<br>SPAC1002.09c<br>SPAC31G5.14<br>SPBP19A11.01	1.4.4.2,<br>1.8.1.4,<br>2.1.2.10
glycine hydroxymethyltransferase	Cytosol	SER + THF <-> GLY + H2O + MLTHF	Glycine and Serine Metabolism	SPAC24C9.12c	2.1.2.1
glycine hydroxymethyltransferase	Mitochondria	SER + THF <-> GLY + H2O + MLTHF	Glycine and Serine Metabolism	SPAC18G6.04c	2.1.2.1
glycine cleavage system	Mitochondria	GLY + NAD + THF -> CO2 + MLTHF + NADH + NH4	Glycine and Serine Metabolism	SPAC13G6.06c<br>SPAC1002.09c<br>SPAC31G5.14<br>SPBP19A11.01	1.4.4.2,<br>1.8.1.4,<br>2.1.2.10
homoserine dehydrogenase (NADH)	Cytosol	aspSA + H + NADH -> Hom-L + NAD	Glycine and Serine Metabolism	SPBC776.03	1.1.1.3
homoserine dehydrogenase (NADP)	Cytosol	aspSA + H + NADPH -> Hom-L + NADP	Glycine and Serine Metabolism	SPBC776.03	1.1.1.3
homoserine kinase	Cytosol	ATP + Hom-L -> ADP + H + PHOM	Glycine and Serine Metabolism	SPBC4C3.03	2.7.1.39
L-serine dehydrogenase	Cytosol	NADP + SER <-> 2amSA + H + NADPH	Glycine and Serine Metabolism	SPAC521.03	
phosphoglycerate dehydrogenase	Cytosol	3pg + NAD -> 3pHp + H + NADH	Glycine and Serine Metabolism	SPCC364.07	1.1.1.95
phosphoserine transaminase	Cytosol	3pHp + GLU -> AKG + PSER	Glycine and Serine Metabolism	SPAC1F12.07	2.6.1.52
phosphoserine phosphatase (L-serine)	Cytosol	H2O + PSER -> Pi + SER	Glycine and Serine Metabolism	SPBC3H7.07c	3.1.3.3
L-serine deaminase	Cytosol	SER -> NH4 + PYR	Glycine and Serine Metabolism	SPBC1677.03c	4.3.1.19
Diphosphoglyceromutase	Nucleus	13dpg <-> 23dpg + H	Glycolysis/Gluconeogenesis	SPAC26F1.06<br>SPAC1687.21	5.4.2.1
phosphoglycerate mutase	Nucleus	2pg <-> 3pg	Glycolysis/Gluconeogenesis	SPAC26F1.06	5.4.2.1
enolase	Nucleus	2pg <-> H2O + PEP	Glycolysis/Gluconeogenesis	SPBC1815.01<br>SPBPB21E7.01c	4.2.1.11
phosphoglycerate kinase	Nucleus	3pg + ATP <-> 13dpg + ADP	Glycolysis/Gluconeogenesis	SPBC14F5.04c	2.7.2.3
hexokinase (D-fructose:ATP)	Nucleus	ATP + fru -> ADP + F6P + H	Glycolysis/Gluconeogenesis	SPAC24H6.04	2.7.1.1
hexokinase (D-glucose:ATP)	Nucleus	ATP + GLC -> ADP + G6P + H	Glycolysis/Gluconeogenesis	SPAC4F8.07c<br>SPAC24H6.04	2.7.1.1
Glucokinase	Nucleus	ATP + GLC -> ADP + G6P-B + H	Glycolysis/Gluconeogenesis	SPAC4F8.07c	2.7.1.1
hexokinase (D-mannose:ATP)	Nucleus	ATP + man -> ADP + H + man6p	Glycolysis/Gluconeogenesis	SPAC24H6.04	2.7.1.1
triose-phosphate isomerase	Nucleus	DHAP <-> G3P	Glycolysis/Gluconeogenesis	SPCC24B10.21	5.3.1.1
D-Fructose 1-phosphate D-glyceraldehyde-3-phosphate-lyase	Nucleus	f1p <-> DHAP + GLYald	Glycolysis/Gluconeogenesis	SPBC19C2.07	4.1.2.13
fructose-bisphosphate aldolase	Nucleus	FDP <-> DHAP + G3P	Glycolysis/Gluconeogenesis	SPBC19C2.07	4.1.2.13
Sedoheptulose 1,7-bisphosphate D-glyceraldehyde-3-phosphate-lyase	Nucleus	s17bp <-> DHAP + E4P	Glycolysis/Gluconeogenesis	SPBC19C2.07	4.1.2.13
Diphosphoglyceromutase	Cytosol	13dpg <-> 23dpg + H	Glycolysis/Gluconeogenesis	SPAC26F1.06<br>SPAC1687.21	5.4.2.1
enolase	Cytosol	2pg <-> H2O + PEP	Glycolysis/Gluconeogenesis	SPBC1815.01	4.2.1.11
fructose-bisphosphate aldolase	Cytosol	FDP <-> DHAP + G3P	Glycolysis/Gluconeogenesis	SPBC19C2.07	4.1.2.13
D-Fructose 1-phosphate D-glyceraldehyde-3-phosphate-lyase	Cytosol	f1p <-> DHAP + GLYald	Glycolysis/Gluconeogenesis	SPBC19C2.07	4.1.2.13
Sedoheptulose 1,7-bisphosphate D-glyceraldehyde-3-phosphate-lyase	Cytosol	s17bp <-> DHAP + E4P	Glycolysis/Gluconeogenesis	SPBC19C2.07	4.1.2.13
Glucose-6-phosphate isomerase	Cytosol	G6P <-> G6P-B	Glycolysis/Gluconeogenesis	SPBC1604.05	5.3.1.9
Glucose-6-phosphate isomerase	Cytosol	G6P-B <-> F6P	Glycolysis/Gluconeogenesis	SPBC1604.05	5.3.1.9
glyceraldehyde-3-phosphate dehydrogenase	Cytosol	G3P + NAD + Pi <-> 13dpg + H + NADH	Glycolysis/Gluconeogenesis	SPBC32F12.11<br>SPBC354.12	1.2.1.12
Glucokinase	Cytosol	ATP + GLC -> ADP + G6P-B + H	Glycolysis/Gluconeogenesis	SPAC4F8.07c	2.7.1.1
hexokinase (D-glucose:ATP)	Cytosol	ATP + GLC -> ADP + G6P + H	Glycolysis/Gluconeogenesis	SPAC4F8.07c<br>SPAC24H6.04	2.7.1.1
hexokinase (D-mannose:ATP)	Cytosol	ATP + man -> ADP + H + man6p	Glycolysis/Gluconeogenesis	SPAC24H6.04	2.7.1.1
hexokinase (D-fructose:ATP)	Cytosol	ATP + fru -> ADP + F6P + H	Glycolysis/Gluconeogenesis	SPAC24H6.04	2.7.1.1
pyruvate dehydrogenase (dihydrolipoamide dehydrogenase)	Mitochondria	dHlam + NAD -> H + lpam + NADH	Glycolysis/Gluconeogenesis	SPBC3H7.03c<br>SPAC1002.09c<br>SPBC776.15c	1.2.4.2,<br>1.8.1.4,<br>2.3.1.61

pyruvate dehydrogenase	Mitochondria	CoA + NAD + PYR -> ACCoA + CO2 + NADH	Glycolysis/Gluconeogenesis	SPAC1002.09c SPCC794.07 SPBC30D10.13c, SPAC26F1.03 SPCC1259.09c	1.8.1.4, 2.3.1.12, 1.2.4.1
phosphofructokinase	Cytosol	ATP + F6P -> ADP + FDP + H	Glycolysis/Gluconeogenesis	SPBC16H5.02	2.7.1.11
Phosphofructokinase	Cytosol	ATP + taG6P-D -> ADP + H + taGDP-D	Glycolysis/Gluconeogenesis	SPBC16H5.02	2.7.1.11
phosphofructokinase (s7p)	Cytosol	ATP + s7p -> ADP + H + s17bp	Glycolysis/Gluconeogenesis	SPBC16H5.02	2.7.1.11
glucose-6-phosphate isomerase	Cytosol	G6P <-> F6P	Glycolysis/Gluconeogenesis	SPBC1604.05	5.3.1.9
phosphoglycerate kinase	Cytosol	13dpg + ADP <-> 3pg + ATP	Glycolysis/Gluconeogenesis	SPBC14F5.04c	2.7.2.3
phosphoglycerate mutase	Cytosol	3pg <-> 2pg	Glycolysis/Gluconeogenesis	SPAC26F1.06	5.4.2.1
pyruvate kinase	Cytosol	ADP + H + PEP -> ATP + PYR	Glycolysis/Gluconeogenesis	SPAC4H3.10c	2.7.1.40
triose-phosphate isomerase	Cytosol	DHAP <-> G3P	Glycolysis/Gluconeogenesis	SPCC24B10.21	5.3.1.1
pyruvate decarboxylase	Cytosol	PYR + H -> ACAL + CO2	Glycolysis/Gluconeogenesis	SPAC13A11.06 SPAC3G9.11c SPAC186.09 SPAC1F8.07c	4.1.1.1
Dolichol kinase	Cytosol	CTP + doliCHOL -> CDP + dolp + H	Glycoprotein Metabolism	SPCC63.10c	2.7.1.108
Dolichol kinase	Endoplasmic Reticulum	CTP + doliCHOL -> CDP + dolp + H	Glycoprotein Metabolism	SPCC63.10c	2.7.1.108
Dolichyl-phosphate-mannose--protein mannosyltransferase	Endoplasmic Reticulum	dolmanp -> dolp + H + mannan	Glycoprotein Metabolism	SPAC22A12.07c SPAPB1E7.09 SPBC16C6.09	2.4.1.109
Dolichyl-phosphate D-mannosyltransferase	Endoplasmic Reticulum	dolp[c] + GDPmann[c] -> dolmanp[r]+ GDP[c]	Glycoprotein Metabolism	SPAC31G5.16c, SPBC21B10.11, SPBC1677.02	2.4.1.83
GlycoliPid 1,2-alpha-D-mannosyltransferase	Golgi apparatus	GDPmann + m2maccHITPpdol -> GDP + H + M3MACCHITPPDOL	Glycoprotein Metabolism	SPBC19C7.12c SPBC1773.08c	
GlycoliPid 1,2-alpha-D-mannosyltransferase	Golgi apparatus	GDPmann + M3MACCHITPPDOL -> GDP + H + m4maccHITPpdol	Glycoprotein Metabolism	SPBC19C7.12c SPBC1773.08c	
GlycoliPid 1,3-alpha-D-mannosyltransferase	Golgi apparatus	GDPmann + maccHITPpdol -> GDP + H + m1maccHITPpdol	Glycoprotein Metabolism		
GlycoliPid 1,6-alpha-D-mannosyltransferase	Golgi apparatus	GDPmann + m1maccHITPpdol -> GDP + H + m2maccHITPpdol	Glycoprotein Metabolism		
Ureidoglycolate hydrolase	Nucleus	2 H + H2O + URDGLYC <-> CO2 + GLX + 2 NH4	Histidine Metabolism	SPAC19G12.04	3.5.3.19
Allantoate amidinohydrolase	Nucleus	alltt + H2O <-> URDGLYC + urea	Histidine Metabolism	SPAC1F7.09c	3.5.3.4
hnRNP arginine N-methyltransferase	Nucleus	SAM + HIS -> NPmeHis + SAH + H	Histidine Metabolism	SPAC890.07c	2.1.1.-
phosphoribosylpyrophosphate synthetase	Nucleus	ATP + R5P <-> AMP + H + PRPP	Histidine Metabolism	SPAC4A8.14	2.7.6.1
imidazoleglycerol-phosphate dehydratase	Nucleus	eiG3P -> H2O + imacp	Histidine Metabolism	SPBC21H7.07c	4.2.1.19
histidinol-phosphate transaminase	Nucleus	GLU + imacp -> AKG + Hisp	Histidine Metabolism	SPBC11B10.02c	2.6.1.9
1-(5-phosphoribosyl)-5-[(5-phosphoribosylamino)methylideneamino)imidazole-4-carboxamide isomerase	Nucleus	prfp -> prlp	Histidine Metabolism	SPAC3F10.09	5.3.1.16
Allantoate amidinohydrolase	Cytosol	alltt + H2O <-> URDGLYC + urea	Histidine Metabolism	SPAC1F7.09c	3.5.3.4
hnRNP arginine N-methyltransferase	Cytosol	SAM + HIS -> NPmeHis + SAH + H	Histidine Metabolism	SPAC890.07c	2.1.1.-
ATP phosphoribosyltransferase	Cytosol	ATP + PRPP -> PPi + prbATP	Histidine Metabolism	SPAC25G10.05c	2.4.2.17
histidinol dehydrogenase	Cytosol	H2O + Histd + 2 NAD -> 3 H + HIS + 2 NADH	Histidine Metabolism	SPBC29A3.02c SPBC1711.13	1.1.1.23
histidinol dehydrogenase	Nucleus	H2O + Histd + 2 NAD -> 3 H + HIS + 2 NADH	Histidine Metabolism	SPBC29A3.02c SPBC1711.13	1.1.1.23
histidinol-phosphate transaminase	Cytosol	GLU + imacp -> AKG + Hisp	Histidine Metabolism	SPBC11B10.02c	2.6.1.9
imidazoleglycerol-phosphate dehydratase	Cytosol	eiG3P -> H2O + imacp	Histidine Metabolism	SPBC21H7.07c	4.2.1.19
phosphoribosyl-AMP cyclohydrolase	Cytosol	H2O + prbAMP -> prfp	Histidine Metabolism	SPBC29A3.02c SPBC1711.13	3.5.4.19, 3.6.1.31
phosphoribosyl-ATP pyrophosphatase	Cytosol	H2O + prbATP -> H + PPi + prbAMP	Histidine Metabolism	SPBC29A3.02c SPBC1711.13	3.5.4.19, 3.6.1.31
phosphoribosyl-AMP cyclohydrolase	Nucleus	H2O + prbAMP -> prfp	Histidine Metabolism	SPBC29A3.02c SPBC1711.13	3.5.4.19, 3.6.1.31
phosphoribosyl-ATP pyrophosphatase	Nucleus	H2O + prbATP -> H + PPi + prbAMP	Histidine Metabolism	SPBC29A3.02c SPBC1711.13	3.5.4.19, 3.6.1.31
1-(5-phosphoribosyl)-5-[(5-phosphoribosylamino)methylideneamino)imidazole-4-carboxamide isomerase	Cytosol	prfp -> prlp	Histidine Metabolism	SPAC3F10.09	5.3.1.16
phosphoribosylpyrophosphate synthetase	Cytosol	ATP + R5P <-> AMP + H + PRPP	Histidine Metabolism	SPCC1620.06c SPAC4A8.14 SPBC3D6.06c	2.7.6.1
Ureidoglycolate hydrolase	Cytosol	2 H + H2O + URDGLYC <-> CO2 + GLX + 2 NH4	Histidine Metabolism	SPAC19G12.04	3.5.3.19
S-Formylglutathione hydralase	Cytosol	SfGLUttH + H2O <-> FORM + GTHrd + H	Methane Metabolism		3.1.2.12
5'-methylthioadenosine phosphorylase	Nucleus	5mta + Pi -> 5mdr1p + ADE	Methionine Metabolism	SPAC16C9.02c	2.4.2.28
methionine synthase	Nucleus	5mTHF + HCYS -> H + MET + THF	Methionine Metabolism	SPAC9.09	2.1.1.14
O-acetylhomoserine (thiol)-lyase	Nucleus	ACHMS + CH4S -> AC + H + MET	Methionine Metabolism	SPBC428.11	2.5.1.49
metb1	Nucleus	ACHMS + CYS -> AC + cyst-L + H	Methionine Metabolism	SPBC15D4.09c	2.5.1.48
O-acetylhomoserine (thiol)-lyase	Nucleus	ACHMS + H2S -> AC + H + HCYS	Methionine Metabolism	SPBC428.11	2.5.1.49
diphthine synthase	Nucleus	SAM + CAPHIS -> SAH + CMAPHIS + H	Methionine Metabolism	SPCC576.14	2.1.1.98
adenosylmethionine decarboxylase	Nucleus	SAM + H -> ametam + CO2	Methionine Metabolism	SPBP4H10.05c	4.1.1.50
methionine adenosyltransferase	Nucleus	ATP + H2O + MET -> SAM + Pi + PPi	Methionine Metabolism	SPBC14F5.05c	2.5.1.6
2,3-diketo-5-methylthio-1-phosphopentane degradation reaction	Nucleus	dkmpp + 3 H2O -> 2kmb + FORM + 6 H + Pi	Methionine Metabolism	SPAC644.08 SPBC887.01	3.1.3.77, 1.13.11.53, 1.13.11.54
O-succinylhomoserine lyase (elimination)	Nucleus	H2O + SUCHMS <-> 2obut + H + NH4 + SUCC	Methionine Metabolism	SPBC15D4.09c	2.5.1.48
5-methyltetrahydropteroyltriglutamate-homocysteine S-methyltransferase	Nucleus	HCYS + MHPGLU -> HpGLU + MET	Methionine Metabolism	SPAC9.09	2.1.1.14
adenosylmethionine decarboxylase	Cytosol	SAM + H -> ametam + CO2	Methionine Metabolism	SPBP4H10.05c	4.1.1.50
adenosylhomocysteinase	Cytosol	SAH + H2O -> ADN + HCYS	Methionine Metabolism	SPBC8D2.18c	3.3.1.1
O-acetylhomoserine (thiol)-lyase	Cytosol	ACHMS + CH4S -> AC + H + MET	Methionine Metabolism	SPBC428.11	2.5.1.49
O-acetylhomoserine (thiol)-lyase	Cytosol	ACHMS + H2S -> AC + H + HCYS	Methionine Metabolism	SPBC428.11	2.5.1.49
cystathionine b-lyase	Cytosol	cyst-L + H2O -> HCYS + NH4 + PYR	Methionine Metabolism		
cystathione b-lyase	Peroxisome	cyst-L + H2O -> HCYS + NH4 + PYR	Methionine Metabolism	SPCC11E10.01	4.4.1.8
diphthine synthase	Cytosol	SAM + CAPHIS -> SAH + CMAPHIS + H	Methionine Metabolism	SPCC576.14	2.1.1.98
2,3-diketo-5-methylthio-1-phosphopentane degradation reaction	Cytosol	dkmpp + 3 H2O -> 2kmb + FORM + 6 H + Pi	Methionine Metabolism	SPAC644.08 SPBC887.01	3.1.3.77, 1.13.11.53, 1.13.11.54
homoserine O-trans-acetylase	Cytosol	ACCoA + Hom-L <-> ACHMS + CoA	Methionine Metabolism	SPBC56F2.11	2.3.1.31
5-Methylthio-5-deoxy-D-ribulose 1-phosphate dehydratase	Cytosol	5mdru1p -> dkmpp + H2O	Methionine Metabolism		
methionine adenosyltransferase	Cytosol	ATP + H2O + MET -> SAM + Pi + PPi	Methionine Metabolism	SPBC14F5.05c	2.5.1.6
metb1	Cytosol	ACHMS + CYS -> AC + cyst-L + H	Methionine Metabolism	SPBC15D4.09c	2.5.1.48
methionine synthase	Cytosol	5mTHF + HCYS -> H + MET + THF	Methionine Metabolism	SPAC9.09	2.1.1.14
5-methyltetrahydropteroyltriglutamate-homocysteine S-methyltransferase	Cytosol	HCYS + MHPGLU -> HpGLU + MET	Methionine Metabolism	SPAC9.09	2.1.1.14
5'-methylthioadenosine phosphorylase	Cytosol	5mta + Pi -> 5mdr1p + ADE	Methionine Metabolism	SPAC16C9.02c	2.4.2.28
5-methylthioribose-1-phosphate isomerase	Cytosol	5mdr1p <-> 5mdru1p	Methionine Metabolism		

O-succinylhomoserine lyase (L-cysteine)	Cytosol	CYS + SUCHMS -> cyst-L + H + SUCC	Methionine Metabolism		
O-succinylhomoserine lyase (elimination)	Cytosol	H2O + SUCHMS <-> 2obut + H + NH4 + SUCC	Methionine Metabolism	SPBC15D4.09c	2.5.1.48
2-keto-4-methylthiobutyrate transamination	Cytosol	2kmb + GLU -> AKG + MET	Methionine Metabolism		
NAD diphosphatase	Cytosol	H2O + NAD -> AMP + 2 H + nmn	NAD Biosynthesis	SPBC1778.03c	3.6.1.22
pURIne-nucleoside phosphorylase (Adenosine)	Nucleus	ADN + Pi <-> ADE + r1p	NAD Biosynthesis	SPAC1805.16c	2.4.2.1
nicotinate-nucleotide adenylyltransferase	Nucleus	ATP + H + nicrnt -> dNAD + PPi	NAD Biosynthesis	SPAC806.06c	2.7.7.1
nicotinamide-nucleotide adenylyltransferase	Nucleus	ATP + H + nmn -> NAD + PPi	NAD Biosynthesis	SPAC806.06c	2.7.7.1
ribosylnicotinamide kinase	Nucleus	ATP + rnam -> ADP + H + nmn	NAD Biosynthesis	SPBP22H7.06	2.7.1.-
pURIne-nucleoside phosphorylase (Guanosine)	Nucleus	GSN + Pi <-> GUA + r1p	NAD Biosynthesis	SPAC1805.16c	2.4.2.1
NAPRTase	Mitochondria	H + nac + PRPP -> nicrnt + PPi	NAD Biosynthesis	SPAC1486.06	2.4.2.11
NAPRTase	Nucleus	H + nac + PRPP -> nicrnt + PPi	NAD Biosynthesis	SPAC1486.06	2.4.2.11
NAD diphosphatase	Nucleus	H2O + NAD -> AMP + 2 H + nmn	NAD Biosynthesis	SPBC1778.03c	3.6.1.22
NAD kinase	Cytosol	ATP + NAD -> ADP + H + NADP	NAD Biosynthesis	SPCC24B10.02c	2.7.1.23
NAD kinase	Mitochondria	ATP + NAD -> ADP + H + NADP	NAD Biosynthesis	SPAC323.01c	2.7.1.86
NADP phosphatase	Cytosol	H2O + NADP -> NAD + Pi	NAD Biosynthesis		
NADP phosphatase	Mitochondria	H2O + NADP -> NAD + Pi	NAD Biosynthesis		
NAD synthase (nh3)	Cytosol	ATP + dNAD + NH4 -> AMP + H + NAD + PPi	NAD Biosynthesis	SPCC553.02	6.3.5.1
NAD synthase (nh3)	Nucleus	ATP + dNAD + NH4 -> AMP + H + NAD + PPi	NAD Biosynthesis	SPCC553.02	6.3.5.1
nicotinamide-nucleotide adenylyltransferase	Cytosol	ATP + H + nmn -> NAD + PPi	NAD Biosynthesis	SPAC806.06c	2.7.7.1
nicotinate-nucleotide adenylyltransferase	Cytosol	ATP + H + nicrnt -> dNAD + PPi	NAD Biosynthesis	SPAC806.06c	2.7.7.1
Nicotinamide N-methyltransferase	Cytosol	SAM + ncam -> 1mncam + SAH	NAD Biosynthesis	SPAC8F11.09c	2.1.1.1
pURIne-nucleoside phosphorylase	Cytosol	Pi + rnam <-> H + ncam + r1p	NAD Biosynthesis		
ribosylnicotinamide kinase	Cytosol	ATP + rnam -> ADP + H + nmn	NAD Biosynthesis	SPBP22H7.06	2.7.1.-
carbonate dehydratase	Cytosol	CO2 + H2O -> H2CO3	Nitrogen Metabolism	SPBP8B7.05c	4.2.1.1
Urea amidohydrolase	Cytosol	urea + H2O -> CO2 + 2 NH4	Nitrogen Metabolism	SPAC1952.11c	3.5.1.5
nitrilase	Nucleus	2 H2O + ind3acnl -> ind3ac + NH4	Nitrogen Metabolism	SPBC651.02 SPCC965.09	3.5.-.-
nitrilase	Cytosol	2 H2O + ind3acnl -> ind3ac + NH4	Nitrogen Metabolism	SPBC651.02 SPCC965.09	3.5.-.-
dinucleoside tetraphosphatase	Cytosol	gp4g + H2O <-> GTP + GMP	Nucleotide Salvage Pathway	SPCC4G3.02	3.6.1.17
P1,P4-Bis(5'-nucleosyl)-tetraphosphate nucleotidohydrolase	Cytosol	xp4x + H2O <-> XTP + XMP	Nucleotide Salvage Pathway	SPCC4G3.02	3.6.1.17
P1,P4-bis(5'-URIdyl)-tetraphosphate URIdylohydrolase	Cytosol	up4u + H2O -> UTP + UMP	Nucleotide Salvage Pathway	SPCC4G3.02	3.6.1.17
dinucleoside tetraphosphatase	Mitochondria	gp4g + H2O <-> GTP + GMP	Nucleotide Salvage Pathway	SPCC4G3.02	3.6.1.17
P1,P4-Bis(5'-nucleosyl)-tetraphosphate nucleotidohydrolase	Mitochondria	xp4x + H2O <-> XTP + XMP	Nucleotide Salvage Pathway	SPCC4G3.02	3.6.1.17
P1,P4-bis(5'-URIdyl)-tetraphosphate URIdylohydrolase	Mitochondria	up4u + H2O -> UTP + UMP	Nucleotide Salvage Pathway	SPCC4G3.02	3.6.1.17
dinucleoside tetraphosphatase	Nucleus	gp4g + H2O <-> GTP + GMP	Nucleotide Salvage Pathway	SPCC4G3.02	3.6.1.17
P1,P4-Bis(5'-nucleosyl)-tetraphosphate nucleotidohydrolase	Nucleus	xp4x + H2O <-> XTP + XMP	Nucleotide Salvage Pathway	SPCC4G3.02	3.6.1.17
P1,P4-bis(5'-URIdyl)-tetraphosphate URIdylohydrolase	Nucleus	up4u + H2O -> UTP + UMP	Nucleotide Salvage Pathway	SPCC4G3.02	3.6.1.17
Inosine ribohydrolase	Cytosol	INS + H2O -> HXAN + rib-D	Nucleotide Salvage Pathway	SPBC1683.06c	3.2.2.1
Xanthosine ribohydrolase	Cytosol	xtsn + H2O -> XAN + rib-D	Nucleotide Salvage Pathway	SPBC1683.06c	3.2.2.1
N-ribosylnicotinamide ribohydrolase	Cytosol	rnam + H2O -> ncam + rib-D	Nucleotide Salvage Pathway	SPBC1683.06c	3.2.2.1
Inosine ribohydrolase	Nucleus	INS + H2O -> HXAN + rib-D	Nucleotide Salvage Pathway	SPBC1683.06c	3.2.2.1
Xanthosine ribohydrolase	Nucleus	xtsn + H2O -> XAN + rib-D	Nucleotide Salvage Pathway	SPBC1683.06c	3.2.2.1
N-ribosylnicotinamide ribohydrolase	Nucleus	rnam + H2O -> ncam + rib-D	Nucleotide Salvage Pathway	SPBC1683.06c	3.2.2.1
nucleoside-diphosphatase (GDP)	Endoplasmic Reticulum	GDP + H2O -> GMP + H + Pi	Nucleotide Salvage Pathway	SPAC824.08	
guanine phosphoribosyltransferase	Endoplasmic Reticulum	GUA + PRPP -> GMP + PPi	Nucleotide Salvage Pathway	SPAC23C11.13c	
hypoxanthine phosphoribosyltransferase (Hypoxanthine)	Endoplasmic Reticulum	HXAN + PRPP -> IMP + PPi	Nucleotide Salvage Pathway	SPAC23C11.13c	
adenine deaminase	Nucleus	ADE + H + H2O -> HXAN + NH4	Nucleotide Salvage Pathway	SPBC1198.02	3.5.4.4
adenine phosphoribosyltransferase	Nucleus	ADE + PRPP -> AMP + PPi	Nucleotide Salvage Pathway	SPAC23A1.03	2.4.2.7
adenosine kinase	Nucleus	ADN + ATP -> ADP + AMP + H	Nucleotide Salvage Pathway	SPCC338.14	2.7.1.20
Adenosine deaminase	Nucleus	ADN + H + H2O -> INS + NH4	Nucleotide Salvage Pathway	SPBC1198.02	3.5.4.4
adenosine hydrolase	Nucleus	ADN + H2O -> ADE + rib-D	Nucleotide Salvage Pathway	SPAC17G8.02	
adenylate kinase	Nucleus	AMP + ATP <-> 2 ADP	Nucleotide Salvage Pathway	SPAC4G9.03	2.7.4.3
adentylate kinase (GTP). nucleus	Nucleus	AMP + GTP <-> ADP + GDP	Nucleotide Salvage Pathway	SPAC4G9.03	2.7.4.3
adentylate kinase (ITP)	Nucleus	AMP + ITP <-> ADP + IDP	Nucleotide Salvage Pathway	SPAC4G9.03	2.7.4.3
dTMP kinase	Nucleus	ATP + dTMP <-> ADP + dTDP	Nucleotide Salvage Pathway	SPCC70.07c	2.7.4.9
5'-nucleotidase (CMP)	Nucleus	CMP + H2O -> cytd + Pi	Nucleotide Salvage Pathway	SPAC24B11.05	
cytidine kinase (GTP)	Nucleus	cytd + GTP -> CMP + GDP + H	Nucleotide Salvage Pathway	SPCC162.11c	2.7.1.48
Deoxyadenosine deaminase	Nucleus	dad-2 + H + H2O -> din + NH4	Nucleotide Salvage Pathway	SPBC1683.02 SPBC1198.02	3.5.4.4
pURIne-nucleoside phosphorylase (Deoxyadenosine)	Nucleus	dad-2 + Pi <-> 2dr1p + ADE	Nucleotide Salvage Pathway	SPAC1805.16c	2.4.2.1
dCTP deaminase	Nucleus	dCTP + H + H2O -> dUTP + NH4	Nucleotide Salvage Pathway	SPBC2G2.13c	3.5.4.12
deoxycytidine deaminase	Nucleus	dcyt + H + H2O -> dURI + NH4	Nucleotide Salvage Pathway	SPAC1556.04c	3.5.4.5
pURIne-nucleoside phosphorylase (Deoxyguanosine)	Nucleus	dGSN + Pi <-> 2dr1p + GUA	Nucleotide Salvage Pathway	SPAC1805.16c	2.4.2.1
pURIne-nucleoside phosphorylase (Deoxyinosine)	Nucleus	din + Pi <-> 2dr1p + HXAN	Nucleotide Salvage Pathway	SPAC1805.16c	2.4.2.1
gnnuc	Nucleus	GSN + H2O -> GUA + rib-D	Nucleotide Salvage Pathway	SPAC17G8.02	
guanine phosphoribosyltransferase	Nucleus	GUA + PRPP -> GMP + PPi	Nucleotide Salvage Pathway	SPAC23C11.13c	
5'-nucleotidase (IMP)	Nucleus	H2O + IMP -> INS + Pi	Nucleotide Salvage Pathway	SPBC30D10.03c	3.1.3.-
5'-nucleotidase (UMP)	Nucleus	H2O + UMP -> Pi + URI	Nucleotide Salvage Pathway	SPAC24B11.05	
hypoxanthine phosphoribosyltransferase (Hypoxanthine)	Nucleus	HXAN + PRPP -> IMP + PPi	Nucleotide Salvage Pathway	SPAC23C11.13c	
pURIne-nucleoside phosphorylase (Inosine)	Nucleus	INS + Pi <-> HXAN + r1p	Nucleotide Salvage Pathway	SPAC1805.16c	2.4.2.1
pURIne-nucleoside phosphorylase (Xanthosine)	Nucleus	Pi + xtsn <-> r1p + XAN	Nucleotide Salvage Pathway	SPAC1805.16c	2.4.2.1
Adenosine deaminase	Cytosol	ADN + H + H2O -> INS + NH4	Nucleotide Salvage Pathway	SPBC1198.02	3.5.4.4
adenine deaminase	Cytosol	ADE + H + H2O -> HXAN + NH4	Nucleotide Salvage Pathway	SPBC1198.02	3.5.4.4
adenylate kinase	Cytosol	AMP + ATP <-> 2 ADP	Nucleotide Salvage Pathway	SPAC4G9.03	2.7.4.3
adenylate kinase	Mitochondria	AMP + ATP <-> 2 ADP	Nucleotide Salvage Pathway	SPAC4G9.03	2.7.4.3
adentylate kinase (GTP)	Cytosol	AMP + GTP <-> ADP + GDP	Nucleotide Salvage Pathway	SPAC4G9.03	2.7.4.3
adentylate kinase (GTP)	Mitochondria	AMP + GTP <-> ADP + GDP	Nucleotide Salvage Pathway	SPAC4G9.03	2.7.4.3
adentylate kinase (ITP)	Cytosol	AMP + ITP <-> ADP + IDP	Nucleotide Salvage Pathway	SPAC4G9.03	2.7.4.3
adentylate kinase (ITP)	Mitochondria	AMP + ITP <-> ADP + IDP	Nucleotide Salvage Pathway	SPAC4G9.03	2.7.4.3
adenosine kinase	Cytosol	ADN + ATP -> ADP + AMP + H	Nucleotide Salvage Pathway	SPCC338.14	2.7.1.20
adenosine hydrolase	Cytosol	ADN + H2O -> ADE + rib-D	Nucleotide Salvage Pathway	SPAC17G8.02	
adenine phosphoribosyltransferase	Cytosol	ADE + PRPP -> AMP + PPi	Nucleotide Salvage Pathway	SPAC23A1.03	2.4.2.7
AMP nucleosidase	Cytosol	AMP + H2O -> ADE + R5P	Nucleotide Salvage Pathway	SPBC106.04	3.5.4.6

ATP maintenance requirement	Cytosol	ATP + H2O -> ADP + H + Pi	Nucleotide Salvage Pathway		
CMP nucleosidase	Cytosol	CMP + H2O -> csn + R5P	Nucleotide Salvage Pathway		
cytidine kinase (GTP)	Cytosol	cytd + GTP -> CMP + GDP + H	Nucleotide Salvage Pathway	SPCC162.11c	2.7.1.48
cytidylate kinase (CMP)	Cytosol	ATP + CMP <-> ADP + CDP	Nucleotide Salvage Pathway		
cytidylate kinase (dCMP)	Cytosol	ATP + dCMP <-> ADP + dCDP	Nucleotide Salvage Pathway		
Deoxyadenosine deaminase	Cytosol	dad-2 + H + H2O -> din + NH4	Nucleotide Salvage Pathway	SPBC1683.02<br>SPBC1198.02	3.5.4.4
deoxyadenylate kinase	Cytosol	ATP + dAMP <-> ADP + dADP	Nucleotide Salvage Pathway		
dCTP deaminase	Cytosol	dCTP + H + H2O -> dUTP + NH4	Nucleotide Salvage Pathway	SPBC2G2.13c	3.5.4.12
deoxycytidine deaminase	Cytosol	dcyt + H + H2O -> dURI + NH4	Nucleotide Salvage Pathway	SPAC1556.04c	3.5.4.5
dTMP kinase	Cytosol	ATP + dTMP <-> ADP + dTDP	Nucleotide Salvage Pathway	SPCC70.07c	2.7.4.9
gnnuc	Cytosol	GSN + H2O -> GUA + rib-D	Nucleotide Salvage Pathway	SPAC17G8.02	
guanosine kinase	Cytosol	ATP + GSN -> ADP + GMP + H	Nucleotide Salvage Pathway		
guanine deaminase	Cytosol	GUA + H + H2O -> NH4 + XAN	Nucleotide Salvage Pathway	SPCC1672.03c	3.5.4.3
guanine phosphoribosyltransferase	Cytosol	GUA + PRPP -> GMP + PPi	Nucleotide Salvage Pathway	SPAC23C11.13c	
hypoxanthine phosphoribosyltransferase (Hypoxanthine)	Cytosol	HXAN + PRPP -> IMP + PPi	Nucleotide Salvage Pathway	SPAC23C11.13c	
insosine kinase	Cytosol	ATP + INS -> ADP + H + IMP	Nucleotide Salvage Pathway		
nucleoside-diphosphatase (GDP)	Golgi apparatus	GDP + H2O -> GMP + H + Pi	Nucleotide Salvage Pathway	SPAC824.08	
nucleoside-diphosphatase (dGDP)	Cytosol	dGDP + H2O -> dGMP + H + Pi	Nucleotide Salvage Pathway		
nucleoside-diphosphate kinase (ATP:GDP)	Cytosol	ATP + GDP <-> ADP + GTP	Nucleotide Salvage Pathway	SPAC806.07	2.7.4.6
nucleoside-diphosphate kinase (ATP:UDP)	Cytosol	ATP + UDP <-> ADP + UTP	Nucleotide Salvage Pathway	SPAC806.07	2.7.4.6
nucleoside-diphosphate kinase (ATP:CDP)	Cytosol	ATP + CDP <-> ADP + CTP	Nucleotide Salvage Pathway	SPAC806.07	2.7.4.6
nucleoside-diphosphate kinase (ATP:dTDP)	Cytosol	ATP + dTDP <-> ADP + dTTP	Nucleotide Salvage Pathway	SPAC806.07	2.7.4.6
nucleoside-diphosphate kinase (ATP:dGDP)	Cytosol	ATP + dGDP <-> ADP + dGTP	Nucleotide Salvage Pathway	SPAC806.07	2.7.4.6
nucleoside-diphosphate kinase (ATP:dUDP)	Cytosol	ATP + dUDP <-> ADP + dUTP	Nucleotide Salvage Pathway	SPAC806.07	2.7.4.6
nucleoside-diphosphate kinase (ATP:dCDP)	Cytosol	ATP + dCDP <-> ADP + dCTP	Nucleotide Salvage Pathway	SPAC806.07	2.7.4.6
nucleoside-diphosphate kinase (ATP:dADP)	Cytosol	ATP + dADP <-> ADP + dATP	Nucleotide Salvage Pathway	SPAC806.07	2.7.4.6
nucleoside-diphosphate kinase (ATP:IDP)	Cytosol	ATP + IDP <-> ADP + ITP	Nucleotide Salvage Pathway	SPAC806.07	2.7.4.6
5'-nucleotidase (dUMP)	Cytosol	dUMP + H2O -> dURI + Pi	Nucleotide Salvage Pathway		
5'-nucleotidase (XMP)	Cytosol	XMP + H2O -> xtsn + Pi	Nucleotide Salvage Pathway		
5'-nucleotidase (IMP)	Cytosol	H2O + IMP -> INS + Pi	Nucleotide Salvage Pathway	SPBC30D10.03c	3.1.3.-
5'-nucleotidase (UMP)	Cytosol	H2O + UMP -> Pi + URI	Nucleotide Salvage Pathway	SPAC24B11.05	
5'-nucleotidase (dCMP)	Cytosol	dCMP + H2O -> dcyt + Pi	Nucleotide Salvage Pathway		
5'-nucleotidase (CMP)	Cytosol	CMP + H2O -> cytd + Pi	Nucleotide Salvage Pathway	SPAC24B11.05	
5'-nucleotidase (dTMP)	Cytosol	dTMP + H2O -> Pi + THYMD	Nucleotide Salvage Pathway		
5'-nucleotidase (dAMP)	Cytosol	dAMP + H2O -> dad-2 + Pi	Nucleotide Salvage Pathway		
5'-nucleotidase (AMP)	Cytosol	AMP + H2O -> ADN + Pi	Nucleotide Salvage Pathway		
5'-nucleotidase (dGMP)	Cytosol	dGMP + H2O -> dGSN + Pi	Nucleotide Salvage Pathway		
5'-nucleotidase (GMP)	Cytosol	GMP + H2O -> GSN + Pi	Nucleotide Salvage Pathway		
nucleoside-triphosphatase (GTP)	Cytosol	GTP + H2O -> GDP + H + Pi	Nucleotide Salvage Pathway		
nucleoside-triphosphatase (dGTP)	Cytosol	dGTP + H2O -> dGDP + H + Pi	Nucleotide Salvage Pathway		
pURIne-nucleoside phosphorylase (Adenosine)	Cytosol	ADN + Pi <-> ADE + r1p	Nucleotide Salvage Pathway	SPAC1805.16c	2.4.2.1
pURIne-nucleoside phosphorylase (Deoxyadenosine)	Cytosol	dad-2 + Pi <-> 2dr1p + ADE	Nucleotide Salvage Pathway	SPAC1805.16c	2.4.2.1
pURIne-nucleoside phosphorylase (Guanosine)	Cytosol	GSN + Pi <-> GUA + r1p	Nucleotide Salvage Pathway	SPAC1805.16c	2.4.2.1
pURIne-nucleoside phosphorylase (Deoxyguanosine)	Cytosol	dGSN + Pi <-> 2dr1p + GUA	Nucleotide Salvage Pathway	SPAC1805.16c	2.4.2.1
pURIne-nucleoside phosphorylase (Inosine)	Cytosol	INS + Pi <-> HXAN + r1p	Nucleotide Salvage Pathway	SPAC1805.16c	2.4.2.1
pURIne-nucleoside phosphorylase (Deoxyinosine)	Cytosol	din + Pi <-> 2dr1p + HXAN	Nucleotide Salvage Pathway	SPAC1805.16c	2.4.2.1
pURIne-nucleoside phosphorylase (Xanthosine)	Cytosol	Pi + xtsn <-> r1p + XAN	Nucleotide Salvage Pathway	SPAC1805.16c	2.4.2.1
pyrimidine-nucleoside phosphorylase (uracil)	Cytosol	Pi + URI <-> r1p + ura	Nucleotide Salvage Pathway		
ribonucleoside-diphosphate reductase (ADP)	Cytosol	ADP + TRDrd -> dADP + H2O + TRDox	Nucleotide Salvage Pathway	SPAC7D4.07c<br>SPBC25D12.04+SPAC1F7.05	
ribonucleoside-diphosphate reductase	Nucleus	ADP + TRDrd -> dADP + H2O + TRDox	Nucleotide Salvage Pathway	SPAC7D4.07c<br>SPBC25D12.04+SPAC1F7.05	
ribonucleoside-diphosphate reductase (GDP)	Cytosol	GDP + TRDrd -> dGDP + H2O + TRDox	Nucleotide Salvage Pathway	SPAC7D4.07c<br>SPBC25D12.04+SPAC1F7.05	
ribonucleoside-diphosphate reductase (GDP)	Nucleus	GDP + TRDrd -> dGDP + H2O + TRDox	Nucleotide Salvage Pathway	SPAC7D4.07c<br>SPBC25D12.04+SPAC1F7.05	
ribonucleoside-diphosphate reductase (CDP)	Cytosol	CDP + TRDrd -> dCDP + H2O + TRDox	Nucleotide Salvage Pathway	SPAC7D4.07c<br>SPBC25D12.04+SPAC1F7.05	
ribonucleoside-diphosphate reductase (CDP)	Nucleus	CDP + TRDrd -> dCDP + H2O + TRDox	Nucleotide Salvage Pathway	SPAC7D4.07c<br>SPBC25D12.04+SPAC1F7.05	
ribonucleoside-diphosphate reductase (UDP)	Cytosol	UDP + TRDrd -> dUDP + H2O + TRDox	Nucleotide Salvage Pathway	SPAC7D4.07c<br>SPBC25D12.04+SPAC1F7.05	
ribonucleoside-diphosphate reductase (UDP)	Nucleus	UDP + TRDrd -> dUDP + H2O + TRDox	Nucleotide Salvage Pathway	SPAC7D4.07c<br>SPBC25D12.04+SPAC1F7.05	
ribonucleoside-triphosphate reductase (ATP)	Cytosol	ATP + TRDrd -> dATP + H2O + TRDox	Nucleotide Salvage Pathway	SPAC7D4.07c	
ribonucleoside-triphosphate reductase (GTP)	Cytosol	GTP + TRDrd -> dGTP + H2O + TRDox	Nucleotide Salvage Pathway	SPAC7D4.07c	
ribonucleoside-triphosphate reductase (CTP)	Cytosol	CTP + TRDrd -> dCTP + H2O + TRDox	Nucleotide Salvage Pathway	SPAC7D4.07c	
ribonucleoside-triphosphate reductase (UTP)	Cytosol	UTP + TRDrd -> dUTP + H2O + TRDox	Nucleotide Salvage Pathway	SPAC7D4.07c	
UMP kinase	Cytosol	ATP + UMP <-> ADP + UDP	Nucleotide Salvage Pathway	SPCC1795.05c	
UMP kinase	Nucleus	ATP + UMP <-> ADP + UDP	Nucleotide Salvage Pathway	SPCC1795.05c	
URIdylate kinase (dUMP)	Cytosol	ATP + dUMP <-> ADP + dUDP	Nucleotide Salvage Pathway	SPCC1795.05c	
URIdylate kinase (dUMP)	Nucleus	ATP + dUMP <-> ADP + dUDP	Nucleotide Salvage Pathway	SPCC1795.05c	
UTP diphosphohydrolase	Cytosol	2 H2O + UTP -> 2 H + 2 Pi + UMP	Nucleotide Salvage Pathway	SPCC11E10.05c	
xanthine phosphoribosyltransferase	Cytosol	PRPP + XAN -> PPi + XMP	Nucleotide Salvage Pathway	SPAC23C11.13c	
ADP-ribose 2,3-cyclic phosphodiesterase	Cytosol	23cAMP + H + H2O -> AMP2P	Other	SPACUNK4.15	
2-deoxyglucose-6-phosphatase	Cytosol	2doxG6P + H2O -> 2dGLC + Pi	Other		
trans-aconitate 3-methyltransferase	Cytosol	acon-T + SAM -> acon5m + SAH	Other	SPAC25B8.09<br>SPAC25B8.10	
HCO3 equilibration reaction	Cytosol	CO2 + H2O <-> H + HCO3	Other		
HCO3 equilibration reaction	Mitochondria	CO2 + H2O <-> H + HCO3	Other		
HCO3 equilibrium reaction	Nucleus	CO2 + H2O <-> H + HCO3	Other		
hydrogen peroxide reductase (thioredoxin)	Cytosol	H2O2 + TRDrd -> 2 H2O + TRDox	Other	SPCC576.03c<br>SPAC7D4.07c	
hydrogen peroxide reductase (thioredoxin)	Mitochondria	H2O2 + TRDrd <-> 2 H2O + TRDox	Other	SPBC12D12.07c	

Thioredoxin	Nucleus	H2O2 + TRDrd -> 2 H2O + TRDox	Other	SPAC7D4.07c SPBC1773.02c	
hydrogen peroxide reductase (thioredoxin)	Peroxisome	H2O2 + TRDrd <-> 2 H2O + TRDox	Other	SPAC7D4.07c SPCC330.06c	
Aminobutyraldehyde dehydrogenase, cytoplasm	Cytosol	4abutn + H2O + NAD -> 4abut + 2 H + NADH	Other Amino Acid Metabolism	SPAC9E9.09c	1.2.1.3
Aminobutyraldehyde dehydrogenase	Golgi apparatus	4abutn + H2O + NAD -> 4abut + 2 H + NADH	Other Amino Acid Metabolism	SPAC9E9.09c	1.2.1.3
Peptide alpha-N-acetyltransferase	Mitochondria	ACCoA + PEPD -> APEP + CoA + H	Other Amino Acid Metabolism	SPBC106.07c	
nitrilase	Mitochondria	acybut + 2 H2O -> GLU + NH4	Other Amino Acid Metabolism	SPAC26A3.11	
nitrilase	Mitochondria	aprop + 2 H2O -> ALA + NH4	Other Amino Acid Metabolism	SPAC26A3.11	
glutathione peridoxase	Nucleus	2 GTHrd + H2O2 <-> GTHox + 2 H2O	Other Amino Acid Metabolism	SPAC4F10.20 SPBC32F12.03c	1.11.1.9
Aminobutyraldehyde dehydrogenase	Nucleus	4abutn + H2O + NAD -> 4abut + 2 H + NADH	Other Amino Acid Metabolism	SPAC9E9.09c	1.2.1.3
nitrilase	Nucleus	acybut + 2 H2O -> GLU + NH4	Other Amino Acid Metabolism	SPBC651.02 SPCC965.09	
nitrilase	Nucleus	aprop + 2 H2O -> ALA + NH4	Other Amino Acid Metabolism	SPBC651.02 SPCC965.09	
gamma-glutamylcysteine synthetase	Nucleus	ATP + CYS + GLU -> ADP + GLUcys + H + Pi	Other Amino Acid Metabolism	SPAC22F3.10c	6.3.2.2
4-acetamidobutyrate deacetylase	Cytosol	4aabutn + H2O -> 4abut + ac	Other Amino Acid Metabolism		
Aminobutyraldehyde dehydrogenase	Mitochondria	4abutn + H2O + NAD -> 4abut + 2 H + NADH	Other Amino Acid Metabolism	SPAC9E9.09c	1.2.1.3
N-acetylputrescine: oxygen oxireductase (deaminating)	Cytosol	aprut + H2O + O2 -> H2O2 + n4abutn + NH4	Other Amino Acid Metabolism		
glycylpeptide N-tetradecanoyltransferase	Cytosol	glp + C140CoA -> CoA + tglp	Other Amino Acid Metabolism	SPBC2G2.11	2.3.1.9
gamma-glutamylcysteine synthetase	Cytosol	ATP + CYS + GLU -> ADP + GLUcys + H + Pi	Other Amino Acid Metabolism	SPAC22F3.10c	6.3.2.2
glutathione oxidoreductase	Cytosol	GTHox + H + NADPH -> 2 GTHrd + NADP	Other Amino Acid Metabolism	SPBC17A3.07 SPAC4F10.20 SPBC26H8.06	1.8.1.7
glutathione peridoxase	Cytosol	2 GTHrd + H2O2 <-> GTHox + 2 H2O	Other Amino Acid Metabolism	SPAC4F10.20 SPBC32F12.03c	1.11.1.9
glutathione synthetase	Cytosol	ATP + GLUcys + GLY -> ADP + GTHrd + H + Pi	Other Amino Acid Metabolism	SPAC3F10.04	
N4-Acetylaminobutanal:NAD+ oxidoreductase	Cytosol	H2O + n4abutn + NAD -> 4aabutn + 2 H + NADH	Other Amino Acid Metabolism		1 .2.1.3
nitrilase	Cytosol	aprop + 2 H2O -> ALA + NH4	Other Amino Acid Metabolism	SPBC651.02 SPCC965.09 SPAC26A3.11	
nitrilase	Cytosol	acybut + 2 H2O -> GLU + NH4	Other Amino Acid Metabolism	SPBC651.02 SPCC965.09 SPAC26A3.11	
ATP synthase	Mitochondria	ADP[m] + 3 H[c] + Pi[m] -> ATP[m] + 2 H[m] + H2O[m]	Oxidative Phosphorylation	SPAC222.12c SPAC14C4.14 SPBC1734.13 SPCC1840.06 SPBC13E7.04 SPBC31F10.15c ScpofMp09 ScpofMp06 SPBC1604.07 ScpofMp08 SPBC29A10.13 SPBC1604.11 SPAC23C4.11 SPAC343.05 SPAC637.05c SPAPB2B4.05 SPCC965.03 SPAC11E3.07 SPBC3B9.18c SPBC1289.05c SPAC16E8.07c SPAC17A2.03c SPAC7D4.10	3.6.3.14 3.6.3.6 3.6.1.1
Cytochrome c peroxidase	Mitochondria	2 focytc + H2O2 -> 2 ficytc + 2 H2O	Oxidative Phosphorylation	SPCC191.07	
cytochrome c oxidase	Mitochondria	4 focytc[m] + 6 H[m] + O2[m] -> 4 ficytc[m] + 6 H[c] + 2 H2O[m]	Oxidative Phosphorylation	SPBC365.02c ScpofMp01 ScpofMp10 SPCC338.10c SPAC1B2.04 SPAC1296.02 SPCC1739.09c SPCC1442.08c SPCC1259.05c SPAC24C9.16c SPAC1420.04c SPAC22E12.10c SPBC26H8.14c	1.9.3.1
ubiquinol-6 cytochrome c reductase	Mitochondria	2 ficytc[m] + 1.5 H[m] + Q6H2[m] -> 2 focytc[m] + 1.5 H[c] + Q6[m]	Oxidative Phosphorylation	SPBC16H5.06 ScpofMp04 SPBC29A3.18 SPCC613.10 SPBC16C6.08c SPCC737.02c SPAC1782.07 SPCC1682.01 SPBP4H10.09	1.10.2.2
formate dehydrogenaseic/mitochondrial	Mitochondria	for[c] + H[c] + Q6[m] -> CO2[c] + Q6H2[m]	Oxidative Phosphorylation		1.2.1.2
fumarate reductase	Mitochondria	FADH2 + FUM -> fad + SUCC	Oxidative Phosphorylation	SPAC17A2.05	
fumarase	Cytosol	FUM + H2O <-> MAL	Oxidative Phosphorylation	SPCC18.18c	4.2.1.2
fumarase	Mitochondria	FUM + H2O <-> MAL	Oxidative Phosphorylation	SPCC18.18c	4.2.1.2
malate dehydrogenase	Cytosol	MAL + NAD <-> H + NADH + OAA	Oxidative Phosphorylation		
malate dehydrogenase	Mitochondria	MAL + NAD <-> H + NADH + OAA	Oxidative Phosphorylation	SPCC306.08c	
malate dehydrogenase	Peroxisome	MAL + NAD <-> H + NADH + OAA	Oxidative Phosphorylation		
NADH dehydrogenaseic/mitochondrial		H[c] + NADH[c] + Q6[m] -> NAD[c] + Q6H2[m]	Oxidative Phosphorylation	SPAC3A11.07 SPBC947.15c	
NADH dehydrogenase	Mitochondria	H + NADH + Q6 -> NAD + Q6H2	Oxidative Phosphorylation	SPAC3A11.07 SPBC947.15c	
inorganic diphosphatase	Cytosol	H2O + PPi -> H + 2 Pi	Oxidative Phosphorylation	SPAC23C11.05 SPAC3A12.02	
succinate dehydrogenase	Mitochondria	fad + SUCC <-> FADH2 + FUM	Oxidative Phosphorylation	SPCC330.12c SPBP23A10.16 SPAC1556.02c SPAC140.01	1.3.5.1
succinate dehydrogenase (ubiquinone-6)	Mitochondria	FADH2 + Q6 <-> fad + Q6H2	Oxidative Phosphorylation		
3-methyl-2-oxobutanoate hydroxymethyltransferase, mitochrondria	Mitochondria	3MOB + H2O + MLTHF -> 2DHP + THF	Pantothenate and CoA Biosynthesis	SPAC5H10.09c	2.1.2.11
CoA hydrolase	Mitochondria	CoA + H2O -> 2 H + pan4p + PAP	Pantothenate and CoA Biosynthesis	SPAC6G9.05	
biotin synthase	Mitochondria	dtbt + s <-> btn + 2 H	Pantothenate and CoA Biosynthesis	SPCC1235.02	2.8.1.6
2-dehydropantoate 2-reductase	Nucleus	2DHP + H + NADPH -> NADP + pant-R	Pantothenate and CoA Biosynthesis	SPBPB2B2.09c	1.1.1.169
pantothenate synthase	Nucleus	ala-B + ATP + pant-R -> AMP + H + pnto-R + PPi	Pantothenate and CoA Biosynthesis	SPAC5H10.08c	6.3.2.1
aldehyde dehydrogenase (3-aminopropanal, NAD)	Nucleus	aproa + H2O + NAD -> ala-B + 2 H + NADH	Pantothenate and CoA Biosynthesis	SPAC922.07c	1.2.1.5
aldehyde dehydrogenase (3-aminopropanal, NAD)	Cytosol	aproa + H2O + NAD -> ala-B + 2 H + NADH	Pantothenate and CoA Biosynthesis	SPAC922.07c	1.2.1.5
biotin-[acetyl-CoA-carboxylase] ligase	Cytosol	ATP + btn + H -> btAMP + PPi	Pantothenate and CoA Biosynthesis	SPBC30D10.07c	6.3.4.9, 6.3.4.10, 6.3.4.11, 6.3.4.15
CoA hydrolase	Peroxisome	CoA + H2O -> 2 H + pan4p + PAP	Pantothenate and CoA Biosynthesis	SPAC6G9.05	
dephospho-CoA kinase	Cytosol	ATP + dpCoA -> ADP + CoA + H	Pantothenate and CoA Biosynthesis		

dephospho-CoA kinase	Mitochondria	ATP + dpCoA -> ADP + CoA + H	Pantothenate and CoA Biosynthesis		
2-dehydropantoate 2-reductase	Cytosol	2DHP + H + NADPH -> NADP + pant-R	Pantothenate and CoA Biosynthesis	SPBPB2B2.09c	1.1.1.169
pantothenate synthase	Cytosol	ala-B + ATP + pant-R -> AMP + H + pnto-R + PPi	Pantothenate and CoA Biosynthesis	SPAC5H10.08c	6.3.2.1
polyamine oxidase	Cytosol	N1aspmd + H2O + O2 -> aproa + aprut + H2O2	Pantothenate and CoA Biosynthesis		
poylamine oxidase	Cytosol	N1sprm + H2O + O2 -> N1aspmd + aproa + H2O2	Pantothenate and CoA Biosynthesis		
poylamine oxidase	Cytosol	H2O + O2 + sprm -> aproa + H2O2 + spmd	Pantothenate and CoA Biosynthesis		
phosphopantothenoylcysteine decarboxylase	Cytosol	4ppcys + H -> CO2 + pan4p	Pantothenate and CoA Biosynthesis		
phosphopantothenate-cysteine ligase	Cytosol	4ppan + CTP + CYS -> 4ppcys + CMP + H + PPi	Pantothenate and CoA Biosynthesis		
pantetheine-phosphate adenylyltransferase	Cytosol	ATP + H + pan4p -> dpCoA + PPi	Pantothenate and CoA Biosynthesis		
panthetheine-phosphate adenylyltransferase	Mitochondria	ATP + H + pan4p -> dpCoA + PPi	Pantothenate and CoA Biosynthesis		
phosphogluconate dehydrogenase	Mitochondria	6pgc + NADP -> CO2 + NADPH + ru5p-D	Pentose Phosphate Pathway	SPBC660.16	1.1.1.44
6-phosphogluconolactonase	Nucleus	6pgl + H2O -> 6pgc + H	Pentose Phosphate Pathway	SPCC16C4.10	3.1.1.31
Deoxyribokinase	Nucleus	ATP + drib -> 2dR5P + ADP + H	Pentose Phosphate Pathway	SPBC16G5.02c	
ribokinase	Nucleus	ATP + rib-D -> ADP + H + R5P	Pentose Phosphate Pathway	SPBC16G5.02c	2.7.1.15
transketolase	Nucleus	E4P + xu5p-D <-> F6P + G3P	Pentose Phosphate Pathway	SPBC2G5.05	2.2.1.1
phosphoglucomutase	Nucleus	g1p <-> G6P	Pentose Phosphate Pathway	SPBC32F12.10<br>SPCC1840.05c	5.4.2.2
phosphopentomutase	Nucleus	r1p <-> R5P	Pentose Phosphate Pathway	SPBC32F12.10<br>SPCC1840.05c	5.4.2.2
transketolase	Nucleus	R5P + xu5p-D <-> G3P + s7p	Pentose Phosphate Pathway	SPBC2G5.05	2.2.1.1
ribulose 5-phosphate 3-ePimerase	Nucleus	ru5p-D <-> xu5p-D	Pentose Phosphate Pathway	SPAC31G5.05c	5.1.3.1
Deoxyribokinase	Cytosol	ATP + drib -> 2dR5P + ADP + H	Pentose Phosphate Pathway	SPBC16G5.02c	
glucose 6-phosphate dehydrogenase	Cytosol	G6P + NADP -> 6pgl + H + NADPH	Pentose Phosphate Pathway	SPCC794.01c<br>SPAC3C7.13c<br>SPAC3A12.18	1.1.1.49
phosphogluconate dehydrogenase	Cytosol	6pgc + NADP -> CO2 + NADPH + ru5p-D	Pentose Phosphate Pathway	SPBC660.16	1.1.1.44
6-phosphogluconolactonase	Cytosol	6pgl + H2O -> 6pgc + H	Pentose Phosphate Pathway	SPCC16C4.10	3.1.1.31
phosphoglucomutase	Cytosol	g1p <-> G6P	Pentose Phosphate Pathway	SPBC32F12.10<br>SPCC1840.05c	5.4.2.2
phosphopentomutase	Cytosol	r1p <-> R5P	Pentose Phosphate Pathway	SPBC32F12.10<br>SPCC1840.05c	5.4.2.2
ribokinase	Cytosol	ATP + rib-D -> ADP + H + R5P	Pentose Phosphate Pathway	SPBC16G5.02c	2.7.1.15
ribulose 5-phosphate 3-ePimerase	Cytosol	ru5p-D <-> xu5p-D	Pentose Phosphate Pathway	SPAC31G5.05c	5.1.3.1
ribose-5-phosphate isomerase	Cytosol	R5P <-> ru5p-D	Pentose Phosphate Pathway	SPAC144.12	5.3.1.6
transaldolase	Cytosol	G3P + s7p <-> E4P + F6P	Pentose Phosphate Pathway	SPCC1020.06c	2.2.1.2
transketolase	Cytosol	R5P + xu5p-D <-> G3P + s7p	Pentose Phosphate Pathway	SPBC2G5.05	2.2.1.1
transketolase	Cytosol	E4P + xu5p-D <-> F6P + G3P	Pentose Phosphate Pathway	SPBC2G5.05	2.2.1.1
acyldihydroxyacetonephosphate reductase	Cytosol	1aGLY3p + H + NADPH -> 1AG3P + NADP	Phospholipid Biosynthesis	SPAC23D3.11	
1-Acyl-glycerol-3-phosphate acyltransferase	Cytosol	0.01 1AG3P + 0.02 C100CoA + 0.06 C120CoA + 0.17 C161CoA + 0.09 C182CoA + 0.24 C181CoA + 0.27 C160CoA + 0.05 C180CoA + 0.1 C140CoA -> CoA + 0.01 pa	Phospholipid Biosynthesis	SPAC1851.02	
CDP-diacylglycerol--serine O-phosphatidyltransferae	Mitochondria	CDPdag + GLYC3p <-> CMP + H + pgp	Phospholipid Biosynthesis	SPBP18G5.02	2.7.8.5
choline phosphate cytididyltransferase	Cytosol	CHOLp + CTP + H -> CDPCHOL + PPi	Phospholipid Biosynthesis	SPCC1827.02c	
Choline kinase	Cytosol	ATP + CHOL -> ADP + CHOLp + H	Phospholipid Biosynthesis	SPAC13G7.12c	
diacylglycerol pyrophosphate phosphatase	Cytosol	H2O + 0.01 pa -> 0.01 12dgr + Pi	Phospholipid Biosynthesis	SPBC409.18	
CDP-Diacylglycerol synthetase	Cytosol	CTP + H + 0.01 pa <-> 0.01 CDPdag + PPi	Phospholipid Biosynthesis	SPBC13A2.03	
CDP-Diacylglycerol synthetase	Mitochondria	CTP + H + 0.01 pa <-> 0.01 CDPdag + PPi	Phospholipid Biosynthesis	SPBC13A2.03	
Ethanolamine kinase	Cytosol	ATP + etHa -> ADP + etHAMP + H	Phospholipid Biosynthesis	SPAC13G7.12c	
Ethanolaminephosphotransferase	Cytosol	0.01 12dgr + CDPEA <-> CMP + H + 0.01 pe	Phospholipid Biosynthesis	SPAC22A12.10	
glycerol 3-phosphate acyltransferase (glycerol 3-phosphate)	Cytosol	0.02 C100CoA + 0.06 C120CoA + GLYC3p + 0.17 C161CoA + 0.09 C182CoA + 0.24 C181CoA + 0.27 C160CoA + 0.05 C180CoA + 0.1 C140CoA -> 0.01 1AG3P + CoA	Phospholipid Biosynthesis	SPBC1718.04	
glycerol 3-phosphate acyltransferase (glycerone phosphate)	Cytosol	0.02 C100CoA + 0.06 C120CoA + DHAP + 0.17 C161CoA + 0.09 C182CoA + 0.24 C181CoA + 0.27 C160CoA + 0.05 C180CoA + 0.1 C140CoA -> 0.01 1aGLY3p + CoA	Phospholipid Biosynthesis	SPBC1718.04	
Lyso-phosphatidylcholine acyltransferase acyltransferase	Cytosol	0.01 1agpc + 0.02 C100CoA + 0.06 C120CoA + 0.17 C161CoA + 0.09 C182CoA + 0.24 C181CoA + 0.27 C160CoA + 0.05 C180CoA + 0.1 C140CoA -> CoA + 0.01 pc	Phospholipid Biosynthesis		
liPid phosphate phosphatase	Cytosol	0.01 dagpy + H2O -> H + 0.01 pa + Pi	Phospholipid Biosynthesis	SPBC409.18	
methylene-fatty-acyl-phospholiPid synthase	Cytosol	SAM + 0.01 ptdmeeta -> SAH + H + 0.01 ptd2meeta	Phospholipid Biosynthesis	SPBC337.16	
inositol-1,3,4,5,6-pentakisphosphate 2-kinase	Nucleus	ATP + mi13456p -> ADP + H + minoHp	Phospholipid Biosynthesis	SPCC4B3.10c	
inositol-1,3,4,5-triphosphate 6-kinase	Nucleus	ATP + mi1345p -> ADP + H + mi13456p	Phospholipid Biosynthesis	SPAC607.04	
inositol-1,4,5,6- tetrakisphosphate 3-kinase	Nucleus	ATP + mi1456p -> ADP + H + mi13456p	Phospholipid Biosynthesis	SPAC607.04	
inositol-1,4,5-triphosphate 6-kinase	Nucleus	ATP + mi145p -> ADP + H + mi1456p	Phospholipid Biosynthesis	SPAC607.04	
inositol-1,4,5-trisphosphate 3-kinase	Nucleus	ATP + mi145p -> ADP + H + mi1345p	Phospholipid Biosynthesis	SPAC607.04	
myo-inositol 1-phosphatase	Cytosol	H2O + mi1p-D -> inost + Pi	Phospholipid Biosynthesis		
myo-Inositol-1-phosphate synthase	Cytosol	G6P -> mi1p-D	Phospholipid Biosynthesis		
phosphatidate kinase	Cytosol	ATP + 0.01 pa -> ADP + 0.01 dagpy	Phospholipid Biosynthesis		
phosphoethanolamine cytidyltransferase	Cytosol	CTP + etHAMP + H -> CDPEA + PPi	Phospholipid Biosynthesis	SPAC15E1.05c	
phosphatidylethanolamine N-methyltransferase Cho2	Cytosol	SAM + 0.01 pe -> SAH + H + 0.01 ptdmeeta	Phospholipid Biosynthesis	SPBC26H8.03	
phosphatidylglycerol phosphate phosphatase A	Mitochondria	H2O + pgp -> pg + Pi	Phospholipid Biosynthesis		3.1.3.27
	Mitochondria	pg + CDPdag -> cl + CMP	Phospholipid Biosynthesis		
1-phosphatidylinositol-3,5-bisphosphate 5-phosphatase	Cytosol	H2O + 0.01 ptd135bp -> Pi + 0.01 ptd3ino	Phospholipid Biosynthesis	SPAC3C7.01c<br>SPBC19F5.03<br>SPAC9G1.10c	2.7.1.150
phosphatidylinositol-3-phosphate 4-kinase	Cytosol	ATP + 0.01 ptd3ino -> ADP + H + 0.01 ptd134bp	Phospholipid Biosynthesis		
phosphatidylinositol-3-phosphate 5-kinase	Cytosol	ATP + 0.01 ptd3ino -> ADP + H + 0.01 ptd135bp	Phospholipid Biosynthesis	SPBC3E7.01	
1-phosphatidylinositol-4,5-bisphosphate 5-phosphatase	Cytosol	H2O + 0.01 ptd145bp -> Pi + 0.01 ptd4ino	Phospholipid Biosynthesis	SPBC2G2.02<br>SPBC577.13	2.7.1.68
1-phosphatidylinositol-4,5-bisphosphate phosphodiesterase	Cytosol	H2O + 0.01 ptd145bp -> 0.01 12dgr + H + mi145p	Phospholipid Biosynthesis	SPAC22F8.11	
phosphatidylinositol-4-phosphate 5-kinase	Cytosol	ATP + 0.01 ptd4ino -> ADP + H + 0.01 ptd145bp	Phospholipid Biosynthesis	SPAC19G12.14	
1-phosphatidylinositol 3-kinase	Cytosol	ATP + 0.01 ptd1ino -> ADP + H + 0.01 ptd3ino	Phospholipid Biosynthesis	SPBC216.07c<br>SPBC30D10.10C	
phosphatidylinositol 4-kinase	Cytosol	ATP + 0.01 ptd1ino -> ADP + H + 0.01 ptd4ino	Phospholipid Biosynthesis	SPAC22E12.16c<br>SPBC577.06c	
phosphatidylinositol 4-kinase	Nucleus	ATP + 0.01 ptd1ino -> ADP + H + 0.01 ptd4ino	Phospholipid Biosynthesis	SPAC22E12.16c	
phosphatidylinositol synthase	Cytosol	0.01 CDPdag + inost -> CMP + H + 0.01 ptd1ino	Phospholipid Biosynthesis		
Phosphatidyl-N-methylethanolamine N-methyltransferase	Cytosol	SAM + 0.01 ptd2meeta -> SAH + H + 0.01 pc	Phospholipid Biosynthesis	SPBC337.16	
phosphatidylserine decarboxylase	Golgi apparatus	H + 0.01 ps -> CO2 + 0.01 pe	Phospholipid Biosynthesis	SPAC25B8.03<br>SPBC16E9.18<br>SPAC31G5.15	
phosphatidylserine decarboxylase	Mitochondria	H + 0.01 ps -> CO2 + 0.01 pe	Phospholipid Biosynthesis	SPAC25B8.03<br>SPBC16E9.18<br>SPAC31G5.15	

phosphatidylserine decarboxylase	Vacuole	H + 0.01 ps -> CO2 + 0.01 pe	Phospholipid Biosynthesis	SPAC25B8.03<br>SPBC16E9.18<br>SPAC31G5.15	
phosphatidylserine synthase	Cytosol	0.01 CDPdag + SER <-> CMP + H + 0.01 ps	Phospholipid Biosynthesis	SPCC1442.12	
phosphatidylserine synthase	Mitochondria	0.01 CDPdag + SER <-> CMP + H + 0.01 ps	Phospholipid Biosynthesis	SPCC1442.12	
phospholipase B (phosphatidylinositol) (extracellular)	Extracellular	H2O + 0.005 ptd1ino -> 0.02 C100 + 0.06 C120 + 0.5 G3Pi + H + 0.27 C160 + 0.17 C161 + 0.05 C180 + 0.24 C181 + 0.09 C182 + 0.1 C140	Phospholipid Metabolism	SPAC1786.02<br>SPAC977.09C<br>SPCC1450.09C<br>SPAC1A6.03C<br>SPBC1348.10C<br>SPAC1A6.04C	
phospholipase B (phosphatidylcholine)	Cytosol	H2O + 0.005 pc -> 0.02 C100 + 0.06 C120 + 0.5 G3Pc + H + 0.27 C160 + 0.17 C161 + 0.05 C180 + 0.24 C181 + 0.09 C182 + 0.1 C140	Phospholipid Metabolism	SPAC1786.02<br>SPAC977.09C<br>SPCC1450.09C<br>SPAC1A6.03C<br>SPBC1348.10C<br>SPAC1A6.04C	
phospholipase B (phosphatidylcholine) (extracellular)	Extracellular	H2O + 0.005 pc -> 0.02 C100 + 0.06 C120 + 0.5 G3Pc + H + 0.27 C160 + 0.17 C161 + 0.05 C180 + 0.24 C181 + 0.09 C182 + 0.1 C140	Phospholipid Metabolism	SPAC1786.02<br>SPAC977.09C<br>SPCC1450.09C<br>SPAC1A6.03C<br>SPBC1348.10C<br>SPAC1A6.04C	
phospholipase D	Cytosol	H2O + 0.01 pc -> CHOL + H + 0.01 pa	Phospholipid Metabolism	SPAC2F7.16c	
5-diphosphoinositol-1,2,3,4,6-pentakisphosphate diphosphohydrolase	Cytosol	H2O + ppmi12346p -> H + minoHp + Pi	Phospholipid Metabolism	SPAC13G6.14	
5-diphosphoinositol-1,2,3,4,6-pentakisphosphate synthase	Cytosol	ATP + minoHp -> ADP + ppmi12346p	Phospholipid Metabolism	SPCC970.08	
diphosphoinositol-1,3,4,6-tetrakisphosphate diphosphohydrolase	Cytosol	H2O + ppmi1346p -> H + mi13456p + Pi	Phospholipid Metabolism	SPAC13G6.14	
diphosphoinositol-1,3,4,6-tetrakisphosphate synthase	Cytosol	ATP + mi13456p -> ADP + ppmi1346p	Phospholipid Metabolism	SPCC970.08	
protoporphyrinogen oxidase	Cytosol	3 O2 + 2 pppg9 -> 6 H2O + 2 ppp9	Porphyrin and Chlorophyll Metabolism	SPAC1F5.07c	1.3.3.4
protoporphyrinogen oxidase	Nucleus	3 O2 + 2 pppg9 -> 6 H2O + 2 ppp9	Porphyrin and Chlorophyll Metabolism	SPAC1F5.07c	1.3.3.4
coproporphyrinogen oxidase (O2 required)	Mitochondria	cpppg3 + 2 H + O2 -> 2 CO2 + 2 H2O + pppg9	Porphyrin and Chlorophyll Metabolism	SPAC222.11	1.3.3.3
porphobilinogen synthase	Nucleus	2 5aop -> H + 2 H2O + ppbng	Porphyrin and Chlorophyll Metabolism	SPAC1805.06c	4.2.1.24
uroporphyrinogen decarboxylase (uroporphyrinogen III)	Nucleus	4 H + uppg3 -> 4 CO2 + cpppg3	Porphyrin and Chlorophyll Metabolism	SPCC4B3.05c	4.1.1.37
coproporphyrinogen oxidase (O2 required)	Nucleus	cpppg3 + 2 H + O2 -> 2 CO2 + 2 H2O + pppg9	Porphyrin and Chlorophyll Metabolism	SPAC222.11	1.3.3.3
sirohydrochlorin dehydrogenase	Nucleus	dscl + NADP -> H + NADPH + scl	Porphyrin and Chlorophyll Metabolism	SPAC4D7.06c	1.3.1.76, 4.99.1.4
sirohydrochlorin ferrochetalase	Nucleus	fe2 + scl -> 3 H + sHEME	Porphyrin and Chlorophyll Metabolism	SPAC4D7.06c	1.3.1.76, 4.99.1.4
hydroxymethylbilane synthase	Nucleus	H2O + 4 ppbng -> HMBIL + 4 NH4	Porphyrin and Chlorophyll Metabolism	SPAC24B11.13	2.5.1.61
uroporphyrinogen-III synthase	Nucleus	HMBIL -> H2O + uppg3	Porphyrin and Chlorophyll Metabolism	SPAC31G5.08	4.2.1.75
5-aminolevulinate synthase	Mitochondria	GLY + H + SUCCoA -> 5aop + CO2 + CoA	Porphyrin and Chlorophyll Metabolism	SPAC2F3.09	2.3.1.37
coproporphyrinogen oxidase (O2 required)	Cytosol	cpppg3 + 2 H + O2 -> 2 CO2 + 2 H2O + pppg9	Porphyrin and Chlorophyll Metabolism	SPAC222.11	1.3.3.3
Ferrochelatase	Cytosol	fe2 + ppp9 -> 2 H + pHEME	Porphyrin and Chlorophyll Metabolism	SPCC320.09	4.99.1.1
Ferrochelatase	Nucleus	fe2 + ppp9 -> 2 H + pHEME	Porphyrin and Chlorophyll Metabolism	SPCC320.09	4.99.1.1
Ferrochelatase	Mitochondria	fe2 + ppp9 -> 2 H + pHEME	Porphyrin and Chlorophyll Metabolism	SPCC320.09	4.99.1.1
Heme O monooxygenase	Mitochondria	hemeO + NADH + O2 -> H2O + hemeA + NAD	Porphyrin and Chlorophyll Metabolism	SPAC22E12.10c<br>SPBC3B8.01c	
Heme O synthase	Mitochondria	FRPP + H2O + pHEME -> hemeO + PPi	Porphyrin and Chlorophyll Metabolism	SPBC365.02c	2.5.1.-
hydroxymethylbilane synthase	Cytosol	H2O + 4 ppbng -> HMBIL + 4 NH4	Porphyrin and Chlorophyll Metabolism	SPAC24B11.13	2.5.1.61
porphobilinogen synthase	Cytosol	2 5aop -> H + 2 H2O + ppbng	Porphyrin and Chlorophyll Metabolism	SPAC1805.06c	4.2.1.24
protoporphyrinogen oxidase	Mitochondria	3 O2 + 2 pppg9 -> 6 H2O + 2 ppp9	Porphyrin and Chlorophyll Metabolism	SPAC1F5.07c	1.3.3.4
sirohydrochlorin dehydrogenase	Cytosol	dscl + NADP -> H + NADPH + scl	Porphyrin and Chlorophyll Metabolism	SPAC4D7.06c	1.3.1.76, 4.99.1.4
sirohydrochlorin ferrochetalase	Cytosol	fe2 + scl -> 3 H + sHEME	Porphyrin and Chlorophyll Metabolism	SPAC4D7.06c	1.3.1.76, 4.99.1.4
uroporphyrinogen methyltransferase	Cytosol	2 SAM + uppg3 -> 2 SAH + dscl + H	Porphyrin and Chlorophyll Metabolism	SPCC1739.06c	
uroporphyrinogen-III synthase	Cytosol	HMBIL -> H2O + uppg3	Porphyrin and Chlorophyll Metabolism	SPAC31G5.08	4.2.1.75
uroporphyrinogen decarboxylase (uroporphyrinogen III)	Cytosol	4 H + uppg3 -> 4 CO2 + cpppg3	Porphyrin and Chlorophyll Metabolism	SPCC4B3.05c	4.1.1.37
phosphoribosylformylglycinamidine synthase	Golgi apparatus	ATP + fgar + GLN + H2O -> ADP + fgam + GLU + H + Pi	Purine and Pyrimidine Biosynthesis	SPAC6F12.10c	6.3.5.3
Ap4A hydrolase	Mitochondria	ap4a + H2O <-> 2 ADP + 2 H	Purine and Pyrimidine Biosynthesis	SPCC4G3.02	3.6.1.17
Fumarate dependent DHORD	Mitochondria	DHOR-S + FUM -> orot + SUCC	Purine and Pyrimidine Biosynthesis	SPAC57A10.12c	1.3.3.1
dihydoorotic acid dehydrogenase	Mitochondria	DHOR-S + O2 -> H2O2 + orot	Purine and Pyrimidine Biosynthesis	SPAC57A10.12c	1.3.3.1
yUMP synthetase	Mitochondria	R5P + ura <-> H2O + psd5p	Purine and Pyrimidine Biosynthesis	SPCC126.03<br>SPBC11C11.10	5.4.99.12
adenylosuccinate lyase	Nucleus	SAICAR <-> aicar + FUM	Purine and Pyrimidine Biosynthesis	SPBC14F5.09c	4.3.2.2
phosphoribosylaminoimidazolesuccinocarboxamide synthase	Nucleus	CAIR + ASP + ATP <-> SAICAR + ADP + H + Pi	Purine and Pyrimidine Biosynthesis	SPBC409.10	6.3.2.6
Ap4A hydrolase	Nucleus	ap4a + H2O <-> 2 ADP + 2 H	Purine and Pyrimidine Biosynthesis	SPCC4G3.02	3.6.1.17
adenylosuccinate synthase	Nucleus	ASP + GTP + IMP -> dcAMP + GDP + 2 H + Pi	Purine and Pyrimidine Biosynthesis	SPAC144.03	6.3.4.4
deoxyguanylate kinase (dGMP:ATP)	Nucleus	ATP + dGMP <-> ADP + dGDP	Purine and Pyrimidine Biosynthesis	SPBC1198.05	2.7.4.8
guanylate kinase (GMP:ATP)	Nucleus	ATP + GMP <-> ADP + GDP	Purine and Pyrimidine Biosynthesis	SPBC1198.05	2.7.4.8
URIdine kinase (ATP:URIdine)	Nucleus	ATP + URI -> ADP + H + UMP	Purine and Pyrimidine Biosynthesis	SPCC162.11c	2.7.1.48
3',5'-cyclic-nucleotide phosphodiesterase	Nucleus	cAMP + H2O -> AMP + H	Purine and Pyrimidine Biosynthesis	SPCC285.09c	3.1.4.17
cytidine deaminase	Nucleus	cytd + H + H2O -> NH4 + URI	Purine and Pyrimidine Biosynthesis	SPAC1556.04c	3.5.4.5
guanylate kinase (GMP:dATP)	Nucleus	dATP + GMP <-> dADP + GDP	Purine and Pyrimidine Biosynthesis	SPBC1198.05	2.7.4.8
adenylsuccinate lyase	Nucleus	dcAMP <-> AMP + FUM	Purine and Pyrimidine Biosynthesis	SPBC14F5.09c	4.3.2.2
dCMP deaminase	Nucleus	dCMP + H + H2O <-> dUMP + NH4	Purine and Pyrimidine Biosynthesis	SPBC2G2.13c	3.5.4.12
deoxyURIdine phosphorylase	Nucleus	dURI + Pi <-> 2dr1p + ura	Purine and Pyrimidine Biosynthesis	SPAC1805.16c	2.4.2.1
dUTP diphosphatase	Nucleus	dUTP + H2O -> dUMP + H + PPi	Purine and Pyrimidine Biosynthesis	SPAC644.05c	3.6.1.23
URIdine kinase (GTP:URIdine)	Nucleus	GTP + URI -> GDP + H + UMP	Purine and Pyrimidine Biosynthesis	SPCC162.11c	2.7.1.48
thioredoxin reductase (NADPH)	Nucleus	H + NADPH + TRDox -> NADP + TRDrd	Purine and Pyrimidine Biosynthesis	SPBC3F6.03<br>SPAC7D4.07c	1.8.1.9
orotidine-5'-phosphate decarboxylase	Nucleus	H + orot5p -> CO2 + UMP	Purine and Pyrimidine Biosynthesis	SPCC330.05c	4.1.1.23
orotate phosphoribosyltransferase	Nucleus	orot5p + PPi <-> orot + PRPP	Purine and Pyrimidine Biosynthesis	SPBC725.15	2.4.2.10
thymidine phosphorylase	Nucleus	Pi + THYMD <-> 2dr1p + THYM	Purine and Pyrimidine Biosynthesis	SPAC1805.16c	2.4.2.1
uracil phosphoribosyltransferase	Nucleus	PRPP + ura -> PPi + UMP	Purine and Pyrimidine Biosynthesis	SPAC1002.17c<br>SPAC1399.04c<br>SPAC1B3.01c<br>SPCC16C4.06c	2.4.2.9
yUMP synthetase	Nucleus	R5P + ura <-> H2O + psd5p	Purine and Pyrimidine Biosynthesis	SPCC126.03<br>SPBC11C11.10	5.4.99.12
adenylate cyclase	Cytosol	ATP -> cAMP + PPi	Purine and Pyrimidine Biosynthesis	SPBC19C7.03	4.6.1.1
adenylsuccinate lyase	Cytosol	dcAMP <-> AMP + FUM	Purine and Pyrimidine Biosynthesis	SPBC14F5.09c	4.3.2.2
adenylosuccinate lyase	Cytosol	SAICAR <-> aicar + FUM	Purine and Pyrimidine Biosynthesis	SPBC14F5.09c	4.3.2.2
adenylosuccinate synthase	Cytosol	ASP + GTP + IMP -> dcAMP + GDP + 2 H + Pi	Purine and Pyrimidine Biosynthesis	SPAC144.03	6.3.4.4

phosphoribosylaminoimidazolecarboxamide formyltransferase	Cytosol	10FTHF + aicar <-> fprica + THF	Purine and Pyrimidine Biosynthesis	SPCPB16A4.03c	2.1.2.3, 3.5.4.10
phosphoribosylaminoimidazole carboxylase	Cytosol	air + CO2 <-> CAIR + H	Purine and Pyrimidine Biosynthesis	SPCC1322.13	4.1.1.21
Adenosine monophosphate deaminase	Cytosol	AMP + H + H2O -> IMP + NH4	Purine and Pyrimidine Biosynthesis	SPBC106.04	3.5.4.6
Ap4A hydrolase	Cytosol	ap4a + H2O <-> 2 ADP + 2 H	Purine and Pyrimidine Biosynthesis	SPCC4G3.02	3.6.1.17
aspartate carbamoyltransferase	Cytosol	ASP + cbp -> cbasp + H + Pi	Purine and Pyrimidine Biosynthesis	SPAC22G7.06c SPAC16.03c SPBC56F2.09c	6.3.5.5, 2.1.3.2
ATP adenylyltransferase	Cytosol	ADP + ATP + H -> ap4a + Pi	Purine and Pyrimidine Biosynthesis		
CTP synthase (NH3)	Cytosol	ATP + NH4 + UTP -> ADP + CTP + 2 H + Pi	Purine and Pyrimidine Biosynthesis	SPAC10F6.03c	6.3.4.2
CTP synthase (glutamine)	Cytosol	ATP + GLN + H2O + UTP -> ADP + CTP + GLU + 2 H + Pi	Purine and Pyrimidine Biosynthesis	SPAC10F6.03c	6.3.4.2
cytidine deaminase	Cytosol	cytd + H + H2O -> NH4 + URI	Purine and Pyrimidine Biosynthesis	SPAC1556.04c	3.5.4.5
dCMP deaminase	Cytosol	dCMP + H + H2O <-> dUMP + NH4	Purine and Pyrimidine Biosynthesis	SPBC2G2.13c	3.5.4.12
deoxyguanylate kinase (dGMP:ATP)	Cytosol	ATP + dGMP <-> ADP + dGDP	Purine and Pyrimidine Biosynthesis	SPBC1198.05	2.7.4.8
dihydoorotic acid (ubiquinone-6)		DHOR-S[c] + Q6[m] -> orot[c] + Q6H2[m]	Purine and Pyrimidine Biosynthesis	SPAC57A10.12c	1.3.3.1
dihydroorotase	Cytosol	DHOR-S + H2O <-> cbasp + H	Purine and Pyrimidine Biosynthesis	SPAC22G7.06c SPAC16.03c	6.3.5.5, 2.1.3.2, 3.5.2.3
deoxyURIdine kinase (ATP:DeoxyURIdine)	Cytosol	ATP + dURI -> ADP + dUMP + H	Purine and Pyrimidine Biosynthesis		
deoxyURIdine phosphorylase	Cytosol	dURI + Pi <-> 2dr1p + ura	Purine and Pyrimidine Biosynthesis	SPAC1805.16c	2.4.2.1
dUTP diphosphatase	Cytosol	dUTP + H2O -> dUMP + H + PPi	Purine and Pyrimidine Biosynthesis	SPAC644.05c	3.6.1.23
guanylate kinase (GMP:ATP)	Cytosol	ATP + GMP <-> ADP + GDP	Purine and Pyrimidine Biosynthesis	SPBC1198.05	2.7.4.8
guanylate kinase (GMP:dATP)	Cytosol	dATP + GMP <-> dADP + GDP	Purine and Pyrimidine Biosynthesis	SPBC1198.05	2.7.4.8
glutamine phosphoribosyldiphosphate amidotransferase	Cytosol	GLN + H2O + PRPP -> GLU + PPi + pram	Purine and Pyrimidine Biosynthesis	SPAC4D7.08c	2.4.2.14
GMP synthase	Cytosol	ATP + GLN + H2O + XMP -> AMP + GLU + GMP + 2 H + PPi	Purine and Pyrimidine Biosynthesis	SPAP7G5.02c	6.3.5.2
IMP cyclohydrolase	Cytosol	H2O + IMP <-> fprica	Purine and Pyrimidine Biosynthesis	SPCPB16A4.03c	2.1.2.3, 3.5.4.10
IMP dehydrogenase	Cytosol	H2O + IMP + NAD -> H + NADH + XMP	Purine and Pyrimidine Biosynthesis	SPBC2F12.14c	1.1.1.205
orotidine-5'-phosphate decarboxylase	Cytosol	H + orot5p -> CO2 + UMP	Purine and Pyrimidine Biosynthesis	SPCC330.05c	4.1.1.23
orotate phosphoribosyltransferase	Cytosol	orot5p + PPi <-> orot + PRPP	Purine and Pyrimidine Biosynthesis	SPBC725.15	2.4.2.10
3',5'-cyclic-nucleotide phosphodiesterase	Cytosol	cAMP + H2O -> AMP + H	Purine and Pyrimidine Biosynthesis	SPCC285.09c	3.1.4.17
3',5'-cyclic-nucleotide phosphodiesterase	Cytosol	35cdAMP + H2O -> dAMP + H	Purine and Pyrimidine Biosynthesis	SPCC285.09c	3.1.4.17
3',5'-cyclic-nucleotide phosphodiesterase	Cytosol	35cIMP + H2O -> H + IMP	Purine and Pyrimidine Biosynthesis	SPCC285.09c	3.1.4.17
3',5'-cyclic-nucleotide phosphodiesterase	Cytosol	35cGMP + H2O -> GMP + H	Purine and Pyrimidine Biosynthesis	SPCC285.09c	3.1.4.17
3',5'-cyclic-nucleotide phosphodiesterase	Cytosol	35cCMP + H2O -> CMP + H	Purine and Pyrimidine Biosynthesis	SPCC285.09c	3.1.4.17
phosphoribosylglycinamide synthase	Cytosol	ATP + GLY + pram <-> ADP + gar + H + Pi	Purine and Pyrimidine Biosynthesis	SPBC405.01	6.3.4.13, 6.3.3.1
phosphoribosylaminoimidazole synthase	Cytosol	ATP + fgam -> ADP + air + 2 H + Pi	Purine and Pyrimidine Biosynthesis	SPBC405.01	6.3.4.13, 6.3.3.1
phosphoribosylaminoimidazolesuccinocarboxamide synthase	Cytosol	CAIR + ASP + ATP <-> SAICAR + ADP + H + Pi	Purine and Pyrimidine Biosynthesis	SPBC409.10	6.3.2.6
phosphoribosylformylglycinamidine synthase	Cytosol	ATP + fgar + GLN + H2O -> ADP + fgam + GLU + H + Pi	Purine and Pyrimidine Biosynthesis	SPAC6F12.10c	6.3.5.3
thymidine kinase (ATP:thymidine)	Cytosol	ATP + THYMD -> ADP + dTMP + H	Purine and Pyrimidine Biosynthesis		
thymidine phosphorylase	Cytosol	Pi + THYMD <-> 2dr1p + THYM	Purine and Pyrimidine Biosynthesis	SPAC1805.16c	2.4.2.1
thymidylate synthase	Cytosol	dUMP + MLTHF -> DHF + dTMP	Purine and Pyrimidine Biosynthesis	SPAC15E1.04	2.1.1.45
thioredoxin reductase (NADPH)	Cytosol	H + NADPH + TRDox -> NADP + TRDrd	Purine and Pyrimidine Biosynthesis	SPBC3F6.03 SPAC7D4.07c	1.8.1.9
thioredoxin reductase (NADPH)	Mitochondria	H + NADPH + TRDox -> NADP + TRDrd	Purine and Pyrimidine Biosynthesis	SPBC3F6.03 SPBC12D12.07c	1.8.1.9
uracil phosphoribosyltransferase	Cytosol	PRPP + ura -> PPi + UMP	Purine and Pyrimidine Biosynthesis	SPAC1002.17c SPAC1399.04c SPAC1B3.01c	2.4.2.9
URIdine kinase (ATP:URIdine)	Cytosol	ATP + URI -> ADP + H + UMP	Purine and Pyrimidine Biosynthesis	SPCC162.11c	2.7.1.48
URIdine kinase (GTP:URIdine)	Cytosol	GTP + URI -> GDP + H + UMP	Purine and Pyrimidine Biosynthesis	SPCC162.11c	2.7.1.48
yUMP synthetase	Cytosol	R5P + ura <-> H2O + psd5p	Purine and Pyrimidine Biosynthesis	SPCC16C4.06c	5.4.99.12
hypothetical enyme	Cytosol	H2O + pyam5p -> Pi + pydam	Pyridoxine Metabolism		
O-Phospho-4-hydroxy-L-threonine:2-oxoglutarate aminotransferase	Cytosol	GLU + oHpb <-> AKG + PHTHR	Pyridoxine Metabolism	SPAC1F12.07	2.6.1.52
pyridoxine 5'-phosphate oxidase	Cytosol	O2 + pdx5p <-> H2O2 + pydx5p	Pyridoxine Metabolism	SPAC1093.02	1.4.3.5
pyridoxamine 5'-phosphate oxidase	Cytosol	H2O + O2 + pyam5p -> H2O2 + NH4 + pydx5p	Pyridoxine Metabolism	SPAC1093.02	1.4.3.5
pyridoxamine kinase	Cytosol	ATP + pydam -> ADP + H + pyam5p	Pyridoxine Metabolism		2.7.1.35
pyridoxal kinase	Cytosol	ATP + pydx -> ADP + H + pydx5p	Pyridoxine Metabolism		
pyridoxine kinase	Cytosol	ATP + pydxn -> ADP + H + pdx5p	Pyridoxine Metabolism		2.7.1.35
pyridoxine oxidase	Cytosol	O2 + pydxn <-> H2O2 + pydx	Pyridoxine Metabolism	SPAC1093.02	1.4.3.5
pyridoxal oxidase	Cytosol	2 H2O + NH4 + 0.5 O2 + pydx <-> 2 H2O2 + pydam	Pyridoxine Metabolism	SPAC1093.02	1.4.3.5
D-lactate dehydrogenase	Cytosol	2 ficytc + dLAC -> 2 focytc + PYR	Pyruvate Metabolism	SPCC191.07	
D-lactate dehydrogenase	Mitochondria	2 ficytc + dLAC -> 2 focytc + PYR	Pyruvate Metabolism	SPCC191.07	
D-lactate dehydrogenase	Nucleus	2 ficytc + dLAC -> 2 focytc + PYR	Pyruvate Metabolism	SPCC191.07	
aldehyde dehydrogenase (acetylaldehyde, NAD)	Cytosol	ACAL + H2O + NAD -> AC + 2 H + NADH	Pyruvate Metabolism	SPAC9E9.09c	1.2.1.3
homocitrate synthase	Cytosol	ACCoA + AKG + H2O -> CoA + H + HCIT	Pyruvate Metabolism	SPBC1105.02c	2.3.3.14
acetyl-CoA hydrolase, cytoplasm	Cytosol	ACCoA + H2O -> AC + CoA + H	Pyruvate Metabolism	SPAC1952.09c	3.1.2.1
aldehyde dehydrogenase (acetylaldehyde, NAD)	Golgi apparatus	ACAL + H2O + NAD -> AC + 2 H + NADH	Pyruvate Metabolism	SPAC9E9.09c	1.2.1.3
aldehyde dehydrogenase (acetylaldehyde, NADP)	Golgi apparatus	ACAL + H2O + NADP -> AC + 2 H + NADPH	Pyruvate Metabolism	SPAC9E9.09c	1.2.1.3
alcohol dehydrogenase (ethanol)	Mitochondria	ETOH + NAD <-> ACAL + H + NADH	Pyruvate Metabolism	SPCC13B11.04c SPBC1539.07c	1.1.1.284
formaldehyde dehydrogenase	Mitochondria	fald + GTHrd + NAD <-> SfGLUttH + H + NADH	Pyruvate Metabolism	SPCC13B11.04c SPBC1539.07c	1.1.1.284
aldehyde dehydrogenase (acetylaldehyde, NAD)	Nucleus	ACAL + H2O + NAD -> AC + 2 H + NADH	Pyruvate Metabolism	SPAC9E9.09c	1.2.1.3
aldehyde dehydrogenase (acetaldehyde, NADP)	Nucleus	ACAL + H2O + NADP -> AC + 2 H + NADPH	Pyruvate Metabolism	SPAC9E9.09c	1.2.1.3
alcohol dehydrogenase (ethanol)	Nucleus	ETOH + NAD <-> ACAL + H + NADH	Pyruvate Metabolism	SPCC13B11.04c SPBC1539.07c	1.1.1.284
formaldehyde dehydrogenase	Nucleus	fald + GTHrd + NAD <-> SfGLUttH + H + NADH	Pyruvate Metabolism	SPCC13B11.04c SPBC1539.07c	1.1.1.284
acetyl-CoA hydrolase	Mitochondria	ACCoA + H2O -> AC + CoA + H	Pyruvate Metabolism	SPAC1952.09c	3.1.2.1
acetyl-CoA synthetase	Cytosol	AC + ATP + CoA -> ACCoA + AMP + PPi	Pyruvate Metabolism	SPCC191.02c	6.2.1.1
acetyl-CoA synthetase	Mitochondria	AC + ATP + CoA -> ACCoA + AMP + PPi	Pyruvate Metabolism	SPCC191.02c	6.2.1.1
acetyl-CoA synthetase	Peroxisome	AC + ATP + CoA -> ACCoA + AMP + PPi	Pyruvate Metabolism	SPCC191.02c	6.2.1.1
alcohol dehydrogenase, forward rxn (ethanol -> acetaldehyde)	Cytosol	ETOH + NAD -> ACAL + H + NADH	Pyruvate Metabolism	SPCC13B11.01	1.1.1.1
alcohol dehydrogenase, reverse rxn (acetaldehyde -> ethanol)	Cytosol	ACAL + H + NADH -> ETOH + NAD	Pyruvate Metabolism	SPAC5H10.06c	1.1.1.1
alcohol dehydrogenase, reverse rxn (acetaldehyde -> ethanol)	Mitochondria	ACAL + H + NADH -> ETOH + NAD	Pyruvate Metabolism	SPCC13B11.01	1.1.1.1
alcohol dehydrogenase (ethanol)	Cytosol	ETOH + NAD <-> ACAL + H + NADH	Pyruvate Metabolism	SPCC13B11.04c SPBC1539.07c	1.1.1.284
aldehyde dehydrogenase (acetylaldehyde, NAD)	Mitochondria	ACAL + H2O + NAD -> AC + 2 H + NADH	Pyruvate Metabolism	SPAC9E9.09c	1.2.1.3
aldehyde dehydrogenase (acetaldehyde, NADP)	Cytosol	ACAL + H2O + NADP -> AC + 2 H + NADPH	Pyruvate Metabolism	SPAC9E9.09c	1.2.1.3

aldehyde dehydrogenase (acetylaldehyde, NADP)	Mitochondria	ACAL + H2O + NADP -> AC + 2 H + NADPH	Pyruvate Metabolism	SPAC9E9.09c	1.2.1.3
formaldehyde dehydrogenase	Cytosol	fald + GTHrd + NAD <-> SfGLUttH + H + NADH	Pyruvate Metabolism	SPCC13B11.04c<br>SPBC1539.07c	1.1.1.284
homocitrate synthase	Mitochondria	ACCoA + AKG + H2O -> CoA + H + HCIT	Pyruvate Metabolism	SPBC1105.02c	2.3.3.14
homocitrate synthase	Nucleus	ACCoA + AKG + H2O -> CoA + H + HCIT	Pyruvate Metabolism	SPBC1105.02c	2.3.3.14
L-Lactate dehydrogenaseic		2 ficytc[m] + LAC[c] -> 2 focytc[m] + PYR[c]	Pyruvate Metabolism	SPCC191.07<br>SPAPB1A11.03	1.1.2.3
2-hexaprenyl-3-methyl-5-hydroxy-6-methoxy-1,4-benzoquinone methyltransferase	Mitochondria	2OMHMB + SAM -> SAH + H + Q6	Quinone Biosynthesis	SPCC4G3.04c<br>SPBC337.15c<br>SPAC19G12.11<br>SPBC2D10.18<br>SPBC146.12 SPCC162.05<br>SPBC1347.09	2.1.1.114
2-Hexaprenyl-6-methoxyphenol monooxygenase	Mitochondria	2OPMP + O2 -> 2OPMB + H2O	Quinone Biosynthesis	SPCC4G3.04c<br>SPBC337.15c<br>SPAC19G12.11<br>SPBC2D10.18<br>SPBC146.12 SPCC162.05<br>SPBC1347.09	2.1.1.114
2-hexaprenyl-6-methoxy-1,4-benzoquinone methyltransferase	Mitochondria	2OPMB + SAM -> 2OPMMB + SAH + H	Quinone Biosynthesis	SPCC4G3.04c<br>SPBC337.15c<br>SPAC19G12.11<br>SPBC2D10.18<br>SPBC146.12 SPCC162.05<br>SPBC1347.09	2.1.1.114
2-hexaprenyl-3-methyl-6-methoxy-1,4-benzoquinone monooxygenase	Mitochondria	2OPMMB + 0.5 O2 -> 2OMHMB	Quinone Biosynthesis	SPCC4G3.04c<br>SPBC337.15c<br>SPAC19G12.11<br>SPBC2D10.18<br>SPBC146.12 SPCC162.05<br>SPBC1347.09	2.1.1.114
3-(4-hydroxyphenyl-)lactate formation	Mitochondria	34Hpp + H + NADH -> 34Hpl + NAD	Quinone Biosynthesis		
S-adenosyl-L-methionine:3-hexaprenyl-4,5-dihydroxylate O-methyltransferase	Mitochondria	3H45DHBZ + SAM -> 3H4H5MOBZ + SAH + H	Quinone Biosynthesis	SPCC4G3.04c<br>SPBC337.15c<br>SPAC19G12.11<br>SPBC2D10.18<br>SPBC146.12 SPCC162.05<br>SPBC1347.09	2.1.1.114
3-Hexaprenyl-4-hydroxy-5-methoxybenzoate decarboxylase	Mitochondria	3H4H5MOBZ -> 2OPMP + CO2	Quinone Biosynthesis		
3-Hexaprenyl-4,5-dihydroxybenzoate hydroxylase	Cytosol	O4HBZ + 0.5 O2 -> 3H45DHBZ	Quinone Biosynthesis		
4-hydroxybenzoyl-CoA formation	Mitochondria	CoA + couCoA + H2O + NAD -> 4HbzCoA + ACCoA + H + NADH	Quinone Biosynthesis		
4-hydroxybenzoate formation	Mitochondria	4HbzCoA + H2O -> 4Hbz + CoA + H	Quinone Biosynthesis		
Chorismate pyruvate lyase	Cytosol	CHOR -> 4Hbz + PYR	Quinone Biosynthesis		
p-coumaroyl-CoA formation	Mitochondria	T4Hcinnm + ATP + CoA -> AMP + couCoA + PPi	Quinone Biosynthesis		
geranylgeranyltranstransferase	Cytosol	GGPP + IPPP -> PNPP + PPi	Quinone Biosynthesis		
Hydroxybenzoate C080prenyltransferase	Mitochondria	4Hbz + HXPP -> O4HBZ + PPi	Quinone Biosynthesis	SPAC56F8.04c	2.1.1.114
trans-pentaprenyltranstransferase	Mitochondria	IPPP + PNPP -> HXPP + PPi	Quinone Biosynthesis	SPBPJ4664.01<br>SPAC19G12.12	2.5.1.33
Quinolinate Synthase (Eukaryotic)	Cytosol	cmuSA -> H + H2O + Quln	Quinone Biosynthesis		
4-hydroxycinnamate formation	Mitochondria	34Hpl -> T4Hcinnm + H2O	Quinone Biosynthesis		
	Cytosol	FRPP + IPPP -> GGPP + PPi	Quinone Biosynthesis		
acid phosphatase	Golgi apparatus	fmn + H2O -> Pi + ribflv	Riboflavin Metabolism	SPBP4G3.02<br>SPBC428.03c<br>SPBC21H7.03c	3.1.3.2
2,5-diamino-6-ribitylamino-4(3H)-pyrimidinone 5'-phosphate deaminase	Mitochondria	25dtHpp + H + H2O -> 5aprbu + NH4	Riboflavin Metabolism	SPAC18B11.02c<br>SPCC4G3.16	
3,4-Dihydroxy-2-butanone-4-phosphate synthase	Mitochondria	ru5p-D -> db4p + FORM + H	Riboflavin Metabolism	SPBC23E6.06c	
acid phosphatase	Endoplasmic Reticulum	fmn + H2O -> Pi + ribflv	Riboflavin Metabolism	SPBP4G3.02<br>SPBC428.03c<br>SPBC21H7.03c	3.1.3.2
riboflavin synthase	Nucleus	2 dmlz -> 4r5au + ribflv	Riboflavin Metabolism	SPCC1450.13c	
2,5-diamino-6-ribosylamino-4(3H)-pyrimidinone 5'-phosphate reductase (nADPh)	Nucleus	25dHpp + H + NADPH -> 25dtHpp + NADP	Riboflavin Metabolism	SPBC21C3.10c	
2,5-diamino-6-ribitylamino-4(3H)-pyrimidinone 5'-phosphate deaminase	Nucleus	25dtHpp + H + H2O -> 5aprbu + NH4	Riboflavin Metabolism	SPAC18B11.02c<br>SPCC4G3.16	
riboflavin synthase	Nucleus	4r5au + db4p -> dmlz + 2 H2O + Pi	Riboflavin Metabolism	SPBC409.13	
riboflavin kinase	Nucleus	ATP + ribflv -> ADP + fmn + H	Riboflavin Metabolism	SPCC18.16c	
FMN reductase	Nucleus	fmn + H + NADPH -> fmnH2 + NADP	Riboflavin Metabolism	SPCC4B3.06c	
GTP cyclohydrolase II	Nucleus	GTP + 3 H2O -> 25dHpp + FORM + 2 H + PPi	Riboflavin Metabolism	SPAP27G11.09c	3.5.4.25
3,4-Dihydroxy-2-butanone-4-phosphate synthase	Nucleus	ru5p-D -> db4p + FORM + H	Riboflavin Metabolism	SPBC23E6.06c	
acid phosphatase	Extracellular	fmn + H2O -> Pi + ribflv	Riboflavin Metabolism	SPBP4G3.02<br>SPBC428.03c<br>SPBC21H7.03c	3.1.3.2
3,4-Dihydroxy-2-butanone-4-phosphate synthase	Cytosol	ru5p-D -> db4p + FORM + H	Riboflavin Metabolism	SPBC23E6.06c	
2,5-diamino-6-ribosylamino-4(3H)-pyrimidinone 5'-phosphate reductase (nADPh)	Cytosol	25dHpp + H + NADPH -> 25dtHpp + NADP	Riboflavin Metabolism	SPBC21C3.10c	
2,5-diamino-6-ribitylamino-4(3H)-pyrimidinone 5'-phosphate deaminase	Cytosol	25dtHpp + H + H2O -> 5aprbu + NH4	Riboflavin Metabolism	SPAC18B11.02c<br>SPCC4G3.16	
FMN adenylyltransferase	Cytosol	ATP + fmn + H -> fad + PPi	Riboflavin Metabolism	SPCC1235.04c	2.7.7.2
FMN adenylyltransferase	Nucleus	ATP + fmn + H -> fad + PPi	Riboflavin Metabolism	SPCC1235.04c	2.7.7.2
FMN reductase	Cytosol	fmn + H + NADPH -> fmnH2 + NADP	Riboflavin Metabolism	SPCC4B3.06c	
GTP cyclohydrolase II	Cytosol	GTP + 3 H2O -> 25dHpp + FORM + 2 H + PPi	Riboflavin Metabolism	SPAP27G11.09c	3.5.4.25
pyrimidine phosphatase	Cytosol	5aprbu + H2O -> 4r5au + Pi	Riboflavin Metabolism		
riboflavin kinase	Cytosol	ATP + ribflv -> ADP + fmn + H	Riboflavin Metabolism	SPCC18.16c	
riboflavin kinase	Mitochondria	ATP + ribflv -> ADP + fmn + H	Riboflavin Metabolism	SPCC18.16c	
riboflavin synthase	Cytosol	4r5au + db4p -> dmlz + 2 H2O + Pi	Riboflavin Metabolism	SPBC409.13	
riboflavin synthase	Cytosol	2 dmlz -> 4r5au + ribflv	Riboflavin Metabolism	SPCC1450.13c	
Sulfatide sulfohydrolase	Cytosol	gala + SO4 -> sft + H2O	Sphingolipid Metabolism	SPBPB10D8.02c	3.1.6.1
Sulfatide sulfohydrolase	Nucleus	gala + SO4 -> sft + H2O	Sphingolipid Metabolism	SPBPB10D8.02c	3.1.6.1
alpha-galactosidase, melibiase	Endoplasmic Reticulum	dgala + H2O -> gala + gal	Sphingolipid Metabolism	SPAC869.07c	3.2.1.22
alpha-galactosidase, melibiase	Extracellular	dgala + H2O -> gala + gal	Sphingolipid Metabolism	SPAC869.07c	3.2.1.22
Inositol phosphorylceramide synthase (ceramide-1, 24C)	Golgi apparatus	cer124 + 0.01 ptd1ino -> 0.01 12dgr + 0.01 ipc124	Sphingolipid Metabolism	SPAC3H8.06	
Inositol phosphorylceramide synthase (ceramide-1, 26C)	Golgi apparatus	cer126 + 0.01 ptd1ino -> 0.01 12dgr + 0.01 ipc126	Sphingolipid Metabolism	SPAC3H8.06	
Inositol phosphorylceramide synthase (ceramide-2, 24C)	Golgi apparatus	cer224 + 0.01 ptd1ino -> 0.01 12dgr + 0.01 ipc224	Sphingolipid Metabolism	SPAC3H8.06	
Inositol phosphoryceramide synthase (ceramide-2, 26C)	Golgi apparatus	cer226 + 0.01 ptd1ino -> 0.01 12dgr + 0.01 ipc226	Sphingolipid Metabolism	SPAC3H8.06	
Inositol phosphorylceramide synthase (ceramide-3, 24C)	Golgi apparatus	cer324 + 0.01 ptd1ino -> 0.01 12dgr + 0.01 ipc324	Sphingolipid Metabolism	SPAC3H8.06	

Inositol phosphorylceramide synthase (ceramide-3, 26C)	Golgi apparatus	cer326 + 0.01 ptd1ino -> 0.01 12dgr + 0.01 ipc326	Sphingolipid Metabolism	SPAC3H8.06	
mannose-inositiol phophorylceramide synthase (ceramide-1, 24C)	Golgi apparatus	GDPmann + 0.01 ipc124 -> GDP + H + 0.01 mipc124	Sphingolipid Metabolism	SPCC4F11.04c<br>SPAC17G8.11c<br>SPAC2F3.01	
mannose-inositol phosphorylceramide synthase (ceramide-1, 26C)	Golgi apparatus	GDPmann + 0.01 ipc126 -> GDP + H + 0.01 mipc126	Sphingolipid Metabolism	SPCC4F11.04c<br>SPAC17G8.11c<br>SPAC2F3.01	
mannose-inositol phosphorylceramide synthase (ceramide-2, 24C)	Golgi apparatus	GDPmann + 0.01 ipc224 -> GDP + H + 0.01 mipc224	Sphingolipid Metabolism	SPCC4F11.04c<br>SPAC17G8.11c<br>SPAC2F3.01	
mannose-inositol phosphorylceramide synthase (ceramide-2, 26C)	Golgi apparatus	GDPmann + 0.01 ipc226 -> GDP + H + 0.01 mipc226	Sphingolipid Metabolism	SPCC4F11.04c<br>SPAC17G8.11c<br>SPAC2F3.01	
mannose-inositol phosphorylceramide synthase (ceramide-3, 24C)	Golgi apparatus	GDPmann + 0.01 ipc324 -> GDP + H + 0.01 mipc324	Sphingolipid Metabolism	SPCC4F11.04c<br>SPAC17G8.11c<br>SPAC2F3.01	
mannose-inositol phosphorylceramide synthase (ceramide-3, 26C)	Golgi apparatus	GDPmann + 0.01 ipc326 -> GDP + H + 0.01 mipc326	Sphingolipid Metabolism	SPCC4F11.04c<br>SPAC17G8.11c<br>SPAC2F3.01	
mannose-inositiol phophorylceramide synthase (ceramide-1, 24C)	Endoplasmic Reticulum	GDPmann + 0.01 ipc124 -> GDP + H + 0.01 mipc124	Sphingolipid Metabolism	SPCC4F11.04c<br>SPAC17G8.11c	
mannose-inositol phosphorylceramide synthase (ceramide-1, 26C)	Endoplasmic Reticulum	GDPmann + 0.01 ipc126 -> GDP + H + 0.01 mipc126	Sphingolipid Metabolism	SPCC4F11.04c<br>SPAC17G8.11c	
mannose-inositol phosphorylceramide synthase (ceramide-2, 24C)	Endoplasmic Reticulum	GDPmann + 0.01 ipc224 -> GDP + H + 0.01 mipc224	Sphingolipid Metabolism	SPCC4F11.04c<br>SPAC17G8.11c	
mannose-inositol phosphorylceramide synthase (ceramide-2, 26C)	Endoplasmic Reticulum	GDPmann + 0.01 ipc226 -> GDP + H + 0.01 mipc226	Sphingolipid Metabolism	SPCC4F11.04c<br>SPAC17G8.11c	
mannose-inositol phosphorylceramide synthase (ceramide-3, 24C)	Endoplasmic Reticulum	GDPmann + 0.01 ipc324 -> GDP + H + 0.01 mipc324	Sphingolipid Metabolism	SPCC4F11.04c<br>SPAC17G8.11c	
mannose-inositol phosphorylceramide synthase (ceramide-3, 26C)	Endoplasmic Reticulum	GDPmann + 0.01 ipc326 -> GDP + H + 0.01 mipc326	Sphingolipid Metabolism	SPCC4F11.04c<br>SPAC17G8.11c	
Mannose-inositol phosphorylceramide, ceramide-1 (24C) phospholipase C	Mitochondria	H2O + 0.01 mipc124 -> cer124 + H + manmi1p-D	Sphingolipid Metabolism	SPBC32F12.01c	3.1.4.-
Mannose-inositol phosphorylceramide, ceramide-1 (26C) phospholipase C	Mitochondria	H2O + 0.01 mipc126 -> cer126 + H + manmi1p-D	Sphingolipid Metabolism	SPBC32F12.01c	3.1.4.-
Mannose-inositol phosphorylceramide, ceramide-2 (24C) phospholipase C	Mitochondria	H2O + 0.01 mipc224 -> cer224 + H + manmi1p-D	Sphingolipid Metabolism	SPBC32F12.01c	3.1.4.-
Mannose-inositol phosphorylceramide, ceramide-2 (26C) phospholipase C	Mitochondria	H2O + 0.01 mipc226 -> cer226 + H + manmi1p-D	Sphingolipid Metabolism	SPBC32F12.01c	3.1.4.-
Mannose-inositol phosphorylceramide, ceramide-3 (24C) phospholipase C	Mitochondria	H2O + 0.01 mipc324 -> cer324 + H + manmi1p-D	Sphingolipid Metabolism	SPBC32F12.01c	3.1.4.-
Mannose-inositol phosphorylceramide, ceramide-3 (26C) phospholipase C	Mitochondria	H2O + 0.01 mipc326 -> cer326 + H + manmi1p-D	Sphingolipid Metabolism	SPBC32F12.01c	3.1.4.-
Phytosphingosine synthesis	Endoplasmic Reticulum	H + NADPH + O2 + spHgn -> H2O + NADP + pspHings	Sphingolipid Metabolism	SPBC887.15c	1.14.-.-
serine C-palmitoyltransferase	Endoplasmic Reticulum	H + C160CoA + SER -> 3dspHgn + CO2 + CoA	Sphingolipid Metabolism	SPAC21E11.08+SPBC18E5.02c	2.3.1.50
Mannose-inositol phosphorylceramide, ceramide-1 (24C) phospholipase C	Endoplasmic Reticulum	H2O + 0.01 mipc124 -> cer124 + H + manmi1p-D	Sphingolipid Metabolism	SPBC32F12.01c	3.1.4.-
Mannose-inositol phosphorylceramide, ceramide-1 (26C) phospholipase C	Endoplasmic Reticulum	H2O + 0.01 mipc126 -> cer126 + H + manmi1p-D	Sphingolipid Metabolism	SPBC32F12.01c	3.1.4.-
Mannose-inositol phosphorylceramide, ceramide-2 (24C) phospholipase C	Endoplasmic Reticulum	H2O + 0.01 mipc224 -> cer224 + H + manmi1p-D	Sphingolipid Metabolism	SPBC32F12.01c	3.1.4.-
Mannose-inositol phosphorylceramide, ceramide-2 (26C) phospholipase C	Endoplasmic Reticulum	H2O + 0.01 mipc226 -> cer226 + H + manmi1p-D	Sphingolipid Metabolism	SPBC32F12.01c	3.1.4.-
Mannose-inositol phosphorylceramide, ceramide-3 (24C) phospholipase C	Endoplasmic Reticulum	H2O + 0.01 mipc324 -> cer324 + H + manmi1p-D	Sphingolipid Metabolism	SPBC32F12.01c	3.1.4.-
Mannose-inositol phosphorylceramide, ceramide-3 (26C) phospholipase C	Endoplasmic Reticulum	H2O + 0.01 mipc326 -> cer326 + H + manmi1p-D	Sphingolipid Metabolism	SPBC32F12.01c	3.1.4.-
Ceramide-1 hydroxylase (24C)	Cytosol	cer124 + H + NADPH + O2 -> cer224 + H2O + NADP	Sphingolipid Metabolism	SPBC887.15c	
Ceramide-1 hydroxylase (26C)	Cytosol	cer126 + H + NADPH + O2 -> cer226 + H2O + NADP	Sphingolipid Metabolism	SPBC887.15c	
Ceramide-2' synthase (24C)	Cytosol	cer124 + H + NADPH + O2 -> cer2'24 + H2O + NADP	Sphingolipid Metabolism	SPAC19G12.08	
Ceramide-2' synthase (26C)	Cytosol	cer126 + H + NADPH + O2 -> cer2'26 + H2O + NADP	Sphingolipid Metabolism	SPAC19G12.08	
Ceramide-3 synthase (24C)	Cytosol	cer224 + H + NADPH + O2 -> cer324 + H2O + NADP	Sphingolipid Metabolism	SPAC19G12.08	
Ceramide-3 synthase (26C)	Cytosol	cer226 + H + NADPH + O2 -> cer326 + H2O + NADP	Sphingolipid Metabolism	SPAC19G12.08	
inositol phosphorylceramide, ceramide-1 (24C) phospholipase C	Cytosol	H2O + 0.01 ipc124 -> cer124 + H + mi1p-D	Sphingolipid Metabolism	SPBC32F12.01c	
inositol phosphorylceramide, ceramide-1 (26C) phospholipase C	Cytosol	H2O + 0.01 ipc126 -> cer126 + H + mi1p-D	Sphingolipid Metabolism	SPBC32F12.01c	
inositol phosphorylceramide, ceramide-2 (24C) phospholipase C	Cytosol	H2O + 0.01 ipc224 -> cer224 + H + mi1p-D	Sphingolipid Metabolism	SPBC32F12.01c	
inositol phosphorylceramide, ceramide-2 (26C) phospholipase C	Cytosol	H2O + 0.01 ipc226 -> cer226 + H + mi1p-D	Sphingolipid Metabolism	SPBC32F12.01c	
inositol phosphorylceramide, ceramide-3 (24C) phospholipase C	Cytosol	H2O + 0.01 ipc324 -> cer324 + H + mi1p-D	Sphingolipid Metabolism	SPBC32F12.01c	
inositol phosphorylceramide, ceramide-3 (26C) phospholipase C	Cytosol	H2O + 0.01 ipc326 -> cer326 + H + mi1p-D	Sphingolipid Metabolism	SPBC32F12.01c	
Mannose-(inositol-P)2-ceramide, ceramide-1 (24C) phospholipase C	Cytosol	H2O + 0.01 mip2c124 -> cer124 + H + man2mi1p-D	Sphingolipid Metabolism	SPBC32F12.01c	
Mannose-(inositol-P)2-ceramide, ceramide-1 (26C) phospholipase C	Cytosol	H2O + 0.01 mip2c126 -> cer126 + H + man2mi1p-D	Sphingolipid Metabolism	SPBC32F12.01c	
Mannose-(inositol-P)2-ceramide, ceramide-2 (24C) phospholipase C	Cytosol	H2O + 0.01 mip2c224 -> cer224 + H + man2mi1p-D	Sphingolipid Metabolism	SPBC32F12.01c	
Mannose-(inositol-P)2-ceramide, ceramide-2 (26C) phospholipase C	Cytosol	H2O + 0.01 mip2c226 -> cer226 + H + man2mi1p-D	Sphingolipid Metabolism	SPBC32F12.01c	
Mannose-(inositol-P)2-ceramide, ceramide-3 (24C) phospholipase C	Cytosol	H2O + 0.01 mip2c324 -> cer324 + H + man2mi1p-D	Sphingolipid Metabolism	SPBC32F12.01c	
Mannose-(inositol-P)2-ceramide, ceramide-3 (26C) phospholipase C	Cytosol	H2O + 0.01 mip2c326 -> cer326 + H + man2mi1p-D	Sphingolipid Metabolism	SPBC32F12.01c	
mannose-(inositol-P)2-ceramide synthase (ceramide-1, 24C)	Cytosol	0.01 mipc124 + 0.01 ptd1ino -> 0.01 12dgr + 0.01 mip2c124	Sphingolipid Metabolism		
mannose-(inositol-P)2-ceramide synthase (ceramide-1, 26C)	Cytosol	0.01 mipc126 + 0.01 ptd1ino -> 0.01 12dgr + 0.01 mip2c126	Sphingolipid Metabolism		
mannose-(inositol-P)2-ceramide synthase (ceramide-2, 26C)	Cytosol	0.01 mipc224 + 0.01 ptd1ino -> 0.01 12dgr + 0.01 mip2c224	Sphingolipid Metabolism		
mannose-(inositol-P)2-ceramide synthase (ceramide-2, 24C)	Cytosol	0.01 mipc226 + 0.01 ptd1ino -> 0.01 12dgr + 0.01 mip2c226	Sphingolipid Metabolism		
mannose-(inositol-P)2-ceramide synthase (ceramide-3, 24C)	Cytosol	0.01 mipc324 + 0.01 ptd1ino -> 0.01 12dgr + 0.01 mip2c324	Sphingolipid Metabolism		
mannose-(inositol-P)2-ceramide synthase (ceramide-3, 26C)	Cytosol	0.01 mipc326 + 0.01 ptd1ino -> 0.01 12dgr + 0.01 mip2c326	Sphingolipid Metabolism		
sphingoid base-phosphate phosphatase (sphinganine 1-phosphatase)	Endoplasmic Reticulum	H2O + spH1p -> Pi + spHgn	Sphingolipid Metabolism	SPAC823.11	3.1.3.-
sphingoid base-phosphate phosphatase (phytosphingosine 1-phosphate)	Endoplasmic Reticulum	H2O + pspH1p -> Pi + pspHings	Sphingolipid Metabolism	SPAC823.11	3.1.3.-
Exo-1,3-beta-glucan glucohydrase	Endoplasmic Reticulum	13BDGLCn + H2O -> GLC	Starch and Sucrose Metabolism	SPBC1105.05<br>SPAC12B10.11	3.2.1.58
Endo-1,3-beta-glucan glucohydrase	Cytosol	13BDGLCn + H2O -> GLC	Starch and Sucrose Metabolism	SPAC26H5.08c	3.2.1.58
Hydroxymethylglutaryl CoA synthase	Nucleus	CoA + H + HmgCoA <-> AACoA + ACCoA + H2O	Sterol Metabolism	SPAC4F8.14c	2.3.3.10
mevalonate kinase (CTP)	Nucleus	CTP + mev-R -> 5pmev + CDP + H	Sterol Metabolism	SPAC13G6.11c	2.7.1.36

Hydroxymethylglutaryl CoA reductase	Endoplasmic Reticulum	CoA + mev-R + 2 NADP <-> 2 H + HmgCoA + 2 NADPH	Sterol Metabolism	SPCC162.09c	1.1.1.34
Hydroxymethylglutaryl CoA reductase	Nucleus	CoA + mev-R + 2 NADP <-> 2 H + HmgCoA + 2 NADPH	Sterol Metabolism	SPCC162.09c	1.1.1.34
Hydroxymethylglutaryl CoA synthase	Cytosol	CoA + H + HmgCoA <-> AACoA + ACCoA + H2O	Sterol Metabolism	SPAC4F8.14c	2.3.3.10
Hydroxymethylglutaryl CoA synthase	Endoplasmic Reticulum	CoA + H + HmgCoA <-> AACoA + ACCoA + H2O	Sterol Metabolism	SPAC4F8.14c	2.3.3.10
mevalonate kinase (ATP)	Cytosol	ATP + mev-R -> 5pmev + ADP + H	Sterol Metabolism	SPAC13G6.11c	2.7.1.36
mevalonate kinase (ATP)	Nucleus	ATP + mev-R -> 5pmev + ADP + H	Sterol Metabolism	SPAC13G6.11c	2.7.1.36
mevalonate kinase (CTP)	Cytosol	CTP + mev-R -> 5pmev + CDP + H	Sterol Metabolism	SPAC13G6.11c	2.7.1.36
mevalonate kinase (GTP)	Cytosol	GTP + mev-R -> 5pmev + GDP + H	Sterol Metabolism	SPAC13G6.11c	2.7.1.36
mevalonate kinase (GTP)	Nucleus	GTP + mev-R -> 5pmev + GDP + H	Sterol Metabolism	SPAC13G6.11c	2.7.1.36
mevalonate kinase (UTP)	Cytosol	UTP + mev-R -> 5pmev + UDP + H	Sterol Metabolism	SPAC13G6.11c	2.7.1.36
mevalonate kinase (UTP)	Nucleus	UTP + mev-R -> 5pmev + UDP + H	Sterol Metabolism	SPAC13G6.11c	2.7.1.36
phosphomevalonate kinase	Cytosol	5pmev + ATP -> 5dpmev + ADP	Sterol Metabolism	SPAC343.01c	2.7.4.2
phosphomevalonate kinase	Mitochondria	5pmev + ATP -> 5dpmev + ADP	Sterol Metabolism	SPAC343.01c	2.7.4.2
phosphomevalonate kinase	Nucleus	5pmev + ATP -> 5dpmev + ADP	Sterol Metabolism	SPAC343.01c	2.7.4.2
diphosphomevalonate decarboxylase	Cytosol	5dpmev + ATP -> ADP + CO2 + IPPP + Pi	Sterol Metabolism	SPAC24C9.03	4.1.1.33
diphosphomevalonate decarboxylase	Nucleus	5dpmev + ATP -> ADP + CO2 + IPPP + Pi	Sterol Metabolism	SPAC24C9.03	4.1.1.33
isopentenyl-diphosphate D-isomerase	Cytosol	IPPP <-> DMPP	Sterol Metabolism	SPBC106.15	5.3.3.2
isopentenyl-diphosphate D-isomerase	Nucleus	IPPP <-> DMPP	Sterol Metabolism	SPBC106.15	5.3.3.2
dimethylallyltranstransferase	Cytosol	DMPP + IPPP -> grdp + PPi	Sterol Metabolism	SPBC36.06c SPAC6F12.13c	2.5.1.10
dimethylallyltranstransferase	Nucleus	DMPP + IPPP -> grdp + PPi	Sterol Metabolism	SPBC36.06c SPAC6F12.13c	2.5.1.10
geranyltranstransferase	Cytosol	grdp + IPPP -> FRPP + PPi	Sterol Metabolism	SPBC36.06c SPAC6F12.13c	2.5.1.10
geranyltranstransferase	Nucleus	grdp + IPPP -> FRPP + PPi	Sterol Metabolism	SPBC36.06c SPAC6F12.13c	2.5.1.10
Squalene synthase	Endoplasmic Reticulum	2 FRPP + H + NADPH -> NADP + 2 PPi + SQL	Sterol Metabolism	SPBC646.05c	2.5.1.21
Squalene epoxidase (NADP)	Endoplasmic Reticulum	FADH2 + O2 + SQL -> SQ23EPX + H2O + FAD	Sterol Metabolism	SPBC713.12	1.14.99.7
lanosterol synthase	Cytosol	SQ23EPX -> lanost	Sterol Metabolism	SPAC13G7.01c	5.4.99.7
lanosterol synthase	Endoplasmic Reticulum	SQ23EPX -> lanost	Sterol Metabolism	SPAC13G7.01c	5.4.99.7
Acyl-CoA:lanosterol acyltransferase	Endoplasmic Reticulum	0.655 C161CoA + 0.01 lanost + 0.27 C181CoA + 0.02 C160CoA + 0.03 C180CoA + 0.015 C140CoA -> CoA + 0.01 lanostest	Sterol Metabolism	SPAC13G7.05 SPCP1E11.05	2.3.1.26
lanosterol ester hydrolase	Cytosol	H2O + 0.01 lanostest -> H + 0.02 C160 + 0.655 C161 + 0.01 lanost + 0.03 C180 + 0.27 C181 + 0.015 C140	Sterol Metabolism	SPCC1672.09 SPBC14C8.15 SPBC16A3.12c	
lanosterol ester hydrolase (extracellular)	Extracellular	H2O + 0.01 lanostest -> H + 0.02 C160 + 0.655 C161 + 0.01 lanost + 0.03 C180 + 0.27 C181 + 0.015 C140	Sterol Metabolism		
lanosterol ester hydrolase	Golgi apparatus	H2O + 0.01 lanostest -> H + 0.02 C160 + 0.655 C161 + 0.01 lanost + 0.03 C180 + 0.27 C181 + 0.015 C140	Sterol Metabolism	SPCC1672.09 SPBC14C8.15 SPBC16A3.12c	
cytochrome P450 lanosterol 14-alpha-demethylase (NADP)	Endoplasmic Reticulum	3 H + lanost + 3 NADPH + 3 O2 -> 44mctr + FORM + 4 H2O + 3 NADP	Sterol Metabolism	SPAC13A11.02c	1.14.13.70, 1.6.2.4
C-14 sterol reductase	Endoplasmic Reticulum	44mctr + H + NADPH -> 44mzym + NADP	Sterol Metabolism	SPBC16G5.18	1.3.1.70
C-4 sterol methyl oxidase (4,4-dimethylzymosterol)	Endoplasmic Reticulum	44mzym + 3 H + 3 NADPH + 3 O2 -> 44mzymc + 4 H2O + 3 NADP	Sterol Metabolism	SPAC630.08c	1.14.13.72
C-3 sterol dehydrogenase (4-methylzymosterol)	Endoplasmic Reticulum	44mzymc + NAD -> 3k4mzym + CO2 + H + NADH	Sterol Metabolism	SPBC3F6.02c	1.1.1.170
C-3 sterol dehydrogenase (4-methylzymosterol),golgi	Golgi apparatus	44mzymc + NAD -> 3k4mzym + CO2 + H + NADH	Sterol Metabolism	SPBC3F6.02c	1.1.1.170
C-3 sterol keto reductase (4-methylzymosterol)	Endoplasmic Reticulum	3k4mzym + H + NADPH -> 4mzym + NADP	Sterol Metabolism	SPBC1709.07	1.1.1.270
C-4 sterol methyl oxidase (4-methylzymosterol)	Endoplasmic Reticulum	4mzym + 3 H + 3 NADPH + 3 O2 -> zym_int1 + 4 H2O + 3 NADP	Sterol Metabolism	SPAC630.08c	1.14.13.72
C-3 sterol dehydrogenase (zymosterol), endoplamsic reticulum	Endoplasmic Reticulum	NAD + zym_int1 -> CO2 + H + NADH + zym_int2	Sterol Metabolism	SPBC3F6.02c	1.1.1.170
C-3 sterol dehydrogenase (zymosterol)	Golgi apparatus	NAD + zym_int1 -> CO2 + H + NADH + zym_int2	Sterol Metabolism	SPBC3F6.02c	1.1.1.170
C-3 sterol keto reductase (zymosterol)	Endoplasmic Reticulum	H + NADPH + zym_int2 -> NADP + zymst	Sterol Metabolism	SPBC1709.07	1.1.1.270
Acyl-CoA:zymosterol acyltransferase	Endoplasmic Reticulum	0.655 C161CoA + 0.27 C181CoA + 0.02 C160CoA + 0.03 C180CoA + 0.015 C140CoA + 0.01 zymst -> CoA + 0.01 zymstest	Sterol Metabolism	SPAC13G7.05 SPCP1E11.05	2.3.1.26
zymosterol ester hydrolase	Cytosol	H2O + 0.01 zymstest -> H + 0.02 C160 + 0.655 C161 + 0.03 C180 + 0.27 C181 + 0.015 C140 + 0.01 zymst	Sterol Metabolism	SPCC1672.09 SPBC16A3.12c SPBC14C8.15	
zymosterol ester hydrolase (extracellular)	Extracellular	H2O + 0.01 zymstest -> H + 0.02 C160 + 0.655 C161 + 0.03 C180 + 0.27 C181 + 0.015 C140 + 0.01 zymst	Sterol Metabolism		
S-adenosyl-methionine delta-24-sterol-c-methyltransferase	Endoplasmic Reticulum	SAM + zymst -> SAH + fecost + H	Sterol Metabolism	SPBC16E9.05	2.1.1.41
S-adenosyl-methionine delta-24-sterol-c-methyltransferase	Nucleus	SAM + zymst -> SAH + fecost + H	Sterol Metabolism	SPBC16E9.05	2.1.1.41
Acyl-CoA:fecosterol acyltransferase	Endoplasmic Reticulum	0.01 fecost + 0.655 C161CoA + 0.27 C181CoA + 0.02 C160CoA + 0.03 C180CoA + 0.015 C140CoA -> CoA + 0.01 fecostest	Sterol Metabolism	SPAC13G7.05 SPCP1E11.05	2.3.1.26
fecosterol ester hydrolase	Cytosol	0.01 fecostest + H2O -> 0.01 fecost + H + 0.02 C160 + 0.655 C161 + 0.03 C180 + 0.27 C181 + 0.015 C140	Sterol Metabolism	SPCC1672.09 SPBC14C8.15 SPBC16A3.12c	
fecosterol ester hydrolase (extracellular	Extracellular	0.01 fecostest + H2O -> 0.01 fecost + H + 0.02 C160 + 0.655 C161 + 0.03 C180 + 0.27 C181 + 0.015 C140	Sterol Metabolism		
fecosterol ester hydrolase	Golgi apparatus	0.01 fecostest + H2O -> 0.01 fecost + H + 0.02 C160 + 0.655 C161 + 0.03 C180 + 0.27 C181 + 0.015 C140	Sterol Metabolism	SPCC1672.09 SPBC14C8.15 SPBC16A3.12c	
C-8 sterol isomerase	Endoplasmic Reticulum	fecost -> ePist	Sterol Metabolism	SPAC20G8.07c	
Acyl-CoA:ePisterol acyltransferase	Cytosol	0.01 ePist + 0.655 C161CoA + 0.27 C181CoA + 0.02 C160CoA + 0.03 C180CoA + 0.015 C140CoA -> CoA + 0.01 ePistest	Sterol Metabolism	SPAC13G7.05 SPCP1E11.05	2.3.1.26
ePisterol ester hydrolase	Cytosol	0.01 ePistest + H2O -> 0.01 ePist + H + 0.02 C160 + 0.655 C161 + 0.03 C180 + 0.27 C181 + 0.015 C140	Sterol Metabolism	SPCC1672.09 SPBC14C8.15 SPBC16A3.12c	
ePisterol ester hydrolase (extracellular)	Extracellular	0.01 ePistest + H2O -> 0.01 ePist + H + 0.02 C160 + 0.655 C161 + 0.03 C180 + 0.27 C181 + 0.015 C140	Sterol Metabolism		
ePisterol ester hydrolase	Golgi apparatus	0.01 ePistest + H2O -> 0.01 ePist + H + 0.02 C160 + 0.655 C161 + 0.03 C180 + 0.27 C181 + 0.015 C140	Sterol Metabolism	SPCC1672.09 SPBC14C8.15 SPBC16A3.12c	
cholestenol delta-isomerase, lumped reaction	Cytosol	SAM + O2 + zymst -> SAH + ergtetrol + H + 2 H2O	Sterol Metabolism		
C-s24 sterol reductase	Endoplasmic Reticulum	ergtetrol + H + NADPH -> ergst + NADP	Sterol Metabolism	SPAC20G4.07c	1.3.1.71
Acyl-CoA:ergosterol acyltransferase	Endoplasmic Reticulum	0.01 ergst + 0.655 C161CoA + 0.27 C181CoA + 0.02 C160CoA + 0.03 C180CoA + 0.015 C140CoA -> CoA + 0.01 ergstest	Sterol Metabolism	SPAC13G7.05 SPCP1E11.05	2.3.1.26
ergosterol ester hydrolase	Cytosol	0.01 ergstest + H2O -> 0.01 ergst + H + 0.02 C160 + 0.655 C161 + 0.03 C180 + 0.27 C181 + 0.015 C140	Sterol Metabolism	SPCC1672.09 SPBC14C8.15 SPBC16A3.12c	
ergosterol ester hydrolase (extracellular)	Extracellular	0.01 ergstest + H2O -> 0.01 ergst + H + 0.02 C160 + 0.655 C161 + 0.03 C180 + 0.27 C181 + 0.015 C140	Sterol Metabolism		

ergosterol ester hydrolase aparatus	Golgi apparatus	0.01 ergstest + H2O -> 0.01 ergst + H + 0.02 C160 + 0.655 C161 + 0.03 C180 + 0.27 C181 + 0.015 C140	Sterol Metabolism	SPCC1672.09 SPBC14C8.15 SPBC16A3.12c	
UDP-glucose:ergosterol glucosyltransferase	Cytosol	ergst + UDPg -> ergst3GLC + H + UDP	Sterol Metabolism		
Sucrose 6-phosphate fructohydrolase	Extracellular	sucr6p + H2O -> fru + G6P	Sucrose and Starch Metabolism	SPCC191.11	3.2.1.26
Sucrose 6-phosphate fructohydrolase	Cytosol	sucr6p + H2O -> fru + G6P	Sucrose and Starch Metabolism	SPAC8E11.01c	3.2.1.26
TaURIne dioxygenase	Cytosol	AKG + O2 + taur -> aacald + CO2 + H + SO3 + SUCC	Taurine Metabolism		
thiamin phosphatase	Endoplasmic Reticulum	H2O + THMMP -> Pi + THM	Thiamine Metabolism	SPBP4G3.02 SPBC428.03c SPBC21H7.03c	3.1.3.2
thiamin phosphatase	Golgi apparatus	H2O + THMMP -> Pi + THM	Thiamine Metabolism	SPBC428.03c	3.1.3.2
4-amino-5-hydroxymethyl-2-methylpyrimidine synthetase	Cytosol	air + 2 H -> 4AHMMP + gcald + Pi	Thiamine Metabolism		
hydroxyethylthiazole kinase	Cytosol	4mHetz + ATP -> 4mpetz + ADP + H	Thiamine Metabolism	SPAC23H4.10c	2.7.1.50
thiamin diphosphatase	Extracellular	2 H2O + THMPP -> H + 2 Pi + THM	Thiamine Metabolism	SPBP4G3.02 SPBC428.03c SPBC21H7.03c	3.1.3.2
thiamin phosphatase	Extracellular	H2O + THMMP -> Pi + THM	Thiamine Metabolism	SPBP4G3.02 SPBC428.03c SPBC21H7.03c	3.1.3.2
thiazole phosphate synthesis (xylulose 5-phosphate)	Cytosol	ACHMS + CYS + GLY + H + xu5p-D -> 4abut + 4mpetz + AC + CO2 + 3 H2O + NH4 + PYR	Thiamine Metabolism		
thiazole phosphate synthesis (ribose 5-phosphate)	Cytosol	ACHMS + CYS + GLY + H + R5P -> 4abut + 4mpetz + AC + CO2 + 3 H2O + NH4 + PYR	Thiamine Metabolism		
thiamine diphosphokinase	Cytosol	ATP + THM -> AMP + H + THMPP	Thiamine Metabolism	SPAC6F12.05c	2.7.6.2
thiamine-diphosphate kinase	Cytosol	ATP + THMPP -> ADP + THMTP	Thiamine Metabolism	SPAC6F12.05c	2.7.6.2
thiaminase	Cytosol	H2O + THM -> 4AHMMP + 4mHetz + H	Thiamine Metabolism		
thiamine-phosphate kinase	Cytosol	ATP + THMMP <-> ADP + THMPP	Thiamine Metabolism		
thiamine-phosphate diphosphorylase	Cytosol	2maHmp + 4mpetz + H -> PPi + THMMP	Thiamine Metabolism	SPAC23H4.10c	2.7.1.50
homoisocitrate dehydrogenase	Cytosol	HICIT + NAD <-> H + NADH + oxag	Threonine and Lysine Metabolism	SPAC31G5.04	1.1.1.87
L-allo-Threonine Aldolase	Nucleus	aTHR -> ACAL + GLY	Threonine and Lysine Metabolism	SPAC23H3.09c	4.1.2.5
threonine synthase	Nucleus	H2O + PHOM -> Pi + THR	Threonine and Lysine Metabolism	SPAC9E9.06c	4.2.3.1
4-Hydroxy-L-threonine synthase	Nucleus	H2O + PHTHR -> 4HtHr + Pi	Threonine and Lysine Metabolism	SPAC9E9.06c	4.2.3.1
Threonine aldolase	Nucleus	THR -> ACAL + GLY	Threonine and Lysine Metabolism	SPAC23H3.09c	4.1.2.5
4-Hydroxy-L-threonine synthase	Cytosol	H2O + PHTHR -> 4HtHr + Pi	Threonine and Lysine Metabolism	SPAC9E9.06c	4.2.3.1
L-aminoadipate-semialdehyde dehydrogenase (NADPH)	Cytosol	L2aADP + ATP + H + NADPH -> L2aADP6SA + AMP + NADP + PPi	Threonine and Lysine Metabolism	SPAP7G5.04c	1.2.1.31
L-aminoadipate-semialdehyde dehydrogenase (NADH)	Cytosol	L2aADP + ATP + H + NADH -> L2aADP6SA + AMP + NAD + PPi	Threonine and Lysine Metabolism	SPAP7G5.04c	1.2.1.31
2-aminoadipate transaminase	Cytosol	2oxoADP + GLU <-> L2aADP + AKG	Threonine and Lysine Metabolism		
L-2-amino-3-oxobutanoate decarboxylation	Cytosol	2aobut + H -> aact + CO2	Threonine and Lysine Metabolism		
L-allo-threonine dehydrogenase	Cytosol	aTHR + NADP <-> 2aobut + H + NADPH	Threonine and Lysine Metabolism	SPAC521.03	
glycine C-acetyltransferase	Cytosol	2aobut + CoA -> ACCoA + GLY	Threonine and Lysine Metabolism		2.3.1.29
homoacontinate hydratase	Mitochondria	b124tc + H2O <-> HICIT	Threonine and Lysine Metabolism	SPAC343.16	4.2.1.36
homoisocitrate dehydrogenase	Mitochondria	HICIT + NAD <-> H + NADH + oxag	Threonine and Lysine Metabolism	SPAC31G5.04	1.1.1.87
2-methylcitrate dehydratase	Mitochondria	HCIT <-> b124tc + H2O	Threonine and Lysine Metabolism		
2-Oxobutanoate dehydrogenase	Mitochondria	2obut + CoA + NAD -> CO2 + NADH + ppCoA	Threonine and Lysine Metabolism		1.2.7.2
non-enzymatic reaction	Mitochondria	H + oxag <-> 2oxoADP + CO2	Threonine and Lysine Metabolism		
saccharoPine dehydrogenase (NADP, L-glutamate forming)	Cytosol	L2aADP6SA + GLU + H + NADPH <-> H2O + NADP + SAccrp-L	Threonine and Lysine Metabolism	SPBC3B8.03	1.5.1.10
saccharoPine dehydrogenase (NAD, L-lysine forming)	Cytosol	H2O + NAD + SAccrp-L <-> AKG + H + LYS + NADH	Threonine and Lysine Metabolism	SPAC227.18	1.5.1.7
L-allo-Threonine Aldolase	Cytosol	aTHR -> ACAL + GLY	Threonine and Lysine Metabolism	SPAC23H3.09c	4.1.2.5
Threonine aldolase	Cytosol	THR -> ACAL + GLY	Threonine and Lysine Metabolism	SPAC23H3.09c	4.1.2.5
L-threonine deaminase	Cytosol	THR -> 2obut + NH4	Threonine and Lysine Metabolism	SPCC320.14 SPBC1677.03c	4.3.1.19
L-threonine deaminase	Mitochondria	THR -> 2obut + NH4	Threonine and Lysine Metabolism	SPBC1677.03c	4.3.1.19
threonine synthase	Cytosol	H2O + PHOM -> Pi + THR	Threonine and Lysine Metabolism	SPAC9E9.06c	4.2.3.1
6-phospho-D-glucono-1,5-lactone endoplasmic reticular transport via diffusion		6pgl[c] <-> 6pgl[r]	Transport, Endoplasmic Reticulum		
ceramide-1 (C24) endoplasmic reticular transport	Membrane	cer124[c] <-> cer124[r]	Transport, Endoplasmic Reticulum		
ceramide-1 (C26) endoplasmic reticular transport	Membrane	cer126[c] <-> cer126[r]	Transport, Endoplasmic Reticulum		
ceramide-2 (C24) endoplasmic reticular transport	Membrane	cer224[c] <-> cer224[r]	Transport, Endoplasmic Reticulum		
ceramide-2 (C26) endoplasmic reticular transport	Membrane	cer226[c] <-> cer226[r]	Transport, Endoplasmic Reticulum		
dolichol phosphate endoplasmic reticular transport via proton symport	Membrane	dolp[c] + H[c] <-> dolp[r] + H[r]	Transport, Endoplasmic Reticulum		
ergosterol endoplasmic reticular transport	Membrane	ergst[r] <-> ergst[c]	Transport, Endoplasmic Reticulum		
Ergosta-5,6,22,24,(28)-tetraen-3beta-ol endoplamic reticular transport	Membrane	ergtetrol[c] <-> ergtetrol[r]	Transport, Endoplasmic Reticulum		
glucose 6-phosphate endoplasmic reticular transport via diffusion	Membrane	G6P[c] <-> G6P[r]	Transport, Endoplasmic Reticulum		
H2O endoplasmic reticulum transport	Membrane	H2O[c] <-> H2O[r]	Transport, Endoplasmic Reticulum		
mannan endoplasmic reticulum transport via diffusion	Membrane	mannan[c] <-> mannan[r]	Transport, Endoplasmic Reticulum		
O2 transport	Membrane	O2[c] <-> O2[r]	Transport, Endoplasmic Reticulum		
phytosphinganine 1-phosphate endoplasmic reticular transport	Membrane	pspH1p[c] -> pspH1p[r]	Transport, Endoplasmic Reticulum		
sphinganine 1-phosphate endoplasmic reticular transport	Membrane	spH1p[c] -> spH1p[r]	Transport, Endoplasmic Reticulum		
Squalene-2,3-epoxide endoplamic reticular transport	Membrane	SQ23EPX[r] <-> SQ23EPX[c]	Transport, Endoplasmic Reticulum		
squalene endoplamic reticular transport	Membrane	SQL[c] <-> SQL[r]	Transport, Endoplasmic Reticulum		
Diphosphate endoplasmic reticulum transport	Membrane	PPi[c] <-> PPi[r]	Transport, Endoplasmic Reticulum		
FAD endoplasmic reticulum transport	Membrane	FAD[m] <-> FAD[r]	Transport, Endoplasmic Reticulum		
Proton endoplasmic reticulum transport	Membrane	h[c] <-> h[r]	Transport, Endoplasmic Reticulum		
Formate endoplasmic reticulum transport	Membrane	FORM[c] <-> FORM[r]	Transport, Endoplasmic Reticulum		
FADH2 endoplasmic reticulum transport	Membrane	FADH2[m] <-> FADH2[r]	Transport, Endoplasmic Reticulum		
NADH endoplasmic reticulum transport	Membrane	NADH[c] <-> NADH[r]	Transport, Endoplasmic Reticulum		
NAD endoplasmic reticulum transport	Membrane	NAD[c] <-> NAD[r]	Transport, Endoplasmic Reticulum		
CO2 endoplasmic reticulum transport	Membrane	CO2[c] <-> CO2[r]	Transport, Endoplasmic Reticulum		
SAH endoplasmic reticulum transport	Membrane	SAH[c] <-> SAH[r]	Transport, Endoplasmic Reticulum		
3mop reversible trasport	Membrane	3MOP[e] <-> 3MOP[c]	Transport, Extracellular		
acetaldehyde reversible transport	Membrane	ACAL[e] <-> ACAL[c]	Transport, Extracellular		
acetate reversible transport via proton symport	Membrane	ac[e] + H[e] <-> ac[c] + H[c]	Transport, Extracellular		
acetate transporter	Membrane	ac[e] <-> ac[c]	Transport, Extracellular		
adenine transport in via proton symport	Membrane	ADE[e] + H[e] -> ADE[c] + H[c]	Transport, Extracellular		
adenosine transport in via proton symport	Membrane	ADN[e] + H[e] -> ADN[c] + H[c]	Transport, Extracellular		
alpha-ketoglutarate/malate transporter	Membrane	AKG[c] + MAL[e] <-> AKG[e] + MAL[c]	Transport, Extracellular		
2-oxoglutarate reversible transport via symport	Membrane	AKG[e] + H[e] <-> AKG[c] + H[c]	Transport, Extracellular		

L-alanine reversible transport via proton symport	Membrane	ALA[e] + H[e] <-> ALA[c] + H[c]	Transport, Extracellular	
allantoin uniport	Membrane	alltn[e] -> alltn[c]	Transport, Extracellular	
allantoate uniport	Membrane	alltt[e] -> alltt[c]	Transport, Extracellular	
D-arabinose reversible transport	Membrane	arab-D[e] <-> arab-D[c]	Transport, Extracellular	
L-arabinoase extracellular transport	Membrane	arab-L[e] <-> arab-L[c]	Transport, Extracellular	
L-arganine reversible transport via proton symport	Membrane	ARG[e] + H[e] <-> ARG[c] + H[c]	Transport, Extracellular	
L-asparagine reversible transport via proton symport	Membrane	ASN[e] + H[e] <-> ASN[c] + H[c]	Transport, Extracellular	
L-aspartate reversible transport via proton symport	Membrane	ASP[e] + H[e] <-> ASP[c] + H[c]	Transport, Extracellular	
ATPaseic	Membrane	ATP[c] + H2O[c] -> ADP[c] + H[e] + Pi[c]	Transport, Extracellular	
Biotin uptake	Membrane	btn[e] + H[e] -> btn[c] + H[c]	Transport, Extracellular	
choline transport via proton symport	Membrane	CHOL[e] + H[e] -> CHOL[c] + H[c]	Transport, Extracellular	
citrate reversible transport via symport	Membrane	CIT[e] + H[e] <-> CIT[c] + H[c]	Transport, Extracellular	
CO2 transporter via diffusion	Membrane	CO2[e] <-> CO2[c]	Transport, Extracellular	
cytosine transport in via proton symport	Membrane	csn[e] + H[e] -> csn[c] + H[c]	Transport, Extracellular	
L-cysteine reversible transport via proton symport	Membrane	CYS[e] + H[e] <-> CYS[c] + H[c]	Transport, Extracellular	
cytidine transport in via proton symport	Membrane	cytd[e] + H[e] -> cytd[c] + H[c]	Transport, Extracellular	
D-lactate transport via proton symport	Membrane	H[e] + dLAC[e] <-> H[c] + dLAC[c]	Transport, Extracellular	
deoxyadenosine transport in via proton symport	Membrane	dad-2[e] + H[e] -> dad-2[c] + H[c]	Transport, Extracellular	
deoxycytidine transport in via proton symport	Membrane	dcyt[e] + H[e] -> dcyt[c] + H[c]	Transport, Extracellular	
deoxyguanosine transport in via proton symport	Membrane	dGSN[e] + H[e] -> dGSN[c] + H[c]	Transport, Extracellular	
deoxyinosine transport in via proton symport	Membrane	din[e] + H[e] -> din[c] + H[c]	Transport, Extracellular	
dTTP reversible uniport	Membrane	dTTP[e] <-> dTTP[c]	Transport, Extracellular	
deoxyURIdine transport in via proton symport	Membrane	dURI[e] + H[e] -> dURI[c] + H[c]	Transport, Extracellular	
ethanol reversible transport	Membrane	ETOH[e] <-> ETOH[c]	Transport, Extracellular	
iron (II) transport	Membrane	fe2[e] -> fe2[c]	Transport, Extracellular	
formate transport via diffusion	Membrane	for[e] <-> for[c]	Transport, Extracellular	
D-fructose transport in via proton symport	Membrane	fru[e] + H[e] -> fru[c] + H[c]	Transport, Extracellular	
D-galactose transport in via proton symport	Membrane	gal[e] + H[e] -> gal[c] + H[c]	Transport, Extracellular	
Glycoaldehydye reversible transport	Membrane	gcald[e] <-> gcald[c]	Transport, Extracellular	
glucose transport (uniport)	Membrane	GLC[e] -> GLC[c]	Transport, Extracellular	SPCC548.07C<br>SPAC1F8.01<br>SPBC4B4.08<br>SPBC1683.08<br>SPCC1235.13<br>SPCC1235.14<br>SPCC548.06c
L-glutamine reversible transport via proton symport	Membrane	GLN[e] + H[e] <-> GLN[c] + H[c]	Transport, Extracellular	
L-glutamate transport via proton symport	Membrane	GLU[e] + H[e] <-> GLU[c] + H[c]	Transport, Extracellular	
glyoxylate transport	Membrane	GLX[c] <-> GLX[e]	Transport, Extracellular	
glycerol transport via channel	Membrane	GLYC[c] <-> GLYC[e]	Transport, Extracellular	
glycerol transport via symport	Membrane	GLYC[e] + H[e] -> GLYC[c] + H[c]	Transport, Extracellular	
glycine reversible transport via proton symport	Membrane	GLY[e] + H[e] <-> GLY[c] + H[c]	Transport, Extracellular	
guanosine transport in via proton symport	Membrane	GSN[e] + H[e] -> GSN[c] + H[c]	Transport, Extracellular	
oxidized glutathione uniport	Membrane	GTHox[e] -> GTHox[c]	Transport, Extracellular	
glutathione transport	Membrane	GTHrd[e] -> GTHrd[c]	Transport, Extracellular	
guanine reversible transport via proton symport	Membrane	GUA[e] + H[e] <-> GUA[c] + H[c]	Transport, Extracellular	
H2O transport via diffusion	Membrane	H2O[e] <-> H2O[c]	Transport, Extracellular	
Hexadecanoate (n-C16:0) transport in via uniport	Membrane	C160[e] -> C160[c]	Transport, Extracellular	
hexadecenoate (n-C16:1) transport in via uniport	Membrane	C161[e] -> C161[c]	Transport, Extracellular	
L-histidine reversible transport via proton symport	Membrane	H[e] + HIS[e] <-> H[c] + HIS[c]	Transport, Extracellular	
hypoxanthine reversible transport via proton symport	Membrane	H[e] + HXAN[e] <-> H[c] + HXAN[c]	Transport, Extracellular	
L-isoleucine reversible transport via proton symport	Membrane	H[e] + ILE[e] <-> H[c] + ILE[c]	Transport, Extracellular	
Tryptophol transport (extracellular	Membrane	ind3etH[c] <-> ind3etH[e]	Transport, Extracellular	
potassium reversible transport via proton symport	Membrane	H[e] + k[e] <-> H[c] + k[c]	Transport, Extracellular	SPAC105.01C
L-lactate reversible transport via proton symport	Membrane	H[e] + LAC[e] <-> H[c] + LAC[c]	Transport, Extracellular	
L-leucine reversible transport via proton symport	Membrane	H[e] + LEU[e] <-> H[c] + LEU[c]	Transport, Extracellular	
L-lysine reversible transport via proton symport	Membrane	H[e] + LYS[e] <-> H[c] + LYS[c]	Transport, Extracellular	
L-malate reversible transport via proton symport	Membrane	H[e] + MAL[e] <-> H[c] + MAL[c]	Transport, Extracellular	
maltose transport in via proton symport	Membrane	H[e] + MALT[e] -> H[c] + MALT[c]	Transport, Extracellular	
D-mannose transport in via proton symport	Membrane	H[e] + man[e] -> H[c] + man[c]	Transport, Extracellular	
L-methionine reversible transport via proton symport	Membrane	H[e] + MET[e] <-> H[c] + MET[c]	Transport, Extracellular	
Nicotinic acid transport	Membrane	nac[e] <-> nac[c]	Transport, Extracellular	
NADP transporter	Membrane	NADP[c] <-> NADP[e]	Transport, Extracellular	
sodium proton antiporter (H:NA is 1:1)	Membrane	H[e] + na1[c] <-> H[c] + na1[e]	Transport, Extracellular	SPAC977.10<br>SPAC15A10.06<br>SPAC3A11.09
N,N-bisformyl-dityrosine transport (extracellular)	Membrane	Nbfortyr[c] -> Nbfortyr[e]	Transport, Extracellular	
ammonia reversible transport	Membrane	NH4[e] <-> NH4[c]	Transport, Extracellular	SPCPB1C11.01<br>SPAC664.14<br>SPAC2E1P3.02c
nmntp	Membrane	H[e] + nmn[e] -> H[c] + nmn[c]	Transport, Extracellular	
o2 transport (diffusion)	Membrane	O2[e] <-> O2[c]	Transport, Extracellular	
Oxaloacetate transport	Membrane	OAA[c] <-> OAA[e]	Transport, Extracellular	
C080decanoate (n-C18:0) transport in via uniport	Membrane	C180[e] -> C180[c]	Transport, Extracellular	
C080decenoate (n-C18:1) transport in via uniport	Membrane	C181[e] -> C181[c]	Transport, Extracellular	
C080decynoate (n-C18:2) transport in via uniport	Membrane	C182[e] -> C182[c]	Transport, Extracellular	
ornithine reversible transport in via proton symport	Membrane	H[e] +ORN[e] <-> H[c] +ORN[c]	Transport, Extracellular	
Phenylacetaldehyde transport (extracellular)	Membrane	PACALD[c] <-> PACALD[e]	Transport, Extracellular	
PAP reversible uniport	Membrane	PAP[e] <-> PAP[c]	Transport, Extracellular	
peptide transport in via proton symport	Membrane	H[e] + PEPD[e] -> H[c] + PEPD[c]	Transport, Extracellular	
Phenethyl acetate transport (extracellular)	Membrane	PHEAC[c] -> PHEAC[e]	Transport, Extracellular	
L-phenylalanine reversible transport via proton symport	Membrane	H[e] + PHE[e] <-> H[c] + PHE[c]	Transport, Extracellular	
phosphate reversible transport via symport	Membrane	H[e] + Pi[e] <-> H[c] + Pi[c]	Transport, Extracellular	SPAC23D3.12<br>SPAC23D3.12<br>SPBC1683.01<br>SPCC2H8.02<br>SPBC8E4.01C<br>SPBC3B8.04c<br>SPAC23D3.12
Pantothenate reversible transport via proton symport	Membrane	H[e] + pnto-R[e] <-> H[c] + pnto-R[c]	Transport, Extracellular	
L-proline reversible transport via proton symport	Membrane	H[e] + PRO[e] <-> H[c] + PRO[c]	Transport, Extracellular	
Pyruvate exchange, diffusion	Membrane	PYR[c] -> PYR[e]	Transport, Extracellular	

pyruvate transport in via proton symport	Membrane	H[e] + PYR[e] -> H[c] + PYR[c]	Transport, Extracellular	
riboflavin transport in via proton symport	Membrane	H[e] + ribflv[e] -> H[c] + ribflv[c]	Transport, Extracellular	
ribose transport in via proton symporter	Membrane	H[e] + rib-D[e] -> H[c] + rib-D[c]	Transport, Extracellular	
D-sorbitol transport via passive diffusion	Membrane	sbt-D[e] <-> sbt-D[c]	Transport, Extracellular	
L-sorbitol transport via passive diffusion	Membrane	sbt-L[e] <-> sbt-L[c]	Transport, Extracellular	
L-serine reversible transport via proton symport	Membrane	H[e] + SER[e] <-> H[c] + SER[c]	Transport, Extracellular	
sulfite transport (efflux to extracellular)	Membrane	SO3[c] -> SO3[e]	Transport, Extracellular	
sulfate uniport	Membrane	SO4[e] -> SO4[c]	Transport, Extracellular	SPAC24H6.11C<br>SPCC320.05<br>SPBC3H7.02<br>SPAC869.05C
spermidine transport in via proton antiport	Membrane	H[c] + spmd[e] -> H[e] + spmd[c]	Transport, Extracellular	
spermidine excretion (cytosol to extracellular)	Membrane	spmd[c] -> spmd[e]	Transport, Extracellular	
spermine transport via proton antiport	Membrane	H[c] + sprm[e] -> H[e] + sprm[c]	Transport, Extracellular	
L-sorbose reversible transport	Membrane	srb-L[e] <-> srb-L[c]	Transport, Extracellular	
succinate transport via proton symport	Membrane	H[e] + SUCC[e] <-> H[c] + SUCC[c]	Transport, Extracellular	
sucrose transport in via proton symport	Membrane	H[e] + sucr[e] -> H[c] + sucr[c]	Transport, Extracellular	SPAC2F3.08
taURIne transport	Membrane	taur[c] <-> taur[e]	Transport, Extracellular	
thymidine transport in via proton symport	Membrane	H[e] + THYMD[e] -> H[c] + THYMD[c]	Transport, Extracellular	
Thiamine transport in via proton symport	Membrane	H[e] + THM[e] -> H[c] + THM[c]	Transport, Extracellular	
L-threonine reversible transport via proton symport	Membrane	H[e] + THR[e] <-> H[c] + THR[c]	Transport, Extracellular	
thymine reversible transport via proton antiport	Membrane	H[e] + THYM[c] <-> H[c] + THYM[e]	Transport, Extracellular	
trehalose transport in via proton symporter	Membrane	H[e] + TRE[e] -> H[c] + TRE[c]	Transport, Extracellular	
L-tryptophan reversible transport via proton symport	Membrane	H[e] + TRP[e] <-> H[c] + TRP[c]	Transport, Extracellular	
fatty acid transport	Membrane	C140[e] <-> C140[c]	Transport, Extracellular	
L-tyrosine reversible transport via proton symport	Membrane	H[e] + TYR[e] <-> H[c] + TYR[c]	Transport, Extracellular	
uracil transport in via proton symport	Membrane	H[e] + ura[e] -> H[c] + ura[c]	Transport, Extracellular	SPAC1399.03<br>SPAC29B12.14C
urea reversible transport via proton symport (2 H+)	Membrane	(2 na[e] + urea[e] <-> 2 na[c] + urea[c]	Transport, Extracellular	SPBC23G7.13C<br>SPAC869.03C
URIdine transport in via proton symport	Membrane	H[e] + URI[e] -> H[c] + URI[c]	Transport, Extracellular	
L-valine reversible transport via proton symport	Membrane	H[e] + VAL[e] <-> H[c] + VAL[c]	Transport, Extracellular	
xanthine reversible transport	Membrane	XAN[e] <-> XAN[c]	Transport, Extracellular	
xanthosine transport in via proton symport	Membrane	H[e] + xtsn[e] -> H[c] + xtsn[c]	Transport, Extracellular	
D-xylose reversible transport	Membrane	xyl-D[e] <-> xyl-D[c]	Transport, Extracellular	
Xylitol transport via passive diffusion	Membrane	xylt[e] <-> xylt[c]	Transport, Extracellular	
zymosterol reversible transport	Membrane	zymst[e] <-> zymst[c]	Transport, Extracellular	
MFS myo-inositol transporter	Membrane	inost[e] <-> inost [c]	Transport, Extracellular	SPAC20G8.03<br>SPAC4F8.15
myo-inositol transporter Itr1	Membrane	inost[e] <-> inost [c]	Transport, Extracellular	SPAC4F8.15<br>SPAC20G8.03
CO2 Golgi transport	Membrane	CO2[c] <-> CO2[g]	Transport, Golgi Apparatus	
GDP-mannose antiport	Membrane	GDPmann[c] + GMP[g] -> GDPmann[g] + GMP[c]	Transport, Golgi Apparatus	SPAC144.18
GDP Golgi transport via proton anitport	Membrane	GDP[g] + H[c] <-> GDP[c] + H[g]	Transport, Golgi Apparatus	
phosphatidylethanolamine Golgi transport	Membrane	pe [c] <-> pe [g]	Transport, Golgi Apparatus	
phosphatidylserine Golgi transport	Membrane	ps [c] <-> ps [g]	Transport, Golgi Apparatus	
UDPgalactose transport (Golgi apparatus)	Membrane	UDPgal[c] -> UDPgal[g]	Transport, Golgi Apparatus	
Diphosphate Mitochondrial transport	Membrane	PPi[c] <-> PPi[m]	Transport, Mitochondrial	
2-Dehydro-3-deoxy-D-arabino-heptonate7-phohsphate mitochondrial transport via diffusion	Membrane	2dda7p[c] <-> 2dda7p[m]	Transport, Mitochondrial	
2-Dehydropantoate mitochondrial transport	Membrane	2DHP[c] <-> 2DHP[m]	Transport, Mitochondrial	
2-Methylbutanal transport (mitochondrial)	Membrane	2mbald[c] <-> 2mbald[m]	Transport, Mitochondrial	
2-methyl-1-butanol transport (mitochondrial)	Membrane	2mbtoH[c] <-> 2mbtoH[m]	Transport, Mitochondrial	
2-methylpropanal transport (mitochondrial)	Membrane	2mppal[c] <-> 2mppal[m]	Transport, Mitochondrial	
2obut transporter (mitochondrial)	Membrane	2obut[c] <-> 2obut[m]	Transport, Mitochondrial	
2-oxoadipate transport out of mitochondria via diffusion	Membrane	2oxoADP[m] -> 2oxoADP[c]	Transport, Mitochondrial	
2-phenylethanol reversible transport	Membrane	2PHETOH[m] <-> 2PHETOH[c]	Transport, Mitochondrial	
3-(4-hydroxyphenyl)pyruvate mitochondrial transport via proton symport	Membrane	34Hpp[c] + H[c] <-> 34Hpp[m] + H[m]	Transport, Mitochondrial	
2-Isopropylmalate transport, diffusion	Membrane	3c3Hmp[c] <-> 3c3Hmp[m]	Transport, Mitochondrial	
3-Carboxy-4-methyl-2-oxopentanoate transport, diffusion	Membrane	3c4MOP[c] <-> 3c4MOP[m]	Transport, Mitochondrial	
3-Hexaprenyl-4,5-dihydroxybenzoate transport	Membrane	3H45DHBZ[c] <-> 3H45DHBZ[m]	Transport, Mitochondrial	
3-methylbutanal transport (mitochondrial)	Membrane	3mbald[c] <-> 3mbald[m]	Transport, Mitochondrial	
3-methyl-2-oxobutanoate transport, diffusion	Membrane	3MOB[c] <-> 3MOB[m]	Transport, Mitochondrial	
3-Methyl-2-oxopentanoate transport, diffusion	Membrane	3MOP[c] <-> 3MOP[m]	Transport, Mitochondrial	
3-C080prenyl-4-hydroxybenzoate mitochondrial transport	Membrane	O4HBZ[c] <-> O4HBZ[m]	Transport, Mitochondrial	
4-aminobutanal mitochondrial transport via diffusion	Membrane	4abutn[c] <-> 4abutn[m]	Transport, Mitochondrial	
4-aminobutanoate mitochondrial transport via diffusion	Membrane	4abut[c] <-> 4abut[m]	Transport, Mitochondrial	
4-Aminobenzoate mitochondrial transport via diffusion	Membrane	PABA[c] <-> PABA[m]	Transport, Mitochondrial	
4-hydroxy-2-oxoglutarate mitochondrial transport via diffusion	Membrane	4H2Oglt[c] <-> 4H2Oglt[m]	Transport, Mitochondrial	
4-Hydroxybenzoate mitochondrial transport	Membrane	4Hbz[c] <-> 4Hbz[m]	Transport, Mitochondrial	
trans-4-hydroxy-L-proline mitochondrial transport via diffusion	Membrane	4HPROT[c] <-> 4HPROT[m]	Transport, Mitochondrial	
5-Aminolevulinate mitochondrial transport	Membrane	5aop[c] <-> 5aop[m]	Transport, Mitochondrial	
acetaldehyde mitochondrial diffusion	Membrane	ACAL[m] <-> ACAL[c]	Transport, Mitochondrial	
acetate transport	Membrane	AC[c] <-> AC[m]	Transport, Mitochondrial	
adenine reversible transport	Membrane	ADE[c] <-> ADE[m]	Transport, Mitochondrial	
S-adenosyl-L-homocysteine reversible transport	Membrane	SAH[c] <-> SAH[m]	Transport, Mitochondrial	
Alanine transport from mitochondia to cytoplasm	Membrane	ALA[m] -> ALA[c]	Transport, Mitochondrial	
S-Adenosyl-L-methionine reversible transport	Membrane	SAM[c] <-> SAM[m]	Transport, Mitochondrial	
arginine mitochondrial transport via proton symport	Membrane	ARG[c] + H[c] <-> ARG[m] + H[m]	Transport, Mitochondrial	
asparagine mitochondrial transport via proton transport	Membrane	ASN[c] + H[c] <-> ASN[m] + H[m]	Transport, Mitochondrial	
Aspartate-glutamate transporter	Membrane	ASP[m] + GLU[c] -> ASP[c] + GLU[m]	Transport, Mitochondrial	
aspartate mitochondrial transport via proton symport	Membrane	ASP[c] + H[c] <-> ASP[m] + H[m]	Transport, Mitochondrial	
ADP/ATP transporter	Membrane	ADP[c] + ATP[m] + H[c] -> ADP[m] + ATP[c] + H[m]	Transport, Mitochondrial	SPBC530.10C
citrate transport	Membrane	CIT[c] + MAL[m] <-> CIT[m] + MAL[c]	Transport, Mitochondrial	
citrate transport	Membrane	CIT[c] + PEP[m] <-> CIT[m] + PEP[c]	Transport, Mitochondrial	
citrate transport	Membrane	CIT[c] + ICIT[m] <-> CIT[m] + ICIT[c]	Transport, Mitochondrial	

CO2 transport (diffusion)	Membrane	CO2[c] <-> CO2[m]	Transport, Mitochondrial	
CoA transporter (mitochondrial)	Membrane	CoA[c] -> CoA[m]	Transport, Mitochondrial	
CTP/CMP antiport	Membrane	CMP[m] + CTP[c] + 2 H[c] -> CMP[c] + CTP[m] + 2 H[m]	Transport, Mitochondrial	SPAC688.09
D-lactate/pyruvate antiport	Membrane	dLAC[c] + PYR[m] <-> dLAC[m] + PYR[c]	Transport, Mitochondrial	
D-lactate transport	Membrane	H[c] + dLAC[c] <-> H[m] + dLAC[m]	Transport, Mitochondrial	
dihydroxyacetone phosphate transport	Membrane	DHAP[m] -> DHAP[c]	Transport, Mitochondrial	
dihydrofolate reversible mitochondrial transport	Membrane	DHF[c] <-> DHF[m]	Transport, Mitochondrial	
dhnpt mitochondrial transport	Membrane	DHNPT[c] <-> DHNPT[m]	Transport, Mitochondrial	
Dihydropteroate mitochondrial transport via diffusion	Membrane	DHPT[c] <-> DHPT[m]	Transport, Mitochondrial	
L-erythro-4-hydroxyglutamate mitochondrial transport via diffusion	Membrane	e4HGLU[c] <-> e4HGLU[m]	Transport, Mitochondrial	
D-erythrose 4-phosphate mtiochondrial transport via diffusion	Membrane	E4P[c] <-> E4P[m]	Transport, Mitochondrial	
ethanol transport to mitochondria (diffusion)	Membrane	ETOH[c] <-> ETOH[m]	Transport, Mitochondrial	
fatty-acyl-ACP mitochondrial transport	Membrane	C120ACP[m] -> C120ACP[c]	Transport, Mitochondrial	
fatty-acyl-ACP mitochondrial transport	Membrane	C140ACP[m] -> C140ACP[c]	Transport, Mitochondrial	
fatty-acyl-ACP mitochondrial transport	Membrane	C141ACP[m] -> C141ACP[c]	Transport, Mitochondrial	
fatty-acyl-ACP mitochondrial transport	Membrane	C160ACP[m] -> C160ACP[c]	Transport, Mitochondrial	
fatty-acyl-ACP mitochondrial transport	Membrane	C161ACP[m] -> C161ACP[c]	Transport, Mitochondrial	
fatty-acyl-ACP mitochondrial transport	Membrane	C180ACP[m] -> C180ACP[c]	Transport, Mitochondrial	
fatty-acyl-ACP mitochondrial transport	Membrane	C181ACP[m] -> C181ACP[c]	Transport, Mitochondrial	
fatty-acyl-ACP mitochondrial transport	Membrane	C182ACP[m] -> C182ACP[c]	Transport, Mitochondrial	
FAD/FMN antiport	Membrane	fad[c] + fmn[m] -> fad[m] + fmn[c]	Transport, Mitochondrial	SPBC27B12.09C
iron (II) uptake (mitochondrial)	Membrane	fe2[c] -> fe2[m]	Transport, Mitochondrial	
iron (II) transport	Membrane	fe2[m] -> fe2[c]	Transport, Mitochondrial	
formate mitochondrial transport	Membrane	for[m] -> for[c]	Transport, Mitochondrial	
fumarate reductaseic/mitochondrial	Membrane	FADH2[m] + FUM[c] -> fad[m] + SUCC[c]	Transport, Mitochondrial	SPAC17A2.05
farnesyl diphosphate transport (mitochondrial)	Membrane	FRPP[c] <-> FRPP[m]	Transport, Mitochondrial	
glycoaldehyde mitochondrial transport	Membrane	gcald[c] <-> gcald[m]	Transport, Mitochondrial	
L-glutamate transport into mitochondria via hydroxide ion antiport	Membrane	GLU[c] + oH1[m] -> GLU[m] + oH1[c]	Transport, Mitochondrial	
Glutamate transport (uniporter)	Membrane	GLU[c] -> GLU[m]	Transport, Mitochondrial	
glycerol-3-phosphate shuttle	Membrane	GLYC3p[c] -> GLYC3p[m]	Transport, Mitochondrial	
glycine mitochondrial transport via proton symport	Membrane	GLY[c] + H[c] <-> GLY[m] + H[m]	Transport, Mitochondrial	
guanosine mitochondrial transport via proton symport	Membrane	GSN[c] + H[c] <-> GSN[m] + H[m]	Transport, Mitochondrial	
GTP/GDP translocase (electroneutral)	Membrane	GDP[m] + GTP[c] + H[c] -> GDP[c] + GTP[m] + H[m]	Transport, Mitochondrial	
guanine mitochondrial transport via diffusion	Membrane	GUA[c] <-> GUA[m]	Transport, Mitochondrial	
H2O transport	Membrane	H2O[c] <-> H2O[m]	Transport, Mitochondrial	
all-trans-hexaprenyl diphosphate transport	Membrane	HXPP[c] <-> HXPP[m]	Transport, Mitochondrial	
histidine mitochondrial transport via proton symport	Membrane	H[m] + HIS[m] <-> H[c] + HIS[c]	Transport, Mitochondrial	
Hydroxymethylglutaryl-CoA reversible mitochondrial transport	Membrane	HmgCoA[c] <-> HmgCoA[m]	Transport, Mitochondrial	
isoamyl alcohol transport (mitochondrial)	Membrane	IAMOH[c] <-> IAMOH[m]	Transport, Mitochondrial	
isobutyl alcohol transport (mitochondrial)	Membrane	IBUTOH[c] <-> IBUTOH[m]	Transport, Mitochondrial	
indole-3-acetaldehyde mitochondrial transport via diffusion	Membrane	id3acald[c] <-> id3acald[m]	Transport, Mitochondrial	
Isoleucine transport from mitochondria to cytosol	Membrane	ILE[m] -> ILE[c]	Transport, Mitochondrial	
indole-3-acetate mitochondrial transport via diffusion	Membrane	ind3ac[c] <-> ind3ac[m]	Transport, Mitochondrial	
Tryptophol transport (mitochondrial)	Membrane	ind3etH[c] <-> ind3etH[m]	Transport, Mitochondrial	
Isopentenyl diphosphate transport	Membrane	IPPP[c] <-> IPPP[m]	Transport, Mitochondrial	
L-lactate transport	Membrane	H[c] + LAC[c] <-> H[m] + LAC[m]	Transport, Mitochondrial	
Lysine mitochondrial transport via proton symport	Membrane	H[c] + LYS[c] <-> H[m] + LYS[m]	Transport, Mitochondrial	
malate transport	Membrane	MAL[c] + Pi[m] <-> MAL[m] + Pi[c]	Transport, Mitochondrial	
methionine mitochondrial transport via proton symport	Membrane	H[m] + MET[m] <-> H[c] + MET[c]	Transport, Mitochondrial	
NH3 mitochondrial transport	Membrane	NH4[c] <-> NH4[m]	Transport, Mitochondrial	
NMN mitochondrial transport via proton symport	Membrane	H[c] + nmn[c] <-> H[m] + nmn[m]	Transport, Mitochondrial	
O2 transport (diffusion)	Membrane	O2[c] <-> O2[m]	Transport, Mitochondrial	
oxaloacetate transport	Membrane	H[c] + OAA[c] <-> H[m] + OAA[m]	Transport, Mitochondrial	SPAC139.02C
ornithine mitochondrial transport via proton antiport	Membrane	H[c] +ORN[m] <-> H[m] +ORN[c]	Transport, Mitochondrial	
2-oxodicarboylate transporter	Membrane	AKG[m] + oxag[c] <-> AKG[c] + oxag[m]	Transport, Mitochondrial	
Phenylacetaldehyde transport (mitochondrial)	Membrane	PACALD[c] <-> PACALD[m]	Transport, Mitochondrial	
panthetheine 4'-phosphate reversible mitochondrial transport	Membrane	pan4p[c] <-> pan4p[m]	Transport, Mitochondrial	
pantothenate mitochondrial transport	Membrane	pant-R[c] <-> pant-R[m]	Transport, Mitochondrial	
Adenosine 3',5'-bisphosphate mitochondrial transport	Membrane	PAP[c] <-> PAP[m]	Transport, Mitochondrial	
phosphatidate reversible transport	Membrane	pa [c] <-> pa [m]	Transport, Mitochondrial	
all-trans-Pentaprenyl diphosphate transport	Membrane	PNPP[c] <-> PNPP[m]	Transport, Mitochondrial	
phosphatidylethanolamine mitochondrial transport	Membrane	pe [c] <-> pe [m]	Transport, Mitochondrial	
Phenylalanine mitochondrial transport via proton symport	Membrane	H[m] + PHE[m] <-> H[c] + PHE[c]	Transport, Mitochondrial	
phosphate transporter	Membrane	H[c] + Pi[c] <-> H[m] + Pi[m]	Transport, Mitochondrial	SPBC1703.13C
phosphate transport via hydroxide ion symport	Membrane	oH1[m] + Pi[c] <-> oH1[c] + Pi[m]	Transport, Mitochondrial	
protoporphyrinogen IX mitochondrial transport	Membrane	pppg9[c] <-> pppg9[m]	Transport, Mitochondrial	
L-proline transport	Membrane	PRO[c] <-> PRO[m]	Transport, Mitochondrial	
PRPP reversible transport	Membrane	PRPP[c] <-> PRPP[m]	Transport, Mitochondrial	
phosphatidylserine mitochondrial transport	Membrane	ps [c] <-> ps [m]	Transport, Mitochondrial	
pyruvate mitochondrial transport via proton symport	Membrane	H[c] + PYR[c] <-> H[m] + PYR[m]	Transport, Mitochondrial	
Quinolinate reversible mitochondrial transport	Membrane	Quln[c] <-> Quln[m]	Transport, Mitochondrial	
Riboflavin reversible mitochondrial transport	Membrane	ribflv[c] <-> ribflv[m]	Transport, Mitochondrial	
serine mitochondrial transport via proton symport	Membrane	H[c] + SER[c] <-> H[m] + SER[m]	Transport, Mitochondrial	
succinate transport	Membrane	Pi[m] + SUCC[c] -> Pi[c] + SUCC[m]	Transport, Mitochondrial	
succinate-fumarate transport	Membrane	FUM[m] + SUCC[c] -> FUM[c] + SUCC[m]	Transport, Mitochondrial	
Thiamine diphosphate transport	Membrane	THMPP[c] -> THMPP[m]	Transport, Mitochondrial	
threonine mitochondrial transport via proton symport	Membrane	H[c] + THR[c] <-> H[m] + THR[m]	Transport, Mitochondrial	
tryptophan mitochondrial transport via proton symport	Membrane	H[c] + TRP[c] <-> H[m] + TRP[m]	Transport, Mitochondrial	
tyrosine mitochondrial transport via proton symport	Membrane	H[c] + TYR[c] <-> H[m] + TYR[m]	Transport, Mitochondrial	
UTP/UMP antiport	Membrane	(2 H[c] + UMP[m] + UTP[c] -> 2 H[m] + UMP[c] + UTP[m]	Transport, Mitochondrial	SPAC688.09

Valine reversible mitochondrial transport via proton symport	Membrane	H[c] + VAL[c] <-> H[m] + VAL[m]	Transport, Mitochondrial
acetyl-CoA transport	Membrane	ACCoA[c] <-> ACCoA[n]	Transport, Nuclear
2-oxoglutarate nuclear transport via proton symport	Membrane	AKG[c] + H[c] <-> AKG[n] + H[n]	Transport, Nuclear
AMP transport via diffusion (cytosol to nucleus)	Membrane	AMP[n] <-> AMP[c]	Transport, Nuclear
aspartate nuclear transport via proton symport	Membrane	ASP[c] + H[c] <-> ASP[n] + H[n]	Transport, Nuclear
L-aspartate nuclear transport via diffusion	Membrane	ASP[n] <-> ASP[c]	Transport, Nuclear
N-carbomoyl-L-aspartate transport, diffusion	Membrane	cbasp[n] <-> cbasp[c]	Transport, Nuclear
carbamoyl phosphate nuclear transport via diffusion	Membrane	cbp[c] <-> cbp[n]	Transport, Nuclear
CDP nuclear transport	Membrane	CDP[c] <-> CDP[n]	Transport, Nuclear
CO2 nuclear transport via diffusion	Membrane	CO2[n] <-> CO2[c]	Transport, Nuclear
coenzyme A transport	Membrane	CoA[c] <-> CoA[n]	Transport, Nuclear
DADP nuclear transport	Membrane	dADP[c] <-> dADP[n]	Transport, Nuclear
dCDP nuclear transport	Membrane	dCDP[c] <-> dCDP[n]	Transport, Nuclear
dGDP nuclear transport	Membrane	dGDP[c] <-> dGDP[n]	Transport, Nuclear
dUMP nuclear transport	Membrane	dUMP[c] <-> dUMP[n]	Transport, Nuclear
GDP nuclear transport	Membrane	GDP[c] <-> GDP[n]	Transport, Nuclear
glutamine nuclear transport via proton symport	Membrane	GLN[c] + H[c] <-> GLN[n] + H[n]	Transport, Nuclear
glutamate nuclear transport via proton symport	Membrane	GLU[c] + H[c] <-> GLU[n] + H[n]	Transport, Nuclear
hydrogen peroxide nuclear transport	Membrane	H2O2[c] <-> H2O2[n]	Transport, Nuclear
H2O transport	Membrane	H2O[n] <-> H2O[c]	Transport, Nuclear
bicarbonate nuclear transport via diffusion	Membrane	HCO3[c] <-> HCO3[n]	Transport, Nuclear
4-Aminobenzoate mitochondrial transport via diffusion	Membrane	PABA[c] <-> PABA[n]	Transport, Nuclear
1D-myo-Inositol 1,4,5-trisphosphate nuclear transport via diffusion	Membrane	mi145p[c] <-> mi145p[n]	Transport, Nuclear
inositol hexakisphosphate nuclear transport (diffusion)	Membrane	minoHp[c] <-> minoHp[n]	Transport, Nuclear
NAD transport through pores	Membrane	NAD[n] <-> NAD[c]	Transport, Nuclear
ammonia nuclear transport	Membrane	NH4[c] <-> NH4[n]	Transport, Nuclear
phosphate nuclear transport via proton symport	Membrane	H[c] + Pi[c] <-> H[n] + Pi[n]	Transport, Nuclear
phosphatidyl-1D-myo-insoitol nuclear transport	Membrane	ptd1ino [c] <-> ptd1ino [n]	Transport, Nuclear
phosphatidyl-1D-myo-4-inositol nuclear transport	Membrane	ptd4ino [c] <-> ptd4ino [n]	Transport, Nuclear
UMP nuclear transport	Membrane	UMP[c] <-> UMP[n]	Transport, Nuclear
3-(4-hydroxyphenyl)pyruvate peroxisomal transport via proton symport	Membrane	34Hpp[c] + H[c] <-> 34Hpp[x] + H[x]	Transport, Peroxisomal
4-hydroxy-2-oxoglutarate peroxisomal transport via diffusion	Membrane	4H2Oglt[c] <-> 4H2Oglt[x]	Transport, Peroxisomal
acetate transport	Membrane	ac[c] <-> ac[x]	Transport, Peroxisomal
AKG transporter, peroxisome	Membrane	AKG[c] <-> AKG[x]	Transport, Peroxisomal
aspartate-glutamate peroxisomal shuttle	Membrane	ASP[c] + GLU[x] <-> ASP[x] + GLU[c]	Transport, Peroxisomal
AMP/ATP transporter	Membrane	AMP[x] + ATP[c] + H[x] -> AMP[c] + ATP[x] + H[c]	Transport, Peroxisomal
ADP/ATP transporter	Membrane	ADP[x] + ATP[c] + H[x] -> ADP[c] + ATP[x] + H[c]	Transport, Peroxisomal
citrate/malate antiport into peroxisome	Membrane	CIT[x] + MAL[c] <-> CIT[c] + MAL[x]	Transport, Peroxisomal
citrate/isocitrate antiport into peroxisome	Membrane	CIT[c] + ICIT[x] <-> CIT[x] + ICIT[c]	Transport, Peroxisomal
CO2 peroxisomal transport	Membrane	CO2[c] <-> CO2[x]	Transport, Peroxisomal
cystathione peroxisomal transport	Membrane	cyst-L[c] <-> cyst-L[x]	Transport, Peroxisomal
L-erythro-4-hydroxyglutamate peroxisomal transport via diffusion	Membrane	e4HGLU[c] <-> e4HGLU[x]	Transport, Peroxisomal
fatty acid peroxisomal transport	Membrane	C100[c] -> C100[x]	Transport, Peroxisomal
fatty acid peroxisomal transport	Membrane	C120[c] -> C120[x]	Transport, Peroxisomal
fatty acid peroxisomal transport	Membrane	C141[c] -> C141[x]	Transport, Peroxisomal
fatty acid peroxisomal transport	Membrane	C160[c] -> C160[x]	Transport, Peroxisomal
fatty acid peroxisomal transport	Membrane	C140[c] -> C140[x]	Transport, Peroxisomal
fatty acid peroxisomal transport	Membrane	C161[c] -> C161[x]	Transport, Peroxisomal
fatty acid peroxisomal transport	Membrane	C080[c] -> C080[x]	Transport, Peroxisomal
glyoxylate transport	Membrane	GLX[c] <-> GLX[x]	Transport, Peroxisomal
H2O transport	Membrane	H2O[c] <-> H2O[x]	Transport, Peroxisomal
Homocysteine peroxisomal transport via proton symport	Membrane	H[c] + HCYS[c] <-> H[x] + HCYS[x]	Transport, Peroxisomal
malate/oxaloacetate shuttle	Membrane	MAL[x] + OAA[c] <-> MAL[c] + OAA[x]	Transport, Peroxisomal
ammonia peroxisomal transport	Membrane	NH4[c] <-> NH4[x]	Transport, Peroxisomal
NMN peroxisomal transport via proton symport	Membrane	H[c] + nmn[c] <-> H[x] + nmn[x]	Transport, Peroxisomal
phosphate peroxisomal transport via proton symport	Membrane	H[c] + Pi[c] <-> H[x] + Pi[x]	Transport, Peroxisomal
pyruvate peroxisomal transport via proton symport	Membrane	H[c] + PYR[c] <-> H[x] + PYR[x]	Transport, Peroxisomal
oxidized thioredoxin peroxisomal transport via diffusion	Membrane	TRDox[c] <-> TRDox[x]	Transport, Peroxisomal
reduced thioredoxin peroxisomal transport via diffusion	Membrane	TRDrd[c] <-> TRDrd[x]	Transport, Peroxisomal
tyrosine peroxisomal transport via proton symport	Membrane	H[c] + TYR[c] <-> H[x] + TYR[x]	Transport, Peroxisomal
L-arginine transport in via proton antiport (vacuolar)	Membrane	ARG[c] + H[v] -> ARG[v] + H[c]	Transport, Vacuolar
L-asparagine transport in via proton antiport (vacuolar)	Membrane	ASN[c] + H[v] -> ASN[v] + H[c]	Transport, Vacuolar
L-asparagine transport out via proton symport	Membrane	ASN[v] + H[v] -> ASN[c] + H[c]	Transport, Vacuolar
L-aspartate transport out via proton symport	Membrane	ASP[v] + H[v] -> ASP[c] + H[c]	Transport, Vacuolar
CO2 vacuolar transport	Membrane	CO2[c] <-> CO2[v]	Transport, Vacuolar
Glycogen vacuolar 'transport' via autophagy	Membrane	GLYCogen[c] -> GLYCogen[v]	Transport, Vacuolar
glucose transport	Membrane	GLC[c] <-> GLC[v]	Transport, Vacuolar
L-glutamine transport in via proton antiport (vacuolar)	Membrane	GLN[c] + H[v] -> GLN[v] + H[c]	Transport, Vacuolar
L-glutamine transport out via proton symport	Membrane	GLN[v] + H[v] -> GLN[c] + H[c]	Transport, Vacuolar
L-glutamate transport out via proton symport	Membrane	GLU[v] + H[v] -> GLU[c] + H[c]	Transport, Vacuolar
Reduced glutathione via ABC system (vacuolar)	Membrane	ATP[v] + GTHrd[c] + H2O[v] -> ADP[v] + GTHrd[v] + H[v] + Pi[v]	Transport, Vacuolar
H2O transport	Membrane	H2O[c] <-> H2O[v]	Transport, Vacuolar
L-histidine transport in via proton antiport (vacuolar)	Membrane	H[v] + HIS[c] -> H[c] + HIS[v]	Transport, Vacuolar
L-isoleucine transport in via proton antiport (vacuolar)	Membrane	H[v] + ILE[c] -> H[c] + ILE[v]	Transport, Vacuolar
L-isoleucine transport out via proton symport	Membrane	H[v] + ILE[v] -> H[c] + ILE[c]	Transport, Vacuolar
L-cystine transport via proton symport (vacuolar)	Membrane	Lcystin[v] + H[v] -> Lcystin[c] + H[c]	Transport, Vacuolar
L-leucine transport in via proton antiport (vacuolar)	Membrane	H[v] + LEU[c] -> H[c] + LEU[v]	Transport, Vacuolar
L-leucine transport out via proton symport	Membrane	H[v] + LEU[v] -> H[c] + LEU[c]	Transport, Vacuolar
L-lysine transport in via proton antiport (vacuolar)	Membrane	H[v] + LYS[c] -> H[c] + LYS[v]	Transport, Vacuolar
phosphatidylethanolamine vacuolar transport	Membrane	pe [c] <-> pe [v]	Transport, Vacuolar
phosphate vacuolar transport via proton symport	Membrane	H[c] + Pi[c] <-> H[v] + Pi[v]	Transport, Vacuolar

phosphatidylserine vacuolar transport	Membrane	ps [c] <-> ps [v]	Transport, Vacuolar		
Taurcholate via ABC system (vacuolar)	Membrane	ATP[v] + H2O[v] + tCHOLa[c] -> ADP[v] + H[v] + Pi[v] + tCHOLa[v]	Transport, Vacuolar		
trehalose vacuolar transport via proton symport	Membrane	H[c] + TRE[c] <-> H[v] + TRE[v]	Transport, Vacuolar		
L-tyrosine transport in via proton antiport (vacuolar)	Membrane	H[v] + TYR[c] -> H[c] + TYR[v]	Transport, Vacuolar		
L-tyrosine transport out via proton symport	Membrane	H[v] + TYR[v] -> H[c] + TYR[c]	Transport, Vacuolar		
Alanyl-tRNA synthetase	Cytosol	trnaALA + ALA + ATP -> ALAtrna + AMP + PPi	tRNA charging	SPAC23C11.09	
Arginyl-tRNA synthetase	Cytosol	trnaARG + ARG + ATP -> ARGtrna + AMP + PPi	tRNA charging	SPBC25B2.09c	
Arginyl-tRNA synthetase	Mitochondria	trnaARG + ARG + ATP -> ARGtrna + AMP + PPi	tRNA charging	SPBC25B2.09c	
Asparaginyl-tRNA synthetase	Cytosol	trnaASN + ASN + ATP -> ASNtrna + AMP + PPi	tRNA charging	SPBC1773.10c	
asparaginyl-tRNA synthetase, miotchondrial	Mitochondria	trnaASN + ASN + ATP -> ASNtrna + AMP + PPi	tRNA charging	SPBC1198.10c	
Aspartyl-tRNA synthetase	Cytosol	trnaASP + ASP + ATP -> ASPtrna + AMP + PPi	tRNA charging	SPCC1223.07c	
Aspartyl-tRNA synthetase	Mitochondria	trnaASP + ASP + ATP -> ASPtrna + AMP + PPi	tRNA charging	SPCC736.06	
Cysteinyl-tRNA synthetase	Cytosol	trnaCYS + ATP + CYS -> CYStrna + AMP + PPi	tRNA charging	SPAC29E6.06c	
Methionyl-tRNA formyltransferase	Mitochondria	METtrna + 10FTHF -> fMETtrna + H + THF	tRNA charging	SPAC1805.09c	
Glutaminyl-tRNA synthetase	Cytosol	trnaGLN + GLN + ATP -> GLNtrna + AMP + PPi	tRNA charging	SPBC342.02	
Glutamyl-tRNA synthetase	Cytosol	trnaGLU + GLU + ATP -> GLUtrna + AMP + PPi	tRNA charging	SPAC17A5.15c	
glutamyl-tRNA synthetase	Mitochondria	trnaGLU + GLU + ATP -> GLUtrna + AMP + PPi	tRNA charging	SPAPB1A10.11c	
Glycyl-tRNA synthetase	Cytosol	trnaGLY + GLY + ATP -> GLYtrna + AMP + PPi	tRNA charging	SPAC3F10.03	
Histidyl-tRNA synthetase	Cytosol	trnaHIS + HIS + ATP -> HIStrna + AMP + PPi	tRNA charging	SPBC2G2.12	
histidyl-tRNA synthetase	Mitochondria	trnaHIS + HIS + ATP -> HIStrna + AMP + PPi	tRNA charging	SPBC2G2.12	
Isoleucyl-tRNA synthetase	Cytosol	trnaILE + ILE + ATP -> ILEtrna + AMP + PPi	tRNA charging	SPBC8D2.06	
isoleucyl-tRNA synthetase	Mitochondria	trnaILE + ILE + ATP -> ILEtrna + AMP + PPi	tRNA charging	SPCC18B5.08c	
Leucyl-tRNA synthetase	Cytosol	trnaLEU + LEU + ATP -> AMP + LEUtrna + PPi	tRNA charging	SPAC26F1.13c	
leucyl-tRNA synthetase	Mitochondria	trnaLEU + LEU + ATP -> AMP + LEUtrna + PPi	tRNA charging	SPAC4G8.09	
Lysyl-tRNA synthetase	Cytosol	trnaLYS + LYS + ATP -> AMP + LYStrna + PPi	tRNA charging	SPBC17G9.03c	
Lysyl-tRNA synthetase	Mitochondria	trnaLYS + LYS + ATP -> AMP + LYStrna + PPi	tRNA charging	SPCC18.08	
Methionyl-tRNA synthetase	Cytosol	trnaMET + MET + ATP -> METtrna + AMP + PPi	tRNA charging	SPBC17A3.04c	
methionyl-tRNA synthetase	Mitochondria	trnaMET + MET + ATP -> METtrna + AMP + PPi	tRNA charging	SPAC27E2.06c	
Phenylalanyl-tRNA synthetase	Cytosol	trnaPHE + PHE + ATP -> PHEtrna + AMP + PPi	tRNA charging	SPAC23A1.12c+SPAC3G9.06	
phenylalanyl-tRNA synthetase	Mitochondria	trnaPHE + PHE + ATP -> PHEtrna + AMP + PPi	tRNA charging	SPCC736.03c	
Prolyl-tRNA synthetase	Cytosol	trnaPRO + PRO + ATP -> PROtrna + AMP + Ppi	tRNA charging	SPBC24C6.03 SPBC19C7.06	
Seryl-tRNA synthetase	Cytosol	trnaSER + SER + ATP-> SERtrna + AMP + PPi	tRNA charging	SPAC25B8.06c SPAC29A4.15	
Threonyl-tRNA synthetase	Cytosol	trnaTHR + THR + ATP -> THRtrna + AMP + PPi	tRNA charging	SPBC25H2.02	
threonyl-tRNA synthetase	Mitochondria	trnaTHR + THR + ATP -> THRtrna + AMP + PPi	tRNA charging	SPAC24C9.09	
Tryptophanyl-tRNA synthetase	Cytosol	trnaTRP + TRP + ATP -> TRPtrna + AMP + PPi	tRNA charging	SPAC2F7.13c	
Tryptophanyl-tRNA synthetase	Mitochondria	trnaTRP + TRP + ATP -> TRPtrna + AMP + PPi	tRNA charging	SPAC3G9.13c	
tyrosyl-tRNA synthetase	Cytosol	trnaTYR + TYR + ATP -> TYRtrna + AMP + PPi	tRNA charging	SPCC1672.05c	
tyrosyl-tRNA synthetase	Mitochondria	trnaTYR + TYR + ATP -> TYRtrna + AMP + PPi	tRNA charging	SPCC576.06c	
Valyl-tRNA synthetase	Cytosol	trnaVAL + VAL + ATP -> VALtrna + AMP + PPi	tRNA charging	SPAC4A8.08c SPBC1709.02C	
valyl-tRNA synthetase	Mitochondria	trnaVAL + VAL + ATP -> VALtrna + AMP + PPi	tRNA charging	SPAC4A8.08c SPBC1709.02C	
S-Adenosyl-L-methionine:catechol O-methyltransferase	Vacuole	SAM + DOP -> SAH + MOTAM	Tyrosine, Tryptophan, and Phenylalanine	SPBPB21E7.04c	2.1.1.6
4-(2-Aminoethyl)-1,2-benzenediol:o2 oxidoreductase(deaminating)(flavin-containing)	Cytosol	DOP + H2O + O2 -> DHPACAL + NH4 + H2O2	Tyrosine, Tryptophan, and Phenylalanine	SPAC2E1P3.04	1.4.3.21
3,4-Dihydroxyphenylacetaldehyde:nADP oxidoreductase	Cytosol	DHPACAL + NADP + H2O <-> DHPAC + NADPH + H	Tyrosine, Tryptophan, and Phenylalanine	SPAC922.07c	1.2.1.5
S-Adenosyl-L-methionine:catechol O-methyltransferase	Vacuole	SAM + DHPAC -> SAH + HVAN	Tyrosine, Tryptophan, and Phenylalanine	SPBPB21E7.04c	2.1.1.6
Tyramine:o2 oxidoreductase(deaminating)(flavin-containing)	Cytosol	TAM + H2O + O2 -> HPACAL + NH4 + H2O2	Tyrosine, Tryptophan, and Phenylalanine	SPAC2E1P3.04	1.4.3.21
4-Hydroxyphenylacetaldehyde:nADP oxidoreductase	Cytosol	HPACAL + NADP + H2O <-> HPAC + NADPH + H	Tyrosine, Tryptophan, and Phenylalanine	SPAC922.07c	1.2.1.5
3-Methoxy-4-hydroxyphenylacetaldehyde:nADP oxidoreductase	Cytosol	MOHPACAL + NADP + H2O -> HVAN + NADPH + H	Tyrosine, Tryptophan, and Phenylalanine	SPAC922.07c	1.2.1.5
3-Methoxy-4-hydroxyphenylglycolaldehyde:nADP oxidoreductase	Cytosol	MOHPGAL + NADP + H2O -> MOHMAN + NADPH + H	Tyrosine, Tryptophan, and Phenylalanine	SPAC922.07c	1.2.1.5
3,4-Dihydroxymandelaldehyde:nADP oxidoreductase	Cytosol	DHMANAL + NADP + H2O <-> DHMAN + NADPH + H	Tyrosine, Tryptophan, and Phenylalanine	SPAC922.07c	1.2.1.5
S-Adenosyl-L-methionine:catechol O-methyltransferase	Vacuole	DHMAN + SAM -> SAH + MOHMAN	Tyrosine, Tryptophan, and Phenylalanine	SPBPB21E7.04c	2.1.1.6
S-Adenosyl-L-methionine:catechol O-methyltransferase	Vacuole	NOADREN + SAM -> SAH + NOMNEP	Tyrosine, Tryptophan, and Phenylalanine	SPBPB21E7.04c	2.1.1.6
S-Adenosyl-L-methionine:catechol O-methyltransferase	Vacuole	ADREN + SAM -> SAH + MNEP	Tyrosine, Tryptophan, and Phenylalanine	SPBPB21E7.04c	2.1.1.6
S-Adenosyl-L-methionine:catechol O-methyltransferase	Vacuole	DHPEG + SAM -> SAH + MOHPEG	Tyrosine, Tryptophan, and Phenylalanine	SPBPB21E7.04c	2.1.1.6
Phenethylamine:oxygen oxidoreductase (deaminating)	Cytosol	PEA + O2 + H2O <-> PACALD + NH4 + H2O2	Tyrosine, Tryptophan, and Phenylalanine	SPAC2E1P3.04	1.4.3.21
aldehyde dehydrogenase (indole-3-acetaldehyde, NAD)	Cytosol	H2O + id3acald + NAD -> 2 H + ind3ac + NADH	Tyrosine, Tryptophan, and Phenylalanine	SPAC9E9.09c	1.2.1.3
aldehyde dehydrogenase (indole-3-acetaldehyde, NAD)	Golgi apparatus	H2O + id3acald + NAD -> 2 H + ind3ac + NADH	Tyrosine, Tryptophan, and Phenylalanine	SPAC9E9.09c	1.2.1.3
aldehyde dehydrogenase (indole-3-acetaldehyde, NADP)	Golgi apparatus	H2O + id3acald + NADP -> 2 H + ind3ac + NADPH	Tyrosine, Tryptophan, and Phenylalanine	SPAC9E9.09c	1.2.1.3
catalase A	Mitochondria	2 H2O2 -> 2 H2O + O2	Tyrosine, Tryptophan, and Phenylalanine	SPCC757.07c	1.11.1.6
N-Formyl-L-kynurenine amidohydrolase	Mitochondria	Lfmkynr + H2O -> Lkynr + FORM + H	Tyrosine, Tryptophan, and Phenylalanine	SPAC6B12.04c	2.6.1.-
catalase	Nucleus	2 H2O2 -> 2 H2O + O2	Tyrosine, Tryptophan, and Phenylalanine	SPCC757.07c	1.11.1.6
tyrosine transaminase (nucleus)	Nucleus	34Hpp + GLU -> AKG + TYR	Tyrosine, Tryptophan, and Phenylalanine	SPAC10F6.13c	2.6.1.1
tryptophan synthase (indoleglycerol phosphate)	Nucleus	3iG3P + SER -> G3P + H2O + TRP	Tyrosine, Tryptophan, and Phenylalanine	SPAC19A8.15	4.2.1.20
chorismate synthase	Nucleus	3psme -> CHOR + Pi	Tyrosine, Tryptophan, and Phenylalanine	SPCC1223.14	4.2.3.5
phenylalanine transaminase	Nucleus	AKG + PHE <-> GLU + pHPYR	Tyrosine, Tryptophan, and Phenylalanine	SPBC1773.13 SPAC56E4.03 SPCC569.07	2.6.1.57
tryptophan transaminase	Nucleus	AKG + TRP <-> GLU + indPYR	Tyrosine, Tryptophan, and Phenylalanine	SPBC1773.13 SPAC56E4.03 SPCC569.07	2.6.1.57
anthranilate phosphoribosyltransferase	Nucleus	antH + PRPP -> PPi + pran	Tyrosine, Tryptophan, and Phenylalanine	SPBC16G5.08	2.4.2.18
3-deoxy-D-arabino-heptulosonate 7-phosphate synthetase	Nucleus	E4P + H2O + PEP -> 2dda7p + Pi	Tyrosine, Tryptophan, and Phenylalanine	SPAC24H6.10c SPAP8A3.07c	2.5.1.54
aldehyde dehydrogenase (indole-3-acetaldehyde, NAD)	Nucleus	H2O + id3acald + NAD -> 2 H + ind3ac + NADH	Tyrosine, Tryptophan, and Phenylalanine	SPAC9E9.09c	1.2.1.3
aldehyde dehydrogenase (indole-3-acetaldehyde, NADP)	Nucleus	H2O + id3acald + NADP -> 2 H + ind3ac + NADPH	Tyrosine, Tryptophan, and Phenylalanine	SPAC9E9.09c	1.2.1.3
N-Formyl-L-kynurenine amidohydrolase	Nucleus	Lfmkynr + H2O -> Lkynr + FORM + H	Tyrosine, Tryptophan, and Phenylalanine	SPAC6B12.04c	2.6.1.-
4-Hydroxyphenylpyruvate:oxygen oxidoreductase	Cytosol	34Hpp + O2 -> CO2 + Hgentis	Tyrosine, Tryptophan, and Phenylalanine Metabolism		
aldehyde dehydrogenase (phenylacetaldehyde, NAD)	Cytosol	H2O + NAD + PACALD -> 2 H + NADH + pac	Tyrosine, Tryptophan, and Phenylalanine	SPAC922.07c	1.2.1.5

aldehyde dehydrogenase (indole-3-acetaldehyde, NAD)	Mitochondria	H2O + id3acald + NAD -> 2 H + ind3ac + NADH	Tyrosine, Tryptophan, and Phenylalanine	SPAC9E9.09c	1.2.1.3
aldehyde dehydrogenase (indole-3-acetaldehyde, NADP)	Cytosol	H2O + id3acald + NADP -> 2 H + ind3ac + NADPH	Tyrosine, Tryptophan, and Phenylalanine	SPAC9E9.09c	1.2.1.3
aldehyde dehydrogenase (indole-3-acetaldehyde, NADP)	Mitochondria	H2O + id3acald + NADP -> 2 H + ind3ac + NADPH	Tyrosine, Tryptophan, and Phenylalanine	SPAC9E9.09c	1.2.1.3
amidase	Cytosol	H2O + pad -> NH4 + pac	Tyrosine, Tryptophan, and Phenylalanine	SPBPB8B6.03	3.5.1.4
amidase	Cytosol	H2O + iad -> ind3ac + NH4	Tyrosine, Tryptophan, and Phenylalanine	SPBPB8B6.03	3.5.1.4
anthranilate phosphoribosyltransferase	Cytosol	antH + PRPP -> PPi + pran	Tyrosine, Tryptophan, and Phenylalanine	SPBC16G5.08	2.4.2.18
catalase	Cytosol	2 H2O2 -> 2 H2O + O2	Tyrosine, Tryptophan, and Phenylalanine	SPCC757.07c	1.11.1.6
catalase A	Peroxisome	2 H2O2 -> 2 H2O + O2	Tyrosine, Tryptophan, and Phenylalanine	SPCC757.07c	1.11.1.6
chorismate mutase	Cytosol	CHOR -> PPHN	Tyrosine, Tryptophan, and Phenylalanine	SPAC16E8.04c	2.6.1.-
chorismate mutase	Nucleus	CHOR -> PPHN	Tyrosine, Tryptophan, and Phenylalanine	SPAC16E8.04c	2.6.1.-
chorismate synthase	Cytosol	3psme -> CHOR + Pi	Tyrosine, Tryptophan, and Phenylalanine	SPCC1223.14	4.2.3.5
3-deoxy-D-arabino-heptulosonate 7-phosphate synthetase	Cytosol	E4P + H2O + PEP -> 2dda7p + Pi	Tyrosine, Tryptophan, and Phenylalanine	SPAC24H6.10c SPAP8A3.07c	2.5.1.54
2-deoxy-D-arabino-heptulosonate 7-phosphate synthetase	Mitochondria	E4P + H2O + PEP -> 2dda7p + Pi	Tyrosine, Tryptophan, and Phenylalanine	SPAC24H6.10c SPAP8A3.07c	2.5.1.54
3-dehydroquinate synthase	Cytosol	2dda7p -> 3dHQ + Pi	Tyrosine, Tryptophan, and Phenylalanine	SPAC1834.02	4.2.3.4
3-dehydroquinate dehydratase	Cytosol	3dHQ -> 3dHsk + H2O	Tyrosine, Tryptophan, and Phenylalanine	SPAC1834.02	4.2.1.10
diamine transaminase	Cytosol	ACCoA + sprm -> N1sprm + CoA + H	Tyrosine, Tryptophan, and Phenylalanine Metabolism		
N-Formyl-L-kynurenine amidohydrolase	Cytosol	Lfmkynr + H2O -> Lkynr + FORM + H	Tyrosine, Tryptophan, and Phenylalanine	SPAC6B12.04c	2.6.1.-
fumarylacetoacetase	Cytosol	4FUMacac + H2O -> acac + FUM + H	Tyrosine, Tryptophan, and Phenylalanine Metabolism		3.7.1.2
Homogentisate:oxygen 1,2-oxidoreductase (decyclizing)	Cytosol	Hgentis + O2 -> 4mlacac + H	Tyrosine, Tryptophan, and Phenylalanine Metabolism		
indole-3-glycerol-phosphate synthase	Cytosol	2cpR5P + H -> 3iG3P + CO2 + H2O	Tyrosine, Tryptophan, and Phenylalanine	SPBC1539.09c	4.1.1.48
maleylacetoacetate isomerase	Cytosol	4mlacac -> 4FUMacac	Tyrosine, Tryptophan, and Phenylalanine Metabolism		5.2.1.2
phenylalanine transaminase	Cytosol	AKG + PHE <-> GLU + pHPYR	Tyrosine, Tryptophan, and Phenylalanine	SPBC1773.13 SPAC56E4.03 SPCC569.07	2.6.1.57
prephenate dehydrogenase	Cytosol	NAD + PPHN -> 34Hpp + CO2 + NADH	Tyrosine, Tryptophan, and Phenylalanine	SPCC1494.04c	
prephenate dehydrogenase (NADP)	Cytosol	NADP + PPHN -> 34Hpp + CO2 + NADPH	Tyrosine, Tryptophan, and Phenylalanine	SPCC1494.04c	1.3.1.13
prephenate dehydratase	Cytosol	H + PPHN -> CO2 + H2O + pHPYR	Tyrosine, Tryptophan, and Phenylalanine	SPBC30D10.16	4.2.1.51
prephenate dehydratase	Nucleus	H + PPHN -> CO2 + H2O + pHPYR	Tyrosine, Tryptophan, and Phenylalanine	SPBC30D10.16	4.2.1.51
phosphoribosylanthranilate isomerase	Cytosol	pran -> 2cpR5P	Tyrosine, Tryptophan, and Phenylalanine	SPBC1539.09c	5.3.1.24
3-phosphoshikimate 1-carboxyvinyltransferase	Cytosol	PEP + skm5p -> 3psme + Pi	Tyrosine, Tryptophan, and Phenylalanine	SPAC1834.02	2.5.1.19
shikimate dehydrogenase	Cytosol	3dHsk + H + NADPH -> NADP + skm	Tyrosine, Tryptophan, and Phenylalanine	SPAC1834.02	1.1.1.25
shikimate kinase	Cytosol	ATP + skm -> ADP + H + skm5p	Tyrosine, Tryptophan, and Phenylalanine	SPAC1834.02	2.7.1.71
Spermidine acetyltransferase	Cytosol	ACCoA + spmd -> N1aspmd + CoA + H	Tyrosine, Tryptophan, and Phenylalanine Metabolism		
tryptophan synthase (indoleglycerol phosphate)	Cytosol	3iG3P + SER -> G3P + H2O + TRP	Tyrosine, Tryptophan, and Phenylalanine	SPAC19A8.15	4.2.1.20
tryptophan transaminase	Cytosol	AKG + TRP <-> GLU + indPYR	Tyrosine, Tryptophan, and Phenylalanine	SPBC1773.13 SPAC56E4.03 SPCC569.07	2.6.1.57
L-tyrosine N-formyltransferase	Cytosol	10FTHF + TYR -> Nfortyr + H + THF	Tyrosine, Tryptophan, and Phenylalanine Metabolism		
tyrosine transaminase	Cytosol	34Hpp + GLU -> AKG + TYR	Tyrosine, Tryptophan, and Phenylalanine	SPAC10F6.13c	2.6.1.1
tyrosine transaminase (mitochondrial)	Mitochondria	34Hpp + GLU -> AKG + TYR	Tyrosine, Tryptophan, and Phenylalanine	SPAC10F6.13c	2.6.1.1
2-oxo-4-methyl-3-carboxypentanoate decarboxylation	Nucleus	3c4MOP + H -> 4MOP + CO2	Valine, Leucine, and Isoleucine Metaboli	SPBC428.02c	2.6.1.42
isoleucine transaminase	Nucleus	AKG + ILE <-> 3MOP + GLU	Valine, Leucine, and Isoleucine Metaboli	SPBC428.02c	2.6.1.42
leucine transaminase	Nucleus	AKG + LEU <-> 4MOP + GLU	Valine, Leucine, and Isoleucine Metaboli	SPBC428.02c	2.6.1.42
valine transaminase	Nucleus	AKG + VAL <-> 3MOB + GLU	Valine, Leucine, and Isoleucine Metaboli	SPBC428.02c	2.6.1.42
2-aceto-2-hydroxybutanoate synthase	Cytosol	2obut + H + PYR -> 2aHbut + CO2	Valine, Leucine, and Isoleucine Metaboli	SPBP35G2.07 SPBC14C8.04	2.2.1.6
2-aceto-2-hydroxybutanoate synthase	Mitochondria	2obut + H + PYR -> 2aHbut + CO2	Valine, Leucine, and Isoleucine Metaboli	SPBP35G2.07	2.2.1.6
acetolactate synthase	Cytosol	H + 2 PYR -> alac-S + CO2	Valine, Leucine, and Isoleucine Metaboli	SPBP35G2.07 SPBC14C8.04	2.2.1.6
acetolactate synthase	Mitochondria	H + 2 PYR -> alac-S + CO2	Valine, Leucine, and Isoleucine Metaboli	SPBP35G2.07	2.2.1.6
dihydroxy-acid dehydratase (2,3-dihydroxy-3-methylbutanoate)	Mitochondria	23dHmb -> 3MOB + H2O	Valine, Leucine, and Isoleucine Metaboli	SPAC17G8.06c	4.2.1.9
dihydroxy-acid dehydratase (2,3-dihydroxy-3-methylpentanoate)	Mitochondria	23dHmp -> 3MOP + H2O	Valine, Leucine, and Isoleucine Metaboli	SPAC17G8.06c	4.2.1.9
isoleucine transaminase	Cytosol	AKG + ILE <-> 3MOP + GLU	Valine, Leucine, and Isoleucine Metaboli	SPBC428.02c	2.6.1.42
isoleucine transaminase	Mitochondria	AKG + ILE <-> 3MOP + GLU	Valine, Leucine, and Isoleucine Metaboli	SPBC428.02c	2.6.1.42
3-isopropylmalate dehydrogenase	Cytosol	3c2Hmp + NAD -> 3c4MOP + H + NADH	Valine, Leucine, and Isoleucine Metaboli	SPBC1A4.02c	1.1.1.85
3-isopropylmalate dehydratase	Cytosol	3c2Hmp <-> 2ippm + H2O	Valine, Leucine, and Isoleucine Metaboli	SPAC9E9.03	4.2.1.33
2-isopropylmalate hydratase	Cytosol	2ippm + H2O <-> 3c3Hmp	Valine, Leucine, and Isoleucine Metaboli	SPAC9E9.03	4.2.1.33
acetohydroxy acid isomeroreductase	Mitochondria	alac-S + H + NADPH -> 23dHmb + NADP	Valine, Leucine, and Isoleucine Metaboli	SPBC56F2.12	1.1.1.86
ketol-acid reductoisomerase (2-Aceto-2-hydroxybutanoate)	Mitochondria	2aHbut + H + NADPH -> 23dHmp + NADP	Valine, Leucine, and Isoleucine Metaboli	SPBC56F2.12	1.1.1.86
leucine transaminase	Cytosol	AKG + LEU <-> 4MOP + GLU	Valine, Leucine, and Isoleucine Metaboli	SPBC428.02c	2.6.1.42
leucine transaminase	Mitochondria	AKG + LEU <-> 4MOP + GLU	Valine, Leucine, and Isoleucine Metaboli	SPBC428.02c	2.6.1.42
2-Oxo-4-methyl-3-carboxypentanoate decarboxylation	Cytosol	3c4MOP + H -> 4MOP + CO2	Valine, Leucine, and Isoleucine Metaboli	SPBC428.02c	2.6.1.42
2-oxo-4-methyl-3-carboxypentanoate decarboxylation	Mitochondria	3c4MOP + H -> 4MOP + CO2	Valine, Leucine, and Isoleucine Metaboli	SPBC428.02c	2.6.1.42
valine transaminase	Cytosol	AKG + VAL <-> 3MOB + GLU	Valine, Leucine, and Isoleucine Metaboli	SPBC428.02c	2.6.1.42
valine transaminasediral	Mitochondria	AKG + VAL <-> 3MOB + GLU	Valine, Leucine, and Isoleucine Metaboli	SPBC428.02c	2.6.1.42
xylulokinase	Nucleus	ATP + xylu-D -> ADP + H + xu5p-D	Xylose Metabolism	SPCPJ732.02c	2.7.1.17
xylulokinase	Cytosol	ATP + xylu-D -> ADP + H + xu5p-D	Xylose Metabolism	SPCPJ732.02c	2.7.1.17
primary-amine oxidase	Cytosol	13dAMPp + O2 + H2O -> aproa + NH4 + H2O2		SPAC2E1P3.04	1.4.3.21
Hydrogen-sulfide:ferredoxin oxidoreductase	Cytosol	selt + 6 ferrR + 8 H -> seld + 6 ferrO + 3 H2O		SPAC10F6.01c	1.8.7.1
phosphatidylinositol-3,4,5-trisphosphate3-phosphatase	Cytosol	ptd345tp + H2O <-> ptd145bp + Pi		SPBC609.02	3.1.3.67
phosphatidylinositol-3,4,5-trisphosphate3-phosphatase	Mitochondria	ptd345tp + H2O <-> ptd145bp + Pi		SPBC609.02	3.1.3.67
	Cytosol	CHOR + GLN -> ADCHO + GLU			
	Cytosol	DHF + NAD -> FOL + NADH + H			
	Cytosol	FOL + 2 NADH + 2 H -> THF + 2 NAD			

Supporting Information 1B: List of metabolites used in SpoMBEL1693

ABBREVIATION	METABOLITE NAME	COMPARTMENT
13BDglcn	1,3-beta-D-Glucan	Extracellular
2hb	2-Hydroxybutyrate	Extracellular
2mbac	2-methylbutyl acetate	Extracellular
2mbald	2-methylbutyraldehyde	Extracellular
2mbtoh	2-methyl-1-butanol	Extracellular
2mppal	2-methylpropanal	Extracellular
2phetoh	2-phenylethanol	Extracellular
3c3hmp	3-Carboxy-3-hydroxy-4-methylpentanoate	Extracellular
3mbald	3-Methylbutanal	Extracellular
3mop	(S)-3-Methyl-2-oxopentanoate	Extracellular
4abut	4-Aminobutanoate	Extracellular
PABA	4-Aminobenzoate	Extracellular
5aop	5-Amino-4-oxopentanoate	Extracellular
8aonn	8-Amino-7-oxononanoate	Extracellular
abt	L-Arabinitol	Extracellular
ac	Acetate	Extracellular
ACAL	Acetaldehyde	Extracellular
aces	Acetic ester	Extracellular
ade	Adenine	Extracellular
adn	Adenosine	Extracellular
akg	2-Oxoglutarate	Extracellular
ALA	L-Alanine	Extracellular
alltn	Allantoin	Extracellular
alltt	Allantoate	Extracellular
SAM	S-Adenosyl-L-methionine	Extracellular
arab	D-Arabinose	Extracellular
arab	L-Arabinose	Extracellular
ARG	L-Arginine	Extracellular
ASN	L-Asparagine	Extracellular
ASP	L-Aspartate	Extracellular
btd-RR	(R,R)-2,3-Butanediol	Extracellular
btn	Biotin	Extracellular
chol	Choline	Extracellular
cit	Citrate	Extracellular
CO2	CO2	Extracellular
csn	Cytosine	Extracellular
CYS	L-Cysteine	Extracellular
cytd	Cytidine	Extracellular
dad-2	Deoxyadenosine	Extracellular
dann	7,8-Diaminononanoate	Extracellular
C100	Decanoate (n-C10:0)	Extracellular
dcyt	Deoxycytidine	Extracellular
C120	Dodecanoate (n-C12:0)	Extracellular
dgsn	Deoxyguanosine	Extracellular
din	Deoxyinosine	Extracellular
dttp	dTTP	Extracellular
duri	Deoxyuridine	Extracellular
epist	episterol	Extracellular
epistest	episterol ester	Extracellular
ergst	Ergosterol	Extracellular
ergstest	ergosterol ester	Extracellular
etha	Ethanolamine	Extracellular
etoh	Ethanol	Extracellular

fe2	Fe2+	Extracellular
fecost	fecosterol	Extracellular
fecostest	fecosterol ester	Extracellular
fmn	FMN	Extracellular
FORM	FORM ate	Extracellular
fru	D-Fructose	Extracellular
fum	Fumarate	Extracellular
g3pc	sn-Glycero-3-phosphocholine	Extracellular
g3pi	sn-Glycero-3-phospho-1-inositol	Extracellular
gal	D-Galactose	Extracellular
galur	D-Galacturonate	Extracellular
gam6p	D-Glucosamine 6-phosphate	Extracellular
gcald	Glycolaldehyde	Extracellular
GLC	D-Glucose	Extracellular
GLN	L-Glutamine	Extracellular
GLU	L-Glutamate	Extracellular
glx	Glyoxylate	Extracellular
GLY	Glycine	Extracellular
glyc	Glycerol	Extracellular
gsn	Guanosine	Extracellular
gthox	Oxidized glutathione	Extracellular
gthrd	Reduced glutathione	Extracellular
gua	Guanine	Extracellular
h	H+	Extracellular
H2O	H2O	Extracellular
hdca	Hexadecanoate (n-C16:0)	Extracellular
hdcea	Hexadecenoate (n-C16:1)	Extracellular
hexc	hexacosanoate (n-C26:0)	Extracellular
HIS	L-Histidine	Extracellular
hxan	Hypoxanthine	Extracellular
iamac	isoamyl acetate	Extracellular
iamoh	Isoamyl alcohol	Extracellular
ibutac	isobutyl acetate	Extracellular
ibutoh	isobutyl alcohol	Extracellular
id3acald	Indole-3-acetaldehyde	Extracellular
ILE	L-Isoleucine	Extracellular
ind3eth	Indole-3-ethanol	Extracellular
inost	myo-Inositol	Extracellular
ins	Inosine	Extracellular
k	potassium	Extracellular
dLAC	D-Lactate	Extracellular
LAC	L-Lactate	Extracellular
lanost	Lanosterol	Extracellular
lanostest	lanosterol ester	Extracellular
LEU	L-Leucine	Extracellular
LYS	L-Lysine	Extracellular
MAL	L-Malate	Extracellular
MALt	Maltose	Extracellular
man	D-Mannose	Extracellular
melib	Melibiose	Extracellular
MET	L-Methionine	Extracellular
mmet	S-Methyl-L-methionine	Extracellular
na1	Sodium	Extracellular
nac	Nicotinate	Extracellular
NADP	Nicotinamide adenine dinucleotide phosphate	Extracellular

Nbfortyr	N,N-bisFORM yl-dityrosine	Extracellular
nh4	Ammonium	Extracellular
nmn	NMN	Extracellular
o2	O2	Extracellular
oaa	Oxaloacetate	Extracellular
ocdca	octadecanoate (n-C18:0)	Extracellular
ocdcea	octadecenoate (n-C18:1)	Extracellular
ocdcya	octadecadienoate (n-C18:2)	Extracellular
orn	Ornithine	Extracellular
pacald	Phenylacetaldehyde	Extracellular
pap	Adenosine 3',5'-bisphosphate	Extracellular
pc	Phosphatidylcholine	Extracellular
pectin	Pectin	Extracellular
PEPd	PEPtide	Extracellular
pheac	Phenethyl acetate	Extracellular
PHE	L-Phenylalanine	Extracellular
Pi	Phosphate	Extracellular
pnto-R	(R)-Pantothenate	Extracellular
PRO	L-Proline	Extracellular
ptd1ino	phosphatidyl-1D-myo-inositol	Extracellular
ptrc	Putrescine	Extracellular
PYR	Pyruvate	Extracellular
rib	D-Ribose	Extracellular
ribflv	Riboflavin	Extracellular
sbt	Dorbitol	Extracellular
sbt	Lorbitol	Extracellular
SER	Lerine	Extracellular
SO3	Sulfite	Extracellular
SO4	Sulfate	Extracellular
spmd	Spermidine	Extracellular
sprm	Spermine	Extracellular
srb	Lorbose	Extracellular
succ	Succinate	Extracellular
sucr	Sucrose	Extracellular
taur	Taurine	Extracellular
thm	Thiamin	Extracellular
thmmp	Thiamin monophosphate	Extracellular
thmpp	Thiamine diphosphate	Extracellular
THR	L-Threonine	Extracellular
thym	Thymine	Extracellular
thymd	Thymidine	Extracellular
tre	Trehalose	Extracellular
TRP	L-Tryptophan	Extracellular
ttdca	tetradecanoate (n-C14:0)	Extracellular
TYR	L-Tyrosine	Extracellular
ura	Uracil	Extracellular
urea	Urea	Extracellular
uri	Uridine	Extracellular
VAL	L-Valine	Extracellular
xan	Xanthine	Extracellular
xtsn	Xanthosine	Extracellular
xyl	D-Xylose	Extracellular
xylt	Xylitol	Extracellular
zymst	zymosterol	Extracellular
zymstest	zymosterol ester	Extracellular

GALI	Galactinol, 1-alpha-D-Galactosyl-myo-inositol	Extracellular
10fthf	10-FORM yltetrahydrofolate	Cytosol
10fthf	10-FORM yltetrahydrofolate	Mitochondria
12dgr	1,2-Diacylglycerol	Cytosol
13BDglcn	1,3-beta-D-Glucan	Cytosol
13dampp	1,3-Diaminopropane	Cytosol
13dpg	3-Phospho-D-glyceroyl phosphate	Cytosol
14glun	(1,4-alpha-D-Glucosyl)n	Cytosol
16BDglcn	1,6-beta-D-Glucan	Cytosol
1ag3p	1-Acyln-glycerol 3-phosphate	Cytosol
1agly3p	1-Acyl-glycerone 3-phosphate	Cytosol
1agpc	1-Acyln-glycerol-3-phosphocholine	Cytosol
GALI	Galactinol, 1-alpha-D-Galactosyl-myo-inositol	Endoplasmic Reticulum
1mncam	1-Methylnicotinamide	Cytosol
1p3h5c	L-1-Pyrroline-3-hydroxy-5-carboxylate	Cytosol
1p3h5c	L-1-Pyrroline-3-hydroxy-5-carboxylate	Mitochondria
1pyr5c	1-Pyrroline-5-carboxylate	Cytosol
1pyr5c	1-Pyrroline-5-carboxylate	Mitochondria
23camp	2',3'-Cyclic AMP	Cytosol
23dhmb	(R)-2,3-Dihydroxy-3-methylbutanoate	Mitochondria
23dhmp	(R)-2,3-Dihydroxy-3-methylpentanoate	Mitochondria
23dpg	2,3-Disphospho-D-glycerate	Cytosol
saicar	(S)-2-[5-Amino-1-(5-phospho-D-ribosyl)imidazole-4-carboxamido]succinate	Cytosol
25dhpp	2,5-Diamino-6-hydroxy-4-(5'-phosphoribosylamino)-pyrimidine	Cytosol
25dthpp	2,5-diamino-6-ribitylamino-4(3H)-pyrimidinone 5'-phosphate	Cytosol
2ahbut	(S)-2-Aceto-2-hydroxybutanoate	Mitochondria
2ahhmd	2-Amino-4-hydroxy-6-hydroxymethyl-7,8-dihydropteridine diphosphate	Mitochondria
2ahhmp	2-Amino-4-hydroxy-6-hydroxymethyl-7,8-dihydropteridine	Mitochondria
2amsa	2-Aminomalonate semialdehyde	Cytosol
2aobut	L-2-Amino-3-oxobutanoate	Cytosol
2cpr5p	1-(2-Carboxyphenylamino)-1-deoxy-D-ribulose 5-phosphate	Cytosol
2dda7p	2-Dehydro-3-deoxy-D-arabino-heptonate 7-phosphate	Cytosol
2dda7p	2-Dehydro-3-deoxy-D-arabino-heptonate 7-phosphate	Mitochondria
2dglc	2-Deoxy-D-glucose	Cytosol
2dhp	2-Dehydropantoate	Cytosol
2dhp	2-Dehydropantoate	Mitochondria
2doxG6P	2-Deoxy-D-glucose 6-phosphate	Cytosol
2dr1p	2-Deoxy-D-ribose 1-phosphate	Cytosol
2dr5p	2-Deoxy-D-ribose 5-phosphate	Cytosol
2hb	2-Hydroxybutyrate	Cytosol
2hhxdal	2-Hydroxy-hexadecanal	Cytosol
2OPMB	2-Hexaprenyl-6-methoxy-1,4-benzoquinone	Mitochondria
2OPMP	2-Hexaprenyl-6-methoxyphenol	Mitochondria
2OMHMB	2-hexaprenyl-3-methyl-5-hydroxy-6-methoxy-1,4-benzoquinone	Mitochondria
2OPMMB	2-hexaprenyl-3-methyl-6-methoxy-1,4-benzoquinone	Mitochondria
2ippm	2-Isopropylmaleate	Cytosol
2kmb	2-keto-4-methylthiobutyrate	Cytosol
2mahmp	2-Methyl-4-amino-5-hydroxymethylpyrimidine diphosphate	Cytosol
2mbac	2-methylbutyl acetate	Cytosol
2mbald	2-methylbutyraldehyde	Cytosol
2mbald	2-methylbutyraldehyde	Mitochondria
2mbtoh	2-methyl-1-butanol	Cytosol
2mbtoh	2-methyl-1-butanol	Mitochondria
2mcit	2-Methylcitrate	Mitochondria
2mppal	2-methylpropanal	Cytosol

2mppal	2-methylpropanal	Mitochondria
2obut	2-Oxobutanoate	Cytosol
2obut	2-Oxobutanoate	Mitochondria
2oxoadp	2-Oxoadipate	Cytosol
2oxoadp	2-Oxoadipate	Mitochondria
2pg	D-Glycerate 2-phosphate	Cytosol
2phetoh	2-phenylethanol	Cytosol
2phetoh	2-phenylethanol	Mitochondria
34hpl	3-(4-Hydroxyphenyl)lactate	Mitochondria
34hpp	3-(4-Hydroxyphenyl)pyruvate	Cytosol
34hpp	3-(4-Hydroxyphenyl)pyruvate	Mitochondria
34hpp	3-(4-Hydroxyphenyl)pyruvate	Peroxisome
35cCMP	3',5'-Cyclic CMP	Cytosol
35cdamp	3',5'-Cyclic dAMP	Cytosol
35cGMP	3',5'-Cyclic GMP	Cytosol
35cIMP	3',5'-Cyclic IMP	Cytosol
3c2hmp	3-Carboxy-2-hydroxy-4-methylpentanoate	Cytosol
3c3hmp	3-Carboxy-3-hydroxy-4-methylpentanoate	Cytosol
3c3hmp	3-Carboxy-3-hydroxy-4-methylpentanoate	Mitochondria
3c4mop	3-Carboxy-4-methyl-2-oxopentanoate	Cytosol
3c4mop	3-Carboxy-4-methyl-2-oxopentanoate	Mitochondria
3H45DHBZ	3-Hexaprenyl-4,5-dihydroxybenzoate	Cytosol
3H45DHBZ	3-Hexaprenyl-4,5-dihydroxybenzoate	Mitochondria
3dhq	3-Dehydroquinate	Cytosol
3dhsk	3-Dehydroshikimate	Cytosol
3dsphgn	3-Dehydrosphinganine	Cytosol
3hanthrn	3-Hydroxyanthranilate	Cytosol
3hdCoA	(S)-3-Hydroxydecanoyl-CoA	Peroxisome
3hddCoA	(S)-3-Hydroxydodecanoyl-CoA	Peroxisome
3hhdCoA	(S)-3-Hydroxyhexadecanoyl-CoA	Peroxisome
3hodCoA	(S)-3-Hydroxyoctadecanoyl-CoA	Peroxisome
3H4H5MOBZ	3-Hexaprenyl-4-hydroxy-5-methoxybenzoate	Mitochondria
3htdCoA	(S)-3-Hydroxytetradecanoyl-CoA	Peroxisome
3hxcCoA	(S)-3-Hydroxyhexacosyl-CoA	Peroxisome
3ig3p	C'-(3-Indolyl)-glycerol 3-phosphate	Cytosol
3ipmmest	3-isopropylmalate-methyl-ester	Cytosol
3mbald	3-Methylbutanal	Cytosol
3mbald	3-Methylbutanal	Mitochondria
3mob	3-Methyl-2-oxobutanoate	Cytosol
3mob	3-Methyl-2-oxobutanoate	Mitochondria
3mop	(S)-3-Methyl-2-oxopentanoate	Cytosol
3mop	(S)-3-Methyl-2-oxopentanoate	Mitochondria
3odCoA	3-Oxodecanoyl-CoA	Peroxisome
3oddCoA	3-Oxododecanoyl-CoA	Peroxisome
3ohdCoA	3-Oxohexadecanoyl-CoA	Peroxisome
3ohodCoA	3-Oxooctadecanoyl-CoA	Peroxisome
3ohxcCoA	3-Oxohexacosyl-CoA	Peroxisome
O4HBZ	3-Hexaprenyl-4-hydroxybenzoate	Cytosol
O4HBZ	3-Hexaprenyl-4-hydroxybenzoate	Mitochondria
3otdCoA	3-Oxotetradecanoyl-CoA	Peroxisome
3pg	3-Phospho-D-glycerate	Cytosol
3php	3-Phosphohydroxypyruvate	Cytosol
3psme	5-O-(1-Carboxyvinyl)-3-phosphoshikimate	Cytosol
44mctr	4,4-dimethylcholesta-8,14,24-trienol	Cytosol
44mzym	4,4-dimethylzymosterol	Cytosol

4aabutn	4-Acetamidobutanoate	Cytosol
4abut	4-Aminobutanoate	Cytosol
4abut	4-Aminobutanoate	Mitochondria
4abutn	4-Aminobutanal	Cytosol
4abutn	4-Aminobutanal	Mitochondria
PABA	4-Aminobenzoate	Cytosol
PABA	4-Aminobenzoate	Mitochondria
adcho	4-amino-4-deoxychorismate	Cytosol
4ahmmp	4-Amino-5-hydroxymethyl-2-methylpyrimidine	Cytosol
4ampm	4-Amino-2-methyl-5-phosphomethylpyrimidine	Cytosol
4fumacac	4-Fumarylacetoacetate	Cytosol
4gudbd	4-Guanidinobutanamide	Cytosol
4gudbutn	4-Guanidinobutanoate	Cytosol
4H2Oglt	4-Hydroxy-2-oxoglutarate	Cytosol
4H2Oglt	4-Hydroxy-2-oxoglutarate	Mitochondria
4H2Oglt	4-Hydroxy-2-oxoglutarate	Peroxisome
4hbz	4-Hydroxybenzoate	Cytosol
4hbz	4-Hydroxybenzoate	Mitochondria
4hbzCoA	4-hydroxybenoyl-CoA	Mitochondria
4hglusa	L-4-Hydroxyglutamate semialdehyde	Mitochondria
4hproT	trans-4-Hydroxy-L-proline	Cytosol
4hproT	trans-4-Hydroxy-L-proline	Mitochondria
4hthr	4-Hydroxy-L-threonine	Cytosol
4mhetz	4-Methyl-5-(2-hydroxyethyl)-thiazole	Cytosol
4mlacac	4-Maleylacetoacetate	Cytosol
4mop	4-Methyl-2-oxopentanoate	Cytosol
4mop	4-Methyl-2-oxopentanoate	Mitochondria
4mpetz	4-Methyl-5-(2-phosphoethyl)-thiazole	Cytosol
4mzym	4-methylzymosterol	Cytosol
44mzymc	4alpha-Methylzymosterol-4-carboxylate	Cytosol
3k4mzym	4-Methylzymosterol intermediate 2	Cytosol
4pasp	4-Phospho-L-aspartate	Cytosol
4ppan	D-4'-Phosphopantothenate	Cytosol
4ppcys	N-((R)-4-Phosphopantothenoyl)-L-cysteine	Cytosol
4r5au	4-(1-D-Ribitylamino)-5-aminouracil	Cytosol
cair	5-amino-1-(5-phospho-D-ribosyl)imidazole-4-carboxylate	Cytosol
5aop	5-Amino-4-oxopentanoate	Cytosol
5aop	5-Amino-4-oxopentanoate	Mitochondria
5aprbu	5-Amino-6-(5'-phosphoribitylamino)uracil	Cytosol
5dpmev	(R)-5-Diphosphomevalonate	Cytosol
5fthf	5-FORM yltetrahydrofolate	Cytosol
5fthf	5-FORM yltetrahydrofolate	Mitochondria
5mdr1p	5-Methylthio-5-deoxy-D-ribose 1-phosphate	Cytosol
5mdru1p	5-Methylthio-5-deoxy-D-ribulose 1-phosphate	Cytosol
5mta	5-Methylthioadenosine	Cytosol
5mthf	5-Methyltetrahydrofolate	Cytosol
5pmev	(R)-5-Phosphomevalonate	Cytosol
6dg	D-Gal-alpha-1->6D-Glucose	Cytosol
6pgc	6-Phospho-D-gluconate	Cytosol
6pgl	6-phospho-D-glucono-1,5-lactone	Cytosol
6pgl	6-phospho-D-glucono-1,5-lactone	Endoplasmic Reticulum
8aonn	8-Amino-7-oxononanoate	Cytosol
aacald	Aminoacetaldehyde	Cytosol
aaCoA	Acetoacetyl-CoA	Cytosol
aaCoA	Acetoacetyl-CoA	Mitochondria

aact	Aminoacetone	Cytosol
abt	L-Arabinitol	Cytosol
ac	Acetate	Cytosol
ac	Acetate	Mitochondria
ac	Acetate	Peroxisome
acac	Acetoacetate	Cytosol
acACP	Acetyl-ACP	Cytosol
acACP	Acetyl-ACP	Mitochondria
ACAL	Acetaldehyde	Cytosol
ACAL	Acetaldehyde	Mitochondria
acCoA	Acetyl-CoA	Cytosol
acCoA	Acetyl-CoA	Mitochondria
acCoA	Acetyl-CoA	Nucleus
acCoA	Acetyl-CoA	Peroxisome
aces	Acetic ester	Cytosol
acg5p	N-Acetyl-L-glutamyl 5-phosphate	Mitochondria
acg5sa	N-Acetyl-L-glutamate 5emialdehyde	Mitochondria
acgam1p	N-Acetyl-D-glucosamine 1-phosphate	Cytosol
acgam6p	N-Acetyl-D-glucosamine 6-phosphate	Cytosol
acglu	N-Acetyl-L-glutamate	Mitochondria
achms	O-Acetyl-L-homoserine	Cytosol
acon5m	E-3-carboxyl-2-pentenedioate 5-methyl ester	Cytosol
acon-T	trans-Aconitate	Cytosol
acorn	N2-Acetyl-L-ornithine	Mitochondria
ACP	acyl carrier protein	Cytosol
ACP	acyl carrier protein	Mitochondria
ACP1C	1-Aminocyclopropane-1-carboxylate	Cytosol
acser	O-Acetyl-Lerine	Cytosol
actn-R	(R)-Acetoin	Cytosol
acybut	gamma-Amino-gamma-cyanobutanoate	Cytosol
ade	Adenine	Cytosol
ade	Adenine	Mitochondria
adn	Adenosine	Cytosol
adn	Adenosine	Mitochondria
ADP	ADP	Cytosol
ADP	ADP	Golgi Apparatus
ADP	ADP	Mitochondria
ADP	ADP	Nucleus
ADP	ADP	Vacuole
ADP	ADP	Peroxisome
adprib	ADPribose	Cytosol
adprib	ADPribose	Mitochondria
adren	L-Adrenaline	???
ahcys	S-Adenosyl-L-homocysteine	Cytosol
ahcys	S-Adenosyl-L-homocysteine	Mitochondria
ahdt	2-Amino-4-hydroxy-6-(erythro-1,2,3-trihydroxypropyl)dihydropteridine triphosphate	Cytosol
aicar	5-Amino-1-(5-Phospho-D-ribosyl)imidazole-4-carboxamide	Cytosol
air	5-amino-1-(5-phospho-D-ribosyl)imidazole	Cytosol
akg	2-Oxoglutarate	Cytosol
akg	2-Oxoglutarate	Mitochondria
akg	2-Oxoglutarate	Nucleus
akg	2-Oxoglutarate	Peroxisome
ala-B	beta-Alanine	Cytosol
ala	D-Alanine	Cytosol

alac	(S)-2-Acetolactate	Mitochondria
ALA	L-Alanine	Cytosol
ALA	L-Alanine	Mitochondria
alatrna	L-Alanyl-tRNA(Ala)	Cytosol
allphn	Allophanate	Cytosol
alltn	Allantoin	Cytosol
alltt	Allantoate	Cytosol
alpam	S-aminomethyldihydrolipoamide	Mitochondria
alpro	S-Aminomethyldihydrolipoylprotein	Mitochondria
SAM	S-Adenosyl-L-methionine	Cytosol
SAM	S-Adenosyl-L-methionine	Mitochondria
ametam	S-Adenosylmethioninamine	Cytosol
amob	S-Adenosyl-4-methylthio-2-oxobutanoate	Cytosol
AMP	AMP	Cytosol
AMP	AMP	Mitochondria
AMP	AMP	Nucleus
AMP	AMP	Peroxisome
amp2p	Adenosine 2'-phosphate	Cytosol
anth	Anthranilate	Cytosol
ap4a	P1,P4-Bis(5'-adenosyl) tetraphosphate	Cytosol
ap4g	P1-(5'-adenosyl),P4-(5'-guanosyl) tetraphosphate	Cytosol
aPEP	Nalpha-AcetylPEPtide	Cytosol
aproa	3-Aminopropanal	Cytosol
aprop	alpha-Aminopropiononitrile	Cytosol
aprut	N-Acetylputrescine	Cytosol
aps	Adenosine 5'-phosphosulfate	Cytosol
arab	D-Arabinose	Cytosol
arab	L-Arabinose	Cytosol
ARG	L-Arginine	Cytosol
ARG	L-Arginine	Mitochondria
ARG	L-Arginine	Vacuole
argsuc	N(omega)-(L-Arginino)succinate	Cytosol
argtrna	L-Arginyl-tRNA(Arg)	Cytosol
argtrna	L-Arginyl-tRNA(Arg)	Mitochondria
ASN	L-Asparagine	Cytosol
ASN	L-Asparagine	Mitochondria
ASN	L-Asparagine	Vacuole
asntrna	L-Asparaginyl-tRNA(Asn)	Cytosol
asntrna	L-Asparaginyl-tRNA(Asn)	Mitochondria
ASP	L-Aspartate	Cytosol
ASP	L-Aspartate	Mitochondria
ASP	L-Aspartate	Nucleus
ASP	L-Aspartate	Vacuole
ASP	L-Aspartate	Peroxisome
aspsa	L-Aspartate 4emialdehyde	Cytosol
asptrna	L-Aspartyl-tRNA(Asp)	Cytosol
asptrna	L-Aspartyl-tRNA(Asp)	Mitochondria
athr	L-Allo-threonine	Cytosol
ATP	ATP	Cytosol
ATP	ATP	Golgi Apparatus
ATP	ATP	Mitochondria
ATP	ATP	Nucleus
ATP	ATP	Vacuole
ATP	ATP	Peroxisome
b124tc	But-1-ene-1,2,4-tricarboxylate	Mitochondria

btamp	Biotinyl-5'-AMP	Cytosol
btd-RR	(R,R)-2,3-Butanediol	Cytosol
btn	Biotin	Cytosol
camp	cAMP	Cytosol
caphis	2-(3-Carboxy-3-aminopropyl)-L-histidine	Cytosol
cbasp	N-Carbamoyl-L-aspartate	Cytosol
cbasp	N-Carbamoyl-L-aspartate	Nucleus
cbp	Carbamoyl phosphate	Cytosol
cbp	Carbamoyl phosphate	Nucleus
CDP	CDP	Cytosol
CDP	CDP	Nucleus
CDPchol	CDPcholine	Cytosol
CDPdag	CDPdiacylglycerol	Cytosol
CDPdag	CDPdiacylglycerol	Mitochondria
CDPea	CDPethanolamine	Cytosol
cer124	Ceramide-1 (Sphinganine:n-C24:0)	Cytosol, ER, Golgi apparatus, Mitochondria
cer126	Ceramide-1 (Sphinganine:n-C26:0)	Cytosol, ER, Golgi apparatus, Mitochondria
cer224	Ceramide-2 (Phytosphingosine:n-C24:0)	Cytosol
cer2'_24	Ceramide-2' (Sphinganine:n-C24:0OH)	Cytosol
cer224	Ceramide-2 (Phytosphingosine:n-C24:0)	Endoplasmic Reticulum
cer226	Ceramide-2 (Phytosphingosine:n-C26:0)	Cytosol
cer2'_26	Ceramide-2' (Sphinganine:n-C26:0OH)	Cytosol
cer226	Ceramide-2 (Phytosphingosine:n-C26:0)	Endoplasmic Reticulum
cer324	Ceramide-3 (Phytosphingosine:n-C24:0OH)	Cytosol
cer326	Ceramide-3 (Phytosphingosine:n-C26:0OH)	Cytosol
cgly	Cys-Gly	Cytosol
ch4s	Methanethiol	Cytosol
chitin	Chitin (monomer)	Cytosol
chitos	Chitosan	Cytosol
chol	Choline	Cytosol
cholp	Choline phosphate	Cytosol
CHOR	chorismate	Cytosol
cit	Citrate	Cytosol
cit	Citrate	Mitochondria
cit	Citrate	Peroxisome
citr	L-Citrulline	Cytosol
clpn	Cardiolipin	Mitochondria
cmaphis	2-[3-Carboxy-3-(methylammonio)propyl]-L-histidine	Cytosol
CMP	CMP	Cytosol
CMP	CMP	Mitochondria
cmusa	2-Amino-3-carboxymuconate semialdehyde	Cytosol
CO2	CO2	Cytosol
CO2	CO2	Golgi Apparatus
CO2	CO2	Mitochondria
CO2	CO2	Nucleus
CO2	CO2	Vacuole
CO2	CO2	Peroxisome
CoA	Coenzyme A	Cytosol
CoA	Coenzyme A	Mitochondria
CoA	Coenzyme A	Nucleus
CoA	Coenzyme A	Endoplasmic Reticulum
CoA	Coenzyme A	Peroxisome
couCoA	p-coumaroyl-CoA	Mitochondria
cpppg3	Coproporphyrinogen III	Cytosol

csn	Cytosine	Cytosol
CTP	CTP	Cytosol
CTP	CTP	Mitochondria
CYS	L-Cysteine	Cytosol
cyst	L-Cystathionine	Cytosol
cyst	L-Cystathionine	Peroxisome
cystrna	L-Cysteinyl-tRNA(Cys)	Cytosol
cytd	Cytidine	Cytosol
dad-2	Deoxyadenosine	Cytosol
dadp	dADP	Cytosol
dadp	dADP	Nucleus
dagpy	diacylglycerol pyrophosphate	Cytosol
damp	dAMP	Cytosol
dann	7,8-Diaminononanoate	Cytosol
Dara14lac	D-Arabinono-1,4-lactone	Cytosol
datp	dATP	Cytosol
db4p	3,4-dihydroxy-2-butanone 4-phosphate	Cytosol
dc2CoA	trans-Dec-2-enoyl-CoA	Peroxisome
C100	Decanoate (n-C10:0)	Cytosol
C100	Decanoate (n-C10:0)	Peroxisome
C100ACP	Decanoyl-ACP (n-C10:0ACP)	Mitochondria
C100CoA	Decanoyl-CoA (n-C10:0CoA)	Cytosol
C100CoA	Decanoyl-CoA (n-C10:0CoA)	Peroxisome
dcamp	N6-(1,2-Dicarboxyethyl)-AMP	Cytosol
dCDP	dCDP	Cytosol
dCDP	dCDP	Nucleus
dCMP	dCMP	Cytosol
dCTP	dCTP	Cytosol
dcyt	Deoxycytidine	Cytosol
dd2CoA	trans-Dodec-2-enoyl-CoA	Peroxisome
C120	Dodecanoate (n-C12:0)	Cytosol
C120	Dodecanoate (n-C12:0)	Peroxisome
C120ACP	Dodecanoyl-ACP (n-C12:0ACP)	Cytosol
C120ACP	Dodecanoyl-ACP (n-C12:0ACP)	Mitochondria
C120CoA	Dodecanoyl-CoA (n-C12:0CoA)	Cytosol
C120CoA	Dodecanoyl-CoA (n-C12:0CoA)	Peroxisome
dext	Dextrin	Cytosol
dGDP	dGDP	Cytosol
dGDP	dGDP	Nucleus
dGMP	dGMP	Cytosol
dgsn	Deoxyguanosine	Cytosol
dGTP	dGTP	Cytosol
dha	Dihydroxyacetone	Cytosol
dhap	Dihydroxyacetone phosphate	Cytosol
dhap	Dihydroxyacetone phosphate	Mitochondria
dhf	7,8-Dihydrofolate	Cytosol
dhf	7,8-Dihydrofolate	Mitochondria
dhlam	Dihydrolipoamide	Mitochondria
dhlpro	Dihydrolipolprotein	Mitochondria
dhman	3,4-Dihydroxymandelate	???
dhmanal	3,4-Dihydroxymandelaldehyde	???
dhnpt	Dihydroneopterin	Cytosol
dhnpt	Dihydroneopterin	Mitochondria
dhor	(S)-Dihydroorotate	Cytosol
dhpac	3,4-Dihydroxyphenylacetate	???

dhpacal	3,4-Dihydroxyphenylacetaldehyde	???
dhpeg	3,4-Dihydroxyphenylethyleneglycol	???
dhpmp	Dihydroneopterin monophosphate	Cytosol
dhpt	Dihydropteroate	Cytosol
dhpt	Dihydropteroate	Mitochondria
din	Deoxyinosine	Cytosol
dkmpp	2,3-diketo-5-methylthio-1-phosphopentane	Cytosol
dmlz	6,7-Dimethyl-8-(1-D-ribityl)lumazine	Cytosol
DMPP	Dimethylallyl diphosphate	Cytosol
dNAD	Deamino-NAD+	Cytosol
dNAD	Deamino-NAD+	Mitochondria
dNAD	Deamino-NAD+	Nucleus
dolichol	Dolichol	Cytosol
dolmanp	Dolichyl phosphate D-mannose	Endoplasmic Reticulum
dolp	Dolichol phosphate	Cytosol
dolp	Dolichol phosphate	Endoplasmic Reticulum
dop	Dopamine	???
dpCoA	Dephospho-CoA	Cytosol
dpCoA	Dephospho-CoA	Mitochondria
drib	Deoxyribose	Cytosol
dscl	dihydrosirohydrochlorin	Cytosol
dtbt	Dethiobiotin	Cytosol
dtdp	dTDP	Cytosol
dtmp	dTMP	Cytosol
dttp	dTTP	Cytosol
dudp	dUDP	Cytosol
dudp	dUDP	Nucleus
dump	dUMP	Cytosol
dump	dUMP	Nucleus
duri	Deoxyuridine	Cytosol
dutp	dUTP	Cytosol
e3s	Estrone 3ulfate	Cytosol
e4hglu	L-erythro-4-Hydroxyglutamate	Cytosol
e4hglu	L-erythro-4-Hydroxyglutamate	Mitochondria
e4hglu	L-erythro-4-Hydroxyglutamate	Peroxisome
e4p	D-Erythrose 4-phosphate	Cytosol
e4p	D-Erythrose 4-phosphate	Mitochondria
eig3p	D-erythro-1-(Imidazol-4-yl)glycerol 3-phosphate	Cytosol
emp	Epimelibiose	Extracellular
epist	episterol	Cytosol
epistest	episterol ester	Cytosol
epm	Epimelibiose	Cytosol
ergst	Ergosterol	Cytosol
ergst	Ergosterol	Endoplasmic Reticulum
ergst3glc	ergosterol 3-beta-D-glucoside	Cytosol
ergstest	ergosterol ester	Cytosol
ergtetrol	Ergosta-5,7,22,24,(28)-tetraen-3beta-ol	Cytosol
ergtetrol	Ergosta-5,7,22,24,(28)-tetraen-3beta-ol	Endoplasmic Reticulum
ergtrol	ergosta-5,7,24(28)-trienol	Cytosol
ertascb	D-erythro-Ascorbate	Cytosol
est	Estrone	Cytosol
etha	Ethanolamine	Cytosol
ethamp	Ethanolamine phosphate	Cytosol
etoh	Ethanol	Cytosol
etoh	Ethanol	Mitochondria

f1p	D-Fructose 1-phosphate	Cytosol
f26bp	D-Fructose 2,6-bisphosphate	Cytosol
f6p	D-Fructose 6-phosphate	Cytosol
FAD	Flavin adenine dinucleotide oxidized	Cytosol
FAD	Flavin adenine dinucleotide oxidized	Mitochondria
FADH2	Flavin adenine dinucleotide reduced	Mitochondria
fald	FORM aldehyde	Cytosol
fdp	D-Fructose 1,6-bisphosphate	Cytosol
fe2	Fe2+	Cytosol
fe2	Fe2+	Mitochondria
fecost	fecosterol	Cytosol
fecostest	fecosterol ester	Cytosol
ferrO	Ferredoxin - Oxidized	
ferrR	Ferredoxin - Reduced	
fgar	N2-FORM yl-N1-(5-phospho-D-ribosyl)glycinamide	Cytosol
ficytc	Ferricytochrome c	Mitochondria
fmettrna	N-FORM ylmethionyl-tRNA	Mitochondria
fmn	FMN	Cytosol
fmn	FMN	Mitochondria
fmnh2	Reduced FMN	Cytosol
focytc	Ferrocytochrome c	Mitochondria
fol	Folate	Cytosol
FORM	Formate	Cytosol
FORM	Formate	Mitochondria
fgam	2-(FORM amido)-N1-(5-phospho-D-ribosyl)acetamidine	Cytosol
fprica	5-FORM amido-1-(5-phospho-D-ribosyl)imidazole-4-carboxamide	Cytosol
FRPP	Farnesyl diphosphate	Cytosol
FRPP	Farnesyl diphosphate	Mitochondria
fru	D-Fructose	Cytosol
fum	Fumarate	Cytosol
fum	Fumarate	Mitochondria
g1p	D-Glucose 1-phosphate	Cytosol
g3p	Glyceraldehyde 3-phosphate	Cytosol
g3p	sn-Glycero-3-phosphocholine	Cytosol
g3pi	sn-Glycero-3-phospho-1-inositol	Cytosol
G6P	D-Glucose 6-phosphate	Cytosol
G6P	D-Glucose 6-phosphate	Endoplasmic Reticulum
G6P-B	beta-D-glucose 6-phosphate	Cytosol
gal	D-Galactose	Cytosol
gal1p	alpha-D-Galactose 1-phosphate	Cytosol
gala	Galactosylceramide	Cytosol
galcman	Galactomannan	Cytosol
gam1p	D-Glucosamine 1-phosphate	Cytosol
gam6p	D-Glucosamine 6-phosphate	Cytosol
gar	N1-(5-Phospho-D-ribosyl)glycinamide	Cytosol
gcald	Glycolaldehyde	Cytosol
gcald	Glycolaldehyde	Mitochondria
GDP	GDP	Cytosol
GDP	GDP	Golgi Apparatus
GDP	GDP	Mitochondria
GDP	GDP	Nucleus
GDPmann	GDP-D-mannose	Cytosol
GDPmann	GDP-D-mannose	Golgi Apparatus
GGPP	Geranylgeranyl diphosphate	Cytosol
ggl	Galactosylglycerol	Extracellular

GLC	D-Glucose	Cytosol, ER, Golgi apparatus, Vacuole
GLN	L-Glutamine	Cytosol, Nucleus, Vacuole
glntrna	L-Glutaminyl-tRNA(Gln)	Cytosol
glp	GlycylPEPtide	Cytosol
gls	D-Glucoside	Cytosol
glu5p	L-Glutamate 5-phosphate	Cytosol
glu5sa	L-Glutamate 5emialdehyde	Cytosol, Mitochondria
gluala	5-L-Glutamyl-L-alanine	Cytosol
glucys	gamma-L-Glutamyl-L-cysteine	Cytosol
GLU	L-Glutamate	Cytosol, Mitochondria, Nucleus, Peroxisome, Vacuole
glutrna	L-Glutamyl-tRNA(Glu)	Cytosol, Mitochondria
glx	Glyoxylate	Cytosol, Peroxisome
GLY	Glycine	Cytosol, Mitochondria
glyald	D-Glyceraldehyde	Cytosol
glyc	Glycerol	Cytosol
glyc3p	Glycerol 3-phosphate	Cytosol, Mitochondria
glycogen	glycogen	Cytosol, ER, Golgi apparatus, Vacuole
glytrna	Glycyl-tRNA(Gly)	Cytosol
GMP	GMP	Cytosol, Golgi apparatus
gp4g	P1,P4-Bis(5'-guanosyl) tetraphosphate	Cytosol
grdp	Geranyl diphosphate	Cytosol
gsn	Guanosine	Cytosol, Mitochondria
gthox	Oxidized glutathione	Cytosol, Mitochondria
gthrd	Reduced glutathione	Cytosol, Mitochondria, Vacuole
GTP	GTP	Cytosol, Mitochondria
gua	Guanine	Cytosol, Mitochondria
h	H+	Cytosol, ER, Golgi apparatus, Mitochondria, Nucleus, Peroxisome, Vacuole
H2O	H2O	Cytosol, ER, Golgi apparatus, Mitochondria, Nucleus, Peroxisome, Vacuole
H2O2	Hydrogen peroxide	Cytosol, Mitochondria, Nucleus, Peroxisome
H2S	Hydrogen sulfide	Cytosol
hcit	2-Hydroxybutane-1,2,4-tricarboxylate	Mitochondria
hcit	2-Hydroxybutane-1,2,4-tricarboxylate	Nucleus
HCO3	Bicarbonate	Cytosol
HCO3	Bicarbonate	Mitochondria
HCO3	Bicarbonate	Nucleus
hcys	L-Homocysteine	Cytosol
hcys	L-Homocysteine	Peroxisome
C160	Hexadecanoate (n-C16:0)	Cytosol
C160	Hexadecanoate (n-C16:0)	Peroxisome
C161	Hexadecenoate (n-C16:1)	Cytosol
C161	Hexadecenoate (n-C16:1)	Peroxisome
C161CoA	Hexadecenoyl-CoA (n-C16:1CoA)	Cytosol
C161CoA	Hexadecenoyl-CoA (n-C16:1CoA)	Peroxisome
hdd2CoA	trans-Hexadec-2-enoyl-CoA	Peroxisome
C161ACP	cis-hexadec-9-enoyl-[acyl-carrier protein] (n-C16:1)	Cytosol
C161ACP	cis-hexadec-9-enoyl-[acyl-carrier protein] (n-C16:1)	Mitochondria
hemeA	Heme A	Mitochondria

hemeO	Heme O	Mitochondria
HXPP	all-trans-Hexaprenyl diphosphate	Cytosol
HXPP	all-trans-Hexaprenyl diphosphate	Mitochondria
hgentis	Homogentisate	Cytosol
hicit	Homoisocitrate	Mitochondria
HIS	L-Histidine	Cytosol
HIS	L-Histidine	Mitochondria
HIS	L-Histidine	Vacuole
hisp	L-Histidinol phosphate	Cytosol
histd	L-Histidinol	Cytosol
histrna	L-Histidyl-tRNA(His)	Cytosol
histrna	L-Histidyl-tRNA(His)	Mitochondria
hLkynr	3-Hydroxy-L-kynurenine	Cytosol
hmbil	Hydroxymethylbilane	Cytosol
hmgCoA	Hydroxymethylglutaryl-CoA	Cytosol
hmgCoA	Hydroxymethylglutaryl-CoA	Mitochondria
hom	L-Homoserine	Cytosol
hpac	4-Hydroxyphenylacetate	???
hpacal	4-Hydroxyphenylacetaldehyde	???
hpglu	Tetrahydropteroyltri-L-glutamate	Cytosol
hvan	Homovanillate	???
hxan	Hypoxanthine	Cytosol
hxc2CoA	trans-Hexacos-2-enoyl-CoA	Peroxisome
hxdcal	Hexadecanal	Cytosol
iad	Indole-3-acetamide	Cytosol
iamac	isoamyl acetate	Cytosol
iamoh	Isoamyl alcohol	Cytosol
iamoh	Isoamyl alcohol	Mitochondria
IMASP	Iminoaspartate	Cytosol
ibutac	isobutyl acetate	Cytosol
ibutoh	isobutyl alcohol	Cytosol
ibutoh	isobutyl alcohol	Mitochondria
icit	Isocitrate	Cytosol
icit	Isocitrate	Mitochondria
icit	Isocitrate	Peroxisome
id3acald	Indole-3-acetaldehyde	Cytosol
id3acald	Indole-3-acetaldehyde	Mitochondria
IDP	IDP	Cytosol
IDP	IDP	Mitochondria
ILE	L-Isoleucine	Cytosol
ILE	L-Isoleucine	Mitochondria
ILE	L-Isoleucine	Vacuole
iletrna	L-Isoleucyl-tRNA(Ile)	Cytosol
iletrna	L-Isoleucyl-tRNA(Ile)	Mitochondria
imacp	3-(Imidazol-4-yl)-2-oxopropyl phosphate	Cytosol
IMP	IMP	Cytosol
ind3ac	Indole-3-acetate	Cytosol
ind3ac	Indole-3-acetate	Mitochondria
ind3acnl	Indole-3-acetonitrile	Cytosol
ind3eth	Indole-3-ethanol	Cytosol
ind3eth	Indole-3-ethanol	Mitochondria
indpyr	Indolepyruvate	Cytosol
inost	myo-Inositol	Cytosol
ins	Inosine	Cytosol
ipc124	Inositol phosphorylceramide, ceramide-1 (24C)	Cytosol

ipc126	Inositol phosphorylceramide, ceramide-1 (26C)	Cytosol
ipc224	Inositol phosphorylceramide, ceramide-2 (24C)	Cytosol
ipc226	Inositol phosphorylceramide, ceramide-2 (26C)	Cytosol
ipc324	Inositol phosphorylceramide, ceramide-3 (24C)	Cytosol
ipc326	Inositol phosphorylceramide, ceramide-3 (26C)	Cytosol
IPPP	Isopentenyl diphosphate	Cytosol
IPPP	Isopentenyl diphosphate	Mitochondria
itacCoA	Itaconyl-CoA	Mitochondria
itacon	Itaconate	Mitochondria
ITP	ITP	Cytosol, Mitochondria
K	potassium	Cytosol
L2aadp	L-2-Aminoadipate	Cytosol
L2aadp6sa	L-2-Aminoadipate 6emialdehyde	Cytosol
dLAC	D-Lactate	Cytosol, Mitochondria
LAC	L-Lactate	Cytosol, Mitochondria
lald	L-Lactaldehyde	Cytosol
lanost	Lanosterol	Cytosol
lanostest	lanosterol ester	Cytosol
Lcystin	L-Cystine	Cytosol
Lcystin	L-Cystine	Vacuole
LEU	L-Leucine	Cytosol
LEU	L-Leucine	Mitochondria
LEU	L-Leucine	Vacuole
leutrna	L-Leucyl-tRNA(Leu)	Cytosol
leutrna	L-Leucyl-tRNA(Leu)	Mitochondria
Lfmkynr	L-FORM ylkynurenine	Cytosol
lgt	(R)-Lactoylglutathione	Cytosol
lgt	(R)-Lactoylglutathione	Mitochondria
Lkynr	L-Kynurenine	Cytosol
lpam	Lipoamide	Mitochondria
lpro	Lipoylprotein	Mitochondria
LYS	L-Lysine	Cytosol
LYS	L-Lysine	Mitochondria
LYS	L-Lysine	Vacuole
lystrna	L-Lysine-tRNA (Lys)	Cytosol
lystrna	L-Lysine-tRNA (Lys)	Mitochondria
m1macchitppdol	alpha-D-mannosyl-beta-D-mannosyl-diacylchitobiosyldiphosphodolichol	Golgi Apparatus
m2macchitppdol	(alpha-D-mannosyl)2-beta-D-mannosyl-diacetylchitobiosyldiphosphodolichol	Golgi Apparatus
m3macchitppdol	(alpha-D-mannosyl)3-beta-D-mannosyl-diacetylchitodiphosphodolichol	Golgi Apparatus
m4macchitppdol	(alpha-D-Mannosyl)4-beta-D-mannosyl-diacetylchitobiosyldiphosphodolichol	Golgi Apparatus
macchitppdol	beta-D-Mannosyldiacetylchitobiosyldiphosphodolichol	Golgi Apparatus
MALACP	Malonyl-[acyl-carrier protein]	Cytosol
MALACP	Malonyl-[acyl-carrier protein]	Mitochondria
MALCoA	Malonyl-CoA	Cytosol
MALCoA	Malonyl-CoA	Mitochondria
MAL	L-Malate	Cytosol
MAL	L-Malate	Mitochondria
MAL	L-Malate	Peroxisome
MALt	Maltose	Cytosol
man	D-Mannose	Cytosol
man1p	D-Mannose 1-phosphate	Cytosol
man2mi1p	Mannose-(inositol-P)2	Cytosol
man6p	D-Mannose 6-phosphate	Cytosol
manmi1p	mannose-1D-myo-Inositol 1-phosphate	Cytosol
mannan	Mannan	Cytosol

mannan	Mannan	Endoplasmic Reticulum
mant	Manninotriose	Extracellular
melib	Melibiose	Cytosol
melt	melibiitol	Extracellular
methf	5,10-Methenyltetrahydrofolate	Cytosol
methf	5,10-Methenyltetrahydrofolate	Mitochondria
MET	L-Methionine	Cytosol
MET	L-Methionine	Mitochondria
mettrna	L-Methionyl-tRNA (Met)	Cytosol
mettrna	L-Methionyl-tRNA (Met)	Mitochondria
mev-R	(R)-Mevalonate	Cytosol
mohman	3-Methoxy-4-hydroxymandelate	???
mohpacal	3-Methoxy-4-hydroxyphenylacetaldehyde	???
mohpeg	3-Methoxy-4-hydroxyphenylethyleneglycol	???
mohpgal	3-Methoxy-4-hydroxyphenylglycolaldehyde	???
mhpglu	5-Methyltetrahydropteroyltri-L-glutamate	Cytosol
mi13456p	1D-myo-Inositol 1,3,4,5,6-pentakisphosphate	Cytosol
mi13456p	1D-myo-Inositol 1,3,4,5,6-pentakisphosphate	Nucleus
mi1345p	1D-myo-Inositol 1,3,4,5-tetrakisphosphate	Nucleus
mi1456p	1D-myo-Inositol 1,4,5,6-tetrakisphosphate	Nucleus
mi145p	1D-myo-Inositol 1,4,5-trisphosphate	Cytosol
mi145p	1D-myo-Inositol 1,4,5-trisphosphate	Nucleus
mi1p	1D-myo-Inositol 1-phosphate	Cytosol
micit	methylisocitrate	Mitochondria
minohp	myo-Inositol hexakisphosphate	Cytosol
minohp	myo-Inositol hexakisphosphate	Nucleus
mip2c124	mannose-(inositol-P)2-ceramide, ceramide-1 (24C)	Cytosol
mip2c126	mannose-(inositol-P)2-ceramide, ceramide-1 (26C)	Cytosol
mip2c224	mannose-(inositol-P)2-ceramide, ceramide-2 (24C)	Cytosol
mip2c226	mannose-(inositol-P)2-ceramide, ceramide-2 (26C)	Cytosol
mip2c324	mannose-(inositol-P)2-ceramide, ceramide-3 (24C)	Cytosol
mip2c326	mannose-(inositol-P)2-ceramide, ceramide-3 (26C)	Cytosol
mipc124	mannose-inositol phosphorylceramide, ceramide-1 (24C)	Cytosol
mipc126	mannose-inositol phosphorylceramide, ceramide-1 (26C)	Cytosol
mipc224	mannose-inositol phosphorylceramide, ceramide-2 (24C)	Cytosol
mipc226	mannose-inositol phosphorylceramide, ceramide-2 (26C)	Cytosol
mipc324	mannose-inositol phosphorylceramide, ceramide-3 (24C)	Cytosol
mipc326	mannose-inositol phosphorylceramide, ceramide-3 (26C)	Cytosol
mlthf	5,10-Methylenetetrahydrofolate	Cytosol
mlthf	5,10-Methylenetetrahydrofolate	Mitochondria
mmet	S-Methyl-L-methionine	Cytosol
mnep	L-Metanephrine	???
motam	3-Methoxytyramine	????
mthgxl	Methylglyoxal	Cytosol
C140ACP	Myristoyl-ACP (n-C14:0ACP)	Cytosol
C140ACP	Myristoyl-ACP (n-C14:0ACP)	Mitochondria
N1aspmd	N1-Acetylspermidine	Cytosol
N1sprm	N1-Acetylspermine	Cytosol
n4abutn	N4-Acetylaminobutanal	Cytosol
na1	Sodium	Cytosol
nac	Nicotinate	Cytosol
nac	Nicotinate	Mitochondria
NAD	Nicotinamide adenine dinucleotide	Cytosol, ER, Mitochondria, Nucleus, Peroxisome
NADH	Nicotinamide adenine dinucleotide - reduced	Cytosol, ER, Mitochondria, Peroxisome

NADP	Nicotinamide adenine dinucleotide phosphate	Cytosol, ER, Mitochondria, Peroxisome
NADPH	Nicotinamide adenine dinucleotide phosphate - reduced	Cytosol, ER, Mitochondria, Peroxisome
Nbfortyr	N,N-bisFORM yl-dityrosine	Cytosol
ncam	Nicotinamide	Cytosol
ncam	Nicotinamide	Mitochondria
Nfortyr	N-FORM yl-L-tyrosine	Cytosol
NH4	Ammonium	Cytosol, Mitochondria, Nucleus, Peroxisome
nicrnt	Nicotinate D-ribonucleotide	Cytosol
nicrnt	Nicotinate D-ribonucleotide	Mitochondria
nmn	NMN	Cytosol
nmn	NMN	Mitochondria
nmn	NMN	Peroxisome
noadren	L-Noradrenaline	???
nomnep	L-Normetanephrine	???
NPmehis	N(pai)-Methyl-L-histidine	Cytosol
O2	O2	Cytosol, ER, Mitochondria, Peroxisome
oaa	Oxaloacetate	Cytosol
oaa	Oxaloacetate	Mitochondria
oaa	Oxaloacetate	Peroxisome
C080ACP	Octanoyl-ACP (n-C8:0ACP)	Mitochondria
C080CoA	Octanoyl-CoA (n-C8:0CoA)	Cytosol
C080CoA	Octanoyl-CoA (n-C8:0CoA)	Peroxisome
C180	octadecanoate (n-C18:0)	Cytosol
C180	octadecanoate (n-C18:0)	Peroxisome
C180ACP	Octadecanoyl-ACP (n-C18:0ACP)	Cytosol
C180ACP	Octadecanoyl-ACP (n-C18:0ACP)	Mitochondria
C181	octadecenoate (n-C18:1)	Cytosol
C182	octadecadienoate (n-C18:2)	Cytosol
C182ACP	Octadecynoyl-ACP (n-C18:2ACP)	Cytosol
C182ACP	Octadecynoyl-ACP (n-C18:2ACP)	Mitochondria
C182CoA	Octadecynoyl-CoA (n-C18:2CoA)	Cytosol
C182CoA	Octadecynoyl-CoA (n-C18:2CoA)	Peroxisome
C080	octanoate (n-C8:0)	Cytosol
C080	octanoate (n-C8:0)	Peroxisome
C181ACP	cis-octadec-11-enoyl-[acyl-carrier protein] (n-C18:1)	Cytosol
C181ACP	cis-octadec-11-enoyl-[acyl-carrier protein] (n-C18:1)	Mitochondria
od2CoA	trans-Octadec-2-enoyl-CoA	Peroxisome
C181CoA	Octadecenoyl-CoA (n-C18:1CoA)	Cytosol
C181CoA	Octadecenoyl-CoA (n-C18:1CoA)	Peroxisome
oh1	hydroxide ion	Cytosol
oh1	hydroxide ion	Mitochondria
ohpb	2-Oxo-3-hydroxy-4-phosphobutanoate	Cytosol
orn	Ornithine	Cytosol
orn	Ornithine	Mitochondria
orot	Orotate	Cytosol
orot5p	Orotidine 5'-phosphate	Cytosol
oxag	Oxaloglutarate	Cytosol
oxag	Oxaloglutarate	Mitochondria
pa	Phosphatidate	Cytosol
pa	Phosphatidate	Mitochondria
pac	Phenylacetic acid	Cytosol
pacald	Phenylacetaldehyde	Cytosol

pacald	Phenylacetaldehyde	Mitochondria
pad	2-Phenylacetamide	Cytosol
C160ACP	Palmitoyl-ACP (n-C16:0ACP)	Cytosol
C160ACP	Palmitoyl-ACP (n-C16:0ACP)	Mitochondria
pan4p	Pantetheine 4'-phosphate	Cytosol
pan4p	Pantetheine 4'-phosphate	Mitochondria
pan4p	Pantetheine 4'-phosphate	Peroxisome
pant-R	(R)-Pantoate	Cytosol
pant-R	(R)-Pantoate	Mitochondria
pap	Adenosine 3',5'-bisphosphate	Cytosol
pap	Adenosine 3',5'-bisphosphate	Mitochondria
pap	Adenosine 3',5'-bisphosphate	Peroxisome
paps	3'-Phosphoadenylyl sulfate	Cytosol
pc	Phosphatidylcholine	Cytosol
pdx5p	Pyridoxine 5'-phosphate	Cytosol
pe	phosphatidylethanolamine	Cytosol
pe	phosphatidylethanolamine	Golgi Apparatus
pe	phosphatidylethanolamine	Mitochondria
pe	phosphatidylethanolamine	Vacuole
pea	Phenylethylamine	Cytosol
PNPP	all-trans-Pentaprenyl diphosphate	Cytosol
PNPP	all-trans-Pentaprenyl diphosphate	Mitochondria
PEP	Phosphoenolpyruvate	Cytosol
PEP	Phosphoenolpyruvate	Mitochondria
PEPd	PEPtide	Cytosol
pg	Phosphatidylglycerol	Mitochondria
pgp	Phosphatidylglycerophosphate	Mitochondria
pheac	Phenethyl acetate	Cytosol
PHE	L-Phenylalanine	Cytosol
PHE	L-Phenylalanine	Mitochondria
pheme	Protoheme	Mitochondria
phetrna	L-Phenylalanyl-tRNA(Phe)	Cytosol
phetrna	L-Phenylalanyl-tRNA(Phe)	Mitochondria
phom	O-Phospho-L-homoserine	Cytosol
phpyr	Phenylpyruvate	Cytosol
phthr	O-Phospho-4-hydroxy-L-threonine	Cytosol
Pi	Phosphate	Cytosol
Pi	Phosphate	Golgi Apparatus
Pi	Phosphate	Mitochondria
Pi	Phosphate	Nucleus
Pi	Phosphate	Endoplasmic Reticulum
Pi	Phosphate	Vacuole
Pi	Phosphate	Peroxisome
C160CoA	Palmitoyl-CoA (n-C16:0CoA)	Cytosol
C160CoA	Palmitoyl-CoA (n-C16:0CoA)	Peroxisome
pnto-R	(R)-Pantothenate	Cytosol
ppbng	Porphobilinogen	Cytosol
ppCoA	Propanoyl-CoA	Mitochondria
pphn	Prephenate	Cytosol
PPi	Diphosphate	Cytosol
PPi	Diphosphate	Mitochondria
PPi	Diphosphate	Nucleus
PPi	Diphosphate	Peroxisome
ppmi12346p	5-Diphosphoinositol pentakisphosphate	Cytosol
ppmi1346p	Diphosphoinositol tetrakisphosphate	Cytosol

ppp9	Protoporphyrin	Mitochondria
pppg9	Protoporphyrinogen IX	Cytosol
pppg9	Protoporphyrinogen IX	Mitochondria
pram	5-Phospho-beta-D-ribosylamine	Cytosol
pran	N-(5-Phospho-D-ribosyl)anthranilate	Cytosol
prbamp	1-(5-Phosphoribosyl)-AMP	Cytosol
prbatp	1-(5-Phosphoribosyl)-ATP	Cytosol
prfp	1-(5-Phosphoribosyl)-5-[(5-phosphoribosylamino)methylideneamino]imidazole-4-carboxamide	Cytosol
prlp	5-[(5-phospho-1-deoxyribulos-1-ylamino)methylideneamino]-1-(5-phosphoribosyl)imidazole-4-carboxamide	Cytosol
PRO	L-Proline	Cytosol
PRO	L-Proline	Mitochondria
protrna	L-Prolyl-tRNA(Pro)	Cytosol
prpp	5-Phospho-alpha-D-ribose 1-diphosphate	Cytosol
prpp	5-Phospho-alpha-D-ribose 1-diphosphate	Mitochondria
ps	phosphatidylserine	Cytosol
ps	phosphatidylserine	Golgi Apparatus
ps	phosphatidylserine	Mitochondria
ps	phosphatidylserine	Vacuole
psd5p	Pseudouridine 5'-phosphate	Cytosol
pser	O-Phospho-Lerine	Cytosol
psph1p	Phytosphingosine 1-phosphate	Cytosol
psph1p	Phytosphingosine 1-phosphate	Endoplasmic Reticulum
psphings	Phytosphingosine	Cytosol
psphings	Phytosphingosine	Endoplasmic Reticulum
ptd134bp	phosphatidyl-1D-myo-inositol 3,4-bisphosphate	Cytosol
ptd135bp	1-Phosphatidyl-D-myo-inositol 3,5-bisphosphate	Cytosol
ptd145bp	1-Phosphatidyl-D-myo-inositol 4,5-bisphosphate	Cytosol
ptd345tp	1-Phosphatidyl-1D-myo-inositol 3,4,5-trisphosphate	Cytosol
ptd1ino	phosphatidyl-1D-myo-inositol	Cytosol
ptd1ino	phosphatidyl-1D-myo-inositol	Nucleus
ptd2meeta	Phosphatidyl-N-dimethylethanolamine	Cytosol
ptd3ino	phosphatidyl-1D-myo-3-inositol	Cytosol
ptd4ino	phosphatidyl-1D-myo-4-inositol, yeast specific	Cytosol
ptd4ino	phosphatidyl-1D-myo-4-inositol, yeast specific	Nucleus
ptdmeeta	Phosphatidyl-N-methylethanolamine	Cytosol
ptrc	Putrescine	Cytosol
pyam5p	Pyridoxamine 5'-phosphate	Cytosol
pydam	Pyridoxamine	Cytosol
pydx	Pyridoxal	Cytosol
pydx5p	Pyridoxal 5'-phosphate	Cytosol
pydxn	Pyridoxine	Cytosol
PYR	Pyruvate	Cytosol
PYR	Pyruvate	Mitochondria
PYR	Pyruvate	Peroxisome
q6	Ubiquinone-6	Mitochondria
q6h2	Ubiquinol-6	Mitochondria
quln	Quinolinate	Cytosol
quln	Quinolinate	Mitochondria
r1p	alpha-D-Ribose 1-phosphate	Cytosol
r1p	alpha-D-Ribose 1-phosphate	Mitochondria
r5p	alpha-D-Ribose 5-phosphate	Cytosol
raffin	Raffinose	Cytosol
rib	D-Ribose	Cytosol
ribflv	Riboflavin	Cytosol

ribflv	Riboflavin	Mitochondria
rnam	N-Ribosylnicotinamide	Cytosol
ru5p	D-Ribulose 5-phosphate	Cytosol
s	Sulfur	Cytosol
s17bp	Sedoheptulose 1,7-bisphosphate	Cytosol
s7p	Sedoheptulose 7-phosphate	Cytosol
saccrp	Laccharopine	Cytosol
sbt	Dorbitol	Cytosol
sbt	Lorbitol	Cytosol
scl	sirohydrochlorin	Cytosol
sdhlam	Succinyldihydrolipoamide	Mitochondria
selt	Selenite	Cytosol
seld	Selenide	Cytosol
SER	Lerine	Cytosol
SER	Lerine	Mitochondria
sertrna	Leryl-tRNA(Ser)	Cytosol
Sfglutth	S-FORM ylglutathione	Cytosol
sft	Sulfatide	Cytosol
dgala	Digalactosylceramide	Cytosol
sheme	Siroheme	Cytosol
skm	Shikimate	Cytosol
skm5p	Shikimate 5-phosphate	Cytosol
SO3	Sulfite	Cytosol
SO4	Sulfate	Cytosol
sph1p	Sphinganine 1-phosphate	Cytosol
sph1p	Sphinganine 1-phosphate	Endoplasmic Reticulum
sphgn	Sphinganine	Cytosol
sphgn	Sphinganine	Endoplasmic Reticulum
spmd	Spermidine	Cytosol
sprm	Spermine	Cytosol
SQL	Squalene	Cytosol
SQL	Squalene	Endoplasmic Reticulum
srb	Lorbose	Cytosol
ESQL	(S)qualene-2,3-epoxide	Cytosol
ESQL	(S)qualene-2,3-epoxide	Endoplasmic Reticulum
sta	Stachyose	Cytosol
C180CoA	Stearoyl-CoA (n-C18:0CoA)	Cytosol
C180CoA	Stearoyl-CoA (n-C18:0CoA)	Peroxisome
succ	Succinate	Cytosol
succ	Succinate	Mitochondria
sucCoA	Succinyl-CoA	Mitochondria
suchms	Ouccinyl-L-homoserine	Cytosol
sucr	Sucrose	Cytosol
sucr6p	Sucrose-6-phosphate	Cytosol
sucsal	Succinic semialdehyde	Cytosol
T4hcinnm	trans-4-Hydroxycinnamate	Mitochondria
TAG	Triacylglycerol, triglyceride	Cytosol
TAG	Triacylglycerol, triglyceride	Endoplasmic Reticulum
taG6P	D-Tagatose 6-phosphate	Cytosol
taGDP	D-Tagatose 1,6-biphosphate	Cytosol
tam	Tyramine	Cytosol
taur	Taurine	Cytosol
tchola	taurocholic acid	Cytosol
tchola	taurocholic acid	Vacuole
td2CoA	trans-Tetradec-2-enoyl-CoA	Peroxisome

C140CoA	Tetradecanoyl-CoA (n-C14:0CoA)	Cytosol
C140CoA	Tetradecanoyl-CoA (n-C14:0CoA)	Peroxisome
C141ACP	cis-tetradec-7-enoyl-[acyl-carrier protein] (n-C14:1)	Cytosol
C141ACP	cis-tetradec-7-enoyl-[acyl-carrier protein] (n-C14:1)	Mitochondria
C141CoA	Tetradecenoyl-CoA (n-C14:1CoA)	Cytosol
C141CoA	Tetradecenoyl-CoA (n-C14:1CoA)	Peroxisome
tglp	N-TetradecanoylglycylPEPtide	Cytosol
thf	5,6,7,8-Tetrahydrofolate	Cytosol
thf	5,6,7,8-Tetrahydrofolate	Mitochondria
thfglu	Tetrahydrofolyl-[Glu](2)	Cytosol
thm	Thiamin	Cytosol
thmmp	Thiamin monophosphate	Cytosol
thmpp	Thiamine diphosphate	Cytosol
thmpp	Thiamine diphosphate	Mitochondria
thmtp	Thiamin triphosphate	Cytosol
THR	L-Threonine	Cytosol
THR	L-Threonine	Mitochondria
thrtrna	L-Threonyl-tRNA(Thr)	Cytosol
thrtrna	L-Threonyl-tRNA(Thr)	Mitochondria
thym	Thymine	Cytosol
thymd	Thymidine	Cytosol
trdox	Oxidized thioredoxin	Cytosol
trdox	Oxidized thioredoxin	Mitochondria
trdox	Oxidized thioredoxin	Nucleus
trdox	Oxidized thioredoxin	Peroxisome
trdrd	Reduced thioredoxin	Cytosol
trdrd	Reduced thioredoxin	Mitochondria
trdrd	Reduced thioredoxin	Nucleus
trdrd	Reduced thioredoxin	Peroxisome
tre	Trehalose	Cytosol
tre	Trehalose	Vacuole
tre6p	alpha,alpha'-Trehalose 6-phosphate	Cytosol
trnaala	tRNA(Ala)	Cytosol
trnaarg	tRNA(Arg)	Cytosol
trnaarg	tRNA(Arg)	Mitochondria
trnaasn	tRNA(Asn)	Cytosol
trnaasn	tRNA(Asn)	Mitochondria
trnaasp	tRNA(Asp)	Cytosol
trnaasp	tRNA(Asp)	Mitochondria
trnacys	tRNA(Cys)	Cytosol
trnagln	tRNA(Gln)	Cytosol
trnaglu	tRNA (Glu)	Cytosol
trnaglu	tRNA (Glu)	Mitochondria
trnagly	tRNA(Gly)	Cytosol
trnahis	tRNA(His)	Cytosol
trnahis	tRNA(His)	Mitochondria
trnaile	tRNA(Ile)	Cytosol
trnaile	tRNA(Ile)	Mitochondria
trnaleu	tRNA(Leu)	Cytosol
trnaleu	tRNA(Leu)	Mitochondria
trnalys	tRNA(Lys)	Cytosol
trnalys	tRNA(Lys)	Mitochondria
trnamet	tRNA(Met)	Cytosol
trnamet	tRNA(Met)	Mitochondria
trnaphe	tRNA(Phe)	Cytosol

trnaphe	tRNA(Phe)	Mitochondria
trnapro	tRNA(Pro)	Cytosol
trnaser	tRNA(Ser)	Cytosol
trnathr	tRNA(Thr)	Cytosol
trnathr	tRNA(Thr)	Mitochondria
trnatrp	tRNA(Trp)	Cytosol
trnatrp	tRNA(Trp)	Mitochondria
trnatyr	tRNA(Tyr)	Cytosol
trnatyr	tRNA(Tyr)	Mitochondria
trnaval	tRNA(Val)	Cytosol
trnaval	tRNA(Val)	Mitochondria
TRP	L-Tryptophan	Cytosol
TRP	L-Tryptophan	Mitochondria
trptrna	L-Tryptophanyl-tRNA(Trp)	Cytosol
trptrna	L-Tryptophanyl-tRNA(Trp)	Mitochondria
C140	tetradecanoate (n-C14:0)	Cytosol
C140	tetradecanoate (n-C14:0)	Peroxisome
C141	tetradecenoate (n-C14:1)	Cytosol
C141	tetradecenoate (n-C14:1)	Peroxisome
TYR	L-Tyrosine	Cytosol
TYR	L-Tyrosine	Mitochondria
TYR	L-Tyrosine	Vacuole
TYR	L-Tyrosine	Peroxisome
tyrtrna	L-Tyrosyl-tRNA(Tyr)	Cytosol
tyrtrna	L-Tyrosyl-tRNA(Tyr)	Mitochondria
UDP	UDP	Cytosol
UDP	UDP	Nucleus
udpacgal	UDP-N-acetyl-D-galactosamine	Cytosol
udpg	UDPglucose	Cytosol
udpgal	UDPgalactose	Cytosol
udpgal	UDPgalactose	Golgi Apparatus
UMP	UMP	Cytosol
UMP	UMP	Mitochondria
UMP	UMP	Nucleus
up4u	P1,P4-Bis(5'-uridyl) tetraphosphate	Cytosol
uppg3	Uroporphyrinogen III	Cytosol
ura	Uracil	Cytosol
urdglyc	(-)-Ureidoglycolate	Cytosol
urea	Urea	Cytosol
uri	Uridine	Cytosol
UTP	UTP	Cytosol
UTP	UTP	Mitochondria
VAL	L-Valine	Cytosol
VAL	L-Valine	Mitochondria
valtrna	L-Valyl-tRNA(Val)	Cytosol
valtrna	L-Valyl-tRNA(Val)	Mitochondria
xan	Xanthine	Cytosol
XMP	Xanthosine 5'-phosphate	Cytosol
xtsn	Xanthosine	Cytosol
xp4x	P1,P4-Bis(5'-xanthosyl) tetraphosphate	Cytosol
xu5p	D-Xylulose 5-phosphate	Cytosol
xyl	D-Xylose	Cytosol
xylt	Xylitol	Cytosol
xylu	D-Xylulose	Cytosol
zym_int1	zymosterol intermediate 1	Cytosol

zym_int2	zymosterol intermediate 2	Cytosol
zymst	zymosterol	Cytosol
zymstest	zymosterol ester	Cytosol
