# Supplementary material for: Genome-scale metabolic model of the fission yeast Schizosaccharomyces pombe and the reconciliation of in silico/in vivo mutant growth
Source: BMC Syst Biol. 2012 Jul 5;6:49. doi: 10.1186/1752-0509-6-49 (PMC3390277; doi:10.1186/1752-0509-6-49)
Supplement: Additional file 2: — SpoMBEL1693 Characteristics and biomass composition. [file 1752-0509-6-49-S2.pdf]

Supporting Information 2A: SpoMBEL1693 Characteristics

Genome size	12.5 Mbps
Chromosomes	3
Number of genes annotated	4940
Number of genes represented in the metabolic model	605
Percentage of total genes	12.2%
Reaction number	1693
Metabolite number	1703

Supporting Information 2B: Biomass composition of *S. pombe* used in SpoMBEL1693

Biomass biosynthesis equation (in g for synthesis of 1 g biomass):
0.44 PROTEIN + 0.07 RNA + 0.036 DNA + 0.07 PHOSPHOLIPID + 0.03 COF + 0.110 CW + 0.28 CARBOHYDRATE -> BIOMASS

Table 1. Protein (Amino acid) composition:

Amino acid	% protein (w/w)	MW[a], g/mol	mmol/g protein
Alanine	0.0682091	71.09	0.959
Arginine	0.046196	156.20	0.296
Asparagine	0.032584	114.12	0.286
Aspartate	0.0328652	115.10	0.286
Cysteine	0.0035493	103.16	0.034
Glutamate	0.0469295	128.15	0.366
Glutamine	0.0472903	129.13	0.366
Glycine	0.0678581	57.07	1.189
Histidine	0.0344552	137.16	0.251
Isoleucine	0.0670973	113.18	0.593
Leucine	0.085981	113.18	0.760
Lysine	0.0282115	128.19	0.220
Methionine	0.0194217	131.21	0.148
Phenylalanine	0.0702684	147.19	0.477
Proline	0.1163426	97.13	1.198
Serine	0.0564002	87.09	0.648
Threonine	0.077702	101.12	0.768
Tryptophan	0.000736	186.23	0.004
Tyrosine	0.0184996	163.19	0.113
Valine	0.0794029	99.15	0.801
Energy requirement for polymerisation (ATP):			41.5

[a] water is substracted from MW to account for water excretion during peptide bond formation

Protein biosynthesis equation is therefore (in mmol for synthesis of 1 g protein):
0.959 ala_c + 0.266 arg_c + 0.286 asn_c + 0.286 asp_c + 0.034 cys_c + 0.366 gln_c + 0.366 glu_c + 1.189 gly_c + 0.251 his_c + 0.593 ile_c + 0.760 leu_c + 0.220 lys_c + 0.148 met_c + 0.477 phe_c + 1.198 pro_c + 0.648 ser_c + 0.768 thr_c + 0.004 trp_c + 0.113 tyr_c + 0.801 val_c + 41.5 atp_c -> 41.5 adp_c + 41.5 pi_c + PROTEIN

Table 2. DNA composition:
The composition of DNA was calculated from the genomic sequence of S. pombe. Energy requirement for polymerisation of triphosphates was from (Ingraham et al., 1983).

Nucleotide	mol/mol DNA	MW[a], g/mol	mmol/g DNA
dAMP	0.320	313.2	1.029
dCMP	0.180	289.2	0.579
dTMP	0.320	304.2	1.029
dGMP	0.180	329.2	0.579
Energy requirement for polymerisation (ATP):			4.40

[a] the molecular weight is the weight of the nucleotide monophosphate substracted 1 water, which is lost during esterification

DNA biosynthesis equation is therefore (in mmol for synthesis of 1 g DNA):
1.029 datp_n + 0.579 dctp_n + 1.029 dttp_n + 0.579 dgtp_n + 26.0 atp_n -> DNA + 26.0 adp_n + 26.0 pi_n + 3.223 ppi_n

Table 3. RNA composition:
It was assumed that RNA consisted of 5% mRNA, 75% rRNA and 20% tRNA (molar). The nucleotide composition of mRNA was taken as for genomic DNA. The nucleotide composition of rRNA was calculated from the sequences of 16S, 23S and 5S ribosomal RNA units. tRNA composition was found from sequences of leucine and glycine transporting RNAs. All the sequences were obtained from GenBank (http://www.ncbi.nlm.nih.gov). Energy requirement for polymerisation of triphosphates was from (Ingraham et al., 1983).

Nucleotide	mol/mol RNA			MW[a],	mol/mol	mmol/g
	mRNA 5%	rRNA 75%	tRNA 20%	g/mol	RNA	RNA
AMP	1.029	0.299	0.195	329.2	0.314	0.880
GMP	0.579	0.224	0.307	345.2	0.258	0.723
CMP	1.029	0.170	0.243	305.2	0.228	0.637
UMP	0.579	0.307	0.255	306.2	0.310	0.869
Energy requirement for polymerisation (ATP):						1.25

[a] the molecular weight is the weight of the nucleotide monophosphate substracted 1 water, which is lost during esterification

RNA biosynthesis equation is therefore (in mmol for synthesis of 1 g RNA):
0.880 atp_n + 0.723 gtp_n + 0.637 ctp_n + 0.869 utp_n + 1.25 atp_n -> RNA + 1.25 adp_n + 1.25 pi_n + 3.119 ppi_n

Table 4. Phospholipids composition:

Component	g/g phospholipids	mmol/g phospholipids
Phosphatidylinositol	0.20	0.228
Phosphatidylcholine	0.40	0.511
Phosphatidylserine	0.03	0.038
Phosphatidylethanolami	0.24	0.321
Phosphatidylglycerol	0.18	0.233
Phosphatidic acid	0.02	0.025
Fecosterol	0.02	0.012
Cardiolipin	0.04	0.025

Phospholipids biosynthesis equation is therefore (in mmol for synthesis of 1 g phospholipids):
0.229 ptd1ino_c + 0.514 pc_c + 0.038 ps_c + 0.324 pe_c + 0.235 pg_m + 0.026 pa_c + 0.026 cl_m + 0.012 fecost_r -> PHOSPHOLIPID

Table 4.1 Molecular weights of phospholipids components:

Constituent	MW, g/mol backbone	# of fatty acids residues	total
Fecosterol	398.664	0	398.66
Phosphatidylinositol	300.200	2	860.45
Phosphatidylcholine	223.207	2	783.45
Phosphatidylserine	223.121	2	783.37
Phosphatidylethanolami	181.128	2	741.37
Phosphatidylglycerol	212.139	2	772.39
Phosphatidic acid	228.094	2	788.34
Cardiolipin	332.183	4	1452.68

Table 4.2 Composition of fatty acids in phospholipids:

The composition of fatty acids tails in phospholipids was taken from Koukou AI et al 1990

Fatty acid	g/g total fatty acids	MW[a], g/mol	mmol/g total fatty acids	mol/mol total fatty acids
C12	0.004	200	0.02	0.006
C14	0.003	228	0.01	0.004
C16	0.104	255	0.41	0.114
C16:1	0.011	253	0.04	0.012
C18	0.055	283	0.19	0.054
C18:1	0.818	281	2.91	0.815
Average molecular weigl		280	SUM:	1.00

[a] molecular weight without a proton

Example: phosphatidylethanolamine biosynthesis equations are therefore (in mol):

GL3P_c + 0.006 C120ACP_c + 0.004 C140ACP_c + 0.114 C160ACP_c + 0.012 C161ACP_c + 0.054 C180ACP_c + 0.815 C181ACP_c -> AGL3P_c + ACP_

AGL3P_c + 0.006 C120ACP_c + 0.004 C140ACP_c + 0.114 C160ACP_c + 0.012 C161ACP_c + 0.054 C180ACP_c + 0.815 C181ACP_c -> pa_SP_c + ACP

pa_SP_c + ctp_c <-> CDPDG_c + ppi_c

CDPDG_c + ser_c <-> cmp_c + ps_SP_c

ps_SP_c -> pe_SP_c + co2_c

Table 5. Small molecules pool composition:

For simplification is was assumed that the selected small molecules are equally represented (w/w) in the pool

Molecule	MW, g/mol	g/g pool of small molecules	mmol/g pool of small molecules
NAD	664.438	0.125	0.188
NADP	744.418	0.125	0.168
COA	767.534	0.125	0.163
Q	853.365	0.125	0.146
THF	445.434	0.125	0.281
hemeA	852.837	0.125	0.147
FMN	456.348	0.125	0.274
FAD	785.557	0.125	0.159

[a] molecular mass of recombinant *E. coli* acyl carrier protein (Sigma-Aldrich)

Small molecules pool biosynthesis equation is therefore (in mmol for synthesis of 1 g SMALL MOLECULES):

0.188 nad_c + 0.168 nadp_c + 0.163 coa_c + 0.146 q_m + 0.281 thf_c + 0.274 fmn_c + 0.159 fad_c + 0.147 hemeA_m -> COF

Table 6. Carbohydrates composition:

Isolation and characterization of *Schizosaccharomyces pombe* mutants defective in cell wall (1-3)beta-D-glucan - Ribas JC et al 1991

Component	Molar ratio	MW[a], g/mol	mmol/g carbohydrate
Galactomannan	0.1	325	0.709
a-Glucan	0.3	162	1.843
b-Glucan	0.5	162	2.902

[a] the molecular weight is substracted water to account for the bond formation

Carbohydrates biosynthesis equation is therefore (in mmol for synthesis of 1 g carbohydrates):

0.709 galcman_c + 1.843 Adglcn_c + 2.902 13BDglcn_c + 12.8 atp_c -> CARBOHYDRATE + 12.8 adp_c + 12.8 pi_c

gal_c + man_c -> galcman_c

References

Effect of ethanol on the phospholipid and fatty acid content of *S pombe* membranes - Koukou AI et al 1990

Analysis of a genome-wide set of gene deletions in the fission yeast *Schizosaccharomyces pombe* - Kim DU et al 2010

The molecular structures of some glucans from the cell walls of *S pombe* - Manners DJ and Meyer MT 1977

Isolation and characterization of *Schizosaccharomyces pombe* mutants defective in cell wall (1-3)beta-D-glucan - Ribas JC et al 1991
