# Supplementary material for: Genome-scale metabolic model of the fission yeast Schizosaccharomyces pombe and the reconciliation of in silico/in vivo mutant growth
Source: BMC Syst Biol. 2012 Jul 5;6:49. doi: 10.1186/1752-0509-6-49 (PMC3390277; doi:10.1186/1752-0509-6-49)
Supplement: Additional file 3: — Additional validation studies for SpoMBEL1693 - Carbon source utilization and Flux Variability Analysis of ethanol production capacity. [file 1752-0509-6-49-S3.pdf]

Supporting Information 3A: Carbon source utilization validation - Test whether *S. pombe* is capbabile of utilizing a substrate as a sole source of carbon

Carbon source	Experimental results	*In silico*	Carbon source	Experimental results	*In silico*
Glucose	+	+	D Arabinose	-	-
Maltose	+	+	Erythritol	-	-
Sucrose	+	+	Ethanol	-	-
Cysteine	+	+	Galactitol	-	-
Fructose	+	+	Glucosamine	-	-
Tyrosine	+	+	Inositol	-	-
Glucose/Ethanol	+	+	L Arabinose	-	-
13-Propanediol	-	-	Mannitol	-	-
Acetate	-	-	Melibiose	-	-
Alanine	-	-	Melizitose	-	-
Arabinose	-	-	Methanol	-	-
Asparagine	-	-	Rhamnose	-	-
Butanol	-	-	Ribitol	-	-
Citrate	-	-	Ribose	-	-
Galactose	-	-	Salicin	-	-
Gluctamate	-	-	Soluble Starch	-	-
Glutamine	-	-	Sorbitol	-	-
Glycerol	-	-	Sorbose	-	-
Glycine	-	-	Succinic Acid	-	-
Lactate	-	-	Trehalose	-	-
Lactose	-	-			
Leucine	-	-			
Malate	-	-			
Methionine	-	-			
Phenylalanine	-	-			
Proline	-	-			
Serine	-	-			
Xylose	-	-			
Cellobiose	-	-			

Isolation and Characterization of Ethanol-Producing S. pombe CHFY0201 - Choi GW et al 2010

Metabolic fluxes in chemostat cultures of S pombe grown on mixtures of glucose and ethanol - de Jong-Gubbels P et al 1996

Physiological characterization and fed-batch production of an extracellular maltase of Schizosaccharomyces pombe CBS 356 - Jansen ML et al 2006

Supporting Information 3B: Analysis of SpoMBEL1693 in Ethanol secretion at various dilution rates.

Qualitative and quantitative validation of SpoMBEL1693 through Flux Variability Analysis (FVA) of ethanol production and comparison with the observed results at different dilution rates in a glucose limiting chemo-stat culture of *S. pombe*. FVA is employed in this analysis due to the multiple solutions that can be achieved using FBA and the variability of *in vivo* culture conditions that is experienced with each culture. Furthermore, with the absence of regulatory control information of the metabolic network, direct comparison of *in vivo* and *in silico* flux values is impractical.

In Figure 1, FVA is used to generate a range of fluxes in which *S. pombe* can achieve for ethanol production at a given dilution rate and glucose uptake rate. This range of possible fluxes for ethanol secretion is compared to the results from the observed ethanol secretion rates of ethanol in de Jong-Gubbels et al., 1996, from which the data and constraints used in the analysis were taken from. As seen in the figure, the observed ethanol secretion rates fall within the possible flux values that can be attained for ethanol secretion.

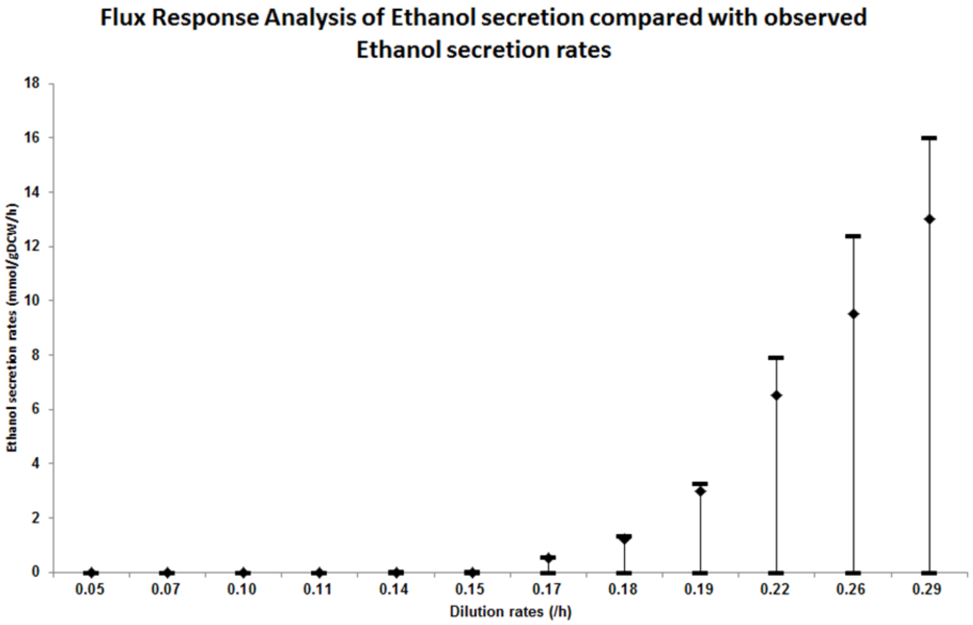

Figure1: Comparison between the observed ethanol secretion rate and the range of possible secretion rates determined from SpoMBEL1693. Diamonds are the observed ethanol secretion rates taken from de Jong-Gubbels et al., 1996 and the dashes connected by a line are the maximum and minimum ethanol secretion rates possible based on FVA.
