# Supplementary material for: Genome-scale metabolic model of the fission yeast Schizosaccharomyces pombe and the reconciliation of in silico/in vivo mutant growth
Source: BMC Syst Biol. 2012 Jul 5;6:49. doi: 10.1186/1752-0509-6-49 (PMC3390277; doi:10.1186/1752-0509-6-49)
Supplement: Additional file 4: — List of False positive and False negative predictions from single knockout simulation using SpoMBEL1693. [file 1752-0509-6-49-S4.pdf]

Supporting Information 4A: List of False postive predictions from single knockout simulation using SpoMBEL1693

Metabolic Reaction	Gene	False positive reconcilitation
GTP_n + H2O_n -> ahdt_n + FORM_n + h_n	SPAC17A5.13	Redundant reaction in a separate compartment. Deletion of redundant reaction gives lethal phenotype
3ig3p_n + ser_n -> g3p_n + H2O_n + trp_n	SPAC19A8.15	Redundant reaction in a separate compartment. Deletion of redundant reaction gives lethal phenotype
ATP_n + glu_n + NH4_n -> ADP_n + gln_n + h_n + pi_n	SPAC23H4.06	Redundant reaction in a separate compartment. Deletion of redundant reaction gives lethal phenotype
5dpmev_n + ATP_n -> ADP_n + CO2_n + IPPP_n + pi_n	SPAC24C9.03	Redundant reaction in a separate compartment. Deletion of redundant reaction gives lethal phenotype
2pg_n <-> 3pg_n	SPAC26F1.06	Redundant reaction in a separate compartment. Deletion of redundant reaction gives lethal phenotype
coa_n + h_n + hmgcoa_n <-> aacoa_n + accoa_n + H2O_n	SPAC4F8.14c	Redundant reaction in a separate compartment. Deletion of redundant reaction gives lethal phenotype
coa_r + h_r + hmgcoa_r <-> aacoa_r + accoa_r + H2O_r	SPAC4F8.14c	Redundant reaction in a separate compartment. Deletion of redundant reaction gives lethal phenotype
ATP_c + h_c + nmn_c -> NAD_c + PPi_c	SPAC806.06c	Redundant reaction in a separate compartment. Deletion of redundant reaction gives lethal phenotype
ATP_n + h_n + nicrnt_n -> dNAD_n + PPi_n	SPAC806.06c	Redundant reaction in a separate compartment. Deletion of redundant reaction gives lethal phenotype
ATP_n + h_n + nmn_n -> NAD_n + PPi_n	SPAC806.06c	Redundant reaction in a separate compartment. Deletion of redundant reaction gives lethal phenotype
H2O_c + phthr_c -> 4hthr_c + pi_c	SPAC9E9.06c	Redundant reaction in a separate compartment. Deletion of redundant reaction gives lethal phenotype
H2O_n + phom_n -> pi_n + thr_n	SPAC9E9.06c	Redundant reaction in a separate compartment. Deletion of redundant reaction gives lethal phenotype
H2O_n + phthr_n -> 4hthr_n + pi_n	SPAC9E9.06c	Redundant reaction in a separate compartment. Deletion of redundant reaction gives lethal phenotype
GTP_n + 3 H2O_n -> 25dhpp_n + FORM_n + 2 h_n + PPi_n	SPAP27G11.09c	Redundant reaction in a separate compartment. Deletion of redundant reaction gives lethal phenotype
IPPP_n <-> dmpp_n	SPBC106.15	Redundant reaction in a separate compartment. Deletion of redundant reaction gives lethal phenotype
3pg_n + ATP_n <-> 13dpg_n + ADP_n	SPBC14F5.04c	Redundant reaction in a separate compartment. Deletion of redundant reaction gives lethal phenotype
ATP_n + H2O_n + met_n -> SAM_n + pi_n + PPi_n	SPBC14F5.05c	Redundant reaction in a separate compartment. Deletion of redundant reaction gives lethal phenotype
anth_n + prpp_n -> PPi_n + pran_n	SPBC16G5.08	Redundant reaction in a separate compartment. Deletion of redundant reaction gives lethal phenotype
25dhpp_n + h_n + NADPH_n -> 25dthpp_n + NADP_n	SPBC21C3.10c	Redundant reaction in a separate compartment. Deletion of redundant reaction gives lethal phenotype
ru5pD_m -> db4p_m + FORM_m + h_m	SPBC23E6.06c	Redundant reaction in a separate compartment. Deletion of redundant reaction gives lethal phenotype
ru5pD_n -> db4p_n + FORM_n + h_n	SPBC23E6.06c	Redundant reaction in a separate compartment. Deletion of redundant reaction gives lethal phenotype
4r5au_n + db4p_n -> dmlz_n + 2 H2O_n + pi_n	SPBC409.13	Redundant reaction in a separate compartment. Deletion of redundant reaction gives lethal phenotype
3psme_n -> CHOR_n + pi_n	SPCC1223.14	Redundant reaction in a separate compartment. Deletion of redundant reaction gives lethal phenotype
coa_n + mev-R_n + 2 NADP_n <-> 2 h_n + hmgcoa_n + 2 NADPH_n	SPCC162.09c	Redundant reaction in a separate compartment. Deletion of redundant reaction gives lethal phenotype
coa_r + mev-R_r + 2 NADP_r <-> 2 h_r + hmgcoa_r + 2 NADPH_r	SPCC162.09c	Redundant reaction in a separate compartment. Deletion of redundant reaction gives lethal phenotype
ATP_m + ribflv_m -> ADP_m + fmn_m + h_m	SPCC18.16c	Redundant reaction in a separate compartment. Deletion of redundant reaction gives lethal phenotype
ATP_n + ribflv_n -> ADP_n + fmn_n + h_n	SPCC18.16c	Redundant reaction in a separate compartment. Deletion of redundant reaction gives lethal phenotype
ATP_n + dNAD_n + NH4_n -> amp_n + h_n + NAD_n + PPi_n	SPCC553.02	Redundant reaction in a separate compartment. Deletion of redundant reaction gives lethal phenotype
ATP_n + dtmp_n <-> ADP_n + dtdp_n	SPCC70.07c	Redundant reaction in a separate compartment. Deletion of redundant reaction gives lethal phenotype
g1p_n <-> G6P_n	SPBC32F12.10, SPCC1840.05c	Redundant reaction in a separate compartment. Deletion of redundant reaction gives lethal phenotype
r1p_n <-> r5p_n	SPBC32F12.10, SPCC1840.05c	Redundant reaction in a separate compartment. Deletion of redundant reaction gives lethal phenotype
25dthpp_m + h_m + H2O_m -> 5aprbu_m + NH4_m	SPAC18B11.02c SPCC4G3.16	Redundant reaction in a separate compartment. Deletion of redundant reaction gives lethal phenotype
25dthpp_n + h_n + H2O_n -> 5aprbu_n + NH4_n	SPAC18B11.02c SPCC4G3.16	Redundant reaction in a separate compartment. Deletion of redundant reaction gives lethal phenotype
dmpp_n + IPPP_n -> grdp_n + PPi_n	SPBC36.06c, SPAC6F12.13c	Redundant reaction in a separate compartment. Deletion of redundant reaction gives lethal phenotype
grdp_n + IPPP_n -> FRPP_n + PPi_n	SPBC36.06c, SPAC6F12.13c	Redundant reaction in a separate compartment. Deletion of redundant reaction gives lethal phenotype
cit_c <-> icit_c	SPAC24C9.06c, SPBP4H10.15	Redundant reaction in a separate compartment. Deletion of redundant reaction gives lethal phenotype
cit_m <-> icit_m	SPAC24C9.06c, SPBP4H10.15	Redundant reaction in a separate compartment. Deletion of redundant reaction gives lethal phenotype
e4p_n + xu5pD_n <-> f6p_n + g3p_n	SPBC2G5.05	Redundant reaction in a separate compartment. Deletion of redundant reaction gives lethal phenotype
r5p_n + xu5pD_n <-> g3p_n + s7p_n	SPBC2G5.05	Redundant reaction in a separate compartment. Deletion of redundant reaction gives lethal phenotype
ru5pD_n <-> xu5pD_n	SPAC31G5.05c	Redundant reaction in a separate compartment. Deletion of redundant reaction gives lethal phenotype
H2O_n + pap_n -> amp_n + pi_n	SPCC1753.04	Redundant reaction in a separate compartment. Deletion of redundant reaction gives lethal phenotype
ADP_c + trdrd_c -> dADP_c + H2O_c + trdox_c	SPBC25D12.04+SPAC1F7.05	Gene assignment revised
ADP_n + trdrd_n -> dADP_n + H2O_n + trdox_n	SPBC25D12.04+SPAC1F7.05	Gene assignment revised
CDP_c + trdrd_c -> dCDP_c + H2O_c + trdox_c	SPBC25D12.04+SPAC1F7.05	Gene assignment revised
CDP_n + trdrd_n -> dCDP_n + H2O_n + trdox_n	SPBC25D12.04+SPAC1F7.05	Gene assignment revised

GDP_c + trdrd_c -> dGDP_c + H2O_c + trdox_c	SPBC25D12.04+SPAC1F7.05	Gene assignment revised
GDP_n + trdrd_n -> dGDP_n + H2O_n + trdox_n	SPBC25D12.04+SPAC1F7.05	Gene assignment revised
UDP_c + trdrd_c -> dUDP_c + H2O_c + trdox_c	SPBC25D12.04+SPAC1F7.05	Gene assignment revised
UDP_n + trdrd_n -> dUDP_n + H2O_n + trdox_n	SPBC25D12.04+SPAC1F7.05	Gene assignment revised
2ahhmd_c + PABA_c -> dhpt_c + PPi_c	SPBC1734.03	Deletion of other reactions with the same gene results in lethal phenotype
2ahhmd_m + PABA_m -> dhpt_m + PPi_m	SPBC1734.03	Deletion of other reactions with the same gene results in lethal phenotype
2ahhmp_c + ATP_c -> 2ahhmd_c + amp_c + h_c	SPBC1734.03	Deletion of other reactions with the same gene results in lethal phenotype
2ahhmp_c + PABA_c -> dhpt_c + H2O_c	SPBC1734.03	Deletion of other reactions with the same gene results in lethal phenotype
2ahhmp_m + ATP_m -> 2ahhmd_m + amp_m + h_m	SPBC1734.03	Deletion of other reactions with the same gene results in lethal phenotype
2ahhmp_m + PABA_m -> dhpt_m + H2O_m	SPBC1734.03	Deletion of other reactions with the same gene results in lethal phenotype
dhnpt_c -> 2ahhmp_c + gcald_c + h_c	SPBC1734.03	Deletion of other reactions with the same gene results in lethal phenotype
dhnpt_m -> 2ahhmp_m + gcald_m + h_m	SPBC1734.03	Deletion of other reactions with the same gene results in lethal phenotype
G6P_c <-> f6p_c	SPBC1604.05	Deletion of other reactions with the same gene results in lethal phenotype
G6P_c <-> G6P-B_c	SPBC1604.05	Deletion of other reactions with the same gene results in lethal phenotype
G6P-B_c <-> f6p_c	SPBC1604.05	Deletion of other reactions with the same gene results in lethal phenotype
2 dmlz_c -> 4r5au_c + ribflv_c	SPCC1450.13c	Deletion of other reactions with the same gene results in lethal phenotype
2 dmlz_n -> 4r5au_n + ribflv_n	SPCC1450.13c	Deletion of other reactions with the same gene results in lethal phenotype
ATP_c + gln_c + H2O_c + UTP_c -> ADP_c + CTP_c + glu_c + 2 h_c + pi_c	SPAC10F6.03c	Deletion of other reactions with the same gene results in lethal phenotype
5pmev_c + ATP_c -> 5dpmev_c + ADP_c	SPAC343.01c	Deletion of other reactions with the same gene results in lethal phenotype
5pmev_n + ATP_n -> 5dpmev_n + ADP_n	SPAC343.01c	Deletion of other reactions with the same gene results in lethal phenotype
2 h_n + mlTHF_n + NADPH_n -> 5mTHF_n + NADP_n	SPAC56F8.10	Deletion of other reactions with the same gene results in lethal phenotype
CTP_m + h_m + pa_m <-> CDPdag_m + PPi_m	SPBC13A2.03	Deletion of other reactions with the same gene results in lethal phenotype
2 accoa_c -> aacoa_c + coa_c	SPBC215.09c	Deletion of other reactions with the same gene results in lethal phenotype
ATP_n + dump_n <-> ADP_n + dUDP_n	SPCC1795.05c	Deletion of other reactions with the same gene results in lethal phenotype
ATP_n + ump_n <-> ADP_n + UDP_n	SPCC1795.05c	Deletion of other reactions with the same gene results in lethal phenotype
amp_c + ATP_c <-> 2 ADP_c	SPAC4G9.03	Deletion of other reactions with the same gene results in lethal phenotype
amp_c + GTP_c <-> ADP_c + GDP_c	SPAC4G9.03	Deletion of other reactions with the same gene results in lethal phenotype
amp_c + itp_c <-> ADP_c + idp_c	SPAC4G9.03	Deletion of other reactions with the same gene results in lethal phenotype
amp_m + ATP_m <-> 2 ADP_m	SPAC4G9.03	Deletion of other reactions with the same gene results in lethal phenotype
amp_m + GTP_m <-> ADP_m + GDP_m	SPAC4G9.03	Deletion of other reactions with the same gene results in lethal phenotype
amp_m + itp_m <-> ADP_m + idp_m	SPAC4G9.03	Deletion of other reactions with the same gene results in lethal phenotype
amp_n + ATP_n <-> 2 ADP_n	SPAC4G9.03	Deletion of other reactions with the same gene results in lethal phenotype
amp_n + GTP_n <-> ADP_n + GDP_n	SPAC4G9.03	Deletion of other reactions with the same gene results in lethal phenotype
amp_n + itp_n <-> ADP_n + idp_n	SPAC4G9.03	Deletion of other reactions with the same gene results in lethal phenotype
ATP_c + mev-R_c -> 5pmev_c + ADP_c + h_c	SPAC13G6.11c	Deletion of other reactions with the same gene results in lethal phenotype
ATP_n + mev-R_n -> 5pmev_n + ADP_n + h_n	SPAC13G6.11c	Deletion of other reactions with the same gene results in lethal phenotype
CTP_c + mev-R_c -> 5pmev_c + CDP_c + h_c	SPAC13G6.11c	Deletion of other reactions with the same gene results in lethal phenotype
CTP_n + mev-R_n -> 5pmev_n + CDP_n + h_n	SPAC13G6.11c	Deletion of other reactions with the same gene results in lethal phenotype
GTP_c + mev-R_c -> 5pmev_c + GDP_c + h_c	SPAC13G6.11c	Deletion of other reactions with the same gene results in lethal phenotype
GTP_n + mev-R_n -> 5pmev_n + GDP_n + h_n	SPAC13G6.11c	Deletion of other reactions with the same gene results in lethal phenotype
UTP_c + mev-R_c -> 5pmev_c + UDP_c + h_c	SPAC13G6.11c	Deletion of other reactions with the same gene results in lethal phenotype
UTP_n + mev-R_n -> 5pmev_n + UDP_n + h_n	SPAC13G6.11c	Deletion of other reactions with the same gene results in lethal phenotype
ATP_c + NH4_c + UTP_c -> ADP_c + CTP_c + 2 h_c + pi_c	SPAC10F6.03c	Deletion of other reactions with the same gene results in lethal phenotype
2 h_m + mlTHF_m + NADPH_m -> 5mTHF_m + NADP_m	SPAC56F8.10	Deletion of other reactions with the same gene results in lethal phenotype
CTP_c + h_c + pa_c <-> CDPdag_c + PPi_c	SPBC13A2.03	Deletion of other reactions with the same gene results in lethal phenotype
2 accoa_m -> aacoa_m + coa_m	SPBC215.09c	Deletion of other reactions with the same gene results in lethal phenotype

e4p_c + xu5pD_c <-> f6p_c + g3p_c	SPBC2G5.05	Deletion of other reactions with the same gene results in lethal phenotype
r5p_c + xu5pD_c <-> g3p_c + s7p_c	SPBC2G5.05	Deletion of other reactions with the same gene results in lethal phenotype
5pmev_m + ATP_m -> 5dpmev_m + ADP_m	SPAC343.01c	Deletion of other reactions with the same gene results in lethal phenotype
ATP_c + dump_c <-> ADP_c + dUDP_c	SPCC1795.05c	Deletion of other reactions with the same gene results in lethal phenotype
H2O_c + PPi_c -> h_c + 2 pi_c	SPAC23C11.05 SPAC3A12.02	Removed alternate reaction found in Mitochondria and added exchange flux for diphosphate between mitochondria and cytoplasm
2 h_c + mlTHF_c + NADPH_c -> 5mTHF_c + NADP_c	SPAC56F8.10, SPAC343.10	Deletion of other reactions encoded by SPAC56f8.10 results in lethal phenotype.
2 h_c + mlTHF_c + NADPH_c -> 5mTHF_c + NADP_c	SPAC56F8.10, SPAC343.10	Deletion of other reactions encoded by SPAC56f8.10 results in lethal phenotype.
2 h_r + lanost_r + 3 NADPH_r + 3 O2_r -> 44mctr_r + FORM_r + 4 H2O_r + 3 NADP_r	SPAC13A11.02c	Pathway connected to the rest of the metabolic network
2 h_c + lanost_c + 3 NADPH_c + 3 O2_c -> 44mctr_c + FORM_c + 4 H2O_c + 3 NADP_c	SPAC13A11.02c	Pathway connected to the rest of the metabolic network
Ssq23epx_c -> lanost_c	SPAC13G7.01c	Pathway connected to the rest of the metabolic network
Ssq23epx_r -> lanost_r	SPAC13G7.01c	Pathway connected to the rest of the metabolic network
44mctr_r + h_r + NADPH_r -> 44mzym_r + NADP_r	SPBC16G5.18	Pathway connected to the rest of the metabolic network
44mzym_r + 3 h_r + 3 NADPH_r + 3 O2_r -> 4mzym_int1_r + 4 H2O_r + 3 NADP_r	SPAC630.08c	Pathway connected to the rest of the metabolic network
4mzym_int1_c + NAD_c -> 4mzym_int2_c + CO2_c + h_c + NADH_c	SPBC3F6.02c	Pathway connected to the rest of the metabolic network
4mzym_int1_g + NAD_g -> 4mzym_int2_g + CO2_g + h_g + NADH_g	SPBC3F6.02c	Pathway connected to the rest of the metabolic network
4mzym_int1_r + NAD_r -> 4mzym_int2_r + CO2_r + h_r + NADH_r	SPBC3F6.02c	Pathway connected to the rest of the metabolic network
4mzym_int2_r + h_r + NADPH_r -> 4mzym_r + NADP_r	SPBC1709.07	Pathway connected to the rest of the metabolic network
4mzym_r + 3 h_r + 3 NADPH_r + 3 O2_r -> zym_int1_r + 4 H2O_r + 3 NADP_r	SPAC630.08c	Pathway connected to the rest of the metabolic network
NAD_g + zym_int1_g -> CO2_g + h_g + NADH_g + zym_int2_g	SPBC3F6.02c	Pathway connected to the rest of the metabolic network
NAD_r + zym_int1_r -> CO2_r + h_r + NADH_r + zym_int2_r	SPBC3F6.02c	Pathway connected to the rest of the metabolic network
zym_int2_r + h_r + NADPH_r -> zymst_r + NADP_r	SPBC1709.07	Pathway connected to the rest of the metabolic network
SAM_n + zymst_n -> SAH_n + fecost_n + h_n	SPBC16E9.05	Pathway connected to the rest of the metabolic network
SAM_r + zymst_r -> SAH_r + fecost_r + h_r	SPBC16E9.05	Pathway connected to the rest of the metabolic network
2 FRPP_r + h_r + NADPH_r -> NADP_r + 2 PPi_r + sql_r	SPBC646.05c	Pathway connected to the rest of the metabolic network
h_r + NADH_r + O2_r + sql_r -> Ssq23epx_r + H2O_r + NAD_r	SPBC713.12	Pathway connected to the rest of the metabolic network
h_r + NADPH_r + O2_r + sql_r -> Ssq23epx_r + H2O_r + NADP_r	SPBC713.12	Pathway connected to the rest of the metabolic network
dhap_c + 0.141 C160ACP_c + 0.015 C161ACP_c + 0.043 C180ACP_c + 0.801 C181ACP_c -> 1agly3p_c + ACP_c	SPBC1718.04	Pathway connected to the rest of the metabolic network
glyc3p_c + 0.141 C160ACP_c + 0.015 C161ACP_c + 0.043 C180ACP_c + 0.801 C181ACP_c -> 1ag3p_c + ACP_c	SPBC1718.04	Pathway connected to the rest of the metabolic network
man1p_c <-> man6p_c	SPAC1556.07	Pathway connected to the rest of the metabolic network
man1p_n <-> man6p_n	SPAC1556.07	Pathway connected to the rest of the metabolic network
dolp_c + GDPmann_c -> dolmanp_r + GDP_c	SPAC31G5.16c SPBC21B10.11 SPBC1677.02	Pathway connected to the rest of the metabolic network
man6p_c <-> f6p_c	SPBC2G2.16	Pathway connected to the rest of the metabolic network
man6p_n <-> f6p_n	SPBC2G2.16	Pathway connected to the rest of the metabolic network
GTP_c + h_c + man1p_c -> GDPmann_c + PPi_c	SPCC1906.01	Pathway connected to the rest of the metabolic network
gly_m + h_m + succoa_m -> 5aop_m + CO2_m + coa_m	SPAC2F3.09	Pathway linked to biomass by adding hemeA
hmbil_c -> H2O_c + uppg3_c	SPAC31G5.08	Pathway linked to biomass by adding hemeA
hmbil_n -> H2O_n + uppg3_n	SPAC31G5.08	Pathway linked to biomass by adding hemeA
cpppg3_c + 2 h_c + O2_c -> 2 CO2_c + 2 H2O_c + pppg9_c	SPAC222.11	Pathway linked to biomass by adding hemeA
cpppg3_m + 2 h_m + O2_m -> 2 CO2_m + 2 H2O_m + pppg9_m	SPAC222.11	Pathway linked to biomass by adding hemeA
cpppg3_n + 2 h_n + O2_n -> 2 CO2_n + 2 H2O_n + pppg9_n	SPAC222.11	Pathway linked to biomass by adding hemeA
fe2_c + ppp9_c -> 2 h_c + pheme_c	SPCC320.09	Pathway linked to biomass by adding hemeA
fe2_m + ppp9_m -> 2 h_m + pheme_m	SPCC320.09	Pathway linked to biomass by adding hemeA
fe2_n + ppp9_n -> 2 h_n + pheme_n	SPCC320.09	Pathway linked to biomass by adding hemeA
hemeO_m + NADH_m + O2_m -> H2O_m + hemeA_m + NAD_m	SPAC22E12.10c	Pathway linked to biomass by adding hemeA
thr_m -> 2obut_m + NH4_m	SPBC1677.03c	Alternate metabolic reaction found in cytoplasm. Deleteion of both reactions give lethal phenotype
r5p_c <-> ru5pD_c	SPAC144.12	Deletion of other reactions with the same gene results in lethal phenotype
ru5pD_c <-> xu5pD_c	SPAC31G5.05c	Deletion of other reactions with the same gene results in lethal phenotype
ADP_c + h_c + pep_c -> ATP_c + PYR_c	SPAC4H3.10c	Deletion of other reactions with the same gene results in lethal phenotype
accoa_m + ATP_m + hco3_m <-> ADP_m + h_m + malcoa_m + pi_m	SPAC56E4.04c	Deletion of other reactions with the same gene results in lethal phenotype
CDPdag_c + ser_c <-> cmp_c + h_c + ps_c	SPCC1442.12	Deletion of other reactions with the same gene results in lethal phenotype
H2O_c + pap_c -> amp_c + pi_c	SPCC1753.04	Deletion of other reactions with the same gene results in lethal phenotype
ATP_c + thm_c -> amp_c + h_c + thmpp_c	SPAC6F12.05c	Deletion of other reactions with the same gene results in lethal phenotype
ATP_c + thmpp_c -> ADP_c + thmtp_c	SPAC6F12.05c	Deletion of other reactions with the same gene results in lethal phenotype
dATP_c + gmp_c <-> dADP_c + GDP_c	SPBC1198.05	Deletion of other reactions with the same gene results in lethal phenotype
ATP_c + gmp_c <-> ADP_c + GDP_c	SPBC1198.05	Deletion of other reactions with the same gene results in lethal phenotype
ATP_c + f6p_c -> ADP_c + fdp_c + h_c	SPBC16H5.02	Deletion of other reactions with the same gene results in lethal phenotype
ATP_c + s7p_c -> ADP_c + h_c + s17bp_c	SPBC16H5.02	Deletion of other reactions with the same gene results in lethal phenotype
ATP_c + tag6pD_c -> ADP_c + h_c + taGDPD_c	SPBC16H5.02	Deletion of other reactions with the same gene results in lethal phenotype
6pgc_c + NADP_c -> CO2_c + NADPH_c + ru5pD_c	SPBC660.16	Deletion of other reactions with the same gene results in lethal phenotype
6pgc_m + NADP_m -> CO2_m + NADPH_m + ru5pD_m	SPBC660.16	Deletion of other reactions with the same gene results in lethal phenotype
ATP_c + 0.01 ptd1ino_c -> ADP_c + h_c + 0.01 ptd3ino_c	SPBC216.07c SPBC30D10.10C	Unreconciled
f1p_c <-> dhap_c + glyald_c	SPBC19C2.07	Unreconciled
f1p_n <-> dhap_n + glyald_n	SPBC19C2.07	Unreconciled

fdp_c <-> dhap_c + g3p_c	SPBC19C2.07	Unreconciled
fdp_n <-> dhap_n + g3p_n	SPBC19C2.07	Unreconciled
s17bp_c <-> dhap_c + e4p_c	SPBC19C2.07	Unreconciled
s17bp_n <-> dhap_n + e4p_n	SPBC19C2.07	Unreconciled
glp_c + C140coa_c -> coa_c + tglp_c	SPBC2G2.11	Unreconciled
h_m + nac_m + prpp_m -> nicrnt_m + PPi_m	SPAC1486.06	Unreconciled
h_n + nac_n + prpp_n -> nicrnt_n + PPi_n	SPAC1486.06	Unreconciled
CTP_r + dolichol_r -> CDP_r + dolp_r + h_r	SPCC63.10c	Unreconciled
ATP_c + mi13456p_c -> ADP_c + ppmi1346p_c	SPCC970.08	No flux observed
ATP_c + minohp_c -> ADP_c + ppmi12346p_c	SPCC970.08	No flux observed
ATP_c + 0.01 ptd4ino_c -> ADP_c + h_c + 0.01 ptd145bp_c	SPAC19G12.14	No flux observed
cmp_m + CTP_c + 2 h_c -> cmp_c + CTP_m + 2 h_m	SPAC688.09	No flux observed
2 h_c + ump_m + UTP_c -> 2 h_m + ump_c + UTP_m	SPAC688.09	No flux observed
GDPmann_c + gmp_g -> GDPmann_g + gmp_c	SPAC144.18	No flux observed
2 focytc_m + H2O2_m -> 2 ficytc_m + 2 H2O_m	SPCC191.07	No flux observed
cer124_g + 0.01 ptd1ino_g -> 0.01 12dgr_g + 0.01 ipc124_g	SPAC3H8.06	No flux observed
cer126_g + 0.01 ptd1ino_g -> 0.01 12dgr_g + 0.01 ipc126_g	SPAC3H8.06	No flux observed
cer224_g + 0.01 ptd1ino_g -> 0.01 12dgr_g + 0.01 ipc224_g	SPAC3H8.06	No flux observed
cer226_g + 0.01 ptd1ino_g -> 0.01 12dgr_g + 0.01 ipc226_g	SPAC3H8.06	No flux observed
cer324_g + 0.01 ptd1ino_g -> 0.01 12dgr_g + 0.01 ipc324_g	SPAC3H8.06	No flux observed
cer326_g + 0.01 ptd1ino_g -> 0.01 12dgr_g + 0.01 ipc326_g	SPAC3H8.06	No flux observed
ATP_n + mi1345p_n -> ADP_n + h_n + mi13456p_n	SPAC607.04	No flux observed
ATP_n + mi1456p_n -> ADP_n + h_n + mi13456p_n	SPAC607.04	No flux observed
ATP_n + mi145p_n -> ADP_n + h_n + mi1345p_n	SPAC607.04	No flux observed
ATP_n + mi145p_n -> ADP_n + h_n + mi1456p_n	SPAC607.04	No flux observed
ATP_c + btn_c + h_c -> btamp_c + PPi_c	SPBC30D10.07c	No flux observed
ALA_c <-> dALA_c	SPBC359.02	No flux observed
DHF_c + h_c + NADPH_c -> NADP_c + THF_c	SPCC1223.08c	No flux observed
DHF_m + h_m + NADPH_m -> NADP_m + THF_m	SPCC1223.08c	No flux observed
2 ficytc_c + dLAC_c -> 2 focytc_c + PYR_c	SPCC191.07	No flux observed
2 ficytc_m + dLAC_m -> 2 focytc_m + PYR_m	SPCC191.07	No flux observed
2 ficytc_n + dLAC_n -> 2 focytc_n + PYR_n	SPCC191.07	No flux observed
2 ficytc_m + LAC_c -> 2 focytc_m + PYR_c	SPCC191.07 SPAPB1A11.03	No flux observed
adn_c + H2O_c -> ade_c + ribD_c	SPAC17G8.02	No flux observed
adn_n + H2O_n -> ade_n + ribD_n	SPAC17G8.02	No flux observed
gsn_c + H2O_c -> gua_c + ribD_c	SPAC17G8.02	No flux observed
gsn_n + H2O_n -> gua_n + ribD_n	SPAC17G8.02	No flux observed
ATP_c + glcD_c -> ADP_c + G6P-B_c + h_c	SPAC4F8.07c	No flux observed
ATP_n + glcD_n -> ADP_n + G6P-B_n + h_n	SPAC4F8.07c	No flux observed
dUTP_c + H2O_c -> dump_c + h_c + PPi_c	SPAC644.05c	No flux observed
dUTP_n + H2O_n -> dump_n + h_n + PPi_n	SPAC644.05c	No flux observed
H2O_r + psph1p_r -> pi_r + psphings_r	SPAC823.11	No flux observed
H2O_r + sph1p_r -> pi_r + sphgn_r	SPAC823.11	No flux observed
1p3h5c_c + 2 h_c + NADH_c -> 4hpro-LT_c + NAD_c	SPAPYUG7.05	No flux observed
1p3h5c_c + 2 h_c + NADPH_c -> 4hpro-LT_c + NADP_c	SPAPYUG7.05	No flux observed
1pyr5c_c + 2 h_c + NADPH_c -> NADP_c + PRO_c	SPAPYUG7.05	No flux observed
ATP_n + dgmp_n <-> ADP_n + dGDP_n	SPBC1198.05	No flux observed
ATP_n + gmp_n <-> ADP_n + GDP_n	SPBC1198.05	No flux observed
dATP_n + gmp_n <-> dADP_n + GDP_n	SPBC1198.05	No flux observed
H2O_c + q6_m + spmd_c -> 13dampp_c + 4abutn_c + q6h2_m	SPBC1271.04c	No flux observed
H2O_n + q6_m + spmd_n -> 13dampp_n + 4abutn_n + q6h2_m	SPBC1271.04c	No flux observed
f6p_c + gln_c -> gam6p_c + glu_c	SPBC12C2.11	No flux observed
ser_c -> NH4_c + PYR_c	SPBC1677.03c	No flux observed
ATP_n + glu_n + THF_n <-> ADP_n + h_n + pi_n + THFglu_n	SPBC1709.17	No flux observed
ade_c + h_c + H2O_c -> hxan_c + NH4_c	SPBC1198.02	No flux observed
ade_n + h_n + H2O_n -> hxan_n + NH4_n	SPBC1198.02	No flux observed
adn_c + h_c + H2O_c -> ins_c + NH4_c	SPBC1198.02	No flux observed
adn_n + h_n + H2O_n -> ins_n + NH4_n	SPBC1198.02	No flux observed
gthox_c + h_c + NADPH_c -> 2 gthrd_c + NADP_c	SPBC17A3.07, SPAC4F10.20, SPAC15E1.09, SPBC26H8.06	Both viable and lethal genes associated - Removed from analysis
gthox_n + h_c + NADPH_n -> 2 gthrd_n + NADP_n	SPBC17A3.07, SPAC4F10.20, SPAC15E1.09, SPBC26H8.06	Both viable and lethal genes associated - Removed from analysis
G6P_c + NADP_c -> 6pgl_c + h_c + NADPH_c	SPCC794.01c, SPAC3C7.13c, SPAC3A12.18	Both viable and lethal genes associated - Removed from analysis
dad_c + h_c + H2O_c -> din_c + NH4_c	SPBC1683.02, SPBC1198.02	Both viable and lethal genes associated - Removed from analysis
dad_n + h_n + H2O_n -> din_n + NH4_n	SPBC1683.02, SPBC1198.02	Both viable and lethal genes associated - Removed from analysis
h_c + C160coa_c + ser_c -> 3dsphgn_c + CO2_c + coa_c	SPAC21E11.08+SPBC18E5.02c	Both viable and lethal genes associated - Removed from analysis
h_r + C160coa_r + ser_r -> 3dsphgn_r + CO2_r + coa_r	SPAC21E11.08+SPBC18E5.02c	Both viable and lethal genes associated - Removed from analysis
ATP_c + glu_c + THF_c <-> ADP_c + h_c + pi_c + THFglu_c	SPAC227.09, SPBC1709.17	Both viable and lethal genes associated - Removed from analysis
ATP_m + glu_m + THF_m <-> ADP_m + h_m + pi_m + THFglu_m	SPAC227.09, SPBC1709.17	Both viable and lethal genes associated - Removed from analysis
13dpg_c <-> 23dpg_c + h_c	SPAC26F1.06, SPAC1687.21	Both viable and lethal genes associated - Removed from analysis
13dpg_n <-> 23dpg_n + h_n	SPAC26F1.06, SPAC1687.21	Both viable and lethal genes associated - Removed from analysis
H2O2_c + trdrd_c -> 2 H2O_c + trdox_c	SPCC576.03c SPAC7D4.07c	Both viable and lethal genes associated - Removed from analysis
UDPg_c -> 16BDglcn_c + h_c + UDP_c	SPAC23H3.11c, SPAC17G6.11c	Both viable and lethal genes associated - Removed from analysis
g1p_c + h_c + UTP_c <-> PPi_c + UDPg_c	SPCC794.10, SPCC1322.04	Both viable and lethal genes associated - Removed from analysis
g1p_n + h_n + UTP_n <-> PPi_n + UDPg_n	SPCC794.10, SPCC1322.04	Both viable and lethal genes associated - Removed from analysis
dhlam_m + NAD_m -> h_m + lpam_m + NADH_m	SPBC3H7.03c, SPAC1002.09c, SPBC776.15c	Both viable and lethal genes associated - Removed from analysis
coa_m + NAD_m + PYR_m -> accoa_m + CO2_m + NADH_m	SPAC1002.09c, SPCC794.07, SPBC30D10.13c+SPAC26F1.03, SPCC1259.09c	Both viable and lethal genes associated - Removed from analysis
C160ACP_c + H2O_c <-> ACP_c + h_c + C160_c	SPAC926.09c+ SPAC4A8.11c	Both viable and lethal genes associated - Removed from analysis
C161ACP_c + H2O_c <-> ACP_c + h_c + C161_c	SPAC926.09c+ SPAC4A8.11c	Both viable and lethal genes associated - Removed from analysis
C180ACP_c + H2O_c <-> ACP_c + h_c + C180_c	SPAC926.09c+ SPAC4A8.11c	Both viable and lethal genes associated - Removed from analysis
C181ACP_c + H2O_c <-> ACP_c + h_c + C181_c	SPAC926.09c+ SPAC4A8.11c	Both viable and lethal genes associated - Removed from analysis
ACP_c + accoa_c <-> acACP_c + coa_c	SPBC1105.15c SPAC4H3.09 SPAC3G9.02 SPAC11G7.05c	Both viable and lethal genes associated - Removed from analysis

C160ACP_m + 4 h_m + malACP_m + 3 NADPH_m + O2_m -> ACP_m + CO2_m + 3 H2O_m + 3 NADP_m + C181ACP_m	SPBC1105.15c SPAC4H3.09 SPAC3G9.02 SPAC11G7.05c SPAC26F1.04c	Both viable and lethal genes associated - Removed from analysis
C140ACP_m + 3 h_m + malACP_m + 2 NADPH_m -> ACP_m + CO2_m + H2O_m + 2 NADP_m + C160ACP_m	SPBC1105.15c SPAC4H3.09 SPAC3G9.02 SPAC11G7.05c SPAC26F1.04c	Both viable and lethal genes associated - Removed from analysis
C120ACP_m + 3 h_m + malACP_m + 2 NADPH_m -> ACP_m + CO2_m + H2O_m + 2 NADP_m + C140ACP_m	SPBC1105.15c SPAC4H3.09 SPAC3G9.02 SPAC11G7.05c SPAC26F1.04c	Both viable and lethal genes associated - Removed from analysis
acACP_m + 9 h_m + 3 malACP_m + 6 NADPH_m -> 3 ACP_m + 3 CO2_m + 3 H2O_m + 6 NADP_m + C080ACP_m	SPBC1105.15c SPAC4H3.09 SPAC3G9.02 SPAC11G7.05c SPAC26F1.04c	Both viable and lethal genes associated - Removed from analysis
C080ACP_m + 3 h_m + malACP_m + 2 NADPH_m -> ACP_m + CO2_m + H2O_m + 2 NADP_m + C100ACP_m	SPBC1105.15c SPAC4H3.09 SPAC3G9.02 SPAC11G7.05c SPAC26F1.04c	Both viable and lethal genes associated - Removed from analysis
C100ACP_m + 3 h_m + malACP_m + 2 NADPH_m -> ACP_m + CO2_m + H2O_m + 2 NADP_m + C120ACP_m	SPBC1105.15c SPAC4H3.09 SPAC3G9.02 SPAC11G7.05c SPAC26F1.04c	Both viable and lethal genes associated - Removed from analysis
ACP_m + malcoa_m <-> coa_m + malACP_m	SPBC1105.15c SPAC4H3.09 SPAC3G9.02 SPAC11G7.05c SPAC26F1.04c	Both viable and lethal genes associated - Removed from analysis
r1p_c <-> r5p_c	SPBC32F12.10, SPCC1840.05c	Both viable and lethal genes associated - Removed from analysis
thr_c -> 2obut_c + NH4_c	SPCC320.14 SPBC1677.03c	Both viable and lethal genes associated - Removed from analysis
ATP_c + glcD_c -> ADP_c + G6P_c + h_c	SPAC4F8.07c, SPAC24H6.04	Both viable and lethal genes associated - Removed from analysis
ATP_n + glcD_n -> ADP_n + G6P_n + h_n	SPAC4F8.07c, SPAC24H6.04	Both viable and lethal genes associated - Removed from analysis
C080_c + 3 h_c + malcoa_c + 2 NADPH_c -> CO2_c + coa_c + H2O_c + 2 NADP_c + C100_c	SPAC56E4.04c, SPAC926.09c+SPAC4A8.11c	Both viable and lethal genes associated - Removed from analysis
C080_x + ATP_x + coa_x <-> amp_x + C080coa_x + PPi_x	SPAC56E4.04c, SPAC926.09c+SPAC4A8.11c	Both viable and lethal genes associated - Removed from analysis
accoa_c + 8 h_c + 3 malcoa_c + 6 NADPH_c -> 3 CO2_c + 4 coa_c + 2 H2O_c + 6 NADP_c + C080_c	SPAC56E4.04c, SPAC926.09c+SPAC4A8.11c	Both viable and lethal genes associated - Removed from analysis
ACP_c + malcoa_c <-> coa_c + malACP_c	SPAC926.09c+ SPAC4A8.11c	Both viable and lethal genes associated - Removed from analysis
C120ACP_c + H2O_c <-> ACP_c + h_c + C120_c	SPAC926.09c+ SPAC4A8.11c	Both viable and lethal genes associated - Removed from analysis
C140ACP_c + H2O_c <-> ACP_c + h_c + C140_c	SPAC926.09c+ SPAC4A8.11c	Both viable and lethal genes associated - Removed from analysis
C141ACP_c + H2O_c <-> ACP_c + h_c + C141_c	SPAC926.09c+ SPAC4A8.11c	Both viable and lethal genes associated - Removed from analysis
C182ACP_c + H2O_c <-> ACP_c + h_c + C182_c	SPAC926.09c+ SPAC4A8.11c	Both viable and lethal genes associated - Removed from analysis
ATP_c + fgam_c -> ADP_c + air_c + 2 h_c + pi_c	SPBC405.01	No experimental data available - Removed from analysis
ATP_c + gly_c + pram_c <-> ADP_c + gar_c + h_c + pi_c	SPBC405.01	No experimental data available - Removed from analysis
4gudbd_c + H2O_c -> 4gudbutn_c + NH4_c	SPBPB8B6.03	No experimental data available - Removed from analysis
H2O_c + iad_c -> ind3ac_c + NH4_c	SPBPB8B6.03	No experimental data available - Removed from analysis
H2O_c + pad_c -> NH4_c + pac_c	SPBPB8B6.03	No experimental data available - Removed from analysis
air_c + CO2_c <-> cair_c + h_c	SPCC1322.13	No experimental data available - Removed from analysis
fum_c + H2O_c <-> mal_c	SPCC18.18c	No experimental data available - Removed from analysis
h_n + orot5p_n -> CO2_n + ump_n	SPCC330.05c	No experimental data available - Removed from analysis
glycogen_c + H2O_c -> dext_c	SPAC23D3.14c	No experimental data available - Removed from analysis
gln_c + H2O_c + prpp_c -> glu_c + PPi_c + pram_c	SPAC4D7.08c	No experimental data available - Removed from analysis
5mTHF_n + hcys_n -> h_n + met_n + THF_n	SPAC9.09	No experimental data available - Removed from analysis
hcys_c + mhpglu_c -> hpglu_c + met_c	SPAC9.09	No experimental data available - Removed from analysis
hcys_n + mhpglu_n -> hpglu_n + met_n	SPAC9.09	No experimental data available - Removed from analysis
fum_m + H2O_m <-> mal_m	SPCC18.18c	No experimental data available - Removed from analysis
ATP_c + ser_c + trnaser_c -> amp_c + PPi_c + sertrna_c	SPAC25B8.06c, SPAC29A4.15	tRNA charging - Removed from analysis
ATP_c + ile_c + trnaile_c -> amp_c + iletrna_c + PPi_c	SPBC8D2.06	tRNA charging - Removed from analysis
ATP_c + gly_c + trnagly_c -> amp_c + glytrna_c + PPi_c	SPAC3F10.03	tRNA charging - Removed from analysis
asn_m + ATP_m + trnaasn_m -> amp_m + asntrna_m + PPi_m	SPBC1198.10c	tRNA charging - Removed from analysis
ATP_c + lys_c + trnalys_c -> amp_c + lystrna_c + PPi_c	SPBC17G9.03c	tRNA charging - Removed from analysis
ATP_m + trnatrp_m + trp_m -> amp_m + PPi_m + trptrna_m	SPAC3G9.13c	tRNA charging - Removed from analysis
arg_c + ATP_c + trnaarg_c -> amp_c + argtrna_c + PPi_c	SPBC25B2.09c	tRNA charging - Removed from analysis
ATP_c + glu_c + trnaglu_c -> amp_c + glutrna_c + PPi_c	SPAC17A5.15c	tRNA charging - Removed from analysis
ATP_m + met_m + trnamet_m -> amp_m + mettrna_m + PPi_m	SPAC27E2.06c	tRNA charging - Removed from analysis
ATP_c + trnatyr_c + tyr_c -> amp_c + PPi_c + tyrtrna_c	SPCC1672.05c	tRNA charging - Removed from analysis
ATP_c + met_c + trnamet_c -> amp_c + mettrna_c + PPi_c	SPBC17A3.04c	tRNA charging - Removed from analysis
asn_c + ATP_c + trnaasn_c -> amp_c + asntrna_c + PPi_c	SPBC1773.10c	tRNA charging - Removed from analysis
arg_m + ATP_m + trnaarg_m -> amp_m + argtrna_m + PPi_m	SPBC25B2.09c	tRNA charging - Removed from analysis
ATP_c + thr_c + trnathr_c -> amp_c + PPi_c + thrtrna_c	YIL078W	tRNA charging - Removed from analysis
ATP_m + thr_m + trnathr_m -> amp_m + PPi_m + thrtrna_m	SPAC24C9.09	tRNA charging - Removed from analysis
ATP_m + leu_m + trnaleu_m -> amp_m + leutrna_m + PPi_m	SPAC4G8.09	tRNA charging - Removed from analysis
ATP_m + lys_m + trnalys_m -> amp_m + lystrna_m + PPi_m	SPCC18.08	tRNA charging - Removed from analysis
ATP_c + cys_c + trnacys_c -> amp_c + cystrna_c + PPi_c	SPAC29E6.06c	tRNA charging - Removed from analysis
ATP_m + glu_m + trnaglu_m -> amp_m + glutrna_m + PPi_m	SPAPB1A10.11c	tRNA charging - Removed from analysis
ATP_c + trnatrp_c + trp_c -> amp_c + PPi_c + trptrna_c	SPAC2F7.13c	tRNA charging - Removed from analysis
ATP_c + gln_c + trnagln_c -> amp_c + glntrna_c + PPi_c	SPBC342.02	tRNA charging - Removed from analysis
ALA_c + ATP_c + trnaALA_c -> alatrna_c + amp_c + PPi_c	SPAC23C11.09	tRNA charging - Removed from analysis
ATP_m + ile_m + trnaile_m -> amp_m + iletrna_m + PPi_m	SPCC18B5.08c	tRNA charging - Removed from analysis
ATP_m + trnatyr_m + tyr_m -> amp_m + PPi_m + tyrtrna_m	SPCC576.06c	tRNA charging - Removed from analysis
ATP_c + leu_c + trnaleu_c -> amp_c + leutrna_c + PPi_c	SPAC26F1.13c	tRNA charging - Removed from analysis
ATP_c + his_c + trnahis_c -> amp_c + histrna_c + PPi_c	SPBC2G2.12	tRNA charging - Removed from analysis
ATP_m + his_m + trnahis_m -> amp_m + histrna_m + PPi_m	SPBC2G2.12	tRNA charging - Removed from analysis
ATP_m + phe_m + trnaphe_m -> amp_m + phetrna_m + PPi_m	SPCC736.03c	tRNA charging - Removed from analysis
ATP_c + PRO_c + trnaPRO_c -> amp_c + PPi_c + protrna_c	SPBC24C6.03 SPBC19C7.06	tRNA charging - Removed from analysis
ATP_c + trnaval_c + val_c -> amp_c + PPi_c + valtrna_c	SPAC4A8.08c SPBC1709.02C	tRNA charging - Removed from analysis
ATP_m + trnaval_m + val_m -> amp_m + PPi_m + valtrna_m	SPAC4A8.08c SPBC1709.02C	tRNA charging - Removed from analysis
ATP_c + phe_c + trnaphe_c -> amp_c + phetrna_c + PPi_c	SPAC23A1.12c+SPAC3G9.06	tRNA charging - Removed from analysis

Supporting Information 4B: List of False negative predictions from single knockout simulation using SpoMBEL1706

Metabolic reactions	Genes	False negative reconcilitation
aps_c + ATP_c -> ADP_c + h_c + paps_c	SPAC1782.11	Lethal mutation if sulfate is used as sulfur source
ATP_c + h_c + so4_c -> aps_c + PPi_c	SPBC27.08c	Lethal mutation if sulfate is used as sulfur source
paps_c + trdrd_c -> 2 h_c + pap_c + so3_c + trdox_c	SPAC13G7.06	Lethal mutation if sulfate is used as sulfur source
ac_c + ATP_c + coa_c -> accoa_c + amp_c + PPi_c	SPCC191.02c	Added reversible accoa transporter between Cytosol and Mitochondria
1ag3p_c + 0.141 C160ACP_c + 0.015 C161ACP_c + 0.043 C180ACP_c + 0.801 C181ACP_c -> pa_c + ACP_c	SPAC1851.02	Unreconciled
dump_c + mlTHF_c -> DHF_c + dtmp_c	SPAC15E1.04	Unreconciled
ATP_c + prpp_c -> PPi_c + prbATP_c	SPAC25G10.05c	Unreconciled
prfp_c -> prlp_c	SPAC3F10.09	Unreconciled
dhor-S_c + q6_m -> orot_c + q6h2_m	SPAC57A10.12c	Unreconciled
ATP_c + UDP_c <-> ADP_c + UTP_c	SPAC806.07	Unreconciled
ATP_c + dtdp_c <-> ADP_c + dttp_c	SPAC806.07	Unreconciled
dhpmp_c + H2O_c -> dhnpt_c + pi_c	SPBC14F5.13c	Unreconciled
adcho_c -> PABA_c + h_c + PYR_c	SPBC19G7.02	Unreconciled
SAM_c + 0.01 pe_c -> SAH_c + h_c + 0.01 ptdmeeta_c	SPBC26H8.03	Unreconciled
SAM_c + 0.01 ptdmeeta_c -> SAH_c + h_c + 0.01 ptd2meeta_c	SPBC337.16	Unreconciled
SAM_c + ptd2meeta_c -> SAH_c + h_c + pc_c	SPBC337.16	Unreconciled
L2aADP6sa_c + glu_c + h_c + NADPH_c <-> H2O_c + NADP_c + saccrp_c	SPBC3B8.03	Unreconciled
12dgr_c + pc_c -> 1agpc_c + triglyc_c	SPBC776.14	Unreconciled
H2O_c + meTHF_c <-> 10fTHF_c + h_c	SPBC839.16	Unreconciled
accoa_m + akg_m + H2O_m -> coa_m + h_m + hcit_m	SPBC1105.02c	Unreconciled
ATP_c + fmn_c + h_c -> fad_c + PPi_c	SPCC1235.04c	Unreconciled
ATP_c + NAD_c -> ADP_c + h_c + NADP_c	SPCC24B10.02c	Unreconciled
10fTHF_c + aicar_c <-> faicar_c + THF_c	SPCPB16A4.03c	Unreconciled
H2O_c + imp_c <-> faicar_c	SPCPB16A4.03c	Unreconciled
dmpp_c + IPPP_c -> grdp_c + PPi_c	SPBC36.06c (V), SPAC6F12.13c (L)	Both viable and lethal genes associated - Removed from analysis
UDPg_c -> 13BDglcn_c + h_c + UDP_c	SPAC24C9.07c (V), SPBC19G7.05c (L), SPCC1840.02c (L), SPAC19B12.03 (L)	Both viable and lethal genes associated - Removed from analysis
25dthpp_c + h_c + H2O_c -> 5aprbu_c + NH4_c	SPAC18B11.02c (V), SPCC4G3.16 (L)	Both viable and lethal genes associated - Removed from analysis
grdp_c + IPPP_c -> FRPP_c + PPi_c	SPBC36.06c (V), SPAC6F12.13c (L)	Both viable and lethal genes associated - Removed from analysis
g1p_c <-> G6P_c	SPBC32F12.10 (L), SPCC1840.05c (V)	Both viable and lethal genes associated - Removed from analysis
ATP_c + r5p_c <-> amp_c + h_c + prpp_c	SPCC1620.06c (L), SPAC4A8.14 (V), SPBC3D6.06c (V)	Both viable and lethal genes associated - Removed from analysis
accoa_c + 9 h_c + 3 malcoa_c + 6 NADPH_c -> 3 CO2_c + 3 coa_c + 3 H2O_c + 6 NADP_c + C080coa_c	SPAC56E4.04c (L), SPAC926.09c (V)+SPAC4A8.11c (L)	Both viable and lethal genes associated - Removed from analysis
C080coa_c + 3 h_c + malcoa_c + 2 NADPH_c -> CO2_c + coa_c + H2O_c + 2 NADP_c + C100coa_c	SPAC56E4.04c (L), SPAC926.09c (V)+SPAC4A8.11c (L)	Both viable and lethal genes associated - Removed from analysis
C100_c + 3 h_c + malcoa_c + 2 NADPH_c -> CO2_c + coa_c + H2O_c + 2 NADP_c + C120_c	SPAC56E4.04c (L), SPAC926.09c (V)+SPAC4A8.11c (L)	Both viable and lethal genes associated - Removed from analysis
C100coa_c + 3 h_c + malcoa_c + 2 NADPH_c -> CO2_c + coa_c + H2O_c + 2 NADP_c + C120coa_c	SPAC56E4.04c (L), SPAC926.09c (V)+SPAC4A8.11c (L)	Both viable and lethal genes associated - Removed from analysis
C120_c + 3 h_c + malcoa_c + 2 NADPH_c -> CO2_c + coa_c + H2O_c + 2 NADP_c + C140_c	SPAC56E4.04c (L), SPAC926.09c (V)+SPAC4A8.11c (L)	Both viable and lethal genes associated - Removed from analysis
so4_e -> so4_c	SPAC24H6.11C SPCC320.05 SPBC3H7.02 SPAC869.05C	Single gene-mutants are all viable. Deletion of all genes may result in lethal phenotype - Sulfate transporter - Removed from analysis
NH4_e <-> NH4_c	SPCPB1C11.01 SPAC664.14 SPAC2E1P3.02c	Single gene-mutants are all viable. Deletion of all genes may result in lethal phenotype - Nitrogen source transporter - Removed from analysis
3 H2O_c + h2s_c + 3 NADP_c <-> 5 h_c + 3 NADPH_c + so3_c	SPCC584.01c, SPAC10F6.01c	genes may result in lethal phenotype - Removed from analysis
h_m + 2 PYR_m -> alac-S_m + CO2_m	SPBP35G2.07, SPBC14C8.04	genes may result in lethal phenotype - Removed from analysis
C161_c + ATP_c + coa_c <-> amp_c + C161coa_c + PPi_c	SPBP4H10.11c, SPBC18H10.02	genes may result in lethal phenotype - Removed from analysis
C181_c + ATP_c + coa_c <-> amp_c + C181coa_c + PPi_c	SPBP4H10.11c, SPBC18H10.02	genes may result in lethal phenotype - Removed from analysis
dhap_c + h_c + NADH_c -> glyc3p_c + NAD_c	SPAC23D3.04c, SPBC215.05	genes may result in lethal phenotype - Removed from analysis

2pg_c <-> H2O_c + pep_c	SPBC1815.01 SPBPB21E7.01c	genes may result in lethal phenotype - Removed from analysis
g3p_c + NAD_c + pi_c <-> 13dpg_c + h_c + NADH_c	SPBC32F12.11, SPBC354.12	genes may result in lethal phenotype - Removed from analysis
IPPP_m + PNPP_m -> HXPP_m + PPi_m	SPBPJ4664.01 SPAC19G12.12	genes may result in lethal phenotype - Removed from analysis
H2O_c + prbamp_c -> prfp_c	SPBC29A3.02c SPBC1711.13	genes may result in lethal phenotype - Removed from analysis
H2O_c + prbATP_c -> h_c + PPi_c + prbamp_c	SPBC29A3.02c SPBC1711.13	genes may result in lethal phenotype - Removed from analysis
2OMHMB_m + SAM_m -> SAH_m + h_m + q6_m	SPCC4G3.04c, SPBC337.15c, SPAC19G12.11, SPBC2D10.18, SPBC146.12, SPCC162.05	Single gene-mutants are all viable. Deletion of all genes may result in lethal phenotype - Removed from analysis
2OPMP_m + O2_m -> 2OPMB_m + H2O_m	SPCC4G3.04c, SPBC337.15c, SPAC19G12.11, SPBC2D10.18, SPBC146.12, SPCC162.05	Single gene-mutants are all viable. Deletion of all genes may result in lethal phenotype - Removed from analysis
2OPMB_m + SAM_m -> 2OPMMB_m + SAH_m + h_m	SPCC4G3.04c, SPBC337.15c, SPAC19G12.11, SPBC2D10.18, SPBC146.12, SPCC162.05	Single gene-mutants are all viable. Deletion of all genes may result in lethal phenotype - Removed from analysis
2OPMMB_m + 0.5 O2_m -> 2OMHMB_m	SPCC4G3.04c, SPBC337.15c, SPAC19G12.11, SPBC2D10.18, SPBC146.12, SPCC162.05	Single gene-mutants are all viable. Deletion of all genes may result in lethal phenotype - Removed from analysis
3H45DHBZ_m + SAM_m -> 3H4H5MOBZ_m + SAH_m + h_m	SPCC4G3.04c, SPBC337.15c, SPAC19G12.11, SPBC2D10.18, SPBC146.12, SPCC162.05	Single gene-mutants are all viable. Deletion of all genes may result in lethal phenotype - Removed from analysis
acser_c + h2s_c -> ac_c + cys_c + h_c	SPBC36.04, SPAC3A12.17c	genes may result in lethal phenotype - Removed from analysis
H2O_c + histd_c + 2 NAD_c -> 3 h_c + his_c + 2 NADH_c	SPBC1711.13 SPBC29A3.02C	genes may result in lethal phenotype - Removed from analysis
2 ATP_c + gln_c + H2O_c + hco3_c -> 2 ADP_c + cbp_c + glu_c + 2 h_c + pi_c	SPAC22G7.06c SPAC16.03c SPBC56F2.09c	Single gene-mutants are all viable. Deletion of all genes may result in lethal phenotype - Removed from analysis
2obut_m + h_m + PYR_m -> 2ahbut_m + CO2_m	SPBP35G2.07, SPBC14C8.04	genes may result in lethal phenotype - Removed from analysis
argsuc_c <-> arg_c + fum_c	SPBC1539.03c, SPBC1773.14	genes may result in lethal phenotype - Removed from analysis
asp_c + cbp_c -> cbasp_c + h_c + pi_c	SPAC22G7.06c SPAC16.03c SPBC56F2.09c	Single gene-mutants are all viable. Deletion of all genes may result in lethal phenotype - Removed from analysis
dhor-S_c + H2O_c <-> cbasp_c + h_c	SPAC22G7.06c, SPAC16.03c	genes may result in lethal phenotype - Removed from analysis
akg_c + phe_c <-> glu_c + PHPYR_c	SPBC1773.13, SPAC56E4.03, SPCC569.07	Single gene-mutants are all viable. Deletion of all genes may result in lethal phenotype - Removed from analysis
23dhmp_m -> 3mop_m + H2O_m	SPAC17G8.06c	Potentially dependent on media condition - Removed from analysis
ser_c + THF_c <-> gly_c + H2O_c + mlTHF_c	SPAC24C9.12c	Potentially dependent on media condition - Removed from analysis
4HBZ_m + HXPP_m -> O4HBZ_m + PPi_m	SPAC56F8.04c	Potentially dependent on media condition - Removed from analysis
h_c + pphn_c -> CO2_c + H2O_c + PHPYR_c	SPBC30D10.16	Potentially dependent on media condition - Removed from analysis
akg_m + ile_m <-> 3mop_m + glu_m	SPBC428.02c	Potentially dependent on media condition - Removed from analysis
akg_c + leu_c <-> 4mop_c + glu_c	SPBC428.02c	Potentially dependent on media condition - Removed from analysis
3c4mop_c + h_c -> 4mop_c + CO2_c	SPBC428.02c	Potentially dependent on media condition - Removed from analysis
akg_m + val_m <-> 3mob_m + glu_m	SPBC428.02c	Potentially dependent on media condition - Removed from analysis

asp_c + ATP_c + citr_c <-> amp_c + argsuc_c + h_c + PPi_c	SPBC428.05c	Potentially dependent on media condition - Removed from analysis
orot5p_c + PPi_c <-> orot_c + prpp_c	SPBC725.15	Potentially dependent on media condition - Removed from analysis
H2O_c + NAD_c + saccrp_c <-> akg_c + h_c + lys_c + NADH_c	SPAC227.18	Potentially dependent on media condition - Removed from analysis
eig3p_c -> H2O_c + imacp_c	SPBC21H7.07c	Potentially dependent on media condition - Removed from analysis
hcit_m <-> b124tc_m + H2O_m	SPAC343.16	Potentially dependent on media condition - Removed from analysis
3c2hmp_c <-> 2ippm_c + H2O_c	SPAC9E9.03	Potentially dependent on media condition - Removed from analysis
2ippm_c + H2O_c <-> 3c3hmp_c	SPAC9E9.03	Potentially dependent on media condition - Removed from analysis
3c2hmp_c + NAD_c -> 3c4mop_c + h_c + NADH_c	SPBC1A4.02c	Potentially dependent on media condition - Removed from analysis
accoa_c + ser_c -> acser_c + coa_c	SPAC1039.08	Potentially dependent on media condition - Removed from analysis
alac-S_m + h_m + NADPH_m -> 23dhmb_m + NADP_m	SPBC56F2.12	Potentially dependent on media condition - Removed from analysis
2ahbut_m + h_m + NADPH_m -> 23dhmp_m + NADP_m	SPBC56F2.12	Potentially dependent on media condition - Removed from analysis
23dhmb_m -> 3mob_m + H2O_m	SPBC56F2.12	Potentially dependent on media condition - Removed from analysis
SAH_c + H2O_c -> adn_c + hcys_c	SPBC8D2.18c	Potentially dependent on media condition - Removed from analysis
cbp_c + ORN_c -> citr_c + h_c + pi_c	SPAC4G9.10	Potentially dependent on media condition - Removed from analysis
glu_c + imacp_c -> akg_c + hisp_c	SPBC11B10.02c	Potentially dependent on media condition - Removed from analysis
